# Radical Borylation of Alkyl Bromides by Photoinduced Halogen‐Atom Transfer

**DOI:** 10.1002/anie.202510162

**Published:** 2025-08-04

**Authors:** Cullen R. Schull, Matthew J. McGill, Ángel Renteria‐Gómez, Poulami Mukherjee, Samuel B. Tyndall, Aaron H. Shoemaker, Michael R. Wasielewski, Osvaldo Gutierrez, Karl A. Scheidt

**Affiliations:** ^1^ Department of Chemistry Northwestern University 2145 Sheridan Road Evanston IL 60208 USA; ^2^ Department of Chemistry and Biochemistry University of California 607 Charles E. Young Drive, East Los Angeles Los Angeles CA 90095 USA

**Keywords:** Alkyls, Borylation, Halogens, Photochemistry, Radicals

## Abstract

Alkyl organoboron compounds are versatile synthons in organic synthesis, enabling rapid access to a variety of carbon─carbon and carbon‐heteroatom bonds. As such, strategies to efficiently access carbon‐boron bonds from simple chemical feedstocks are highly desirable. The radical borylation of alkyl bromides presents an attractive approach. However, the activation of alkyl bromides typically requires strong reductants or transition‐metal catalysts. Herein, we report a metal‐free radical borylation strategy of various alkyl bromides utilizing a photoinduced silyl radical to mediate a halogen‐atom transfer process. This method demonstrates broad utility and functional group tolerance among various primary, secondary, and tertiary unactivated alkyl bromides and can facilitate the functionalization of pharmaceutically relevant motifs. Mechanistic and computational studies support a radical‐chain pathway involving a silyl radical‐mediated halogen‐atom transfer.

## Graphical abstract

The photoinduced borylation of alkyl bromides via halogen atom transfer is reported. The utility of this strategy is demonstrated with broad functional group tolerance in the borylation of various primary, secondary, and tertiary alkyl bromides. Computational studies provide mechanistic insights into the radical‐chain pathway.

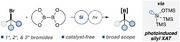

Alkyl boronic esters are highly valuable synthetic intermediates due to their use as substrates in metal‐catalyzed coupling reactions and their efficient derivatizations into other valuable functional groups.^[^ ^1^, ^2^, ^3^, ^4^, ^5^ ^]^ Beyond their notable synthetic utility, boronic acids and ester derivatives have received significant attention for various applications including in drug development (e.g., bortezomib, vaborbactam, tavaborole)^[^ ^6^, ^7^, ^8^ ^]^ and material science.^[^ ^9^, ^10^, ^11^ ^]^ As a result, the development of strategies to access boron‐containing compounds from feedstock chemicals is of significant importance. Alkyl halides are one of the most abundant building blocks in organic chemistry. Traditionally, alkyl halides have been converted into alkyllithium or alkylmagnesium reagents and subsequently trapped with electrophilic alkyl borates.^[^ ^5^, ^12^ ^]^ More recently, transition‐metal catalyst (e.g., Fe,^[^ ^13^ ^]^ Mn,^[^ ^14^ ^]^ Pd,^[^ ^15^ ^]^ Ni,^[^ ^16^ ^]^ Cu,^[^ ^17^, ^18^, ^19^ ^]^ Zn^[^ ^20^ ^]^) complexes have been usefully employed in alternative approaches to access alkyl boron compounds. However, these strategies require a metal catalyst, basic conditions, and often necessitate various ligands to promote these desired transformations. Metal‐free borylation strategies could offer a complementary and potentially milder route to afford these materials.

Recent interest in photochemical transformations has sparked developments in radical‐based borylations utilizing diboron sources as radical acceptors.^[^ ^21^, ^22^, ^23^ ^]^ Early pioneering examples include the borylation of activated carboxylic acids, as *N*‐hydroxyphthalimide esters, independently developed by the groups of Aggarwal,^[^ ^24^ ^]^ Baran,^[^ ^25^ ^]^ and Li.^[^ ^26^ ^]^ Shortly thereafter, radical borylation strategies were extended towards activated amines^[^ ^27^, ^28^, ^29^ ^]^ and alcohols.^[^ ^30^, ^31^, ^32^ ^]^ However, these strategies depend on the preactivation of functional groups for single‐electron activation and subsequent radical bond scission for radical addition into suitable diboron species (Figure 1a). While this framework has been successful for a range of functional groups, it can be challenging as well as potentially cumbersome to append a redox‐active functionality to activate alkyl halides.

**Figure 1:**
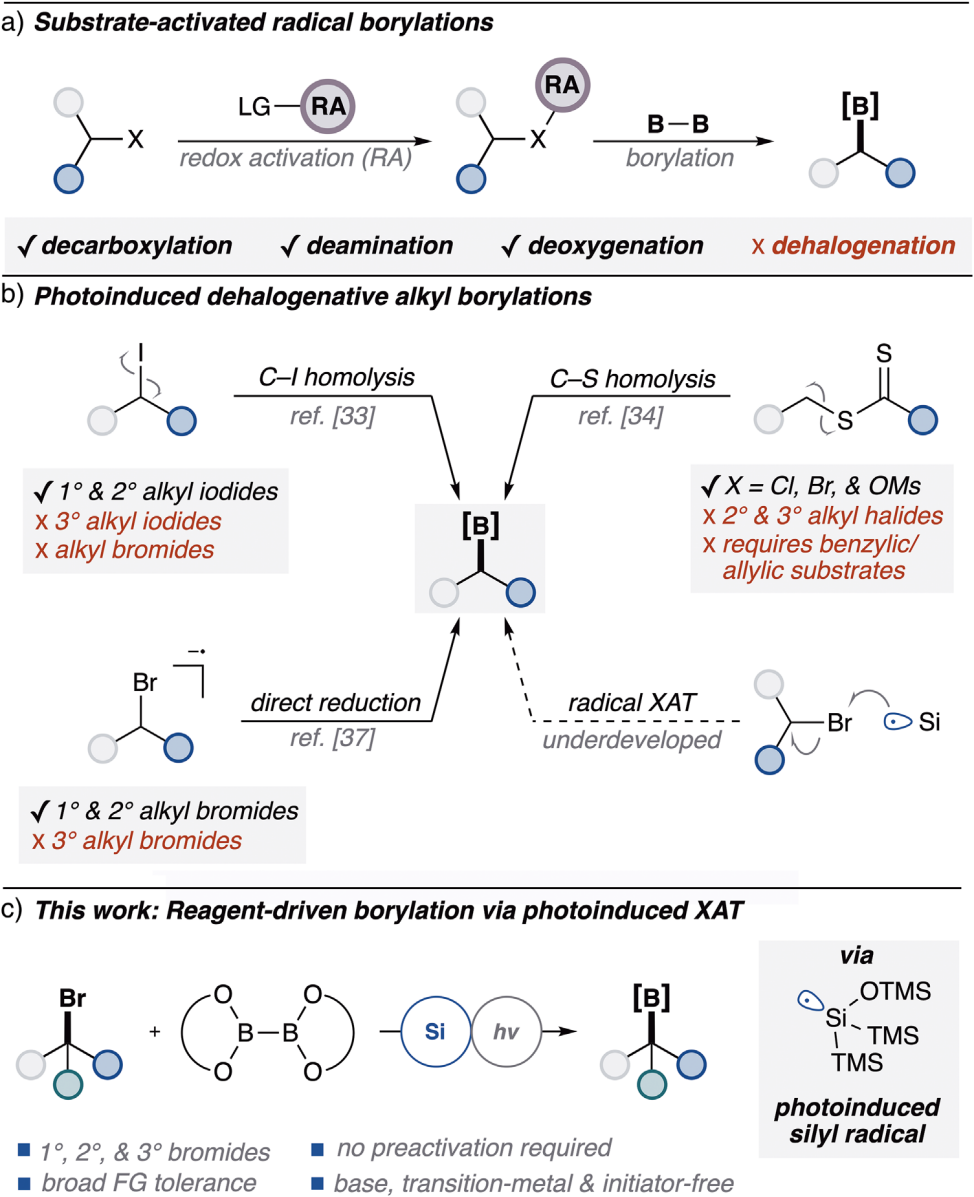
a) Prior photoinduced radical borylation strategies of functional groups; b) Limitations of previous photoinduced dehalogenative borylations; c) XAT radical borylation of alkyl bromides.

Early photoinduced dehalogenative borylation strategies were driven by photolytic cleavage of weak bonds via homolysis. The Studer group developed the radical borylation of alkyl iodides via direct photoinduced C─I bond homolysis.^[^ ^33^ ^]^ In 2019, the Melchiorre group reported a nucleophilic organocatalytic method to activate alkyl halides via an S_N_2 process to undergo subsequent photoinduced C–S bond homolysis.^[^ ^34^ ^]^ Similarly, sodium iodide was found to sufficiently convert alkyl halides to alkyl iodides in situ for photoinduced borylation.^[^ ^35^ ^]^ An alternative strategy to bond homolysis is the development of super electron donors (SEDs) capable of direct single‐electron reduction of alkyl halides. For example, the Mo group disclosed an in situ generated stoichiometric boronate complex capable of reducing alkyl iodides.^[^ ^36^ ^]^ The Jiao group later reported the first pyridine‐catalyzed boronate complex with the capability of extending towards activating alkyl bromides.^[^ ^37^ ^]^ Despite these advancements, SED methods require highly basic conditions, rendering these strategies incompatible with tertiary substrates due to base‐promoted elimination. To the best of our knowledge, there are currently no photoinduced dehalogenative radical borylations amenable to primary, secondary, and tertiary unactivated alkyl bromides.^[^ ^38^ ^]^

Based on previous work in photoinduced radical borylation (Figure 1), we considered a *reagent‐driven* activation approach,^[^ ^39^ ^]^ in which a functionalized silane could activate alkyl bromides via silyl radical halogen‐atom transfer (XAT). In this scenario, we hypothesized that an in situ generated nucleophilic silicon radical could undergo a fast, irreversible radical abstraction of a carbon‐bromide bond from alkyl bromides to generate C(sp^3^) radicals. A subsequent formal homolytic substitution with a compatible diboron reagent would afford the desired borylated products. We hypothesized that this XAT pathway would obviate the use of highly reducing conditions and potentially broaden the scope of radical borylations towards more complex molecules. In this realm, silyl radicals have served as XAT reagents in many recent photochemical manifolds; however, these reagents typically require redox activation or radical initiators for desired reactivity.^[^ ^40^, ^41^, ^42^ ^]^ Inspired by the recent work in halogen atom transfer by the MacMillan group,^[^ ^43^, ^44^, ^45^ ^]^ we posited *N*‐siloxyphthalimides could serve as a suitable silyl radical precursor for the activation of alkyl bromides. However, instead of photoredox activation, we speculated that the phthalimide‐tethered silane could coordinate with the diboron species to activate the silyl radical for XAT. This reaction pathway contrasts traditional XAT reaction mechanisms and represents a novel photoexcitation pathway for borylation.

We commenced our investigations with bromide substrate **1a**, halogen atom transfer reagent **2**, and B_2_cat_2_ in *N, N*‐dimethylacetamide (DMA) under blue light irradiation at 427 nm (Table 1, entry 1). Gratifyingly, these conditions resulted in the formation of boronic ester **3a’** in 83% ^1^H NMR yield upon transesterification with pinacol. An evaluation of solvents indicated the necessity of DMA as a Lewis basic solvent. Notably, Lewis basic solvents have previously demonstrated success in other radical borylation manifolds by Aggarwal and others.^[^ ^24^, ^27^, ^28^, ^30^, ^31^ ^]^ Interestingly, the use of dimethylformamide (DMF) resulted in a significant decrease in reactivity (Table 1, entry 2). The replacement of B_2_cat_2_ with bis(pinacolato)diborane (B_2_Pin_2_) resulted in a significant decrease in yield (Table 1, entry 4). While other silane reagents have previously established photoinduced initiations,^[^ ^46^, ^47^ ^]^ tris(trimethylsilyl)silane and tris(trimethylsilyl)silanol were ineffective in this manifold (Table 1, entries 5 and 6), demonstrating the necessity of a redox activated XAT reagent (**2**). Increasing the amount of **2** exhibited a positive correlation in yield, in which employment of three equivalents of **2** furnished an excellent 88% yield as measured by ^1^H NMR spectroscopy (Table 1, entry 7). Interestingly, heating the reaction at 65 °C resulted in an 8% yield (Table 1, entry 10).  A trace amount of product was observed in the absence of light, while no product was observed without **2** (Table 1, entries 10 and 11).

**Table 1:** Optimization of reaction conditions and control experiments.

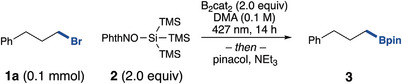		
Entry	Deviation from standard	Yield (%)^a)^
1	None	**83**
2	DMF instead of DMA	24
3	MeCN instead of DMA	15
4	B_2_pin_2_ instead of B_2_cat_2_	8
5	(TMS)_3_SiH instead of **2**	trace
6	(TMS)_3_SiOH instead of **2**	trace
7	x equiv. 2 (1.0, 1.5, 3.0)	48, 59, 88
8	390 nm instead of 427 nm	73
9	456 nm instead of 427 nm	73
**————————– reaction controls ————————–**		
10	no light, 65 °C	8
11	no light	5
12	no 2	0

^a)^ ^1^H NMR (500 MHz) yield of unpurified mixture with 1,3,5‐trimethoxybenzene as an internal standard.

With optimized conditions in‐hand, we explored the scope of this radical debrominative borylation methodology (Figure 2). First, the optimized substrate **1a** was isolated in 78% yield following workup with the more chromatographically stable B(E)pin (**3a**).^[^ ^48^ ^]^ This methodology was amenable to a range of unactivated primary bromides, including a reducible ester functionality in substrate **3b**, an acetal in **3d**, and an unprotected indole in **3** **g**. To demonstrate applicability in late‐stage functionalization, a derivative of oxaprozin was successfully borylated in satisfactory 78% yield to furnish **3** **h**. Activated benzylic bromides were well tolerated (**3i** & **3j**), as well as probenecid derivative **3k** in moderate to excellent yields. Secondary bromides of Boc‐protected azetidine, pyrrolidine, piperidine, and azepane (**3l‐3o**) were borylated in moderate to good yields. Saturated heterocycles were also amenable to this system (**3p‐3q**) as well as secondary alkyl bromides (**3r**). Cyclic alkyl bromides were also successfully borylated (**3s**‐**3 u**).  Additionally, proxyphylline derivative **3v** was prepared in 51% yield.

**Figure 2:**
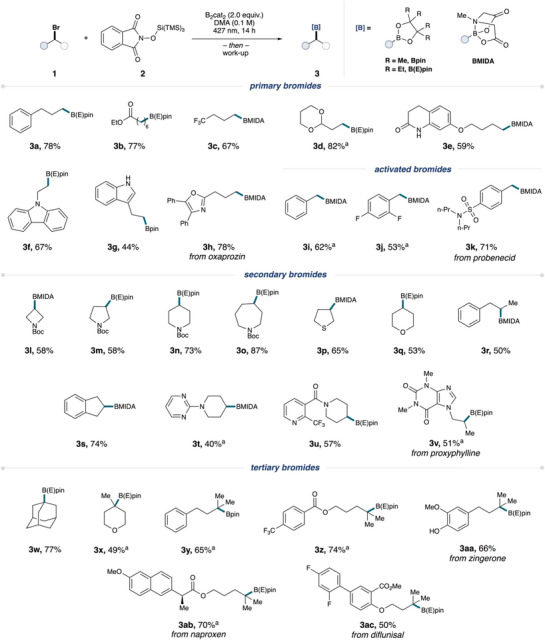
Substrate scope for the borylation of alkyl bromides. Reported yields are isolated yields. ^a)^Performed with 3.0 equiv of B_2_cat_2._

We next turned our attention towards tertiary bromide substrates. A conformationally restricted tertiary alkyl bromide was successfully borylated in 77% yield (**3 w**). A tertiary pyran substrate was amenable (**3x**), while acyclic tertiary alkyl bromides, including those derived from anti‐inflammatory drugs naproxen and diflunisal, were also tolerated in satisfactory yields (**3y‐3ac**). Inspired by the work in alkyl chloride borylation,^[^ ^49^ ^]^ we tested alkyl chlorides with our modified conditions (3.0 equiv B_2_cat_2_). Benzyl chloride was compatible with a 43% ^1^H NMR yield. Unfortunately, 3‐chloropropylbenzene was not tolerated (S27). Additional substrates and unsuccessful substrates can be found in the Supporting Information.

To highlight the utility of this process, we aimed to demonstrate a subset of downstream modifications of alkyl boronic esters. To commence our explorations, **3o** was successfully prepared on a 1.0 mmol scale in moderate yield (Figure 3a). Amination followed by deprotection of **3o** successfully obtained **4** under Morken amination conditions^[^ ^50^ ^]^ in moderate yields. Next, a Matteson homologation and subsequent oxidation of **3o** furnished **5**. A Zweifel olefination with an alkenyl Grignard furnished **6** in 49% yield. Lastly, we sought to apply our strategy towards the formation of *N*‐saturated heterocycles and further support a radical‐based mechanism. Subjecting an intramolecular substrate to our standard conditions provided the 5‐exo‐trig cyclized borylated pyrrolidine **7** in 53% yield.

**Figure 3:**
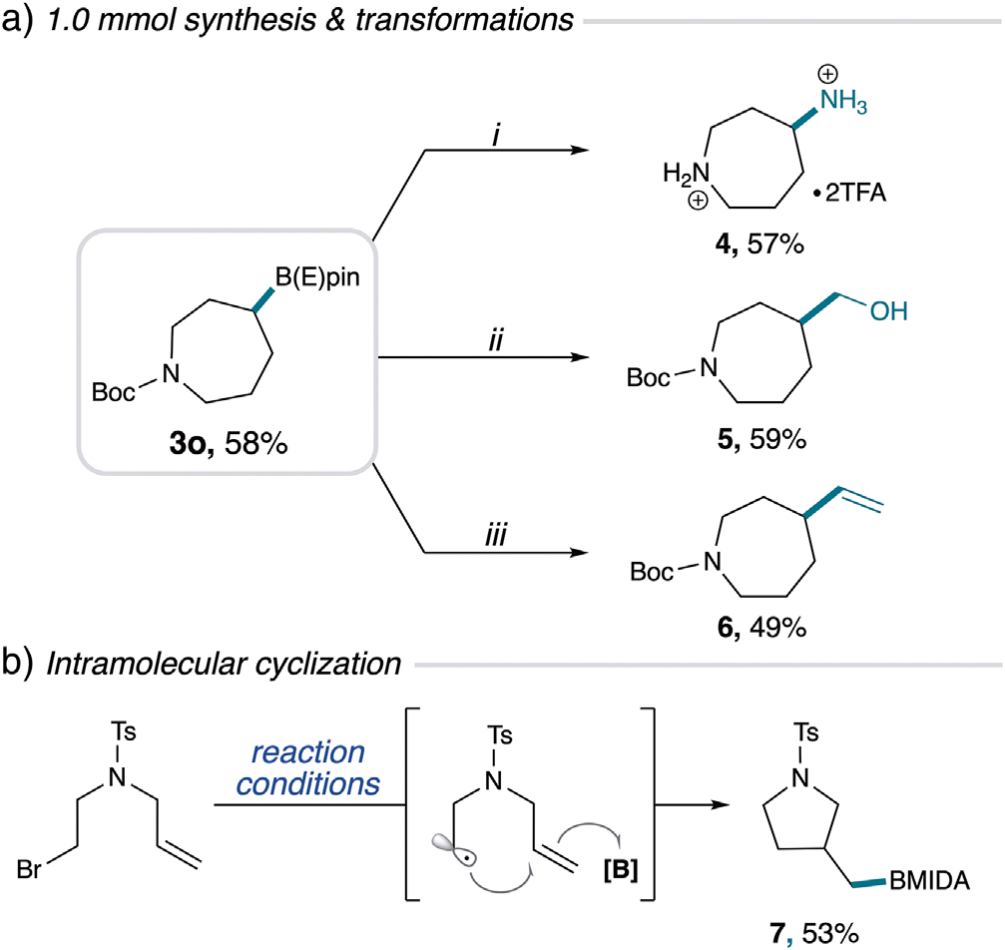
Scale‐up, product diversification, and intramolecular radical cascade example. See Supporting Information for additional experimental details. i) 1) ^t−^BuOK, MeONH_3_Cl then Boc_2_O. ii) LiCH_2_Br then NaOH/H_2_O_2_. iii) CH_2 _= CH_2_MgBr, then NaOMe/I_2_.

To gain a mechanistic understanding of this reaction, several experiments were conducted. UV/Vis absorption spectroscopy did *not* exhibit a bathochromic shift with B_2_cat_2_ and **2** in DMA, but rather a hypochromic shift, thereby excluding an electron‐donor acceptor complex as a plausible mechanistic pathway (Figure 4a). Furthermore, B_2_cat_2_ exhibited a distinctive second boron species upon addition of DMA, consistent with previously reported photoinduced borylations,^[^ ^24^ ^]^ as evidenced by ^11^B NMR spectroscopy and cyclic voltammetry (See Supporting Information). Fluorescence quenching experiments were performed to determine if photoexcited **2** could undergo SET with B_2_cat_2_ to promote radical fragmentation of **2** in accordance to a previous report by Aggarwal.^[^ ^39^ ^]^ However, 2* did not show quenching upon addition of B_2_cat_2_ and instead increased fluorescence, excluding the potential 2* SET mechanistic pathway with B_2_cat_2_. Next, a radical trapping experiment was conducted using TEMPO (2.0 equiv) to support the involvement of radical intermediates. The TEMPO adduct **8** was detected by HRMS, and the formation of **3‐Bcat** was not observed by HRMS or GC‐MS demonstrating the complete inhibition of the radical‐mediated process (Figure 4c). A radical trapping experiment with 1,1‐diphenylethylene supports the formation of light‐induced silyl radical formation with adducts **9** and **10** detected by HRMS (Figure 4d; See Supporting Information for comprehensive radical trapping experiments). Finally, quantum yield experiments revealed a value of Φ = 2.45, signifying a plausible radical chain pathway (Figure 4e).

**Figure 4:**
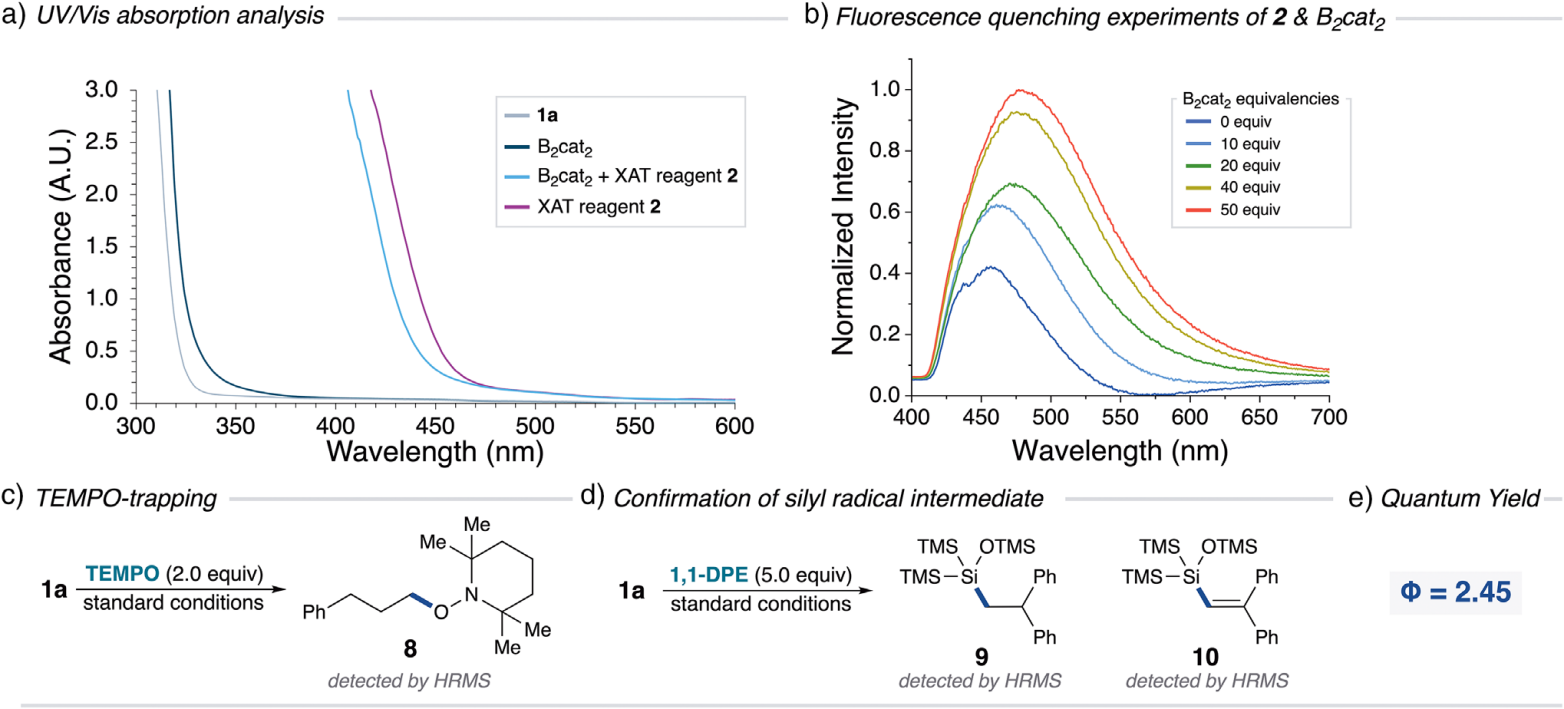
Mechanistic investigations. a) UV/Vis spectropscopy; b) Fluorescence quenching studies of 2 (6 mM) with varying concentrations of B_2_cat_2_ (0–300 mM); c) TEMPO trapping experiment; d) Diphenylethylene trapping experiment; e) Quantum yield measurement.

Based on our experimental studies and previous reports, the proposed reaction pathway is depicted in Figure 5. This pathway is supported by dispersion‐corrected density functional theory (DFT) calculations (see SI for additional details). The first step of the mechanism is likely the formation of complex **^1^I** from B_2_cat_2_, DMA, and **2**. Notably, it is plausible for the formation of complex 1I from 2DMA•B_2_cat_2_, but it is thermodynamically less favorable (barrier of 2.6 kcal mol^Ȓ1^; Figure S30). Photoexcitation of complex **^1^I** under irradiation with 427 nm wavelength light, followed by intersystem crossing (ISC) to reach the triplet excited state **^3^I,** with an excitation energy of (ΔE_T_ = 25.1 kcal mol^Ȓ1^). In turn, **^3^I** can undergo facile B‐B homolysis via a lower energy barrier (ΔG^‡^ = 7.1 kcal mol^Ȓ1^) to generate the DMA‐coordinated radical **II** (downhill by 2.3 kcal mol^Ȓ1^) and radical **A** that could be a source of siloxy radical **IV** (Figure S28). An alternative direct photoexcitation and fragmentation of **2** pathway is also likely operative in some capacity, based on radical trapping experiments (See Figure 4c and Supporting Information). However, the barrier for photoexcitation is *significantly* higher in energy than the proposed complex **I** (52.0 vs. 25.1 kcal mol^Ȓ1^; Figure S28).

**Figure 5:**
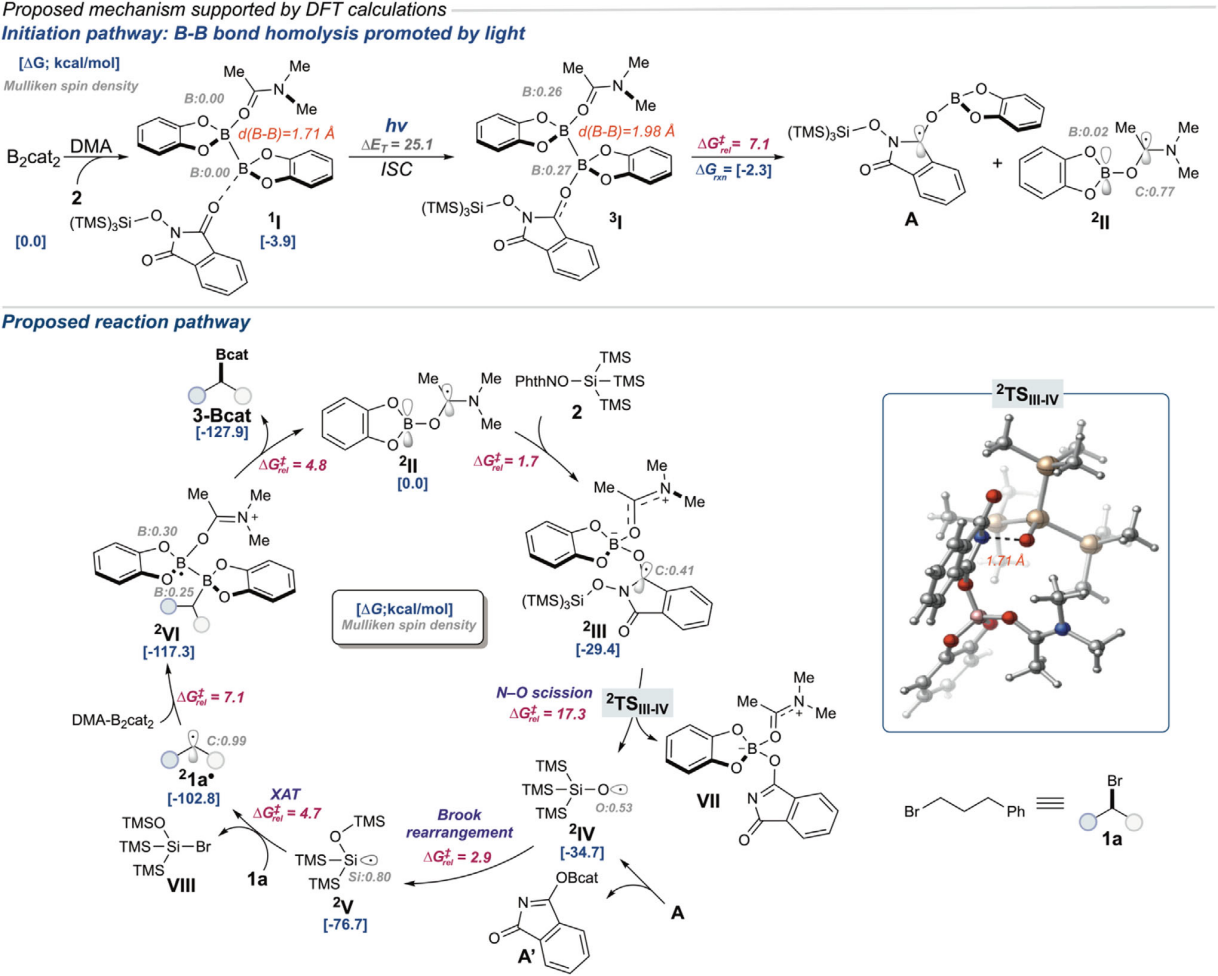
Proposed mechanism calculated at uB3LYP‐D3/def2‐SVP‐CPCM(DMA) level of theory. All energies are reported in kcal mol^Ȓ1^.

Additionally, an in‐depth examination of the boryl radical **^2^II** reveals a DMA‐ligated radical featuring a nucleophilic carbon radical site alongside an electrophilic boron site attributed to its empty p orbital. In turn, the feasibility of the electrophilic boron site in **^2^II** promotes a fast and irreversible addition onto the carbonyl oxygen in **2** (barrier of 1.7 kcal mol^Ȓ1^ and downhill by 29.4 kcal mol^Ȓ1^ concerning **^2^II**), delivering the radical intermediate **III**. Mulliken spin density analysis of **^2^III** indicates that the spin density is primarily localized on the carbonyl carbon, which further promotes the irreversible N‐O bond fragmentation with a barrier of 17.3 kcal mol^Ȓ1^ (via **^2^TS_III‐IV_**), generating a siloxy radical **^2^IV** and the simultaneous release of **VII** (For alternative mechanisms, see Figure S28). After forming a siloxy radical intermediate **^2^IV**, this can undergo an irreversible radical Brook rearrangement (downhill by 42.0 kcal mol^Ȓ1^)^[^ ^51^ ^]^ to form the stabilized Si‐centered radical **V** (barrier of 2.9 kcal mol^Ȓ1^, Figure S25). In turn, formation of radical **^2^1a•** is thermodynamically favored (downhill by 26.1 kcal mol^Ȓ1^) through a halogen atom transfer event (XAT) between silyl radical **^2^** **V** and the alkyl bromide **1a** via a lower energy barrier of 4.7 kcal mol^Ȓ1^. Then, calculations revealed that the alkyl radical **^2^1a•** can add to DMA‐B_2_cat_2_ complex (downhill by 14.5 kcal mol^Ȓ1^, Figure S26) via a small barrier of 7.1 kcal mol^Ȓ1^ to form the radical **^2^VI**. This process eventually leads to facile B‐B homolysis (barrier of only 4.8 kcal mol^Ȓ1^) leading to the desired borylated product **3** with concomitant release of **^2^II,** which can further restart the catalytic cycle. Notably, the energy necessary for B‐B homolysis of **^2^VI** in the absence of DMA is notably greater (4.8 vs. 17.4 kcal mol^Ȓ1^, Figure S26), which further supports the importance of DMA coordination (Table 1) in weakening the B─B bond consistent with previous reports.^[^ ^52^ ^]^

A photoinduced borylation of alkyl halides using primary, secondary, and tertiary bromides has been developed. This strategy offers a mild alternative to previous borylation strategies by obviating the use of bases, metal catalysts, and strong reductants. The utility of this method was demonstrated through a diverse substrate scope, including the borylation of pharmaceutical derivatives and access to valuable downstream diversifications. Our combined experimental and computational studies support a radical‐chain pathway involving an XAT from alkyl bromides by a silyl radical generated by light‐induced B─B bond homolysis, providing a mechanistically distinct approach to accessing valuable alkyl boronic esters from simple haloalkanes.

## Conflict of Interests

The authors declare no conflict of interest.

## Supporting information



Supporting Information

## Data Availability

The data that support the findings of this study are available from the corresponding author upon reasonable request.
