# Supplementary material for: Radical Borylation of Alkyl Bromides by Photoinduced Halogen‐Atom Transfer
Source: Angew Chem Int Ed Engl. 2025 Aug 4;64(38):e202510162. doi: 10.1002/anie.202510162 (PMC12435432; doi:10.1002/anie.202510162)
Supplement: Supplementary file 1 — Supporting Information [file ANIE-64-e202510162-s001.pdf]

Supporting Information

Table of Contents

General Information S2

List of Compounds S3

General Procedures & Characterization of New Compounds S5

General procedure A: Preparation of primary and secondary alkyl bromides. S5

General procedure B: Preparation of tertiary alkyl bromides. S5

Reaction Optimization S10

General procedure C: Photoinduced Borylation S10

Further Transformations S25

Additional and Unsuccessful Substrates S27

Radical Trapping experiments S28

Discussion S29

UV-Vis studies S30

^{11}B NMR study S35

Synthesis of $NaBcat_2$ S42

Fluorescence Quenching studies S43

Cyclic Voltammetry studies S44

Computational Details S47

Selected NMR S100

References S162

General Information

All reactions were carried out under nitrogen atmosphere in flame-dried glassware with magnetic stirring. All solvents were purified by passing through a bed of activated alumina, dried over 3Å molecular sieves and then degassed using freeze-pump-thaw method (3 cycles). Purification of reaction products was carried out by flash chromatography on Biotage Isolera 4 systems. Analytical thin layer chromatography was performed on EM Reagent 0.25 mm silica gel 60-F plates. Visualization was accomplished with UV light or CAM staining. ^{1}H NMR spectra were recorded on a Bruker AVANCE III 500 MHz w/ DCH Cryoprobe (500 MHz) spectrometer and are reported in ppm using solvent as an internal standard ($CDCl_3$ at 7.26 ppm, acetonitrile-$_{d3}$ at 1.94, DMSO-$_{d6}$ at 2.50 ppm). Data are reported as (ap = apparent, s = singlet, d = doublet, t = apparent triplet, q = quartet, m = multiplet; coupling constant(s) in Hz; integration. Proton-decoupled ^{13}C NMR spectra were recorded on a Bruker AVANCE III 500 MHz w/ DCH Cryoprobe (126 MHz) spectrometer and are reported in ppm using solvent as an internal standard ($CDCl_3$ at 77.16 ppm, acetonitrile-$_{d3}$ at 1.32 ppm, DMSO-$_{d6}$ at 39.52 ppm). ^{19}F NMR and ^{11}B NMR spectra were recorded on a Bruker AVANCE III HD 500 MHz w/ BBO Prodigy Probe. Mass spectra were obtained on a WATERS Acquity-H UPLC-MS with a single quad detector (ESI), or an Agilent 7890 gas chromatograph equipped with a 5975C single quadrupole EI-MS. High-resolution mass spectrometry (HRMS ESI/TOF) was obtained using an Agilent 6201 MSLC-TOF (ESI), Bruker IMPACT II (APCI), or Agilent 7250 GC/QTOF. FTIR data was collected at room temperature on a Bruker Tensor 37 FTIR Spectrometer equipped with a Mid IR detector and KBr beam splitter in attenuated total reflectance (ATR) mode in the range of 4000 to 600 cm−1, averaged over 16 scans. The OPUS software was used for the data acquisition. UV-Vis data was obtained on a Cary 60 UV-Vis spectrophotometer (Agilent). Quantum yield data was obtained using Kessil PR160L 427 nm lights. Fluorescence emission spectra were obtained at right-angle detection on a Horiba Nanolog spectrofluorimeter (FL3-2iHR/iHR). All photochemical reactions were performed using Kessil PR160L 427 nm lights. All photochemical reactions were set up in two-dram vials with PTFE lined solid caps (Chemglass CG-4900).

List of Compounds

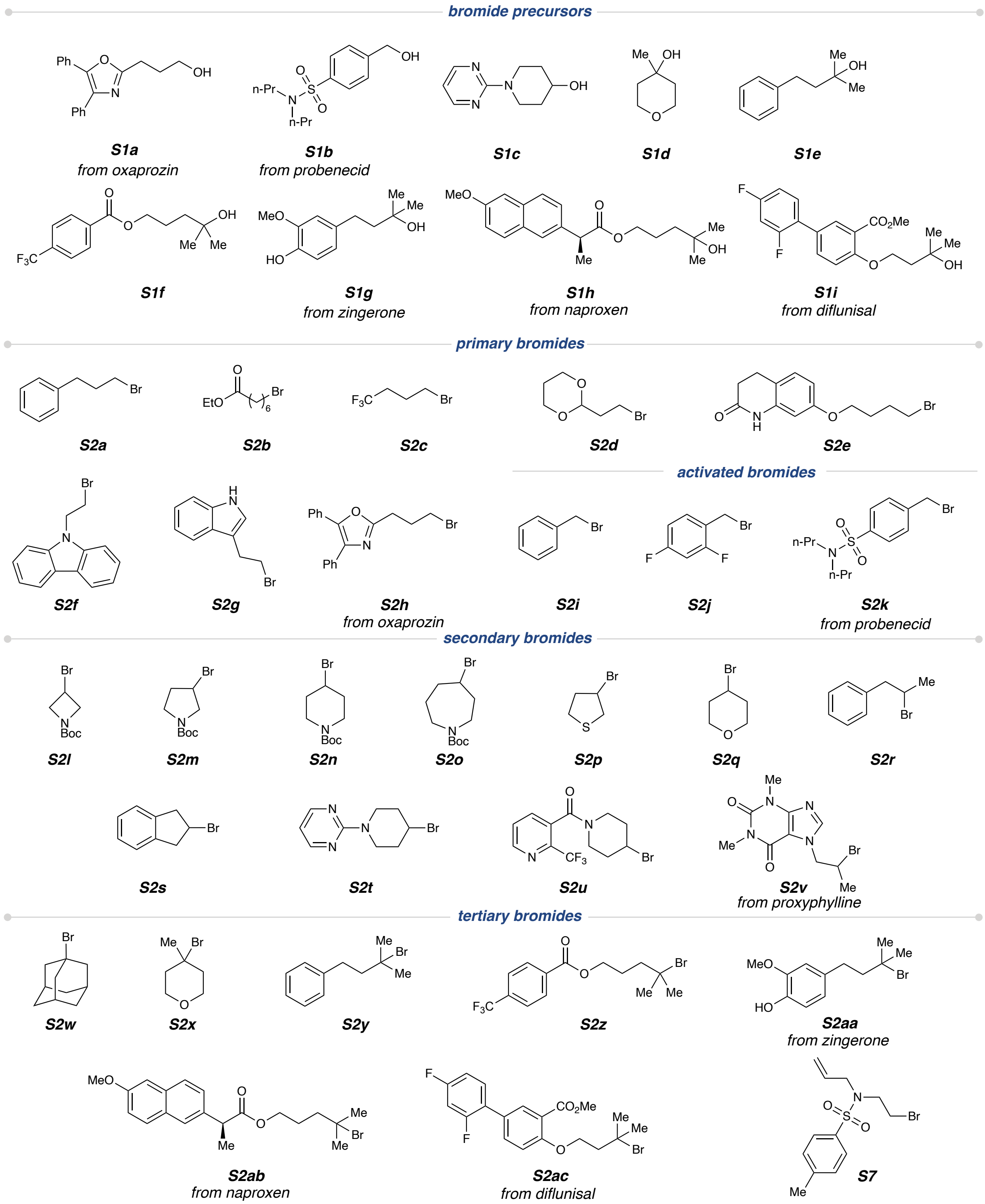

S1a[1], **S1b**[1], **S1c**[2], **S1d**[3], **S1e**[4], **S1g**[5], **S1i**[6] were prepared from reported literature procedures.

S2a, **S2b**, **S2c**, **S2d**, **S2e**, **S2i**, **S2j**, **S2l**, **S2m**, **S2n**, **S2p**, **S2q**, **S2r**, **S2s** & **S2w** were obtained from commercial sources.

S2f[7], S2g[8], S2k[9], S2v[10], S2x[11], S2y[12], S2ac[6] & S7[13] were prepared from reported literature procedures.

1° boronic esters

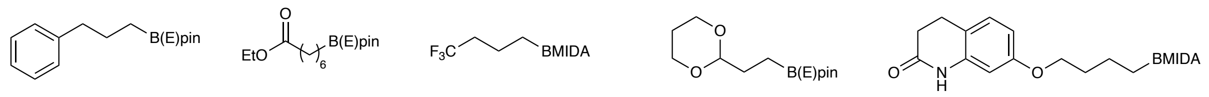

3a ***3b*** ***3c*** ***3d*** ***3e***

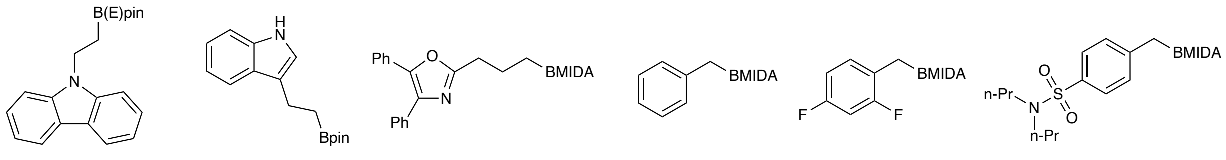

3f ***3g*** ***3h*** ***3i*** ***3j*** ***3k***

2° boronic esters

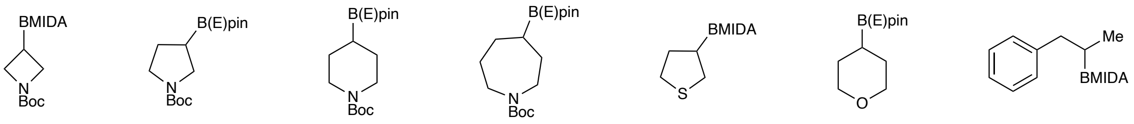

3l ***3m*** ***3n*** ***3o*** ***3p*** ***3q*** ***3r***

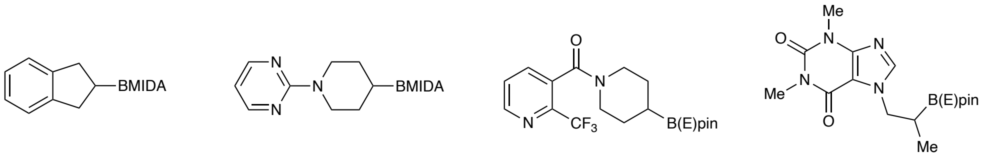

3s ***3t*** ***3u*** ***3v***

3° boronic esters

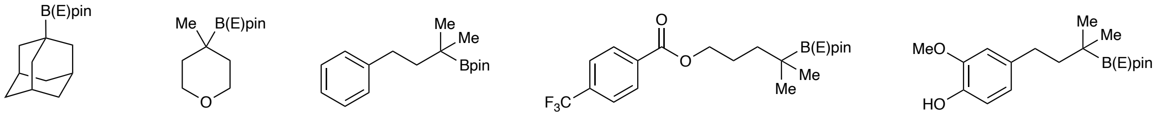

3w ***3x*** ***3y*** ***3z*** ***3aa***

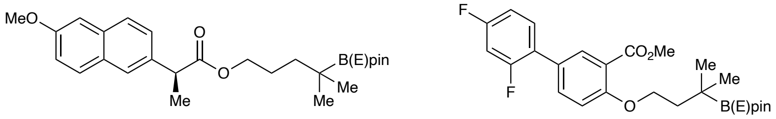

3ab ***3ac***

transformations & intramolecular cyclization

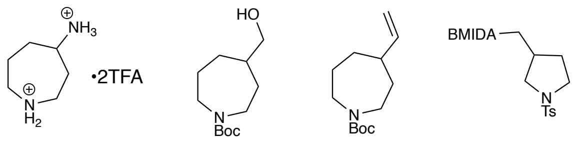

4 ***5*** ***6*** ***7***

General Procedures & Characterization of New Compounds

General procedure A: Preparation of primary and secondary alkyl bromides. Phosphorus tribromide (1 M in DCM, 1 equiv) was added slowly at 0 °C to the respective alcohol substrate in anhydrous DCM (0.5 M) and the mixture was stirred for 2 h. The crude reaction mixture was then diluted with EtOAc, followed by aqueous workup. The organic layer was dried with Na_2SO_4, filtered, and concentrated *in vacuo*. The crude organic residue was purified by flash column chromatography.

General procedure B: Preparation of tertiary alkyl bromides. Tertiary bromides were prepared according to Fu and co-workers.[11] The alcohol (neat or a solution in a minimal amount of DCM) was added to a solution of LiBr (2.0 equiv) in 48 wt% aqueous HBr at 0 °C. The reaction mixture was allowed to warm to rt, and it was stirred for 3–12 h. Next, the reaction mixture was diluted with Et_2O, washed (water, saturated $NaHCO_3$, and brine), dried over Na_2SO_4, and concentrated *in vacuo*. The crude organic residue was purified by flash column chromatography.

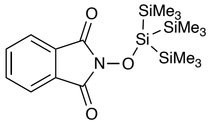

2-((1,1,1,3,3,3-hexamethyl-2-(trimethylsilyl)trisilan-2-yl)oxy)isoindoline-1,3-dione (2) was prepared from reported literature procedure by MacMillan and co-workers.[14]

Cyclic Voltammogram: An oven-dried 10 mL three-neck flask was charged with a magnetic stir bar, **2** (0.05 mmol, 20 mg), and dried-recrystallized $TBAPF_6$ (0.5 mmol, 194 mg). Acetonitrile (10 mL, Pure Process Technology, LLC solvent purification system) was added to the flask, and the resulting reaction mixture was degassed with N_2. Cyclic voltammograms were run with a Pt working electrode (SA = 0.0341 cm^2), Pt wire counter electrode, and pseudoreference Ag wire at a scan rate of 100 mV s^{-1}. iR compensation was performed. $E(Fc^+/Fc^0)$ was measured after the experiment. The second scan of a series of cyclic voltammograms are shown in Figure S1. Assuming peak-to-peak separation for a purely reversible redox couple (57 mV), the half-wave potential ($E_{1/2}$) was calculated to be –1.42 V vs. SCE, where the peak potential (E_p) of the irreversible reduction was determined to be –1.45 V vs. SCE. The internal standard, Fc^+/Fc^0 was adjusted to be +0.40 V vs. SCE in MeCN.

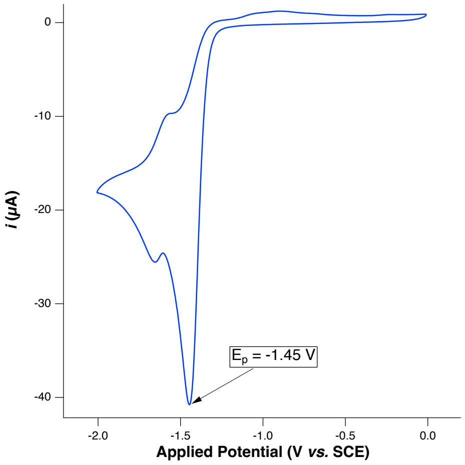

Figure S1. Cyclic voltammogram of **2**

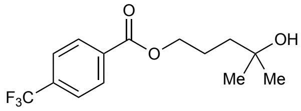

4-hydroxy-4-methylpentyl 4-(trifluoromethyl)benzoate (S1f). A mixture of 4-(trifluoromethyl)benzoic acid (950.6 mg, 5.0 mmol, 1.0 equiv), EDC·HCl (1.150 g, 6.0 mmol, 1.2 equiv), DMAP (122.2 mg, 1.0 mmol, 0.2 equiv) and 4-methylpentane-1,4-diol (886.4 mg, 7.5 mmol, 1.5 equiv) in DCM (0.2 M, 25 mL) was stirred at room temperature for 12 hours. The reaction mixture was diluted with DCM, washed with water, brine, dried over $MgSO_4$, filtered and concentrated *in vacuo*. The crude product was purified by silica gel column chromatography (0–40% EtOAc/hexane gradient) to obtain **S1f** as a clear oil (1.053 g, 73%).

Analytical data for **S1f**:
FTIR (diamond, anvil, oil) cm^{-1}: 3406, 2967, 1720, 1586, 1412, 1323, 1274, 1165, 1123, 1099, 1064, 1017, 949.
^{1}H NMR (500 MHz, $CDCl_3$) δ 8.15 (d, *J* = 8.2 Hz, 2H), 7.70 (d, *J* = 8.2 Hz, 2H), 4.38 (t, *J* = 6.7 Hz, 2H), 1.95 – 1.83 (m, 2H), 1.65 – 1.55 (m, 2H), 1.41 (s, 1H), 1.26 (s, 6H).
^{13}C NMR (126 MHz, $CDCl_3$) δ 165.5, 134.53 (q, *J* = 32.6 Hz), 133.7, 130.1, 125.53 (q, *J* = 3.8 Hz), 123.78 (q, *J* = 272.7 Hz), 70.7, 66.1, 40.0, 29.5, 23.9.
^{19}F NMR (470 MHz, $CDCl_3$) δ -63.1.
LRMS (EI^+) m/z: Mass calcd. $[C_{14}H_{17}F_3O_2]^+$ for $[M\text{-}OH]^+$ 273.1; Found 273.1

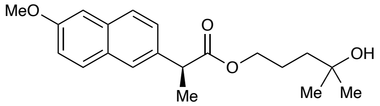

4-hydroxy-4-methylpentyl (*S*)-2-(6-methoxynaphthalen-2-yl)propanoate (S1h). A mixture of (*S*)-Naproxen (1.150 g, 5.0 mmol, 1.0 equiv), EDC·HCl (1.150 g, 6.0 mmol, 1.2 equiv), DMAP (122.2 mg, 1.0 mmol, 0.2 equiv) and 4-methylpentane-1,4-diol (886.4 mg, 7.5 mmol, 1.5 equiv) in DCM (0.2 M, 25 mL) was stirred at room temperature for 12 hours. The reaction mixture was diluted with DCM, washed with water, brine, dried over $MgSO_4$, filtered and concentrated *in vacuo*. The crude product was purified by silica gel column chromatography (0–50% EtOAc/hexane gradient) to obtain **S1h** as a waxy white solid (1.534 g, 93%).
Analytical data for **S1h**:

mp = 39.5–41.1 ºC
FTIR (diamond, anvil, solid) cm^{-1}: 3429, 3058, 2967, 2903, 2839, 1727, 1605, 1155, 1029.
^{1}H NMR (500 MHz, $CDCl_3$) δ 7.77 – 7.60 (m, 3H), 7.41 (dd, *J* = 8.5, 1.8 Hz, 1H), 7.14 (dd, *J* = 8.9, 2.5 Hz, 1H), 7.12 – 7.09 (m, 1H), 4.18 – 4.02 (m, 2H), 3.91 (s, 3H), 3.85 (q, *J* = 7.1 Hz, 1H), 1.70 – 1.61 (m, 2H), 1.58 (dd, *J* = 7.2, 0.8 Hz, 3H), 1.33 – 1.27 (m, 2H), 1.08 (s, 6H).
^{13}C NMR (126 MHz, $CDCl_3$) δ 174.8, 157.8, 136.0, 133.8, 129.4, 129.1, 127.2, 126.4, 126.1, 119.2, 105.7, 70.6, 65.1, 55.5, 45.7, 39.8, 29.2, 23.8, 18.5.
HRMS (ESI^+) *m/z*: $[M+H]^+$ Calcd. For $C_{20}H_{27}O_4^+$ 331.1904; Found 331.1910.

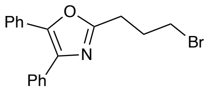

2-(3-bromopropyl)-4,5-diphenyloxazole (S2h). was prepared according to general procedure A from 3-(4,5-diphenyloxazol-2-yl)propan-1-ol (**S1a**) on a 5.37 mmol scale to afford **S2h** as a white solid (350 mg, 28%).
Analytical data for **S2h**:

mp = 47.5–48.2 ºC
FTIR (diamond, anvil, solid) cm^{-1}: 3053, 2980, 1604, 1578, 1500, 1217, 1056, 960, 777.
^{1}H NMR (500 MHz, $CDCl_3$) δ 7.64 (d, *J* = 7.5 Hz, 2H), 7.58 (d, *J* = 7.5 Hz, 2H), 7.41 – 7.30 (m, 6H), 3.59 (t, *J* = 6.5 Hz, 2H), 3.05 (t, *J* = 7.3 Hz, 2H), 2.43 (p, *J* = 6.9 Hz, 2H).
^{13}C NMR (126 MHz, $CDCl_3$) δ 162.1, 145.5, 135.3, 132.6, 129.1, 128.8, 128.7, 128.6, 128.2, 128.1, 126.6, 32.6, 30.0, 26.8.
HRMS (ESI/TOF) *m/z*: $[M+H]^+$ Calcd. For $C_{18}H_{17}BrNO^+$ 342.0488; Found 342.0482.

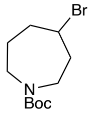

***tert*-butyl 4-bromoazepane-1-carboxylate (S2o)**. To a flame-dried round-bottom flask, *tert*-butyl 4-hydroxyazepane-1-carboxylate (2.153 g, 10.00 mmol, 1.0 equiv) was dissolved in THF (20 mL, 0.5 M) and cooled to 0 ºC. Triphenylphosphine (2.89 g, 11.00 mmol, 1.1 equiv) and CBr_4 (3.65 g, 11.00 mmol, 1.1 equiv) were added sequentially. The solution was warmed to room temperature and stirred overnight. After stirring overnight, the solution was concentrated *in vacuo* and then washed with brine and extracted with EtOAc (x3). The combined organic layer was concentrated *in vacuo* and purified by silica gel column chromatography (0–20% acetone/hexane gradient) to yield **S2o** as a pale-yellow oil (1.49 g, 54%).
Analytical data for **S2o**:

FTIR (diamond, anvil, solid) cm^{-1}: 2973, 2932, 1685, 1410, 1364, 1158, 971.
^{1}H NMR (500 MHz, $CDCl_3$) (mixture of rotamers) δ 4.45 – 4.29 (m, 1H), 3.61 – 3.26 (m, 4H), 2.33 – 2.21 (m, 1H), 2.17 – 2.03 (m, 3H), 2.00 – 1.90 (m, 1H), 1.78 – 1.66 (m, 1H), 1.48 – 1.44 (m, 9H).
^{13}C NMR (126 MHz, $CDCl_3$) δ 155.6 (A+B), 79.7 (A+B), 54.2 (A+B), 46.2 (A), 45.3 (B), 43.5 (A), 43.1 (B), 39.9 (A), 39.6 (B), 37.0 (A), 36.8 (B), 28.6 (A+B), 25.2 (A), 25.0 (B).
HRMS (ESI/TOF) *m/z*: $[M+Na]^+$ Calcd. For $C_{11}H_{20}BrNNaO_2^+$ 300.0570; Found 300.0559.

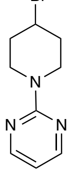

2-(4-bromopiperidin-1-yl)pyrimidine (S2t). To a flame-dried round-bottom flask, 1-(pyrimidin-2-yl)piperidin-4-ol (1.00 g, 5.58 mmol) (**S1c**) and CBr_4 (2.22 g, 6.70 mmol, 1.2 equiv) were dissolved in DCM (14 mL, 0.4 M) and cooled to 0 ºC. Triphenylphosphine (1.464 g, 5.58 mmol, 1.0 equiv) was then added. The solution was warmed to room temperature and stirred overnight. After stirring overnight, the solution was concentrated *in vacuo* and then

washed with brine and extracted with EtOAc (x3). The combined organic layer was concentrated *in vacuo* and purified by silica gel column chromatography (0–20% acetone/hexane gradient) to obtain **S2t** as a pale-yellow oil (833.1 mg, 62%).
Analytical data for **S2t**:

FTIR (diamond, anvil, oil) cm^{-1}: 3024, 2951, 2854, 1731, 1583, 1492, 1446, 1358, 1246, 1190, 978, 843.
^{1}H NMR (500 MHz, $CDCl_3$) δ 8.30 (d, *J* = 4.7 Hz, 2H), 6.48 (t, *J* = 4.7 Hz, 1H), 4.48 – 4.41 (m, 1H), 4.20 – 4.14 (m, 2H), 3.71 – 3.62 (m, 2H), 2.22 – 2.14 (m, 2H), 2.06 – 1.97 (m, 2H).
^{13}C NMR (126 MHz, $CDCl_3$) δ 161.7, 157.9, 110.0, 50.4, 42.6, 35.8.
HRMS (ESI/TOF) *m/z*: $[M+H]^+$ Calcd. For $C_9H_{13}BrN_3^+$ 242.0287; Found 242.0285.

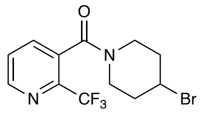

(4-bromopiperidin-1-yl)(2-(trifluoromethyl)pyridin-3-yl)methanone (S2u). To a flame-dried 100 mL round-bottom-flask equipped, with a stir bar was added 4-bromopiperidin-1-ium bromide (1.837 g, 7.5 mmol, 1.0 equiv), HOBt hydrate (1.378 g, 9.0 mmol, 1.2 equiv), and 2-(Trifluoromethyl)pyridine-3-carboxylic acid (1.433 g, 7.5 mmol, 1.0 equiv) followed by 10 mL of DCM. In a separate flask, EDC•HCl (1.725 g, 9.0 mmol, 1.2 equiv) and triethylamine (3.04 g, 4.18 mL, 30 mmol, 4 equiv) were dissolved in 32 mL of DCM. The EDC solution was then transferred to the 100 mL round-bottom-flask. The reaction was stirred overnight and quenched with 1 M HCl followed by addition of brine and extracted with DCM. The crude organic layer was dried with $MgSO_4$, filtered, and concentrated *in vacuo*. Subsequent purification by silica gel column chromatography (0–25% acetone/hexane gradient) furnished **S2u** as a white solid (1.309 g, 45% yield).
Analytical data for **S2u**:

mp = 95.3–99.5 ºC
FTIR (diamond, anvil, solid) cm^{-1}: 3053, 3006, 2959, 1745, 1628, 1442, 1325, 1194, 1123, 999.
^{1}H NMR (500 MHz, $CDCl_3$) (mixture of rotamers) δ 8.77 (d, *J* = 4.7 Hz, 1H, A+B), 7.73 (d, *J* = 7.8 Hz, 0.58H, A), 7.69 (d, *J* = 7.8 Hz, 0.42H, B), 7.56 (td, *J* = 8.0, 4.6 Hz, 1H, A+B), 4.48 (tt, *J* = 6.7, 3.6 Hz, 0.58H, A), 4.41 (tt, *J* = 7.2, 3.8 Hz, 0.42H, B), 4.00 (ddd, *J* = 13.7, 6.8, 4.0 Hz, 1H, A+B), 3.89 – 3.75 (m, 1H, A+B), 3.49 – 3.39 (m, 1H, A+B), 3.18 – 3.05 (m, 1H, A+B), 2.25 – 2.16 (m, 1H, A+B), 2.12 – 1.81 (m, 3H, A+B).
^{13}C NMR (126 MHz, $CDCl_3$) (mixture of rotamers) δ 165.40 (A), 165.36 (B), 150.0 (A+B), 143.72 (q, *J* = 34.3 Hz, A), 143.67 (q, *J* = 34.6 Hz, B), 136.0 (B), 135.9 (A), 131.00 (B), 130.95 (A), 126.64 (B), 126.58 (A), 121.44 (q, *J* = 275.5 Hz, B), 121.39 (q, *J* = 275.5 Hz, A), 48.5 (B), 47.8 (A), 45.3 (A), 44.9 (B), 39.9 (A), 39.4 (B), 35.3 (A), 34.99 (B), 34.97 (A), 34.6 (B).
^{19}F NMR (470 MHz, $CDCl_3$) δ -64.8, -65.0.
HRMS (ESI/TOF) *m/z*: $[M+H]^+$ Calcd. For $C_{12}H_{13}BrF_3N_2O^+$ 337.0158; Found: 337.0149.

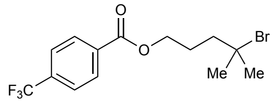

4-bromo-4-methylpentyl 4-(trifluoromethyl)benzoate (S2z) was prepared according to general procedure B on a 1.72 mmol scale to afford **S2z** as a pale yellow oil (564.5 mg, 93%)

Analytical data for **S2z:**

FTIR (diamond, anvil, oil) cm^{-1}: 2965, 2929, 1721, 1586, 1411, 1323, 1273, 1165, 1123, 1098, 1064.
^{1}H NMR (500 MHz, $CDCl_3$) δ 8.16 (d, J = 8.0 Hz, 2H), 7.71 (d, J = 8.0 Hz, 2H), 4.40 (d, J = 6.5 Hz, 2H), 2.16 – 1.99 (m, 2H), 1.97 – 1.85 (m, 2H), 1.80 (s, 6H).
^{13}C NMR (126 MHz, $CDCl_3$) δ 165.5, 134.61 (q, J = 32.7 Hz), 133.6, 130.1, 125.58 (q, J = 3.7 Hz), 122.84 (d, J = 272.9 Hz), 67.0, 65.3, 43.9, 34.4, 26.1.
^{19}F NMR (470 MHz, CDCl3) δ -63.1.
LRMS (EI^+) m/z: Mass calcd. $[C_{14}H_{16}BrF_3O_2]^+$ for $[M]^+$ 354.0; Found 354.2.

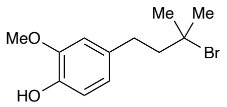

4-(3-bromo-3-methylbutyl)-2-methoxyphenol (S2aa) was prepared according to general procedure B on a 2.38 mmol scale to afford **S2aa** as a pale-yellow oil (509.1 mg, 78% yield).
Analytical data for **S2aa**:

FTIR (diamond, anvil, oil) cm^{-1}: 3505, 2964, 2932, 1607, 1513, 1265, 1099, 1033, 855.
^{1}H NMR (500 MHz, $CDCl_3$) δ 6.84 (d, J = 8.5 Hz, 1H), 6.72 – 6.68 (m, 2H), 5.47 (s, 1H), 3.89 (s, 3H), 2.92 – 2.64 (m, 2H), 2.28 – 1.92 (m, 2H), 1.82 (s, 6H).
^{13}C NMR (126 MHz, $CDCl_3$) δ 146.6, 144.0, 133.6, 121.1, 114.4, 111.1, 67.8, 56.1, 49.9, 34.4, 32.7.
LRMS (EI^+) m/z: Mass calcd. $[C_{12}H_{17}O_2]^+$ for $[M-Br]^+$ 193.1; Found 193.2.

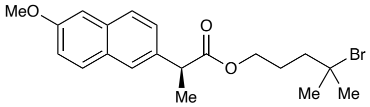

4-bromo-4-methylpentyl (*S*)-2-(6-methoxynaphthalen-2-yl)propanoate (S2ab) was prepared according to general procedure B on a 4.54 mmol scale to afford **S2ab** as a white solid (1.437 g, 80%)
Analytical data for **S2ab**:

mp = 43.0–46.5 °C
FTIR (diamond, anvil, solid) cm^{-1}: 2974, 2940, 1727, 1629, 1604, 1505, 1159, 1003, 856.
^{1}H NMR (500 MHz, $CDCl_3$) δ 7.72 – 7.66 (m, 3H), 7.41 (dd, J = 8.5, 1.9 Hz, 1H), 7.14 (dd, J = 8.9, 2.5 Hz, 1H), 7.11 (d, J = 2.5 Hz, 1H), 4.17 – 4.05 (m, 2H), 3.91 (s, 3H), 3.85 (q, J = 7.2 Hz, 1H), 1.85 – 1.76 (m, 2H), 1.64 – 1.55 (m, 11H).
^{13}C NMR (126 MHz, $CDCl_3$) δ 174.7, 157.8, 135.9, 133.9, 129.4, 129.1, 127.3, 126.4, 126.1, 119.1, 105.7, 67.2, 64.4, 55.5, 45.6, 43.7, 34.2, 34.2, 26.0, 18.4.
HRMS (ESI/TOF) *m/z*: $[M+Na]^+$ Calcd. For $C_{20}H_{25}BrNaO_3^+$ 415.0879; Found 415.0872.

Table S1. Reaction Optimization

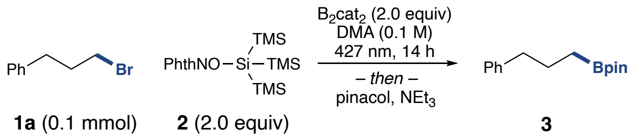

1a (0.1 mmol) **2** (2.0 equiv) **3**

entry	deviation from standard	Yield (%)[a]
1	none	**83**
2	DMF instead of DMA	24
3	MeCN instead of DMA	15
4	B_2pin_2 instead of B_2cat_2	8
5	$(TMS)_3SiH$ instead of **2**	trace
6	$(TMS)_3SiOH$ instead of **2**	trace
7	x equiv. **2** (1.0, 1.5, 3.0)	48, 59, 88
8	390 nm instead of 427 nm	73
9	456 nm instead of 427 nm	73
10	525 nm instead of 427 nm	11
	**reaction controls**	
11	no light, 65 °C	8
12	no light	5
13	no **2**	0

[a] ^{1}H NMR (500 MHz) yield of unpurified mixture with 1,3,5-trimethoxybenzene as an internal standard.

In an N_2 inert atmosphere glovebox, **1a** (19.9 mg, 0.10 mmol), **2** (82.0 mg, 0.20 mmol), and B_2cat_2 (47.6 mg, 0.20 mmol) were added sequentially to a flame-dried 2-dram vial equipped with a magnetic stir bar. The mixture was suspended in DMA (1.0 mL, 0.1 M) and sealed with a cap. The vial was removed from the glovebox and irradiated with stirring using two Kessil PhotoReaction PR160L 427 nm LEDs at 100% intensity with fan cooling. After 14 hours of irradiation, the vial was removed and pinacol (70.9 mg, 0.6 mmol) and triethylamine (0.1 mL) were added to the solution. After 1 hour of stirring, saturated sodium bicarbonate (~1 mL) and H_2O (~1 mL), and the organic layer was extracted with ethyl acetate until the organic layer was colorless (~3 mL EtOAc x 4). The combined organic layers were dried with Na_2SO_4 and concentrated for NMR analysis. The yields were determined by ^{1}H NMR spectroscopy with 1,3,5-trimethylbenzene (0.02 mmol) as an internal standard.

General procedure C: Photoinduced Borylation

In an N_2 inert atmosphere glovebox, the respective bromide (0.20 mmol), **2** (164 mg, 0.40 mmol), and B_2cat_2 (2.0 equiv, 95 mg, 0.40 mmol) were added sequentially to a flame-dried 2-dram vial equipped with a magnetic stir bar. The mixture was suspended in DMA (2.0 mL, 0.1 M) and sealed with a cap. The vial was removed from the glovebox and irradiated with stirring using two Kessil PhotoReaction PR160L 427 nm LEDs at 100% intensity with fan cooling for 14 h. The conversion of starting material was typically monitored by GC-MS. The reaction was removed from irradiation and subjected to workup conditions described below.

Pictures of the Photochemical Set-up

A standard XAT radical borylation set-up is shown below. All reactions were performed in 2-dram vials located at the center of two Kessil PhotoReaction PR160L 427 nm LEDs at 100% intensity. Vials were taped to the stir plate during irradiation.

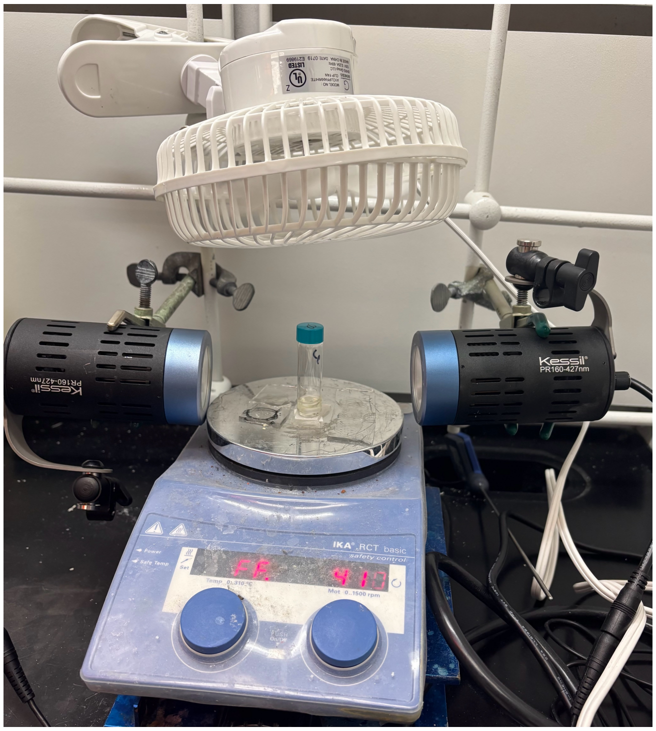

Figure S2. Standard photochemical set-up.

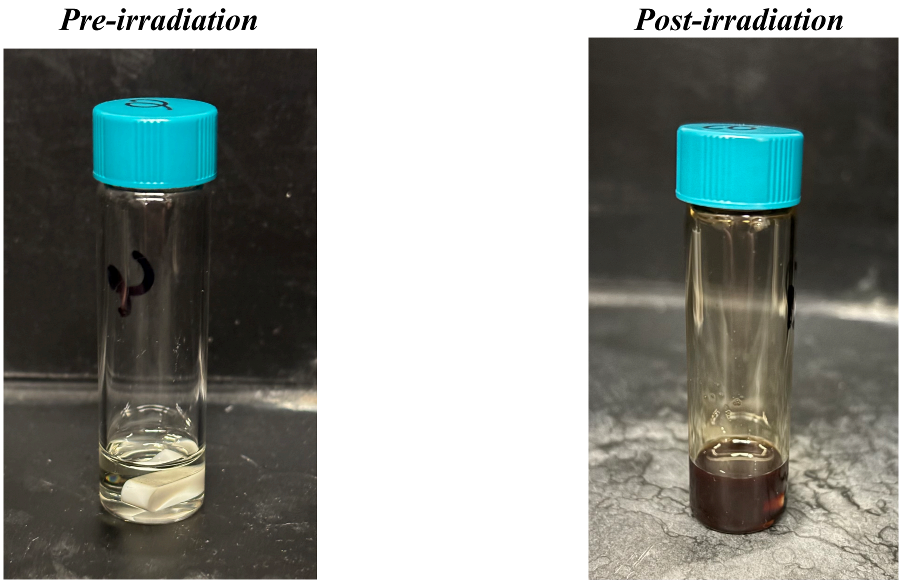

Figure S3. Reaction color change from pre- and post-irradiation.

Workup A

Triethylamine (200 μL) and 2,3-dimethylbutane-2,3-diol (142 mg, 6 equiv) *or* 3,4-diethylhexane-3,4-diol (209 mg, 6 equiv) was charged into the reaction mixture, and the reaction was stirred for 1 h at room temperature. Then, 10 mL of sat. sodium bicarbonate solution and 10 mL of EtOAc were added to the reaction mixture, and the organic layer was extracted. Next, the aqueous layer was re-extracted with 10 mL of EtOAc (x3). The organic layers were combined and washed with deionized water (10 mL x 3), then 10 mL of brine. The organic layer was then dried with Na_2SO_4, filtered, and solvent removed *in vacuo,* and the crude residue was purified by silica gel column chromatography.

Workup B

Triethylamine (200 μL) and 2,3-dimethylbutane-2,3-diol (142 mg, 6 equiv) *or* 3,4-diethylhexane-3,4-diol (209 mg, 6 equiv) were charged into the reaction vial, and the reaction was stirred for 1 h at room temperature. Then, 10 mL of saturated sodium bicarbonate solution and 10 mL of EtOAc were added to the reaction mixture, and the organic layer was extracted. Next, the aqueous layer was re-extracted with 10 mL of EtOAc (3x). The organic layer was combined and washed with deionized water (10 mL x 3), then 10 mL of brine. The organic layer was then dried with Na_2SO_4, filtered and solvent removed *in vacuo.* The crude reaction mixture was resuspended in 5 mL of THF, and the solution was cooled to 0 ºC under stirring. Then, 1M TBAF solution (600 μL, 3 equiv) was added dropwise, and the mixture was allowed to stir for 30 min. Upon addition of TBAF the reaction was observed to turn dark red. **Caution:** TBAF in the presence of water results in efficient protodeboronation.[15] The reaction was concentrated *in vacuo*, resuspended in 5 mL DCM, and washed with 5 mL deionized water and 5 mL brine. The aqueous layer was re-extracted with DCM until colorless. The combined organic layer was dried with Na_2SO_4, filtered, solvent removed *in vacuo*, and the crude residue was purified by silica gel column chromatography.

Note: EtOAc can be used instead of DCM. Additional brine was added to aid separation if needed.

Workup C

2,2'-(methylazanediyl)diacetic acid (176.6 mg, 6 equiv) was added to the reaction vial. The vial was capped and heated to 90 ºC under stirring for 3 hours. After cooling to room temperature, 10 mL of sat. sodium bicarbonate solution and 10 mL of EtOAc were added to the reaction mixture, and the organic layer was extracted. Next, the aqueous layer was re-extracted with 10 mL of EtOAc (3x). The organic layer was combined and washed with deionized water (10 mL x 3), then 10 mL of brine. The organic layer was then dried with Na_2SO_4, filtered and solvent removed *in vacuo,* and the crude residue was purified by silica gel column chromatography (0–60% acetone/hexane gradient). The MIDA product was then resuspended in minimal amounts of acetone and recrystallized upon addition of diethyl ether.

Note: We observed that the conversion of the reaction is highly dependent on the purity of B_2cat_2. B_2cat_2 from AK Scientific and AA Blocks LLC was used for this study.

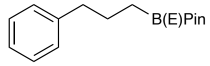

4,4,5,5-tetraethyl-2-(3-phenylpropyl)-1,3,2-dioxaborolane (3a). Prepared according to General procedure C using (3-bromopropyl)benzene **(S2a)**. Work-up B was performed with 5 equiv of TBAF, and the reaction mixture was purified by silica gel chromatography (0–4% Et_2O/hexane gradient) to obtain **3a** as a clear oil (47.0 mg, 78%)
Analytical data for **3a**:

FTIR (diamond, anvil, oil) cm^{-1}: 2974, 2936, 2883, 1604, 1496, 1410, 1374, 1305, 1114, 929.
^{1}H NMR (500 MHz, $CDCl_3$) δ 7.28 – 7.23 (m, 2H), 7.20 – 7.13 (m, 3H), 2.61 (t, *J* = 7.9 Hz, 2H), 1.78 – 1.61 (m, 10H), 0.95 – 0.88 (m, 12H), 0.84 (t, *J* = 7.9 Hz, 2H).
^{13}C NMR (126 MHz, $CDCl_3$) δ 143.0, 128.7, 128.3, 125.7, 88.1, 38.8, 26.5, 9.0. The α-B carbon signal was not observed.
^{11}B NMR (160 MHz, $CDCl_3$) δ 33.8.
HRMS (ESI/TOF) *m/z*: $[M+H]^+$ Calcd. For $C_{19}H_{32}BO_2^+$ 303.2490; Found 303.2483.

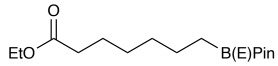

ethyl 7-(4,4,5,5-tetraethyl-1,3,2-dioxaborolan-2-yl)heptanoate (3b). Prepared according to General procedure C using ethyl 7-bromoheptanoate **(S2b)**. Work-up B was performed with 5 equiv of TBAF, and the reaction mixture was purified by silica gel chromatography (0–4% Et_2O/hexane gradient) to obtain **3b** as a pale-yellow oil (52.1 mg, 77%).
Analytical data for **3b**:

FTIR (diamond, anvil, oil) cm^{-1}: 2977, 2932, 2859, 1736, 1459, 1374, 1279, 1181, 1114, 921.
^{1}H NMR (500 MHz, $CDCl_3$) δ 4.10 (q, *J* = 7.1 Hz, 2H), 2.26 (t, *J* = 7.6 Hz, 2H), 1.71 – 1.54 (m, 10H), 1.39 (qd, *J* = 7.3, 3.9 Hz, 2H), 1.31 – 1.28 (m, 4H), 1.23 (t, *J* = 7.1 Hz, 3H), 0.89 (t, *J* = 7.5 Hz, 12H), 0.75 (t, *J* = 7.7 Hz, 2H).
^{13}C NMR (126 MHz, $CDCl_3$) δ 174.0, 88.0, 60.2, 34.5, 32.1, 29.1, 26.5, 25.1, 24.1, 14.4, 11.1, 8.9.
^{11}B NMR (160 MHz, $CDCl_3$) δ 34.0.
HRMS (ESI/TOF) *m/z*: $[M+Na]^+$ Calcd. For $C_{19}H_{37}BNaO_4^+$ 363.2677; Found 363.2671.

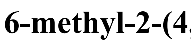

6-methyl-2-(4,4,4-trifluorobutyl)-1,3,6,2-dioxazaborocane-4,8-dione (3c). Prepared according to General procedure C from 4-bromo-1,1,1-trifluorobutane **(S2c)**. Workup C was performed, and the reaction mixture was purified by silica gel chromatography (0–60% acetone/hexane gradient) followed by recrystallization to afford **3c** an off-white solid (35.6 mg, 67%).
Analytical data for **3c**:

mp: 162.5–163.3 ºC
FTIR (diamond, anvil, solid) cm^{-1}: 3028, 2955, 1768, 1736, 1427, 1307, 1258, 1076.
^{1}H NMR (500 MHz, CD_3CN) δ 3.94 (d, *J* = 17.0 Hz, 2H), 3.79 (d, *J* = 17.0 Hz, 2H), 2.84 (s, 3H), 2.29 – 2.15 (m, 2H), 1.72 – 1.53 (m, 2H), 0.67 (t, *J* = 8.7 Hz, 2H).

^{13}**C NMR** (126 MHz, CD_3CN) δ 169.1, 132.0, 128.76 (q, *J* = 275.9 Hz), 62.8, 46.62, 36.5 (q, *J* = 27.3 Hz), 17.75 (q, *J* = 3.2 Hz). The α-B carbon signal was not observed.
^{19}**F NM**R (470 MHz, CD_3CN) δ -66.9.
^{11}**B NMR** (160 MHz, CD_3CN) δ 12.8.
HRMS (ESI/TOF) *m/z*: $[M+H]^+$ Calcd. For $C_9H_{14}BF_3NO_4^+$ 268.0962; Found 268.0965.

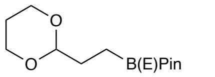

2-(2-(1,3-dioxan-2-yl)ethyl)-4,4,5,5-tetraethyl-1,3,2-dioxaborolane (3d). Prepared according to a modified General procedure C in which B_2cat_2 (142.6 mg, 0.60 mmol, 3 equiv) was used with 2-(2-bromoethyl)-1,3-dioxane **(S2d).** Work-up B was performed, and the reaction mixture was purified by silica gel chromatography (0–6% EtOAc/hexane gradient) to yield **3d** as a yellow oil (48.9 mg, 82%).
Analytical data for **3d**:

FTIR (diamond, anvil, oil) cm^{-1}: 2969, 2884, 2846, 1459, 1370, 1349, 1286, 1145, 1003, 930.
^{1}**H NMR** (500 MHz, $CDCl_3$) δ 4.46 (t, *J* = 5.3 Hz, 1H), 4.09 – 4.03 (m, 2H), 3.71 (td, *J* = 12.2, 2.3 Hz, 2H), 2.10 – 1.98 (m, 1H), 1.72 – 1.55 (m, 10H), 1.29 (d, *J* = 13.5 Hz, 1H), 0.88 (t, *J* = 7.5 Hz, 12H), 0.81 (t, *J* = 8.0 Hz, 2H).
^{13}**C NMR** (126 MHz, $CDCl_3$) δ 103.5, 88.1, 66.9, 29.8, 26.4, 26.0, 8.9, 5.2.
^{11}**B NMR** (160 MHz, $CDCl_3$) δ 33.5.
HRMS (ESI/TOF) *m/z*: $[M+Na]^+$ Calcd. For $C_{16}H_{31}BNaO_4^+$ 321.2208; Found 321.2199.

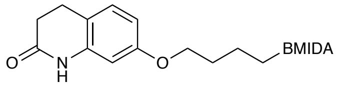

6-methyl-2-(4-((2-oxo-1,2,3,4-tetrahydroquinolin-7-yl)oxy)butyl)-1,3,6,2-dioxazaborocane-4,8-dione (3e). Prepared according to General procedure C with 7-(4-bromobutoxy)-3,4-dihydroquinolin-2(1*H*)-one **(S2e)**. Work-up C was performed, and the reaction mixture was purified by silica gel chromatography (0–60% acetone/hexane gradient) followed by recrystallization to yield **3e** as an off-white solid (44.0 mg, 59%).
Analytical data for **3e**:

mp = 137.2–139.5 ºC
FTIR (diamond, anvil, solid) cm^{-1}: 3003, 2967, 2939, 1749, 1692, 1594, 1492, 1273, 1193, 981.
^{1}**H NMR** (500 MHz, DMSO) δ 9.95 (s, 1H), 7.03 (d, *J* = 8.2 Hz, 1H), 6.47 (dd, *J* = 8.2, 2.5 Hz, 1H), 6.43 (d, *J* = 2.5 Hz, 1H), 4.17 (d, *J* = 17.1 Hz, 2H), 3.97 (d, *J* = 17.1 Hz, 2H), 3.89 (t, *J* = 6.4 Hz, 2H), 2.84 (s, 3H), 2.77 (t, *J* = 7.6 Hz, 2H), 2.41 (dd, *J* = 8.4, 6.5 Hz, 2H), 1.75 – 1.67 (m, 2H), 1.43 – 1.35 (m, 2H), 0.57 (dd, *J* = 10.2, 6.3 Hz, 2H).
^{13}**C NMR** (126 MHz, DMSO) δ 170.3, 169.0, 158.0, 139.2, 128.4, 115.4, 107.6, 101.7, 67.3, 61.6, 45.5, 31.7, 30.8, 24.0, 20.3, 15.3.
^{11}**B NMR** (160 MHz, $CDCl_3$) δ 16.4.
HRMS (ESI/TOF) *m/z*: $[M+H]^+$ Calcd. For $C_{18}H_{24}BN_2O_6^+$ 375.1722; Found 375.1720.

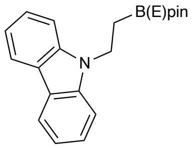

9-(2-(4,4,5,5-tetraethyl-1,3,2-dioxaborolan-2-yl)ethyl)-9*H*-carbazole (3f). prepared according to General procedure C using 9-(2-bromoethyl)-9*H*-carbazole (**S2f**). Workup B was performed, and the reaction mixture was purified by silica gel chromatography (0–3% EtOAc/hexane gradient) to obtain **3f** as a white solid (50.8 mg, 67%).
Analytical data for **3f**:

mp = 79.5–80.7 ºC.
FTIR (diamond, anvil, solid) cm^{-1}: 3053, 2971, 2941, 1628, 1465, 1406, 1352, 1128, 841.
^{1}H NMR (500 MHz, $CDCl_3$) δ 8.09 (d, J = 7.7 Hz, 2H), 7.52 – 7.43 (m, 4H), 7.21 (ddd, J = 7.9, 6.2, 1.8 Hz, 2H), 4.46 (t, J = 7.9 Hz, 2H), 1.61 (q, J = 7.5 Hz, 8H), 1.44 (t, J = 8.1 Hz, 2H), 0.87 (t, J = 7.5 Hz, 12H).
^{13}C NMR (126 MHz, $CDCl_3$) δ 140.1, 125.5, 123.0, 120.3, 118.7, 109.1, 88.7, 39.0, 26.5, 8.9. The α-B carbon signal was not observed.
^{11}B NMR (160 MHz, $CDCl_3$) δ 32.3.
HRMS (ESI/TOF) *m/z*: $[M+H]^+$ Calcd. For $C_{24}H_{33}BNO_2^+$ 378.2599; Found 378.2588

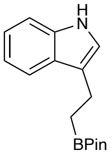

3-(2-(4,4,5,5-tetramethyl-1,3,2-dioxaborolan-2-yl)ethyl)-1*H*-indole (3g). prepared according to General procedure C using 3-(2-bromoethyl)-1H-indole (**S2g**). Work-up A was performed, and the reaction mixture was purified by silica gel chromatography (0–10% EtOAc/hexanes gradient) to yield **3g** as a colorless oil (23.6 mg, 44%).
Analytical data for **3g**:

^{1}H NMR (500 MHz, $CDCl_3$) δ 7.88 (s, 1H), 7.65 (d, J = 7.9 Hz, 1H), 7.33 (d, J = 8.0 Hz, 1H), 7.21 – 7.14 (m, 1H), 7.10 (t, J = 7.4 Hz, 1H), 7.01 – 6.97 (m, 1H), 2.90 (dd, J = 9.0, 7.1 Hz, 2H), 1.26 (t, J = 8.3 Hz, 3H), 1.23 (s, 12H).
^{13}C NMR (126 MHz, $CDCl_3$) δ 136.5, 127.6, 121.9, 120.8, 119.3, 119.3, 119.1, 111.0, 83.2, 25.0, 19.5. The α-B carbon signal was not observed.
Product matched literature characterization.[16]

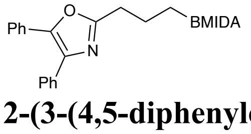

2-(3-(4,5-diphenyloxazol-2-yl)propyl)-6-methyl-1,3,6,2-dioxazaborocane-4,8-dione (3h). Prepared according to General procedure C from 2-(3-bromopropyl)-4,5-diphenyloxazole **(S2h)**. Work-up C was performed, and the reaction mixture was purified by silica gel chromatography (0–60% acetone/hexane gradient) followed by recrystallization to afford **3h** as an off-white solid (64.9 mg, 78%).
Analytical data for **3h**:

mp = 162.4–163.9 ºC

FTIR (diamond, anvil, solid) cm^{-1}: 3022, 2886, 1744, 1607, 1569, 1340, 1305, 1086, 1043, 762.
^{1}**H NMR** (500 MHz, CD_3CN) δ 7.63 – 7.59 (m, 2H), 7.58 – 7.55 (m, 2H), 7.43 – 7.32 (m, 6H), 3.94 (d, *J* = 17.0 Hz, 2H), 3.79 (d, *J* = 17.0 Hz, 2H), 2.90 – 2.84 (m, 5H), 1.91 – 1.82 (m, 2H), 0.77 – 0.71 (m, 2H).
^{13}**C NMR** (126 MHz, CD_3CN) δ 169.1, 164.8, 145.9, 135.7, 133.8, 130.2, 129.7, 129.5, 128.9, 128.6, 127.5, 62.8, 46.6, 31.3, 22.8. The α-B carbon signal was not observed.
^{11}**B NMR** (160 MHz, CD_3CN) δ 13.0.
HRMS (ESI/TOF) *m/z*: $[M+H]^+$ Calcd. For $C_{23}H_{24}BN_2O_5^+$ 419.1773; Found 419.1772.

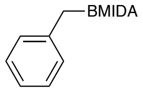

2-benzyl-6-methyl-1,3,6,2-dioxazaborocane-4,8-dione (3i). Prepared according to a modified General procedure C in which B_2cat_2 (142.6 mg, 0.60 mmol, 3 equiv) and (bromomethyl)benzene (**S2i**) were used. Work-up C was performed, and the reaction mixture was purified by silica gel chromatography (0–60% acetone/hexane gradient) followed by recrystallization to afford **3i** as a white solid (30.7 mg, 62%).
Analytical data for **3i**:

^{1}**H NMR** (500 MHz, CD_3CN) δ 7.31 – 7.16 (m, 4H), 7.14 – 7.05 (m, 1H), 3.92 (d, *J* = 16.9 Hz, 2H), 3.70 (d, *J* = 16.7 Hz, 2H), 2.89 (s, 3H), 2.18 (s, 2H).
^{13}**C NMR** (126 MHz, CD_3CN) δ 168.9, 141.4, 130.1, 129.0, 125.5, 62.9, 46.6. The α-B carbon signal was not observed.
Product matched literature characterization.[17]

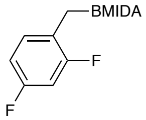

2-(2,4-difluorobenzyl)-6-methyl-1,3,6,2-dioxazaborocane-4,8-dione (3j). Prepared according to a modified General procedure C in which B_2cat_2 (142.6 mg, 0.60 mmol, 3 equiv) and 1-(bromomethyl)-2,4-difluorobenzene (**S2j**) were used. Work-up C was performed, and the reaction mixture was purified by silica gel chromatography (0–60% acetone/hexane gradient) followed by recrystallization to yield **3j** as a light brown solid (31.0 mg, 53%).
Analytical data for **3j**:

mp = decomposition at 253.9–254.6 ºC
FTIR (diamond, anvil, solid) cm^{-1}: 3008, 2887, 1772, 1743, 1615, 1603, 1502, 1342, 1305, 1103, 963.
^{1}**H NMR** (500 MHz, DMSO) δ 7.22 (q, *J* = 8.1 Hz, 1H), 7.08 (td, *J* = 9.6, 2.6 Hz, 1H), 6.96 (d, *J* = 8.6 Hz, 1H), 4.23 (d, *J* = 17.1 Hz, 2H), 4.05 (d, *J* = 17.0 Hz, 2H), 2.93 (s, 3H), 2.01 (s, 2H).
^{13}**C NMR** (126 MHz, DMSO) δ 168.7, 160.8 (dd, *J* = 27.4, 11.8 Hz), 158.9 (dd, *J* = 24.7, 12.0 Hz) 131.9 (dd, *J* = 9.3, 6.6 Hz), 123.4 (dd, *J* = 17.0, 3.6 Hz), 110.7 (dd, *J* = 20.7, 3.5 Hz), 103.1 (apparent t, *J* = 25.2 Hz), 61.7, 45.6, 15.7 (br s).
^{19}**F NMR** (470 MHz, DMSO) δ -112.7, -116.0.
^{11}**B NMR** (160 MHz, DMSO) δ 12.2.
HRMS (ESI/TOF) *m/z*: $[M+NH_4]^+$ Calcd. For $C_{12}H_{16}BF_2N_2O_4^+$ 301.1165; Found 301.1156.

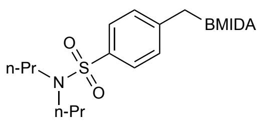

4-((6-methyl-4,8-dioxo-1,3,6,2-dioxazaborocan-2-yl)methyl)-N,N-dipropylbenzenesulfonamide (3k). Prepared according to General procedure C from 4-(bromomethyl)-N,N-dipropylbenzenesulfonamide (**S2k**). Work-up C was performed, and the reaction mixture was purified by silica gel chromatography (0–60% acetone/hexane gradient) followed by recrystallization to afford **3k** as an off-white solid (58 mg, 71%).
Analytical data for **3k**:

mp = 175.4–176.6 ºC
FTIR (diamond, anvil, solid) cm^{-1}: 2966, 2876, 1771, 1751, 1596, 1334, 1293, 1152, 1013, 885.
^{1}H NMR (500 MHz, CD_3CN) δ 7.66 (d, J = 8.0 Hz, 2H), 7.35 (d, J = 8.0 Hz, 2H), 3.96 (d, J = 17.1 Hz, 2H), 3.77 (d, J = 17.0 Hz, 2H), 3.03 (dd, J = 8.7, 6.5 Hz, 4H), 2.90 (s, 3H), 2.22 (s, 2H), 1.63 – 1.40 (m, 4H), 0.85 (t, J = 7.4 Hz, 6H).
^{13}C NMR (126 MHz, CD3CN) δ 168.8, 147.4, 137.2, 130.6, 127.8, 63.0, 51.0, 46.9, 22.9, 11.4.
^{11}B NMR (160 MHz, CD_3CN) δ 12.2, 8.6.
HRMS (ESI/TOF) *m/z*: $[M+H]^+$ Calcd. For $C_{18}H_{28}BN_2O_6S^+$ 411.1756; Found 411.1753.

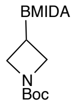

***tert*-butyl 3-(6-methyl-4,8-dioxo-1,3,6,2-dioxazaborocan-2-yl)azetidine-1-carboxylate** (**3l**). Prepared according to General procedure C from *tert*-butyl 3-bromoazetidine-1-carboxylate (**S2l**). Work-up C was performed, and the reaction mixture was purified by silica gel chromatography (0–60% acetone/hexane gradient) followed by recrystallization to afford **3l** as an off-white solid (36.2 mg, 58%).
Analytical data for **3l**:

mp = 160.2-161.5 ºC
FTIR (diamond, anvil, solid) cm^{-1}: 2973, 2919, 1757, 1680, 1410, 1304, 1248, 1131.
^{1}H NMR (500 MHz, CD_3CN) δ 4.01 – 3.96 (m, 2H), 3.96 – 3.88 (br s, 2H), 3.85 – 3.80 (m, 2H), 3.71 (br s, 2H), 2.77 – 2.74 (m, 3H), 2.09 – 2.01 (m, 1H), 1.42 – 1.39 (m, 9H).
^{13}C NMR (126 MHz, CD_3CN) δ 169.0, 157.1, 79.4, 63.3, 51.8, 50.5, 46.7, 28.6. The α-B carbon signal was not observed.
^{11}B NMR (160 MHz, CD_3CN) δ 12.5.
HRMS (ESI/TOF) *m/z*: $[M+Na]^+$ Calcd. For $C_{13}H_{21}BN_2NaO_6^+$ 335.1385; Found 335.1384.

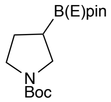

***tert*-butyl 3-(4,4,5,5-tetraethyl-1,3,2-dioxaborolan-2-yl)pyrrolidine-1-carboxylate (3m)**. prepared according to General procedure C using *tert*-butyl 3-bromopyrrolidine-1-carboxylate (**S2m).** Work-up A was performed, and the reaction mixture was purified by silica gel chromatography (0–3% EtOAc/hexane gradient) to obtain **3m** as a clear oil (41.3 mg, 58%).
Analytical data for **3m**:

FTIR (diamond, anvil, oil) cm^{-1}: 2974, 2883, 1695, 1456, 1388, 1164, 1110, 880.

^{1}H NMR (500 MHz, $CDCl_3$) δ 3.57 – 3.35 (m, 2H), 3.20 (s, 2H), 2.03 – 1.91 (m, 1H), 1.78 (m, 1H), 1.71 – 1.52 (m, 9H), 1.45 (s, 9H), 0.89 (t, *J* = 7.4 Hz, 12H).
^{13}C NMR (126 MHz, $CDCl_3$) (mixture of rotamers) δ 154.7, 88.6, 78.9, 48.3, 46.8, 28.7, 28.0, 26.5, 8.9. The α-B carbon signal was not observed.
^{11}B NMR (160 MHz, CDCl3) δ 32.6.
HRMS (ESI/TOF) *m/z*: $[M+Na]^+$ Calcd. For $C_{19}H_{36}BNNaO_4^+$ 376.2629; Found 376.2628.

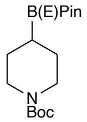

***tert*-butyl 4-(4,4,5,5-tetraethyl-1,3,2-dioxaborolan-2-yl)piperidine-1-carboxylate (3n)**. Prepared according to the General procedure C using *tert*-butyl 4-bromopiperidine-1-carboxylate (**S2n**). Work-up B was performed with 5 equiv of TBAF, and the reaction mixture was purified by silica gel chromatography (0–14% Et_2O/hexane gradient) to yield **3n** as a pale-yellow oil (53.5 mg, 73%).
Analytical data for **3n**:

FTIR (diamond, anvil, oil) cm^{-1}: 2975, 2933, 2852, 1692, 1417, 1388, 1163, 1105, 921.
^{1}H NMR (500 MHz, $CDCl_3$) δ 3.87 – 3.74 (m, 2H), 2.88 (ddd, *J* = 13.7, 10.3, 3.1 Hz, 2H), 1.69 – 1.55 (m, 10H), 1.51 – 1.45 (m, 2H), 1.44 (s, 9H), 1.13 – 1.06 (m, 1H), 0.88 (t, *J* = 7.5 Hz, 12H).
^{13}C NMR (126 MHz, $CDCl_3$) δ 155.0, 88.2, 79.1, 45.2, 28.6, 27.3, 26.5, 19.9, 8.9.
^{11}B NMR (160 MHz, $CDCl_3$) δ 33.0.
HRMS (ESI/TOF) *m/z*: $[M+H]^+$ Calcd. For $C_{20}H_{39}BNO_4^+$ 368.2967; Found 368.2965.

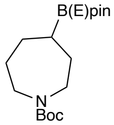

***tert*-butyl 3-(4,4,5,5-tetraethyl-1,3,2-dioxaborolan-2-yl)azepane-1-carboxylate (3o)**. Prepared according to the General procedure C using *tert*-butyl 4-bromoazepane-1-carboxylate **(S2o)** . Work-up B was performed, and the reaction mixture was purified by silica gel chromatography (0–4% EtOAc/hexane gradient) to yield **3o** as a yellow oil (66.6 mg, 87%).
Analytical data for **3o**:

FTIR (diamond, anvil, oil) cm^{-1}: 2974, 2930, 1691, 1458, 1412, 1239,1165, 1113, 922.
^{1}H NMR (500 MHz, $CDCl_3$) (mixture of rotamers) δ 3.52 – 3.37 (m, 2H), 3.33 – 3.13 (m, 2H), 1.92 – 1.72 (m, 3H), 1.69 – 1.50 (m, 10H), 1.42 (s, 9H), 1.40 – 1.32 (m, 1H), 1.06 – 0.99 (m, 1H), 0.89 – 0.83 (m, 12H).
^{13}C NMR (126 MHz, $CDCl_3$) (mixture of rotamers) δ 155.9, 155.8, 88.2, 88.2, 78.9, 78.9, 47.7, 47.3, 47.0, 46.4, 30.5, 30.5, 29.2, 29.2, 28.71, 28.68, 26.49, 26.45, 26.42, 8.93, 8.90, 8.88, 8.79.
^{11}B NMR (160 MHz, $CDCl_3$) δ 33.2.
HRMS (ESI/TOF) *m/z*: $[M+Na]^+$ Calcd. For $C_{21}H_{40}BNNaO_4^+$ 404.2942; Found 404.2941.

1.00 mmol scale: Prepared according to a modified General procedure C using *tert*-butyl 4-bromoazepane-1-carboxylate (**S2o**) (278.2 mg, 1.00 mmol, 1 equiv), B_2cat_2 (713.4 mg, 3.00 mmol, 3 equiv) **2** (819.6 mg, 2.00 mmol, 2 equiv) and DMA (5.0 mL, 0.2 M). After 24 h, work-

up B was performed with 0.2 mL TEA and 3,4-diethylhexane-3,4-diol (1.05 g, 6.00 mmol 6 equiv) followed by 1 M TBAF workup (5.00 mL, 5 equiv), and the reaction mixture was purified by silica gel chromatography (0–8% EtOAc: hexane gradient followed by a second silica gel column (0–12% Et_2O/hexane gradient) to yield **3o** as a yellow oil (220.3 mg, 58%).

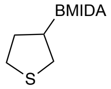

6-methyl-2-(tetrahydrothiophen-3-yl)-1,3,6,2-dioxazaborocane-4,8-dione (3p). Prepared according to General procedure C from 3-bromotetrahydrothiophene (**S2p**). Work-up C was performed, and the reaction mixture was purified by silica gel chromatography (0–60% acetone/hexane gradient) followed by recrystallization to afford **3p** an off-white solid (31.5 mg, 65%).
Analytical data for **3p**:

mp = 184.0–185.9 ºC
FTIR (diamond, anvil, solid) cm^{-1}: 2959, 2923, 2852, 1701, 1613, 1514, 1397, 1364, 1245, 1057, 843.
^{1}**H NMR** (500 MHz, DMSO) δ 4.24 (dd, *J* = 17.1, 11.2 Hz, 2H), 4.02 (t, *J* = 17.3 Hz, 2H), 2.93 (s, 3H), 2.91 – 2.83 (m, 2H), 2.74 – 2.61 (m, 1H), 2.55 – 2.45 (m, 1H), 2.34 – 2.20 (m, 1H), 1.47 – 1.33 (m, 1H), 1.31 – 1.21 (m, 1H).
^{13}**C NMR** (126 MHz, DMSO) δ 168.9, 168.8, 61.8, 61.8, 45.9, 33.8, 33.7, 32.7. The α-B carbon signal was not observed.
^{11}**B NMR** (160 MHz, DMSO) δ 12.2.
HRMS (ESI/TOF) *m/z*: $[M+Na]^+$ Calcd. For $C_9H_{14}BNNaO_4S^+$ 266.0629; Found 266.0637.

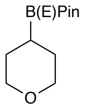

4,4,5,5-tetraethyl-2-(tetrahydro-2H-pyran-4-yl)-1,3,2-dioxaborolane (3q). Prepared according to the General procedure C using 4-bromotetrahydro-2*H*-pyran **(S2q)**. Work-up B was performed with 5 equiv of TBAF, and the reaction mixture was purified by silica gel chromatography (0–6% Et_2O/hexane gradient) to yield **3q** as a yellow oil (28.6 mg, 53%).
Analytical data for **3q**:

FTIR (diamond, anvil, oil) cm^{-1}: 2970, 2929, 2836, 1460, 1378, 1339, 1292, 1162, 1116, 895.
^{1}**H NMR** (500 MHz, $CDCl_3$) δ 3.86 – 3.80 (m, 2H), 3.49 – 3.41 (m, 2H), 1.72 – 1.57 (m, 11H), 1.24 – 1.17 (m, 2H), 0.90 (t, *J* = 7.5 Hz, 12H).
^{13}**C NMR** (126 MHz, $CDCl_3$) δ 88.2, 69.0, 28.0, 26.5, 8.9. The α-B carbon signal was not observed.
^{11}**B NMR** (160 MHz, $CDCl_3$) δ 33.5.
HRMS (ESI/TOF) *m/z*: $[M+H]^+$ Calcd. For $C_{15}H_{30}BO_3^+$ 269.2283; Found 269.2282

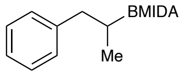

6-methyl-2-(1-phenylpropan-2-yl)-1,3,6,2-dioxazaborocane-4,8-dione (3r). Prepared according to the General procedure C using (2-bromopropyl)benzene **(S2r)**. Work-up C was performed, and the reaction mixture was purified by silica gel chromatography (0–60%

acetone/hexane gradient) followed by recrystallization to yield **3r** as a light brown solid (27.5 mg, 50%).
Analytical data for **3r**:

mp = 168.4-170.8 ºC
FTIR (diamond, anvil, solid) cm^{-1}: 3014, 2951, 2868, 1735, 1673, 1452, 1304, 1025, 971.
^{1}H NMR (500 MHz, DMSO) δ 7.29 – 7.23 (m, 2H), 7.20 – 7.13 (m, 3H), 4.24 (t, *J* = 17.5 Hz, 2H), 4.05 (t, *J* = 17.8 Hz, 2H), 2.98 (s, 3H), 2.88 (dd, *J* = 13.6, 2.9 Hz, 1H), 2.14 – 2.05 (m, 1H), 1.16 – 1.07 (m, 1H), 0.66 (d, *J* = 7.0 Hz, 3H).
^{13}C NMR (126 MHz, DMSO) δ 169.1, 168.9, 142.4, 128.8, 128.0, 125.4, 62.4, 62.3, 45.5, 37.7, 21.1, 13.7.
^{11}B NMR (160 MHz, DMSO) δ 12.9.
HRMS (ESI/TOF) *m/z*: [M+Na]$^+$ Calcd. For $C_{14}H_{18}BNNaO_4^+$ 298.1221; Found 298.1217.

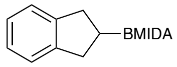

2-(2,3-dihydro-1*H*-inden-2-yl)-6-methyl-1,3,6,2-dioxazaborocane-4,8-dione (3s). Prepared according to General procedure C using 2-bromo-2,3-dihydro-1*H*-indene **(S2s)**. Work-up C was performed, and the reaction mixture was purified by silica gel chromatography (0–60% acetone/hexane gradient) followed by recrystallization to yield **3s** as a tan solid (40.3 mg, 74%).
Analytical data for **3s**:

mp = decomposition at 265.2-269.0 ºC
FTIR (diamond, anvil, solid) cm^{-1}: 2962, 2906, 1724, 1604, 1452, 1332, 1106, 1028, 855.
^{1}H NMR (500 MHz, DMSO) δ 7.18 (dd, *J* = 5.4, 3.3 Hz, 2H), 7.08 (dd, *J* = 5.5, 3.2 Hz, 2H), 4.25 (d, *J* = 17.1 Hz, 2H), 4.04 (d, *J* = 17.1 Hz, 2H), 2.95 (s, 3H), 2.95 – 2.88 (m, 2H), 2.69 (dd, *J* = 15.7, 10.7 Hz, 2H), 1.79 – 1.69 (m, 1H).
^{13}C NMR (126 MHz, DMSO) δ 169.0, 144.3, 125.8, 124.0, 62.1, 46.0, 34.9. The α-B carbon signal was not observed.
^{11}B NMR (160 MHz, DMSO) δ 12.7.
HRMS (ESI/TOF) *m/z*: [M+NH$_4$]$^+$ Calcd. For $C_{14}H_{20}BN_2O_4^+$ 291.1510; Found 291.1504.

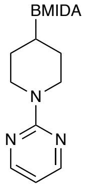

6-methyl-2-(1-(pyrimidin-2-yl)piperidin-4-yl)-1,3,6,2-dioxazaborocane-4,8-dione (3t). Prepared according to a modified General procedure C with B_2cat_2 (142.6 mg, 0.60 mmol, 3 equiv) and 2-(4-bromopiperidin-1-yl)pyrimidine **(S2t)**. Work-up C was performed, and the reaction mixture was purified by silica gel chromatography (0–60% acetone/hexane gradient) followed by recrystallization to yield **3t** as a light brown solid (25.2 mg, 40%).
Analytical data for **3t**:

mp = decomposition at 245.0–246.1 ºC
FTIR (diamond, anvil, solid) cm^{-1}: 2991, 2948, 1763, 1587, 1503, 1336, 1285, 1028, 949.

^{1}**H NMR** (500 MHz, CD_3CN) δ 8.28 (dd, *J* = 4.7, 1.5 Hz, 2H), 6.49 – 6.46 (m, 1H), 4.78 – 4.70 (m, 2H), 3.92 (d, *J* = 1.7 Hz, 2H), 3.79 (d, *J* = 1.7 Hz, 2H), 2.94 (s, 3H), 2.87 – 2.74 (m, 2H), 1.72 – 1.65 (m, 2H), 1.29 (qd, *J* = 12.9, 4.1 Hz, 2H), 1.06 (t, *J* = 12.2 Hz, 1H).
^{13}**C NMR** (126 MHz, CD_3CN) δ 169.1, 162.8, 158.7, 110.2, 63.2, 46.4, 45.9, 27.7. The α-B carbon signal was not observed.
^{11}**B NMR** (160 MHz, CD_3CN) δ 12.6.
HRMS (ESI/TOF) *m/z*: $[M+H]^+$ Calcd. For $C_{14}H_{20}BN_4O_4^+$ 319.1572; Found 319.1571.

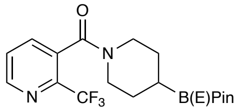

(4-(4,4,5,5-tetraethyl-1,3,2-dioxaborolan-2-yl)piperidin-1-yl)(2-(trifluoromethyl)pyridin-3-yl)methanone (3u). Prepared according to the General procedure C using (4-bromopiperidin-1-yl)(2-(trifluoromethyl)pyridin-3-yl)methanone **(S2u)**. Work-up A was performed, and the reaction mixture was purified by silica gel chromatography (0–20% acetone/hexane gradient) to yield **3u** as a clear oil (50.0 mg, 57%).
Analytical data for **3u**:

FTIR (diamond, anvil, oil) cm^{-1}: 2978, 2940, 2884, 1641, 1588, 1435, 1285, 1140, 1121, 996.
^{1}**H NMR** (500 MHz, $CDCl_3$) δ 8.73 (d, *J* = 4.6 Hz, 1H, A+B), 7.73 (d, *J* = 7.8 Hz, 0.56H, A), 7.67 (d, *J* = 7.8 Hz, 0.44H, B), 7.55 – 7.50 (m, 1H, A+B), 4.36 (dt, *J* = 13.2, 4.4 Hz, 0.56H, A), 4.24 (dt, *J* = 13.2, 4.7 Hz, 0.44H, B), 3.27 – 2.96 (m, 3H, A+B), 1.88 – 1.77 (m, 1H, A+B), 1.70 – 1.57 (m, 9H, A+B), 1.57 – 1.34 (m, 2H, A+B), 1.27 – 1.21 (m, 1H, A+B), 0.89 (t, *J* = 7.5 Hz, 12H, A+B).
^{13}**C NMR** (126 MHz, $CDCl_3$) δ 165.22 (A), 165.21 (B), 149.63 (A+B), 143.8 (q, *J* = 34.4 Hz, B), 143.60 (q, *J* = 34.4 Hz, A), 136.1 (A), 136.0 (B), 131.8 (A+B), 126.50 (A), 126.45 (B), 121.5 (q, *J* = 275.5 Hz, B), 121.4 (q, *J* = 275.5 Hz, A), 88.54 (B), 88.52 (A), 48.6 (A), 48.5 (B), 43.1 (A+B), 27.6 (A), 27.2 (B), 27.0 (A), 26.8 (B), 26.53 (B), 26.48 (A), 19.8 (A+B), 8.91 (A), 8.87 (B), 8.85 (A), 8.84 (B).
^{19}**F NMR** (470 MHz, $CDCl_3$) δ -64.8, -65.3.
^{11}**B NMR** (160 MHz, $CDCl_3$) δ 32.6.
HRMS (ESI/TOF) *m/z*: $[M+H]^+$ Calcd. For $C_{22}H_{33}BF_3N_2O_3^+$ 441.2531; Found 441.2531.

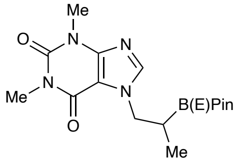

1,3-dimethyl-7-(2-(4,4,5,5-tetraethyl-1,3,2-dioxaborolan-2-yl)propyl)-3,7-dihydro-1H-purine-2,6-dione (3v). Prepared according to a modified General procedure C which B_2cat_2 (142.6 mg, 0.60 mmol, 3 equiv) was used with 7-(2-bromopropyl)-1,3-dimethyl-3,7-dihydro-1H-purine-2,6-dione (**S2v**). Work up A was performed, and the reaction mixture was purified by silica gel chromatography (0–25% acetone/hexane gradient) to yield **3v** as a brown oil (41.5 mg, 51%).
Analytical data for **3v**:

FTIR (diamond, anvil, oil) cm^{-1}: 2970, 2942, 1701, 1656, 1545, 1457, 1368, 1286, 1107, 1024, 919.
^{1}H NMR (500 MHz, $CDCl_3$) δ 7.60 – 7.56 (m, 1H), 4.40 (ddd, *J* = 13.6, 7.6, 1.9 Hz, 1H), 4.18 (ddd, *J* = 13.5, 8.5, 1.9 Hz, 1H), 3.57 – 3.56 (m, 3H), 3.40 – 3.38 (m, 3H), 1.78 (q, *J* = 7.4 Hz, 1H), 1.64 – 1.54 (m, 8H), 0.97 (dd, *J* = 7.6, 2.0 Hz, 3H), 0.89 – 0.82 (m, 12H).
^{13}C NMR (126 MHz, $CDCl_3$) δ 155.2, 151.9, 148.9, 141.7, 107.2, 88.8, 50.1, 29.8, 28.1, 26.4, 26.4, 19.4, 13.1, 8.9, 8.8.
^{11}B NMR (160 MHz, $CDCl_3$) δ 33.8.
HRMS (ESI/TOF) *m/z*: $[M+H]^+$ Calcd. For $C_{20}H_{34}BN_4O_4^+$ 405.2668; Found 405.2669.

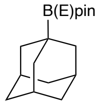

2-(adamantan-1-yl)-4,4,5,5-tetraethyl-1,3,2-dioxaborolane (3w). Prepared according to General procedure C using 1-bromoadamantane **(S2w)**. Work-up A was performed, and the reaction mixture was purified by silica gel chromatography (0–3% EtOAc/hexane gradient) to yield **3w** as a clear oil (48.9 mg, 77%).
Analytical data for **3w**:

FTIR (diamond, anvil, oil) cm^{-1}: 2970, 2897, 2843, 1693, 1450, 1344, 1284, 1070, 927.
^{1}H NMR (500 MHz, $CDCl_3$) δ 1.84 (br s, 3H), 1.75 (t, *J* = 3.3 Hz, 12H), 1.61 (m, 8H), 0.89 (t, *J* = 7.5 Hz, 12H).
^{13}C NMR (126 MHz, $CDCl_3$) δ 87.5, 38.4, 37.7, 27.8, 26.6, 8.9. The α-B carbon signal was not observed.
^{11}B NMR (160 MHz, $CDCl_3$) δ 32.6.
HRMS (ESI/TOF) *m/z*: $[M+Na]^+$ Calcd. For $C_{20}H_{35}BNaO_2^+$ 341.2622; Found 341.2623.

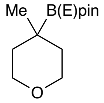

4,4,5,5-tetraethyl-2-(4-methyltetrahydro-2*H*-pyran-4-yl)-1,3,2-dioxaborolane (**3x**). Prepared according to a modified General procedure C in which B_2cat_2 (142.7 mg, 0.60 mmol, 3 equiv) was used with (3-bromo-3-methylbutyl)benzene **(S2X)**. Work up A was performed, and the reaction mixture was purified by silica gel chromatography (0-6% EtOAc/hexanes), fractions containing product were combined and concentrated and resubjected to silica gel chromatography (0-4% Et_2O/pentane gradient to yield **3x** as a clear oil (27.8 mg, 49%).
Analytical data for **3x**:

FTIR (diamond, anvil, oil) cm^{-1}: 2971, 2946, 2884, 1458, 1388, 1294, 1105, 927.
^{1}H NMR (500 MHz, $CDCl_3$) δ 3.84 (ddd, *J* = 11.6, 4.1, 2.5 Hz, 2H), 3.41 (td, *J* = 11.7, 2.1 Hz, 2H), 1.79 – 1.72 (m, 2H), 1.70 – 1.60 (m, 8H), 1.31 – 1.21 (m, 2H), 0.95 (s, 3H), 0.90 (t, *J* = 7.5 Hz, 12H).
^{13}C NMR (126 MHz, $CDCl_3$) δ 88.4, 67.4, 36.9, 26.2, 26.0, 8.8. The α-B carbon signal was not observed.
^{11}B NMR (160 MHz, $CDCl_3$) δ 34.7.
HRMS (EI-QTOF) *m/z*: $[M]^+$ Calcd. For $C_{16}H_{31}BO_3^+$, 282.2366, Found 282.2367

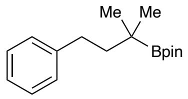

4,4,5,5-tetramethyl-2-(2-methyl-4-phenylbutan-2-yl)-1,3,2-dioxaborolane (3y). Prepared according to a modified General procedure C in which B_2cat_2 (142.7 mg, 0.60 mmol, 3 equiv) was used with (3-bromo-3-methylbutyl)benzene **(S2y)**. A modified work-up B (TBAF, 5 equiv) was performed where TBAF was added to the DMA mixture and extracted with EtOAc. Then, the reaction mixture was purified by silica gel chromatography (0–2% Et_2O/hexanes gradient) to yield **3y** as a clear oil (35.8 mg, 65%).
Analytical data for **3y**:

^{1}H NMR (500 MHz, $CDCl_3$) δ 7.31 – 7.24 (m, 2H), 7.23 – 7.13 (m, 3H), 2.61 – 2.54 (m, 2H), 1.62 – 1.57 (m, 2H), 1.27 (s, 12H), 1.01 (s, 6H).
^{13}C NMR (126 MHz, $CDCl_3$) δ 143.8, 128.5, 128.4, 125.6, 83.1, 43.7, 33.2, 24.9, 24.9. The α-B carbon signal was not observed.
Product matched literature characterization.[11]

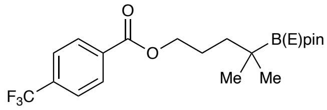

4-methyl-4-(4,4,5,5-tetraethyl-1,3,2-dioxaborolan-2-yl)pentyl 4-(trifluoromethyl)benzoate (3z). Prepared according to modified General procedure C in which B_2cat_2 (142.7 mg, 0.60 mmol, 3 equiv) was used with 4-(3-bromo-3-methylbutyl)-2-methoxyphenol **(S2z)**. Work-up B was performed, and the reaction mixture was purified by silica gel chromatography (0–2% EtOAc/hexane gradient) to yield 3z as a colorless oil (67.2 mg, 74%)

Analytical data for **3z**:

FTIR (diamond, anvil, oil) cm^{-1}: 2971, 2940, 2884, 1725, 1474, 1324, 1273, 1166, 1130, 1065, 1017, 923.
^{1}H NMR (500 MHz, $CDCl_3$) δ 8.16 (d, J = 8.0 Hz, 2H), 7.70 (d, J = 8.1 Hz, 2H), 4.32 (t, J = 6.6 Hz, 2H), 1.76 (dq, J = 10.6, 6.7 Hz, 2H), 1.68 – 1.59 (m, 8H), 1.43 – 1.36 (m, 2H), 0.96 (s, 6H), 0.89 (t, J = 7.4 Hz, 12H).
^{13}C NMR (126 MHz, $CDCl_3$) δ 165.6, 134.27 (d, J = 32.5 Hz)134.0, 130.1, 125.47 (q, J = 3.8 Hz), 123.84 (q, J = 272.7 Hz) 88.1, 66.7, 37.3, 26.3, 25.8, 25.2, 20.3, 8.9.
^{19}F NMR (470 MHz, CDCl3) δ -63.1.
^{11}B NMR (160 MHz, $CDCl_3$) δ 34.2.
HRMS (ESI/TOF) *m/z*: $[M+Na]^+$ Calcd. For $C_{24}H_{36}BF_3NaO_4^+$ 479.2551; Found 479.2559.

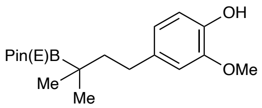

2-methoxy-4-(3-methyl-3-(4,4,5,5-tetraethyl-1,3,2-dioxaborolan-2-yl)butyl)phenol (3aa). Prepared according to General procedure C using 4-(3-bromo-3-methylbutyl)-2-methoxyphenol **(S2aa)**. Work-up B was performed, and the reaction mixture was purified by silica gel chromatography (0–3% EtOAc/hexane gradient) to yield **3aa** as a colorless oil (49.5 mg, 66%)
Analytical data for **3aa**:

FTIR (diamond, anvil, oil) cm^{-1}: 3557, 2935, 2859, 1609, 1514, 1462, 1351, 1264, 1187, 923.
^{1}H NMR (500 MHz, $CDCl_3$) δ 6.82 (d, *J* = 7.9 Hz, 1H), 6.71 – 6.66 (m, 2H), 5.44 (s, 1H), 3.88 (s, 3H), 2.54 – 2.47 (m, 2H), 1.74 – 1.59 (m, *J* = 7.2 Hz, 8H), 1.57 – 1.51 (m, 2H), 0.99 (s, 6H), 0.93 (t, *J* = 7.5 Hz, 12H).
^{13}C NMR (126 MHz, $CDCl_3$) δ 146.4, 143.5, 136.0, 120.9, 114.2, 111.1, 88.1, 56.0, 44.1, 32.9, 26.4, 25.2, 20.8, 8.9.
^{11}B NMR (160 MHz, $CDCl_3$) δ 34.7.
HRMS (ESI/TOF) *m/z*: [M-H]$^-$ Calcd. For $C_{22}H_{36}BO_4^-$ 375.2712; Found 375.2718.

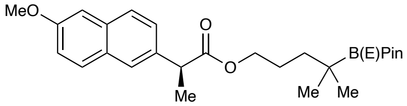

4-methyl-4-(4,4,5,5-tetraethyl-1,3,2-dioxaborolan-2-yl)pentyl (S)-2-(6-methoxynaphthalen-2-yl)propanoate (3ab). Prepared according to a modified General procedure C in which B_2cat_2 (190 mg, 0.80 mmol, 3 equiv) was used with 4-bromo-4-methylpentyl (S)-2-(6-methoxynaphthalen-2-yl)propanoate (**S2ab).** Work-up B was performed (TBAF, 5 equiv), and the reaction mixture was purified by silica gel chromatography (0–5% Et_2O/hexane gradient) to yield **3y** as a pale-yellow oil (69.2 mg, 70%)
Analytical data for **3ab**:

FTIR (diamond, anvil, oil) cm^{-1}: 2969, 2859, 1731, 1634, 1606, 1365, 1264, 1157, 1033, 888.
^{1}H NMR (500 MHz, $CDCl_3$) δ 7.72 – 7.66 (m, 3H), 7.42 (dd, *J* = 8.5, 1.9 Hz, 1H), 7.15 – 7.10 (m, 2H), 4.07 – 4.01 (m, 2H), 3.91 (s, 3H), 3.84 (q, *J* = 7.1 Hz, 1H), 1.66 – 1.50 (m, 13H), 1.23 – 1.18 (m, 2H), 0.90 – 0.83 (m, 18H).
^{13}C NMR (126 MHz, $CDCl_3$) δ 174.9, 157.7, 136.1, 133.8, 129.4, 129.1, 127.2, 126.5, 126.1, 119.0, 105.7, 88.0, 65.9, 55.4, 45.7, 37.1, 26.3, 25.6, 25.1, 18.7, 8.9.
^{11}B NMR (160 MHz, $CDCl_3$) δ 33.8.
HRMS (ESI/TOF) *m/z*: [M+Na]$^+$ Calcd. For $C_{30}H_{45}BNaO_5^+$ 519.3252; Found 519.3242.

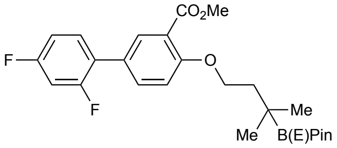

methyl 2',4'-difluoro-4-(3-methyl-3-(4,4,5,5-tetraethyl-1,3,2-dioxaborolan-2-yl)butoxy)-[1,1'-biphenyl]-3-carboxylate (3ac). Prepared according to the general procedure using methyl 4-(3-bromo-3-methylbutoxy)-2',4'-difluoro-[1,1'-biphenyl]-3-carboxylate **(S2z)**. Work-up B was performed, and the reaction mixture was purified by silica gel chromatography (0–2% EtOAc/hexane gradient) to yield **3ac** as a pale-yellow oil (52.0 mg, 50%).
Analytical data for **3ac**:

FTIR (diamond, anvil, oil) cm^{-1}: 2972, 2947, 2885, 1732, 1611, 1573, 1491, 1254, 1160, 910, 732.
^{1}H NMR (500 MHz, $CDCl_3$) δ 7.89 (s, 1H), 7.57 (d, *J* = 8.7 Hz, 1H), 7.47 – 7.34 (m, 1H), 7.10 (d, *J* = 8.7 Hz, 1H), 7.01 – 6.83 (m, 2H), 4.19 (t, *J* = 7.7 Hz, 2H), 3.89 (s, 3H), 1.93 – 1.81 (m, 2H), 1.73 – 1.56 (m, 8H), 1.03 (s, 6H), 0.91 (t, *J* = 7.5 Hz, 12H).
^{13}C NMR (126 MHz, $CDCl_3$) δ 166.9, 162.3 (dd, *J* = 248.8, 11.8 Hz), 159.8 (dd, *J* = 250.1, 11.8 Hz), 158.4, 133.8 (d, *J* = 3.5 Hz), 132.0 (d, *J* = 2.4 Hz), 131.3 (dd, *J* = 9.4, 4.9 Hz), 126.6, 124.3

(dd, J = 13.6, 3.9 Hz), 120.8, 113.6, 111.7 (dd, J = 21.0, 3.8 Hz), 104.5 (dd, J = 26.7, 25.2 Hz), 88.3, 67.8, 52.2, 39.4, 26.4, 25.6, 8.9. The α-B carbon signal was not observed.
^{19}F NMR (470 MHz, $CDCl_3$) δ -111.7, -113.7.
^{11}B NMR (160 MHz, $CDCl_3$) δ 34.8.
HRMS (ESI/TOF) *m/z*: $[M+Na]^+$ Calcd. For $C_{29}H_{39}BF_2NaO_5^+$ 539.2751; Found 539.2750.

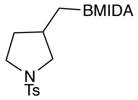

5-methyl-2-((1-tosylpyrrolidin-3-yl)methyl)-1,5,2-oxazaborepane-3,7-dione (7). Prepared according to General procedure C using *N*-allyl-*N*-(2-bromoethyl)-4-methylbenzenesulfonamide (**S7).** Work-up C was performed, and the reaction mixture was purified by silica gel chromatography (0–60% acetone/hexane gradient) followed by recrystallization to yield **7** as a tan solid (41.5 mg, 53%).
Analytical data for **7**:

mp = 188.5–189.6 ºC
FTIR (diamond, anvil, solid) cm^{-1}: 2970, 2886, 1773, 1748, 1514, 1336, 1229, 1158, 986.
^{1}H NMR (500 MHz, DMSO) δ 7.67 (d, J = 8.0 Hz, 2H), 7.43 (d, J = 8.0 Hz, 2H), 4.16 (d, J = 7.8 Hz, 1H), 4.13 (d, J = 7.7 Hz, 1H), 3.93 (d, J = 17.0 Hz, 2H), 3.41 – 3.34 (m, 1H), 3.28 – 3.22 (m, 1H), 3.15 – 3.02 (m, 1H), 2.74 (s, 3H), 2.69 – 2.58 (m, 1H), 2.40 (s, 3H), 1.98 – 1.84 (m, 2H), 1.43 – 1.14 (m, 1H), 0.66 – 0.38 (m, 2H).
^{13}C NMR (126 MHz, DMSO) δ 168.9, 168.8, 143.2, 133.5, 129.8, 127.2, 61.44, 61.41, 55.0, 47.5, 45.4, 34.8, 33.7, 21.0. 18.6.
^{11}B NMR (160 MHz, DMSO) δ 11.4.
HRMS (ESI/TOF) *m/z*: $[M+Na]^+$ Calcd. For $C_{17}H_{23}BN_2NaO_6S^+$ 417.1262; Found 417.1262.

Further Transformations

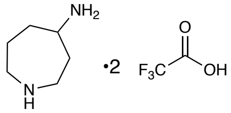

azepan-4-amine 2,2,2-trifluoroacetate (**4**).
The title compound was prepared according to a modified procedure from Morken and co-workers.[18] A two neck round bottom flask, equipped with magnetic stir bar and reflux condenser under nitrogen atmosphere was charged with t-BuOK (67.3 mg, 0.60 mmol, 6.0 equiv), *o*-methylhydroxylammonium chloride (33.4 mg, 4 equiv), and **3o** (38.1 mg, 0.1 mmol, 1 equiv) and toluene (0.300 mL). The reaction mixture was heated to 80 °C and stirred for 16 h. The reaction mixture was then cooled to room temperature before Boc_2O (109 mg, 5.0 equiv) and 0.3 mL aqueous saturated $NaHCO_3$ were added. After refluxing under N_2 at 80 °C for 5 h, the mixture was cooled to room temperature, 5 mL of water were added, and the crude mixture was extracted with ethyl acetate. The organic layer was dried with Na_2SO_4, filtered, concentrated, and purified on silica gel (0–5% acetone/hexane gradient) to deliver the di-boc protected azepane. For ease of characterization, the boc protecting groups were removed by resuspending the boc-protected heterocycle in 1 mL DCM and cooling the mixture to 0 ºC, followed by dropwise addition of 0.5 mL trifluoroacetic acid (60 equiv). The reaction was

allowed to stir for 3 h followed by quenching with 5 mL of water. The aqueous layer was washed with DCM (3x5 mL) and concentrated to yield **4** (19.6 mg, 57%) as a clear crystalline solid.
Analytical data for **4**:

mp = 136.3-138.4 ºC
FTIR (diamond, anvil, oil) cm^{-1}: 3010, 2868, 1662, 1541, 1430, 1176, 1124, 836.
^{1}H NMR (500 MHz, D_2O) δ 3.56 – 3.40 (m, 2H), 3.39 – 3.29 (m, 1H), 3.27 – 3.15 (m, 2H), 2.43 – 2.17 (m, 2H), 2.02 (dq, *J* = 20.4, 9.3 Hz, 2H), 1.90 – 1.77 (m, 1H), 1.74 – 1.62 (m, 1H).
^{13}C NMR (126 MHz, D_2O) δ 162.8 (q, *J* = 35.3 Hz), 116.3 (q, *J* = 291.7 Hz), 50.4, 45.5, 41.2, 30.6, 28.4, 20.0.
^{19}F NMR (470 MHz, D_2O) δ -75.6.
HRMS (ESI/TOF) *m/z*: $[M–C_4HF_6O_4]^+$ For $C_6H_{15}N_2$+ 115.1230; Found 115.1222.

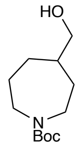

***tert*-butyl 4-(hydroxymethyl)azepane-1-carboxylate (5)** Prepared according to a modified literature procedure from Hartwig and co-workers.[19] To a flame-dried 5 mL round-bottom flask under nitrogen atmosphere was added **3o** (38.1 mg, 0.1 mmol, 1 equiv), dibromomethane (43.5 mg, 0.25 mmol, 2.5 equiv) and THF (1 mL, 0.1 M). The resulting solution was cooled to –78 °C then *n*-butyllithium (1.6 M in hexanes, 137.5 µL, 0.220 mmol, 2.2 equiv) was added dropwise. The reaction was allowed to warm to room temperature and stir for 4 h. The reaction mixture was then cooled to 0 °C and a solution of 2N NaOH/30% H_2O_2 (2:1 v/v, 3.0 mL) was added dropwise. The reaction mixture was allowed to warm to room temperature and stir overnight. The reaction mixture was diluted with Et_2O, followed by further extraction with Et_2O. The combined organic layers were dried with Na_2SO_4, concentrated *in vacuo*, and purified by silica gel chromatography (0–60% EtOAc/hexane gradient) to yield **5** as a colorless oil (13.6 mg, 59%).
Analytical data for **5**:

^{1}H NMR (500 MHz, $CDCl_3$) (mixture of rotamers) δ 3.69 – 3.10 (m, 6H), 1.93 – 1.68 (m, 3H), 1.62 – 1.54 (m, 2H), 1.45 (s, 9H), 1.37 – 1.27 (m, 1H), 1.27 – 1.08 (m, 1H).
^{13}C NMR (126 MHz, $CDCl_3$) (mixture of rotamers) δ 155.8, 155.7, 79.22, 79.18, 68.2, 68.1, 61.0, 47.1, 46.9, 46.4, 46.2, 45.7, 45.3, 44.9, 42.1, 41.5, 40.3, 35.9, 35.3, 35.2, 34.8, 33.8, 33.1, 31.5, 31.3, 30.1, 29.8, 29.6, 28.67, 28.65, 27.3, 27.2, 27.1, 27.0.
Product matched literature characterization.[20]

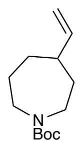

***tert*-butyl 4-vinylazepane-1-carboxylate (6).** Prepared according to a modified literature procedure by Aggarwal and co-workers.[21] To a flame-dried 5 mL round-bottom flask, **3o** (38.1 mg, 0.1 mmol, 1 equiv) was dissolved in THF (0.5 mL) and DMSO (0.5 mL). The flask was cooled to 0 °C and vinyl magnesium bromide in a 1.0 M THF solution was added dropwise (0.24 mL, 0.24 mmol, 2.4 equiv). The resulting mixture was allowed to warm to room temperature and stirred for 1 h. After 1 h, the mixture was cooled to 0 °C and a suspension of NaOMe (3M in

MeOH, 0.10 mL, 0.30 mmol, 3.0 equiv) was added quickly, followed by the addition of I_2 dropwise (0.5 M in MeOH, 0.24 mL, 0.12 mmol, 1.2 equiv). The resulting mixture was stirred at 0 °C for 1 h and then sat. sodium thiosulfate (~2 mL) was added, and the organic layer was extracted with DCM. The combined organic layers were dried with Na_2SO_4, concentrated *in vacuo*, and purified by silica gel column chromatography (0–10% EtOAc/hexane gradient) to yield **6** as a colorless oil (caution: volatile) (11.0 mg, 49%).
Analytical data for **6**:

^{1}H NMR (500 MHz, $CDCl_3$) δ 5.80 (ddd, *J* = 17.3, 10.4, 7.0 Hz, 1H), 5.00 – 4.93 (m, 1H), 4.91 – 4.87 (m, 1H), 3.65 – 3.15 (m, 4H), 2.16 – 2.07 (m, 1H), 1.92 – 1.73 (m, 3H), 1.65 – 1.55 (m, 1H), 1.52 – 1.42 (m, 10H), 1.36 – 1.27 (m, 1H).
^{13}C NMR (126 MHz, $CDCl_3$) δ 155.8 (A+B), 144.1 (A+B), 112.1 (A+B), 79.2 (A+B), 46.8 (A), 46.3 (B), 45.1 (A), 44.7 (B), 43.4 (A), 42.8 (B), 34.7 (A), 34.5 (B), 33.4 (A), 32.8 (B), 28.7 (A+B), 27.0 (A), 26.8 (B).
Product matched literature characterization.[22]

Additional and Unsuccessful Substrates

Additional Substrates

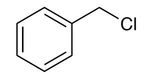

43% ^{1}H NMR yield
3 equiv B_2cat_2

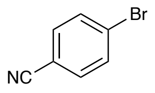

46% ^{1}H NMR yield
2 equiv B_2cat_2

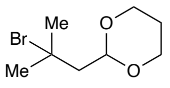

19% isolated yield
3 equiv B_2cat_2

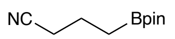

82% ^{1}H NMR yield
2 equiv B_2cat_2

Limitations

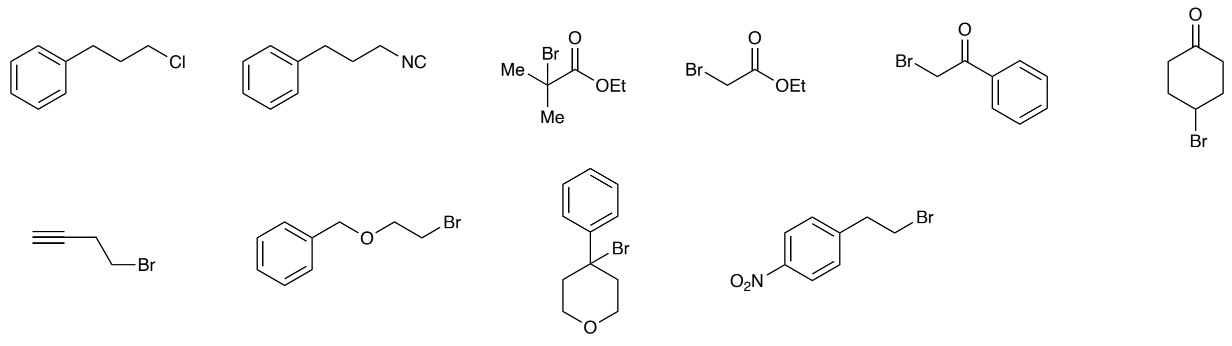

Radical Trapping experiments

TEMPO trapping

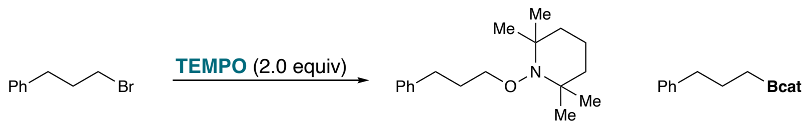

	8	3-Bcat
standard conditions:	*observed by ESI-HRMS*	*not observed*
no B_2cat_2:	*observed by ESI-HRMS*	*N/A*
no hv:	*observed by ESI-HRMS*	*not observed*
no hv & no B_2cat_2:	*not observed*	*not observed*

In an N_2 inert atmosphere glovebox, **1a** (19.9 mg, 0.10 mmol), **2** (82.0 mg, 0.20 mmol), B_2cat_2 (47.6 mg, 0.20 mmol), and TEMPO (31.3 mg, 0.20 mmol) were added sequentially to a flame-dried 2-dram vial equipped with a magnetic stir bar. The mixture was suspended in DMA (1.0 mL, 0.1 M) and sealed with a cap. The vial was removed from the glovebox and irradiated with stirring using two Kessil PhotoReaction PR160L 427 nm LEDs at 100% intensity with fan cooling. After 14 hours of irradiation, the vial was removed, and samples were taken for ESI-HRMS and GC-MS analysis. **3-Bcat** was not observed by GC-MS analysis.
*Note: Three additional TEMPO trapping experiments were performed: 1) no B_2cat_2, 2) no light, 3) no light & no B_2cat_2.

Analytical data for TEMPO experiment

Standard Conditions:
2,2,6,6-tetramethyl-1-(3-phenylpropoxy)piperidine (8):
HRMS (ESI/TOF) m/z: [M+H]+ Calcd. For $C_{18}H_{30}NO^+$: 276.2322, Found 276.2330.

No B_2cat_2:
2,2,6,6-tetramethyl-1-(3-phenylpropoxy)piperidine (8):
HRMS (ESI/TOF) m/z: [M+H]+ Calcd. For $C_{18}H_{30}NO^+$: 276.2322, Found 276.2322.

No hv:
2,2,6,6-tetramethyl-1-(3-phenylpropoxy)piperidine (8):
HRMS (ESI/TOF) m/z: $[M+H]^+$ Calcd. For $C_{18}H_{30}NO^+$: 276.2322, Found 276.2322.

1,1-Diphenylethylene Trapping

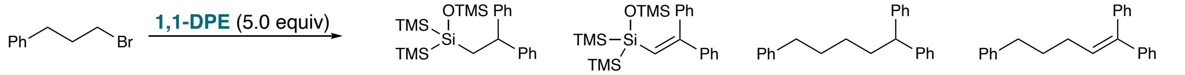

	9	10	11	12
standard conditions:	*observed by HRMS*	*observed by HRMS*	*not observed*	*observed by LRMS*
no B_2cat_2:	*not observed*	*observed by HRMS*	*not observed*	*observed by LRMS*
no hv:	*not observed*	*trace detection by HRMS*	*not observed*	*not observed*
no hv & no B_2cat_2:	*not observed*	*not observed*	*not observed*	*not observed*

In an N_2 inert atmosphere glovebox, **1a** (10.0 mg, 0.05 mmol), **2** (41.0 mg, 0.10 mmol), B_2cat_2 (23.8 mg, 0.10 mmol), and 1,1-DPE (45.1 mg, 0.50 mmol) were added sequentially to a flame-dried 2-dram vial equipped with a magnetic stir bar. The mixture was suspended in DMA (0.5

mL, 0.1 M) and sealed with a cap. The vial was removed from the glovebox and irradiated with stirring using two Kessil PhotoReaction PR160L 427 nm LEDs at 100% intensity with fan cooling. After 14 hours of irradiation, the vial was removed, and samples were taken for EI-QTOF analysis. **3-Bcat** was not observed by GC-MS analysis.

Analytical data for 1,1-DPE experiment

1-(2,2-diphenylethyl)-3,3,3-trimethyl-1,1-bis(trimethylsilyl)disiloxane (9):
HRMS (EI-QTOF) *m/z*: $[M]^+$ Calcd. For $C_{23}H_{40}OSi_4^+$, 444.2156, Found 444.2158.

1-(2,2-diphenylvinyl)-3,3,3-trimethyl-1,1-bis(trimethylsilyl)disiloxane (10):
HRMS (EI-QTOF) *m/z*: $[M]^+$ Calcd. For $C_{23}H_{38}OSi_4^+$, 442.2000, Found 442.2006.

Discussion:

hv/no B_2cat_2 experiments

TEMPO trap: The formation of **8** under light irradiation and no B_2cat_2 indicates that **2** is likely photolabile upon direct photoexcitation, leading to fragmentation and liberation of the siloxy radical ^{2}**IV**.

1,1-DPE trap: The observation of **10** in the 1,1-DPE trapping experiment under light irradiation with no B_2cat_2 is further evidence that **2** is photolabile upon direct photoexcitation.

The energy barrier for photoexcitation of **2** leading directly to fragmentation is significantly higher than that for the formation of the proposed complex **I** (52.0 vs 25.1 kcal/mol; Figure S28). Therefore, we conclude that direct fragmentation of **2** is likely not the dominant radical initiation pathway, although it is likely operative to some extent.

No hv/B_2cat_2 experiments

TEMPO trap: The presence of **8** under no light irradiation indicates that there is likely a light independent initiation pathway of complex **I** to generate the boryl radical species and subsequent silyl radical generation. This is supported by our reaction control experiments (main text Table 1, entries 10 & 11) where product formation was observed. Thermal initiation and light independent processes have been observed in the literature for these diboron-mediated processes.[16, 23]

1,1-DPE trap: The trace presence of **10** under no light irradiation further supports that there is likely some trace light independent initiation pathway of complex **I**.

These experiments are in agreement with our no light reaction control and heating experiments.

UV-Vis studies

UV/Vis data was collected at room temperature under a nitrogen atmosphere. Solutions were prepared in freeze-pump-thawed DMA or DMF purchased from Sigma-Aldrich and measured in a quartz cuvette.

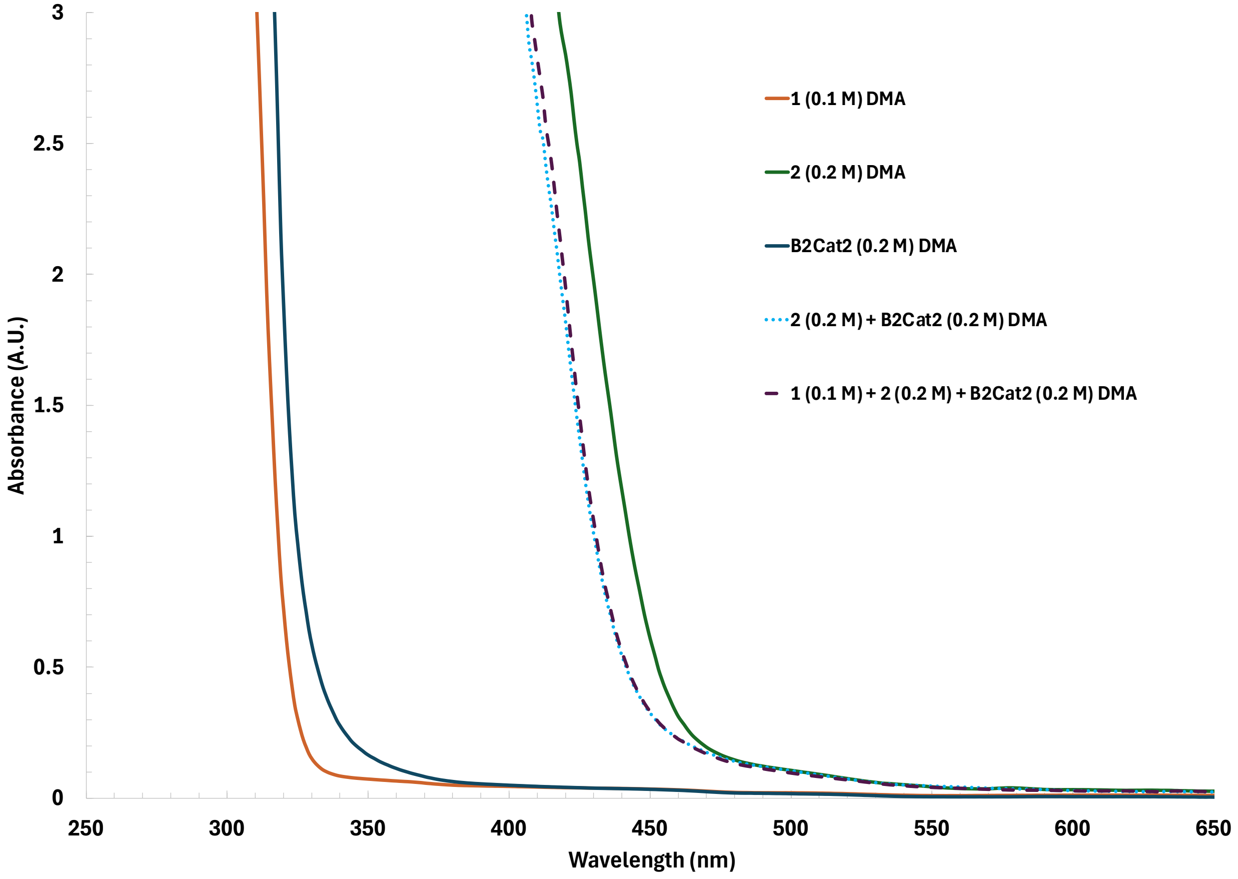

Figure S4. UV Vis spectra of reaction contents in DMA solvent

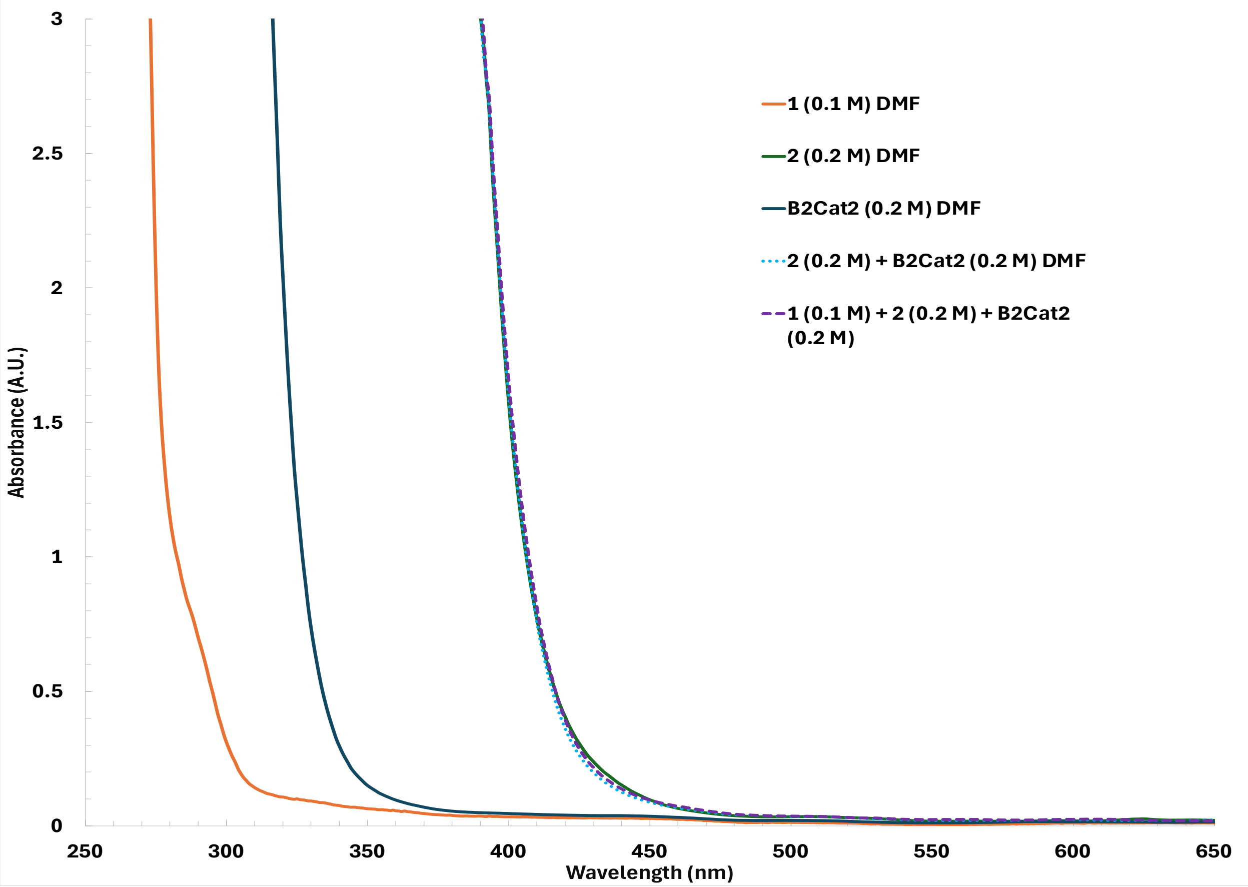

Figure S5. UV Vis spectra of reaction contents in DMF solvent

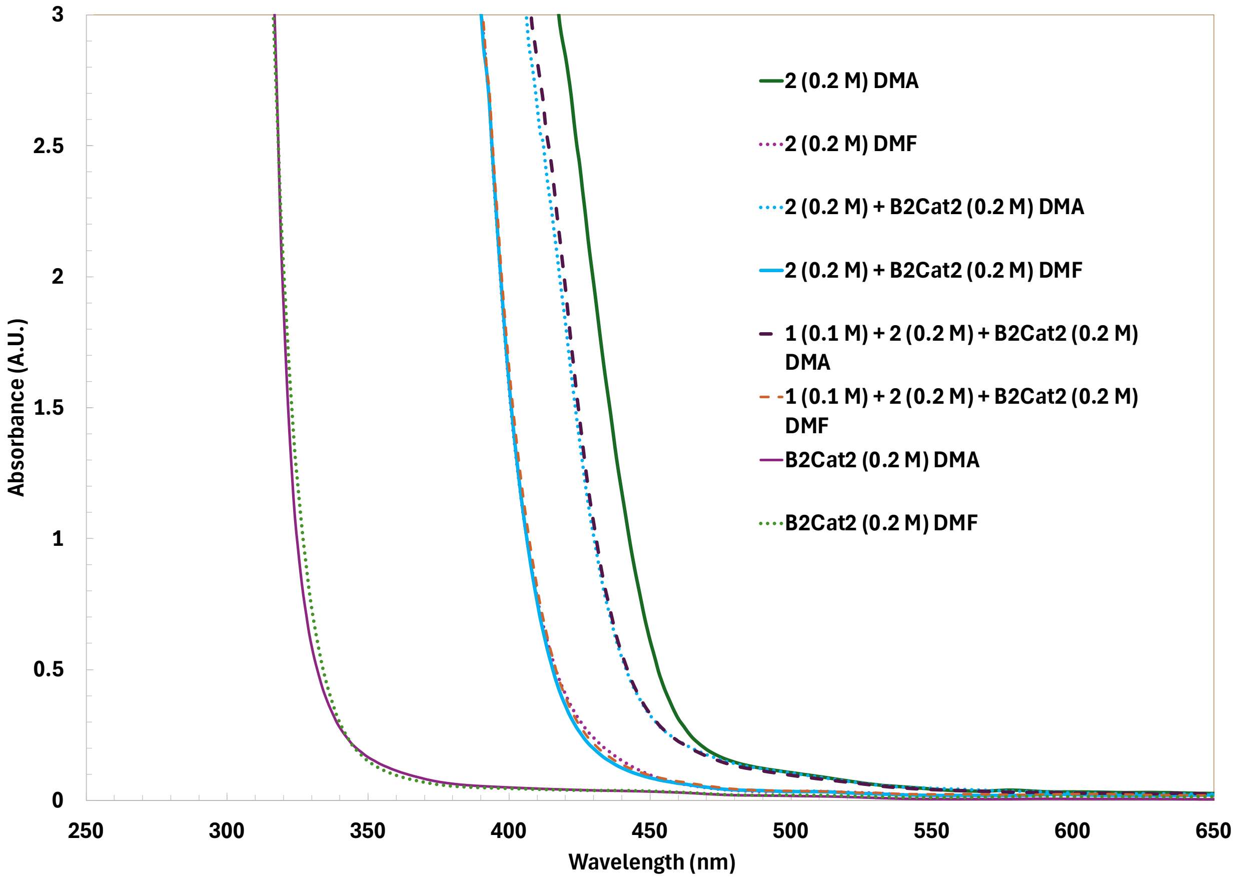

Figure S6. Comparison reaction contents in DMA vs DMF solvent.

Determination of Light Intensity

The photon flux of the spectrophotometer was determined from standard ferrioxalate actinometry[20, 21] following a modified procedure from Yoon and coworkers.[22] A 0.15 M solution of ferrioxalate was prepared by dissolving 1.105 g of potassium ferrioxalate hydrate in 15 mL of 0.05 M H_2SO_4. A buffered phenanthroline solution was prepared by dissolving 25 mg of phenanthroline and 5.625 g of sodium acetate in 25 mL of 0.5 M H_2SO_4. Both solutions were immediately wrapped in foil and stored in the dark. To determine the photon flux, 2.0 mL of the ferrioxalate solution was charged in a 2-dram vial with stir bar in the dark, and the vial placed 10.2 cm away from Kessil PR160L 427 nm light source. The 2-dram was irradiated for 10 seconds at 100% intensity while stirring. After irradiation, 0.35 mL of the phenanthroline solution was added to the 2-dram, and the vial was capped and wrapped in aluminum foil. The solution was then allowed to rest for 1 h in the dark to allow the ferrous ions to completely coordinate to the phenanthroline. After 1 h, absorbance of the solution was measured at 510 nm. A non-irradiated sample was also prepared in the same manner and the absorbance at 510 nm measured. Irradiated and non-irradiated samples were prepared in triplicate. Conversion was calculated using eq 1.

Equation (1) $$mol\ Fe^{2+} = (V * \Delta A)/(l * \varepsilon)$$

Where V is the total volume (0.00235 L) of the solution after addition of phenanthroline, ΔA is the difference in absorbance at 510 nm between the irradiated and non-irradiated solutions, l is the path length (1.000 cm), and ε is the molar absorptivity at 510 nm (11,100 L mol^{-1} cm^{-1}).[20] The mol Fe^{2+} measured (average of three experiments) was 3.975 $x10^{-7}$ mol.

The photon flux was calculated using eq 2

Equation (2) $$proton\ flux = mol\ Fe^{2+}/(\Phi * t * f)$$

Where Φ is the quantum yield for the ferrioxalate actinometer (1.12 for a 0.15 M solution at λ = 416 nm, 1.11 for a 0.15 M solution at λ = 436 nm)[23] value used= 1.115, t is the time (10.0 s), and f is the fraction of light absorbed at λ = 427 nm (0.999, *vide infra).* The photon flux was calculated (average of three experiments) to be 3.57 $x10^{-8}$ einstein/s.

Determination of fraction of light absorbed at 427 nm for the ferrioxalate solution:
The absorbance of the ferrioxalate solution at 427 nm was measured to be 3.43. The fraction of light absorbed (*f*) by this solution was calculated using equation 3, where A is the measured absorbance at 427 nm. By Equation 3, f = 0.999

Equation (3) $$f = 1 - 10^{-A}$$

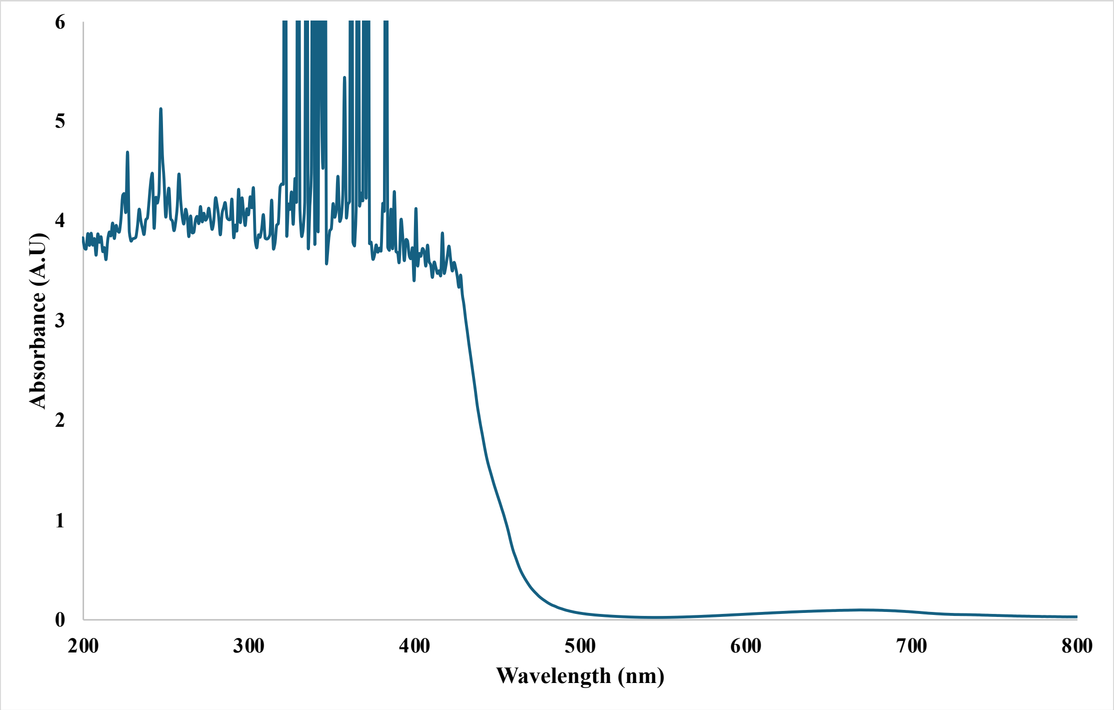

Figure S7. Absorbance spectrum of 0.15 M ferrioxalate actinometer solution.

Determination of Quantum Yield

In an N_2 inert atmosphere glovebox, **1a** (39.8 mg, 0.1 mmol), **2** (164.0 mg, 0.20 mmol), and B_2cat_2 (95.1 mg, 0.20 mmol) were added sequentially to a 2-dram vial. The mixture was suspended in DMA (2 mL, 0.1 M) and sealed with a cap, and the mixture was gently shaken to ensure all components were dissolved. The 2-dram was removed from the glovebox and was then irradiated for 10 min (600 s) seconds with stirring 10.2 cm away from Kessil PR160L 427 nm light source at 100% intensity. The 2-dram was removed, and 2,3-dimethylbutane-2,3-diol (141.8 mg, 1.2 mmol, 6 equiv) and triethylamine (0.4 mL) were added to the solution. After 1 hour of stirring, saturated sodium bicarbonate was added (~6 mL), and the organic layer was extracted with ethyl acetate until the organic layer was colorless (~6 mL EtOAc x 4). The combined organic layers were dried with Na_2SO_4 and concentrated for ^1H NMR analysis. The yields were determined by ^1H NMR with 1,3,5-trimethylbenzene as an internal standard. This experiment was repeated in triplicate and results are in **Table S2**. The average ^1H NMR yield was calculated to be (12.2 mg, 0.0000496 mol, 24.8%)

Note: Although small amounts of product form in the absence of light, it was determined that this process is negligible in the 10-minute timeframe.

The absorbance of the reaction mixture was measured to be 1.28 at 427 nm (*vide infra)*. Using Equation 3, f= 0.947. The average quantum yield of this experiment was then calculated with equation 4 to be 2.44.

Trial	^1H NMR yield (mg, µmol, %)	Quantum yield
1	14.2 mg, 57.6 µmol, 28.8%	2.84
2	10.2 mg, 41.4 µmol, 20.7%	2.04
3	12.2 mg, 49.6 µmol, 24.8%	2.45

Table S2: ^1H NMR yields and quantum yields of three standard reactions irradiated for 10 minutes with stirring 10.2 cm away from Kessil PR160L 427 nm light source at 100% intensity.

Equation (4) $$\Phi = \frac{mol\ pdt}{flux * t * f}$$

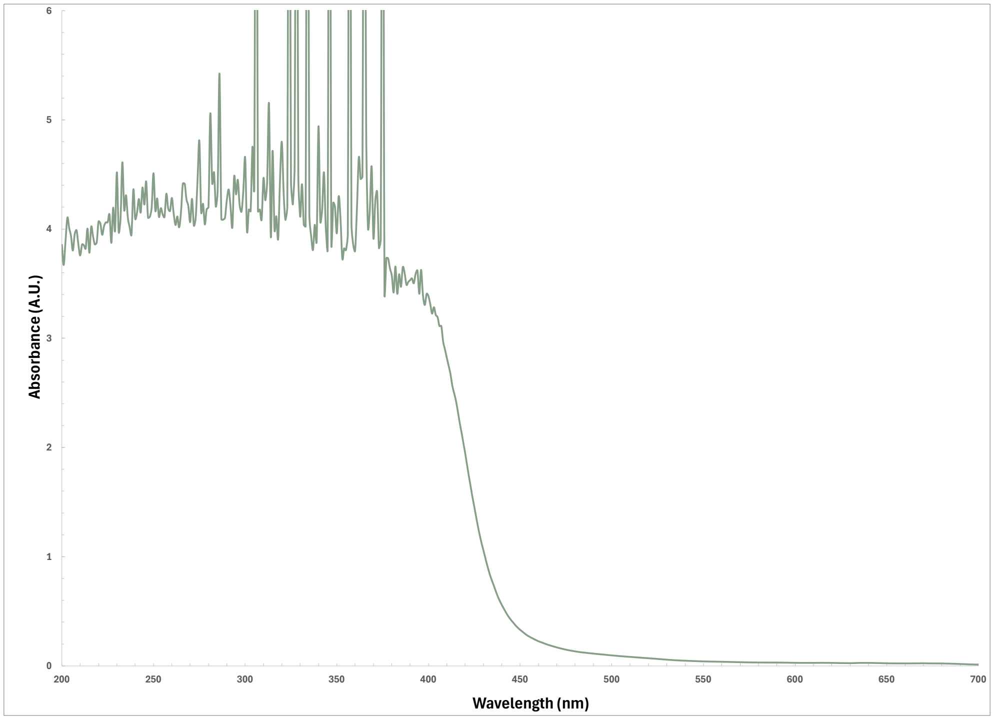

Figure S8. Absorbance spectrum of standard reaction conditions prepared according to General procedure C. The absorbance of the reaction mixture was measured to be 1.28 at 427 nm.

^{11}B NMR study

Sample A: A 0.1 M sample of B_2cat_2 (23.8 mg, 100 μmol) was dissolved in 1 mL of $CDCl_3$ in a glove box and transferred to a quartz NMR tube (Norell). Following ^{11}B NMR acquisition, the sample was returned to the glovebox and dimethylacetamide (343.8mg, 40 equiv) was added and the NMR tube was inverted multiple times to mix the sample. The sample was capped and sealed with parafilm. ^{11}B NMR spectrum was taken and the process was repeated with a further addition of 40 equiv dimethylacetamide for a final equiv of 80 w.r.t B_2cat_2.

Sample B: B_2cat_2 (23.8 mg, 100 μmol) and **2 (**41.0 mg, 100 μmol) was dissolved in 1 mL of $CDCl_3$ in a glove box and transferred to a quartz NMR tube (Norell). The same ^{11}B NMR acquisition and sequential addition of dimethylacetamide procedure was followed as described with Sample A.

Sample C: A 0.1 M sample of B_2cat_2 (23.8 mg, 100 μmol) was dissolved in 1 mL of $CDCl_3$ in a glove box and transferred to a quartz NMR tube (Norell). Following ^{11}B NMR acquisition, the sample was returned to the glovebox and dimethylformamide (292 mg, 40 equiv) was added and the NMR tube was inverted multiple times to mix the sample. The sample was capped and sealed with parafilm. ^{11}B NMR spectrum was taken and the process was repeated with a further addition of 40 equiv dimethylformamide (80 equiv final) w.r.t B_2cat_2.

Sample D: Sample B: B_2cat_2 (23.8 mg, 100 μmol) and **2 (**41.0 mg, 100 μmol) was dissolved in 1 mL of $CDCl_3$ in a glove box and transferred to a quartz NMR tube (Norell). The same ^{11}B NMR acquisition and sequential addition of dimethylformamide procedure was followed as described with Sample C.

Discussion: In $CDCl_3$, it appears that there is little to no interaction between B_2cat_2 and **2** (**Figure S9** vs **S10)** as there is no significant up-field shift or peak broadening (31.22 ppm vs 31.16 ppm respectively). Additionally, ^{13}C NMR of a 1:1 mixture of **2** and B_2cat_2 in CDCl3 showed no corresponding shift to for either reagent's carbon signals, indicating that there is no detectable interaction in $CDCl_3$.

With the addition of dimethylacetamide, a new sharp peak at ~14 ppm is observed that shifts up-field with every sequential addition of dimethylacetamide, as well as upfield shift and broadening of the original boron signal at ~31.2 ppm. We attribute the sharp peak at ~14 ppm to be the $[Bcat2]^-$ anion which has been invoked as a species in previous reports[23] and synthesized for confirmation (*vide infra*). The broadening of the peak at 29.9 ppm is indicative of a rapid interconversion of B_2cat_2 and B_2cat_2-DMA adduct.

We cannot exclude the possibility of Lewis basic interaction between the carbonyl oxygen lone pair of **2**, but because there is far more dimethylacetamide present in the reaction (1 equiv **2** vs 110 equiv dimethylacetamide with respect to B_2cat_2) and the corresponding up-field shift with addition of dimethylacetamide is present in both Sample A and B, it is likely that any interaction is weaker or it is present in a very low concentration not detectable by ^{11}B NMR.

When the experiment was repeated with DMF, the broadening of the and up-field sharp peak observed at ~14 ppm was still present, although the signal was not as strong compared to DMA.

Equiv Lewis basic solvent added	Sample A: ^{11}B NMR (160 MHz, $CDCl_3$)	Sample B: ^{11}B NMR (160 MHz, $CDCl_3$)	Sample C: ^{11}B NMR (160 MHz, $CDCl_3$)	Sample D: ^{11}B NMR (160 MHz, $CDCl_3$)
0	δ 31.22	δ 31.16	δ 31.18	δ 31.25
40	δ 29.43, 13.88	δ 29.68, 13.89	δ 29.26, 13.27	δ 27.79, 13.61
80	δ 27.95, 13.57	δ 29.48, 13.84.	δ 25.23, 13.46	25.58, 13.66,

Table S3: ^{11}B NMR signals of Lewis basic solvent titration

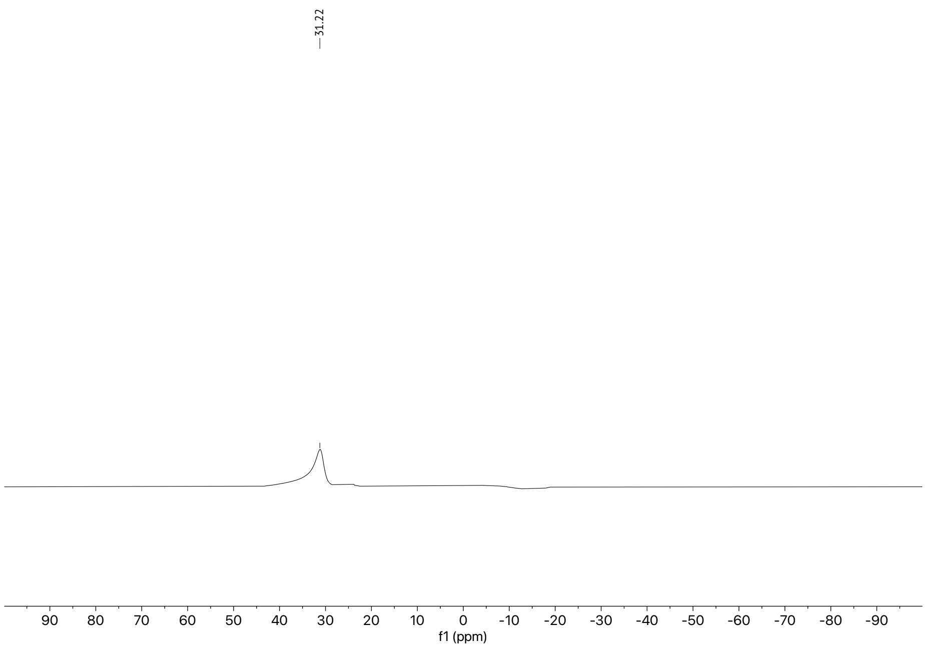

Figure S9. ^{11}B NMR (160 MHz, $CDCl_3$) of B_2cat_2

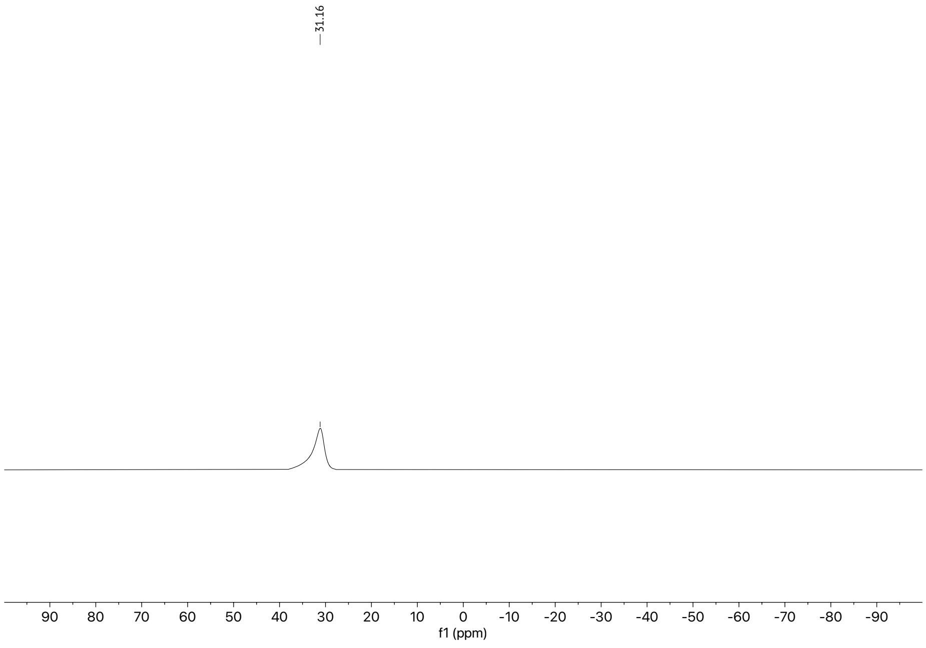

Figure S10. ^{11}B NMR (160 MHz, $CDCl_3$) of B_2cat_2 + **2**

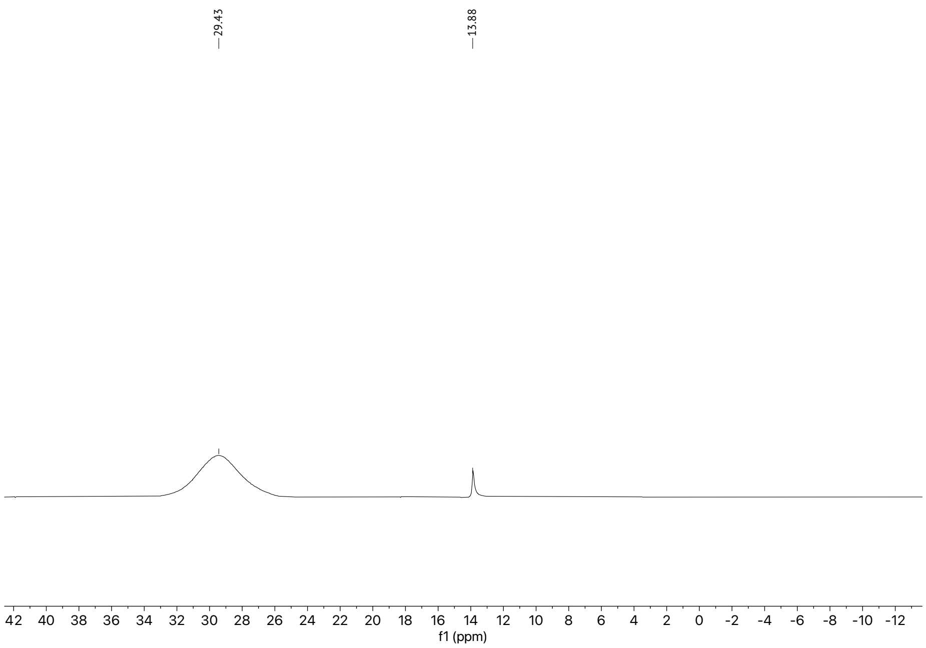

Figure S11. ^{11}B NMR (160 MHz, $CDCl_3$) of B_2cat_2 + 40 equiv DMA

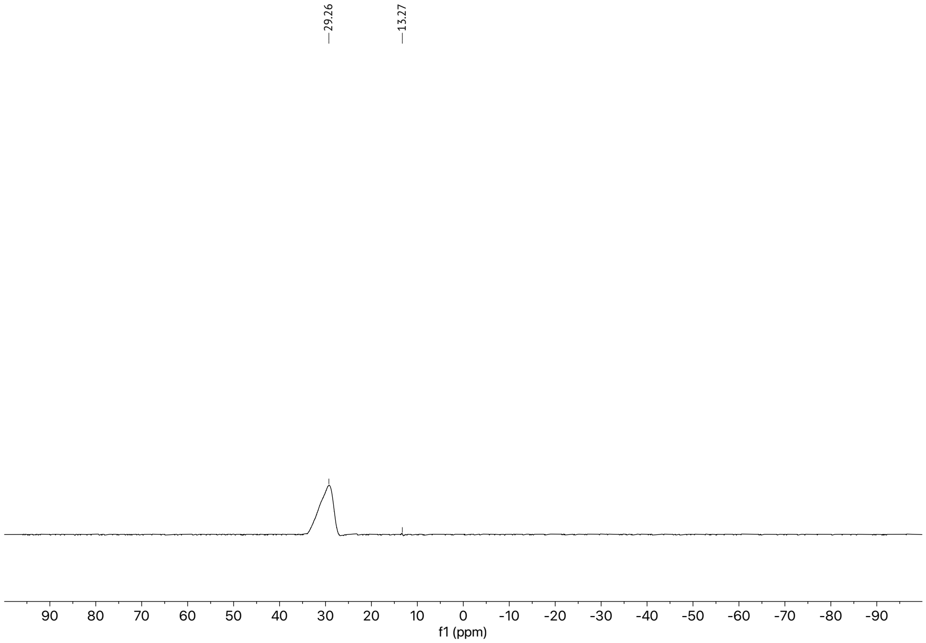

Figure S12. ^{11}B NMR (160 MHz, $CDCl_3$) of B_2cat_2 + 40 equiv DMF

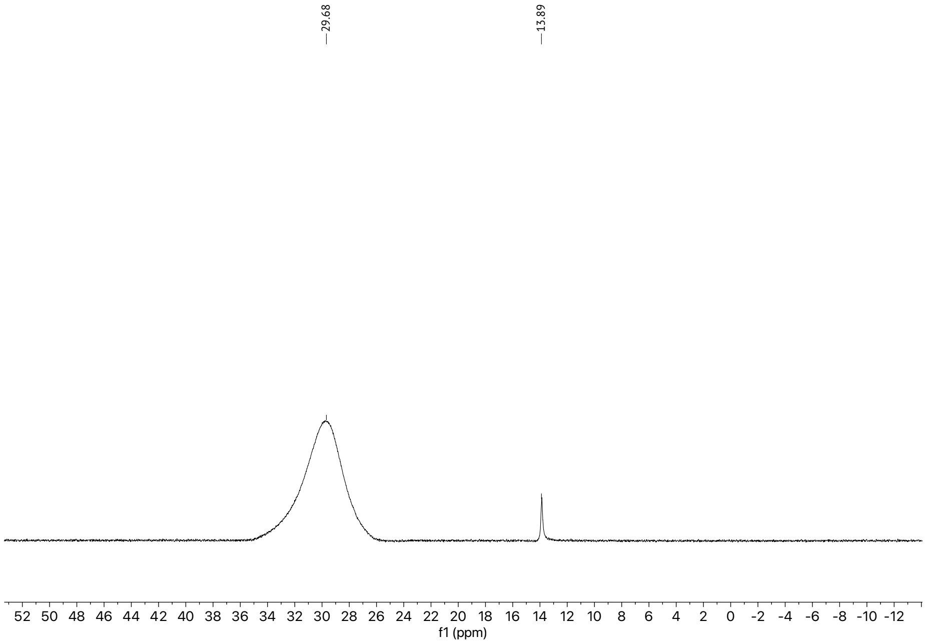

Figure S13. ^{11}B NMR (160 MHz, $CDCl_3$) B_2cat_2 + **2** + 40 equiv DMA

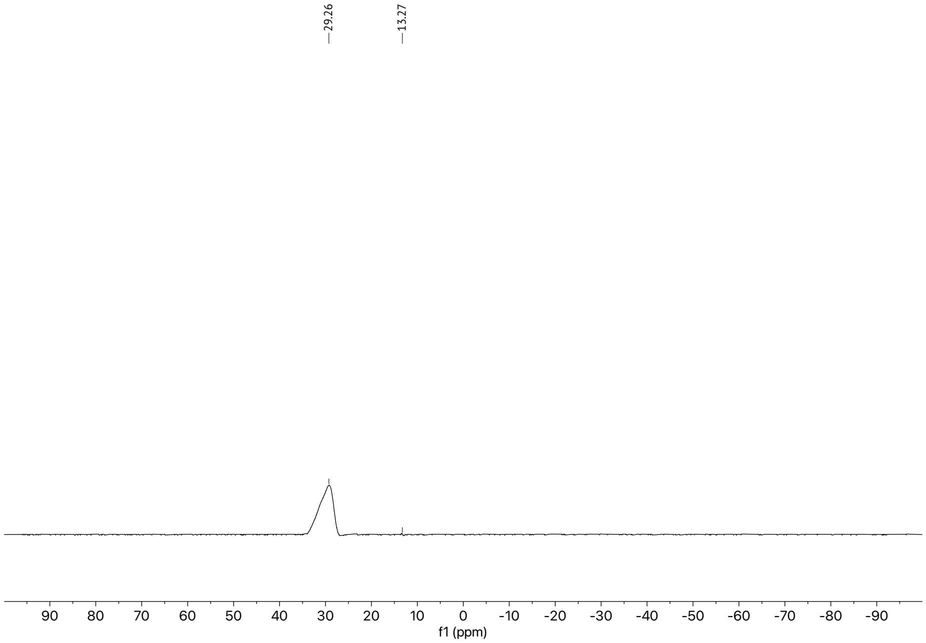

Figure S14. ^{11}B NMR (160 MHz, $CDCl_3$) B_2cat_2 + **2** + 40 equiv DMF

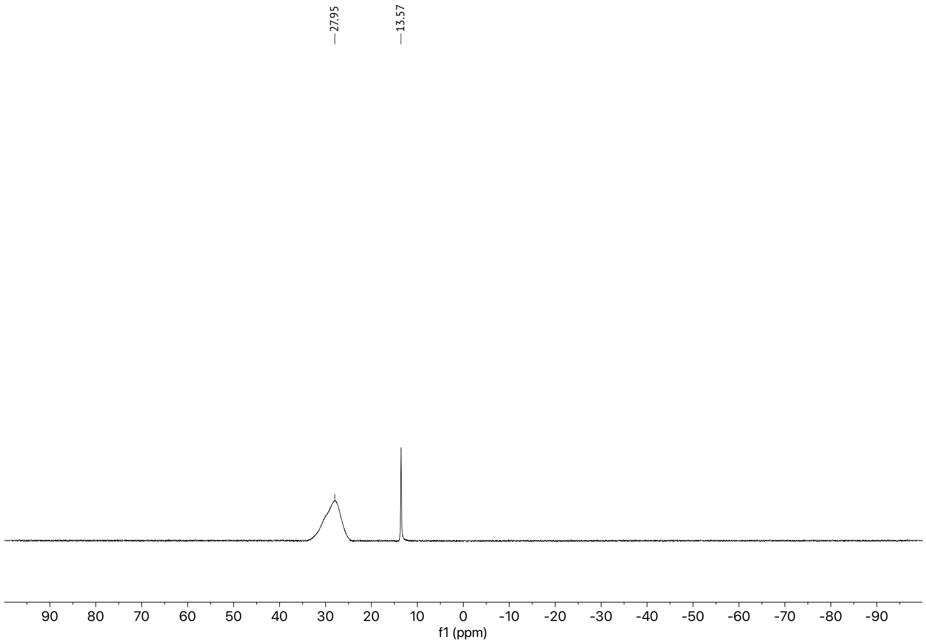

Figure S15. ^{11}B NMR (160 MHz, $CDCl_3$) B_2cat_2 + 80 equiv DMA

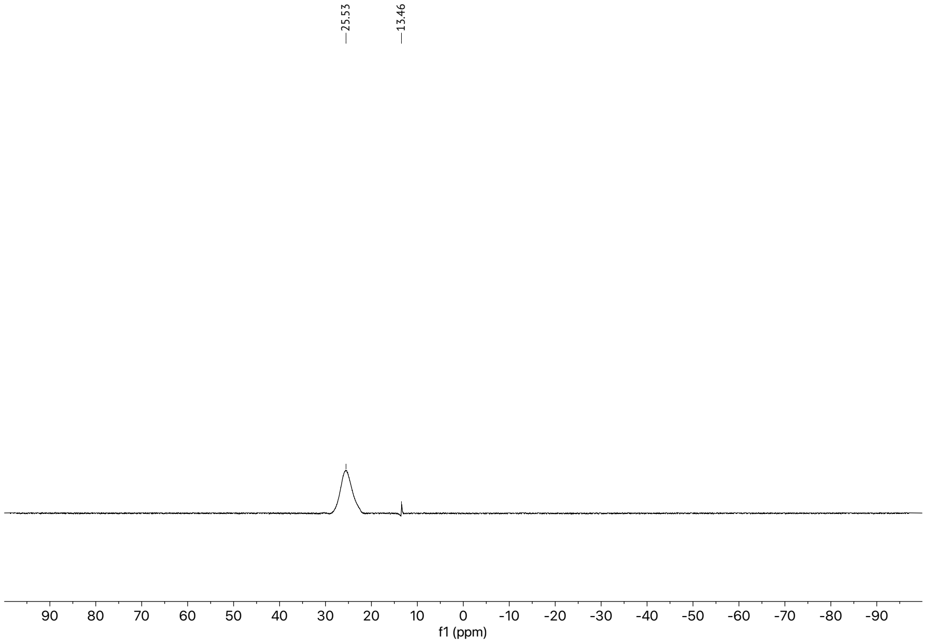

Figure S16. ^{11}B NMR (160 MHz, $CDCl_3$) B_2cat_2 + 80 equiv DMF

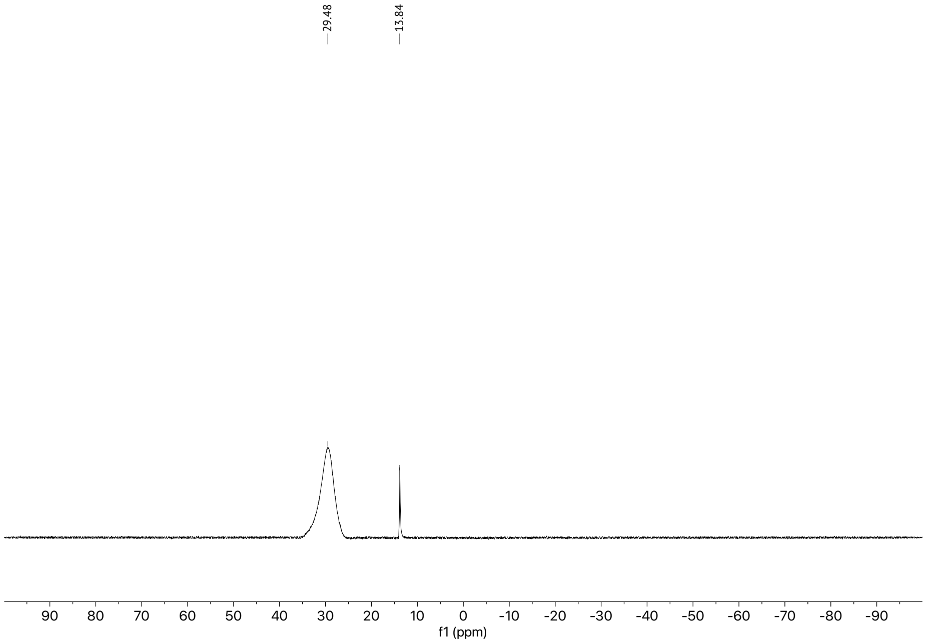

Figure S17. B_2cat_2 + **2** + 80 equiv DMA

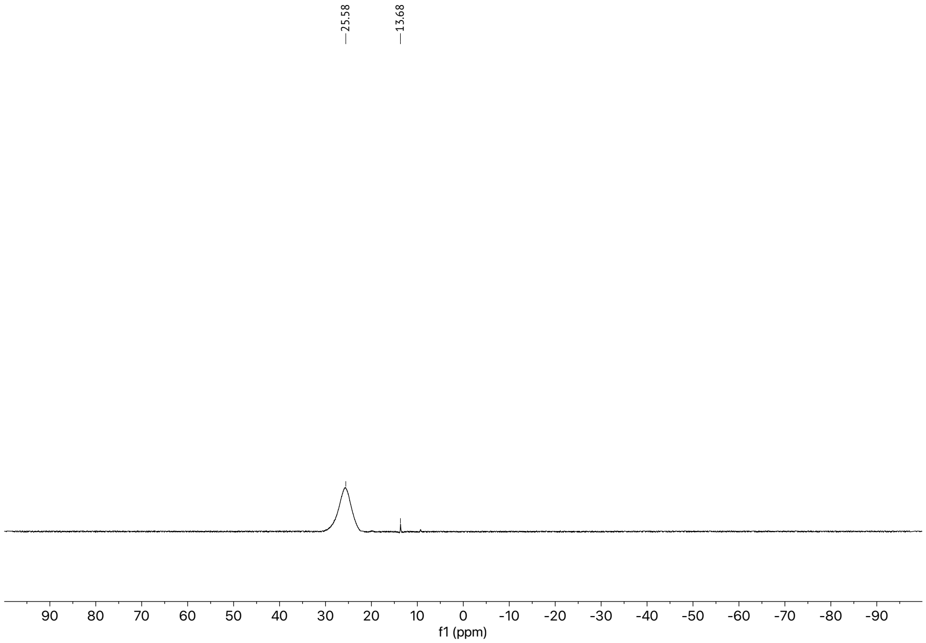

Figure S18. B_2cat_2 + **2** + 80 equiv DMF

Synthesis of NaBcat$_2$

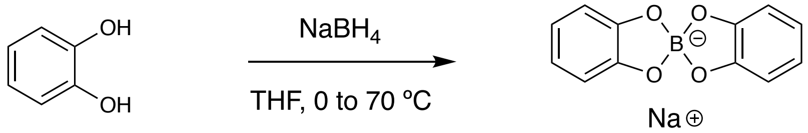

NaBcat$_2$ was prepared according to a reported procedure[23] by mixing catechol (220 mg, 2.0 mmol, 2.0 eq.) and NaBH$_4$ (38 mg, 1.0 mmol, 1.0 eq.) in anhydrous THF (5 mL) at 0 °C for 2 h, room temperature for 1 h and 70 °C for 1 h. The solvent was removed under vacuum and the resulting white solid was dissolved in 1:1 DMA:CDCl$_3$ for ^{11}B NMR analysis. ^{11}B NMR (160 MHz, 1:1 CDCl$_3$: DMA) δ 13.52.

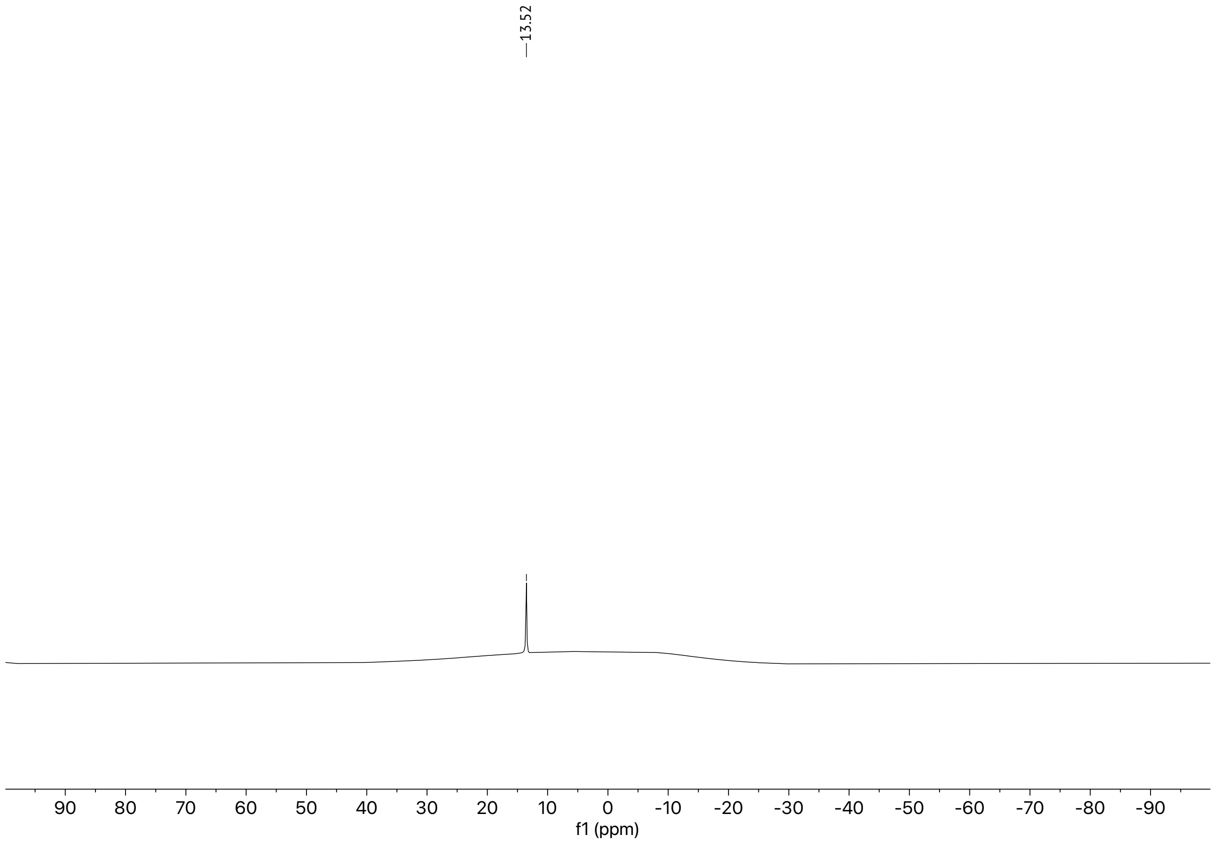

Figure S19. ^{11}B NMR of NaBcat$_2$ in 1:1 DMA: CDCl$_3$

Fluorescence Quenching studies

All emission spectra were obtained at right-angle detection on a Horiba Nanolog spectrofluorimeter (FL3-2iHR/iHR). Stern-Volmer emission studies were carried out at an ambient atmosphere in a 1mm path length quartz cuvette capped with a PTFE stopper. DMA was distilled immediately before use. Solutions were excited at 390 nm and a 410 nm filter was used in front of the detector to block pump scatter. An integration time of 8 seconds and 2.5 nm slit widths were employed for each scan to maximize signal to noise. The detector was parked at 500 nm, and spectra were worked up from 400-700 nm. Sample quenching studies were run with freshly prepared 6 mM solutions of **2** in DMA with varying concentrations (0 mM- 300 mM) B_2cat_2.The sample concentration of **2** was determined to achieve an optical density (OD) of 0.1 at an excitation wavelength of 390 nm, ensuring the detector was not oversaturated. A blank spectrum of DMA was taken and was subtracted out from all subsequently obtained spectra.

Stock solution **2** in DMA (mM)	Stock solution B_2cat_2 in DMA (mM)	Volume **2** (µL)	Volume B_2cat_2 (µL)	DMA standardizing volume (µL)	Total volume sample	**2** sample concentration (mM)	B2cat2 sample concentration	Equivalency B_2cat_2: **2**
60.0	600.0	300	0	2700	3000	6.0	0	0 : 1
60.0	600.0	300	300	2400	3000	6.0	60	10 : 1
60.0	600.0	300	600	2100	3000	6.0	120	20 : 1
60.0	600.0	300	1200	1500	3000	6.0	240	40 : 1
60.0	600.0	300	1500	1200	3000	6.0	300	50 : 1

Table S4: Sample preparation for Florescence quenching studies. Stock solutions were prepared immediately prior to use and samples were prepared under ambient conditions.

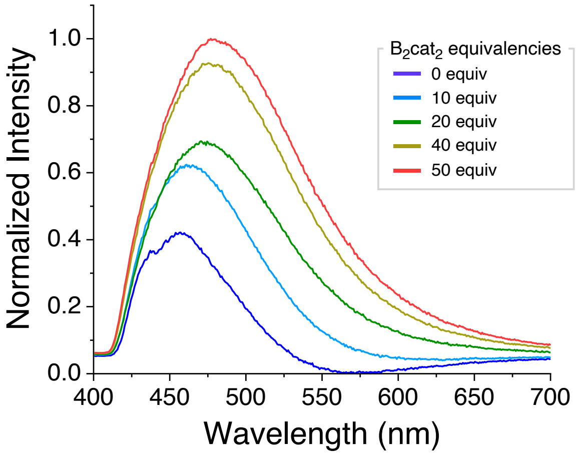

Figure S20. Fluorescence emission spectra of **2** with addition of B_2cat_2. Samples contained 6 mM of **2** with varying concentrations of B_2cat_2 (0–300 mM or 0–50 equiv w.r.t. **2**). Refer to **Table S4** for additional sample preparation details.

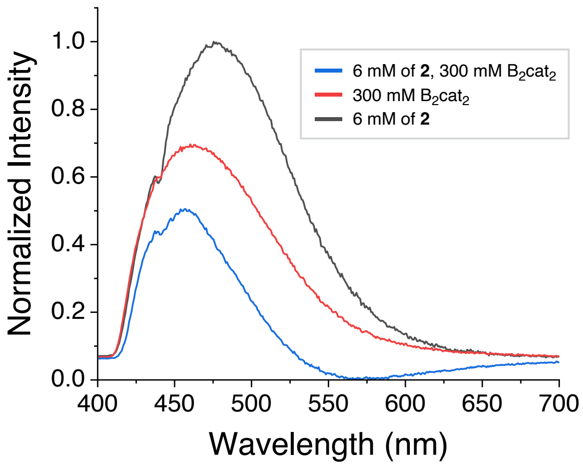

Figure S21. Comparison of fluorescence emission spectra of 6 mM sample of **2** (blue), 300 mM of B_2cat_2 (red) and 6 mM sample of 2 + 300 mM B_2Cat_2 (black).

Discussion: These experiments show that fluorescence of **2** is not quenched by varying concentrations of B_2cat_2, which indicates that a photoinduced electron transfer mechanism is not likely. We observe a minor red shift in fluorescence signal of **2** with the addition of B_2cat_2.

Cyclic Voltammetry studies

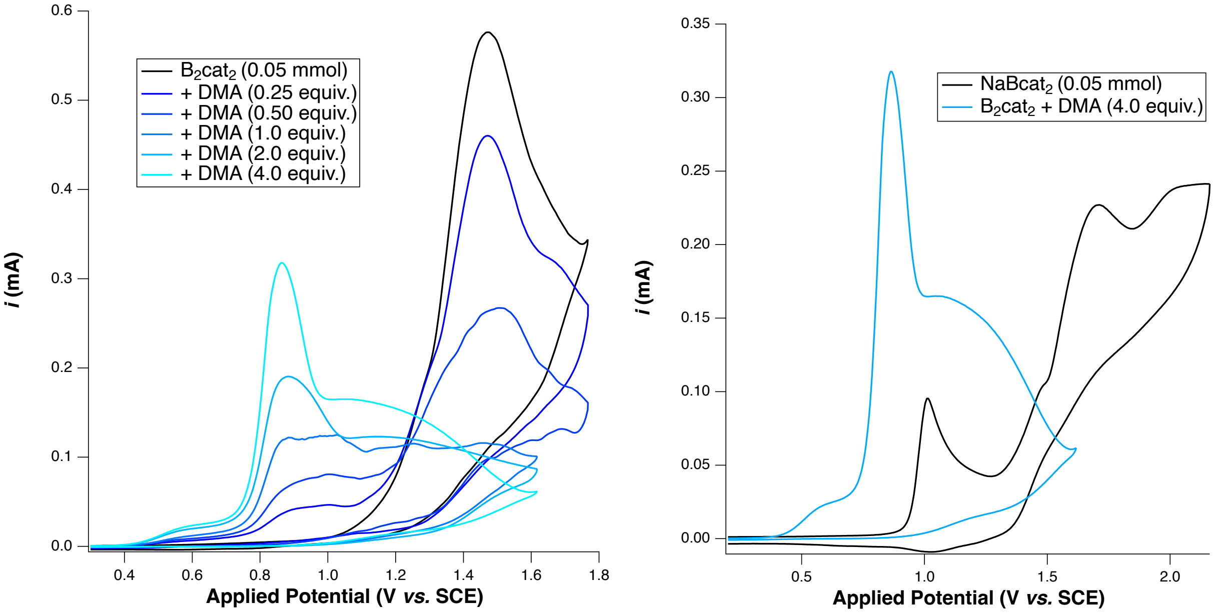

Figure S22. CV of B_2cat_2 (left) or $NaBcat_2$ (right) with titrated DMA.

An oven-dried 10 mL three-neck flask was charged with a magnetic stir bar, B_2cat_2 (0.05 mmol, 11.89 mg), and dried-recrystallized $TBAPF_6$ (0.5 mmol, 194 mg) and was purged with N_2. Solvent

system acetonitrile (5 mL) was added, and the resulting reaction mixture was degassed with N_2. Cyclic voltammograms were run with w.e. platinum (surface area = 0.0341 cm^2), c.e. platinum wire, and pseudoreference silver wire (referenced to $E(Fc^+/Fc)$) at a scan rate of 100 mV s^{-1}. Aliquots of DMA (0.25, 0.5, 1.0, 2.0, and 4.0 equiv) were added to the reaction mixture between scans and was magnetically stirred between scans. *iR* compensation was performed before each scan to ensure that no significant change in resistance resulted from the titration of DMA. The titration was repeated with DMF (0.25, 0.5, 1, 2, 4, 8, and 16 equiv).

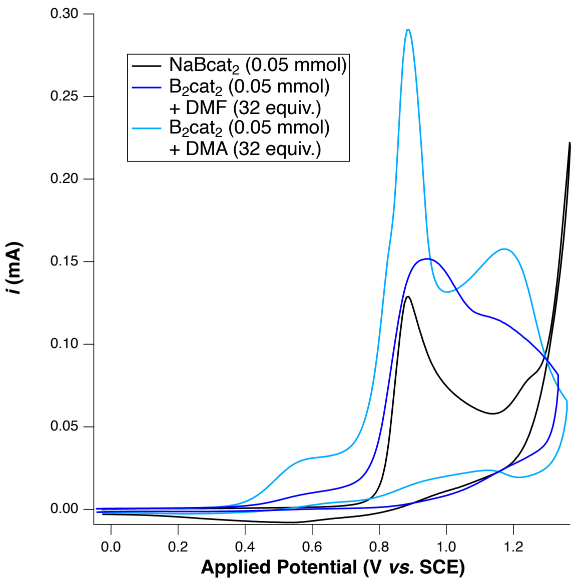

Figure S23. Comparison of B_2cat_2 with addition of DMA or DMF and $Bcat_2$

An oven-dried 10 mL three-neck flask was charged with a magnetic stir bar, $NaBcat_2$ (0.05 mmol, 12.50 mg) or B_2cat_2 (0.05 mmol, 11.89 mg), and dried-recrystallized $TBAPF_6$ (0.5 mmol, 194 mg) and was purged with N_2. Solvent system acetonitrile (5 mL) was added, and the resulting reaction mixture was degassed with N_2. Cyclic voltammograms were run with w.e. platinum (surface area = 0.0341 cm^2), c.e. platinum wire, and pseudoreference silver wire (referenced to $E(Fc^+/Fc)$) at a scan rate of 100 mV s^{-1}. DMA (32 equiv, 149 μL) or DMF (32 equiv, 125 μL) was added to the solution with B_2cat_2.

An oven-dried 10 mL three-neck flask was charged with a magnetic stir bar, B_2cat_2 (0.2 mmol, 47.6 mg), and dried-recrystallized $TBAPF_6$ (0.5 mmol, 194 mg) and purged. Solvent system acetonitrile (5 mL) was added, and the resulting reaction mixture was degassed. Cyclic voltammograms were run with w.e. platinum (surface area = 0.0341 cm^2), c.e. platinum wire, and pseudoreference silver wire (referenced to $E(Fc^+/Fc)$) at a scan rate of 100 mV s^{-1}.
Substrate (0.05 mmol, 1 equiv, 20.46 mg) was added to the mixture and CVs were run. The solution was magnetically stirred between scans.

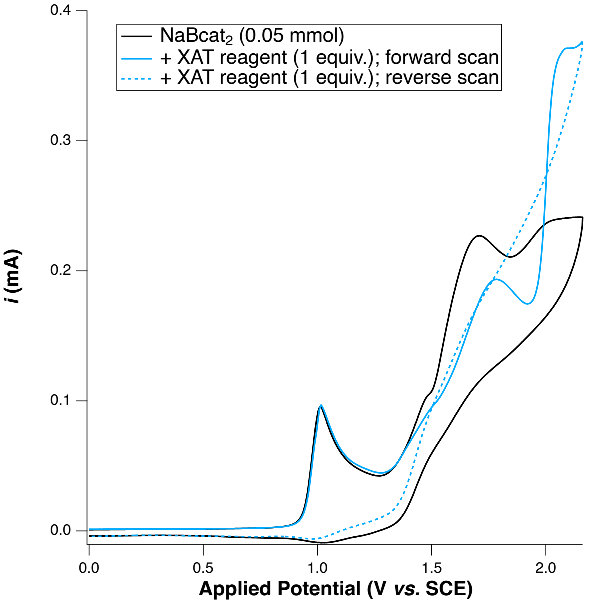

Figure S24. CV of **2** with $NaBcat_2$

An oven-dried 10 mL three-neck flask was charged with a magnetic stir bar, substrate (0.05 mmol, 12.5 mg), and dried-recrystallized $TBAPF_6$ (0.5 mmol, 194 mg) and was purged with N_2. Solvent system acetonitrile (5 mL) was added, and the resulting reaction mixture was degassed with N_2. Cyclic voltammograms were run with w.e. platinum (surface area = 0.0341 cm^2), c.e. platinum wire, and pseudoreference silver wire (referenced to E(Fc^+/Fc)) at a scan rate of 100 mV s^{-1}. *iR* compensation was performed before each scan.

2 (0.05 mmol, 1 equiv, 20.46 mg) was added to the mixture and CVs were run. The solution was magnetically stirred between scans.

Discussion: DMA titration (0.25–4 equiv) into a 5 mM solution of B_2cat_2 (100 mM $TBAPF_6$ in MeCN as the supporting electrolyte) yields a new species with a lower reduction potential as shown in (**Figure S22**). The results of this titration suggest the formation of a DMA-B_2cat_2 adduct that is easier to oxidize than the initial B_2cat_2 reagent. Additionally, $BCat_2^-$, showed similar features to the DMA-B_2cat_2 adduct, but comparison of the CVs shows that the $Bcat_2^-$ anion oxidizes at a higher potential than the DMA-B_2cat_2 adduct.

The titration was repeated with DMF additions. Though there was clear evidence for a new DMF-B_2cat_2 adduct formed *in situ*, this species had a higher reduction potential compared to the DMA analog, indicating a "more difficult" oxidation. Qualitatively, the titration was more sluggish with respect to the equivalents of DMF added (**Figure S23**).

We also wished to see if there was any interaction with the $Bcat_2^-$ species formed *in situ* (as observed in ^{11}B NMR studies) and **2**. $Bcat_2^-$ does not appear to have any interaction with **2** (**Figure S24**).

Computational Details

All geometry optimizations of intermediates and transition states were achieved using spin-unrestricted uB3LYP[24],[25] -D3[26] /def2SVP-CPCM[27],[28] method, in DMA solvent using the CPCM solvent model[29] with "opt=noeigen" and "guess=mix" keywords as implemented in Gaussian16.[38] Frequency calculations were also conducted at the same level of theory to obtain vibrational frequencies to determine the identity of stationary points as intermediates (no imaginary frequencies) or transition states (only one imaginary frequency), as well as obtaining the thermochemistry: enthalpy (ΔH) and free energy (ΔG) at the temperature of 298 K. Also, extensive conformation was performed and only the lowest-energy species were shown and discussed. All structural figures were generated with CYLview.[39] Distances in structural figures are shown in Å and energies are in kcal/mol. To compare energetics, we also carried out single-point energy calculations for the lowest energy structures using uB3LYP-D3/def2-TZVPP[27-28] CPCM(DMA), uM062X-D3/def2-TZVPP-CPCM(DMA), and uωB97XD/def2-TZVPP-CPCM(DMA).

Proposed Mechanism

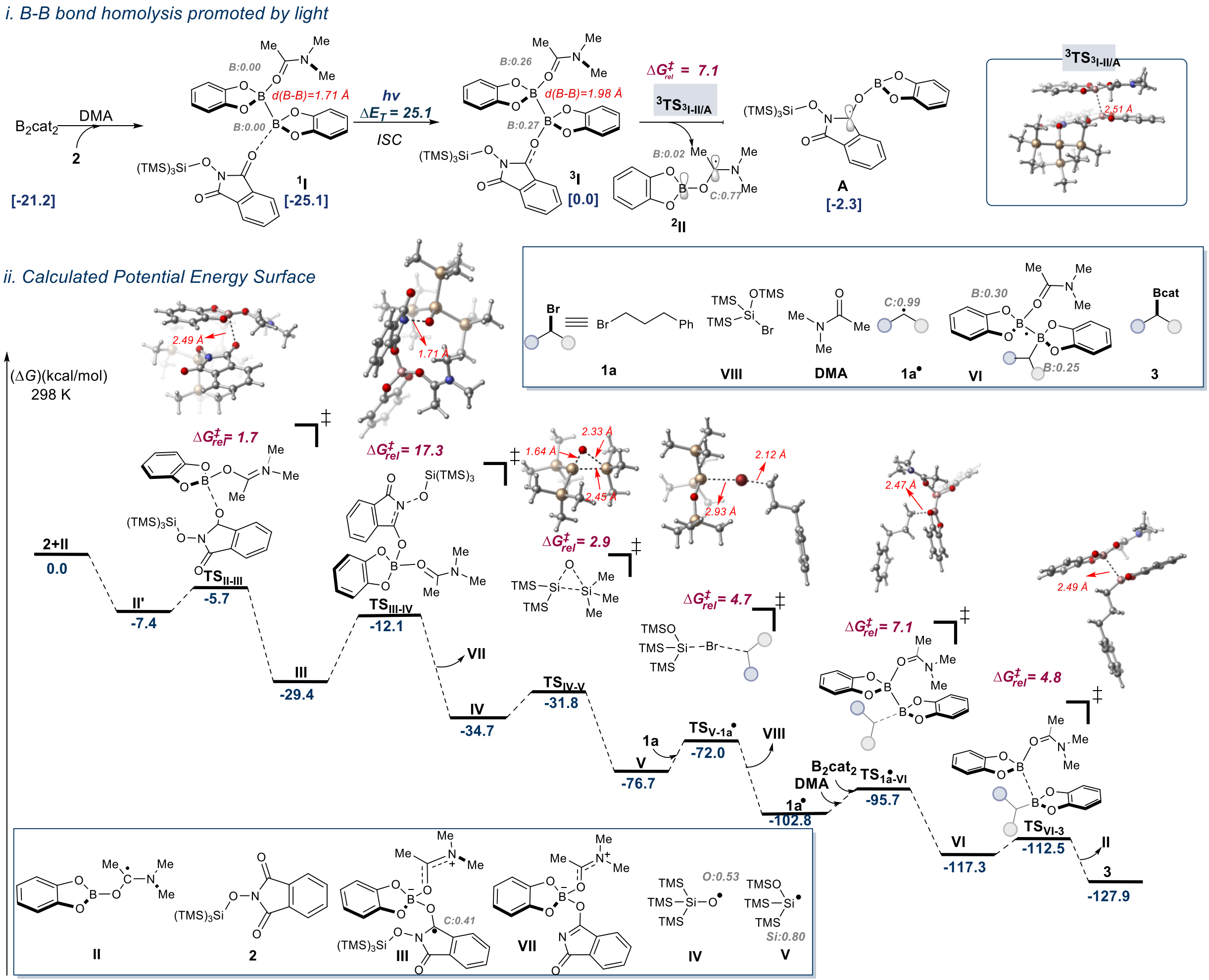

Figure S25: Proposed Mechanism supported by computational studies. Calculated Gibbs free energies [uB3LYP-D3/def2-svp-CPCM(DMA)] are given in kcal/mol.

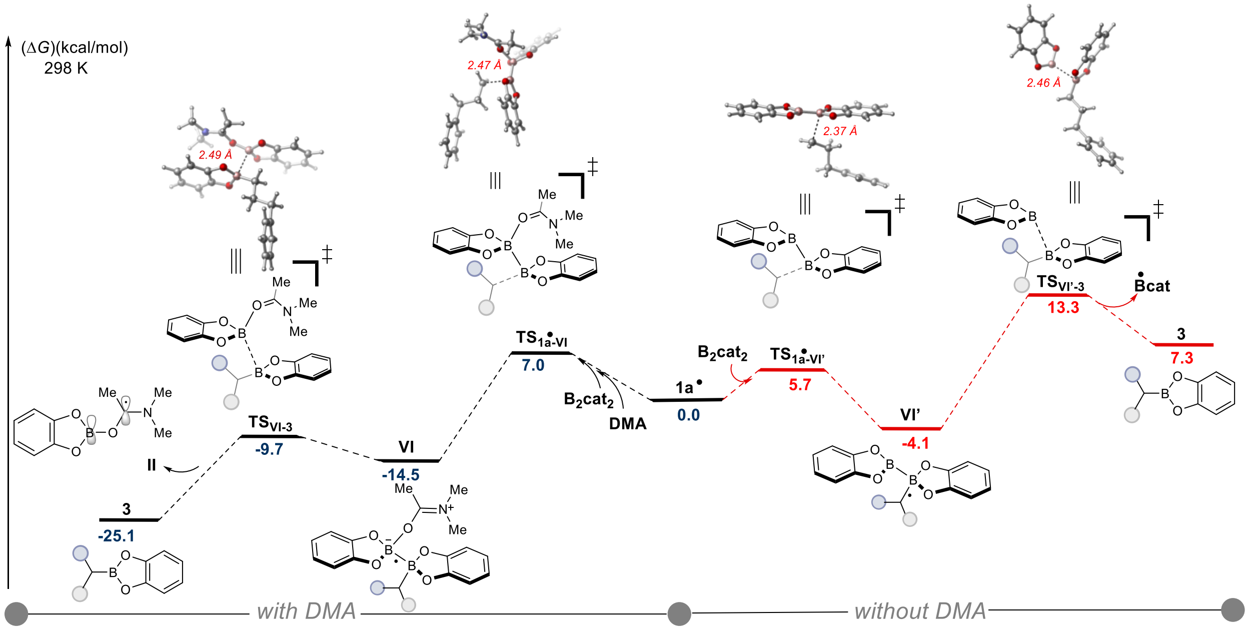

Figure S26: DMA coordination helps to cleave B-B bond and stabilize the formed boryl radical

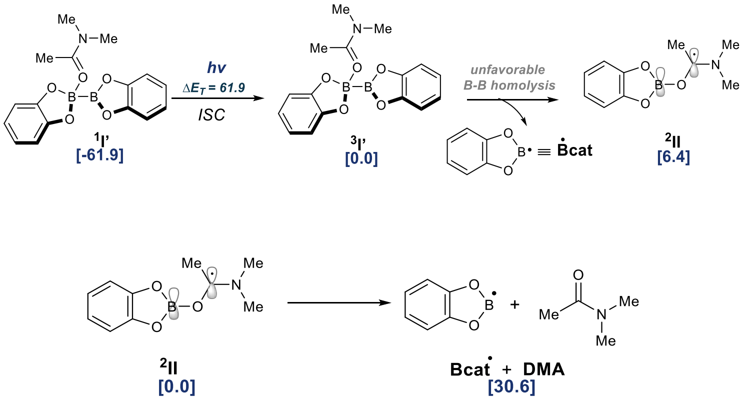

Figure S27: Homolysis required coordination of each DMA molecule at both boron centers to weaken the B-B bond and stabilize the boryl radical; calculated Gibbs free energies [uB3LYP-D3/def2-svp-CPCM(DMA)] are given in kcal/mol.

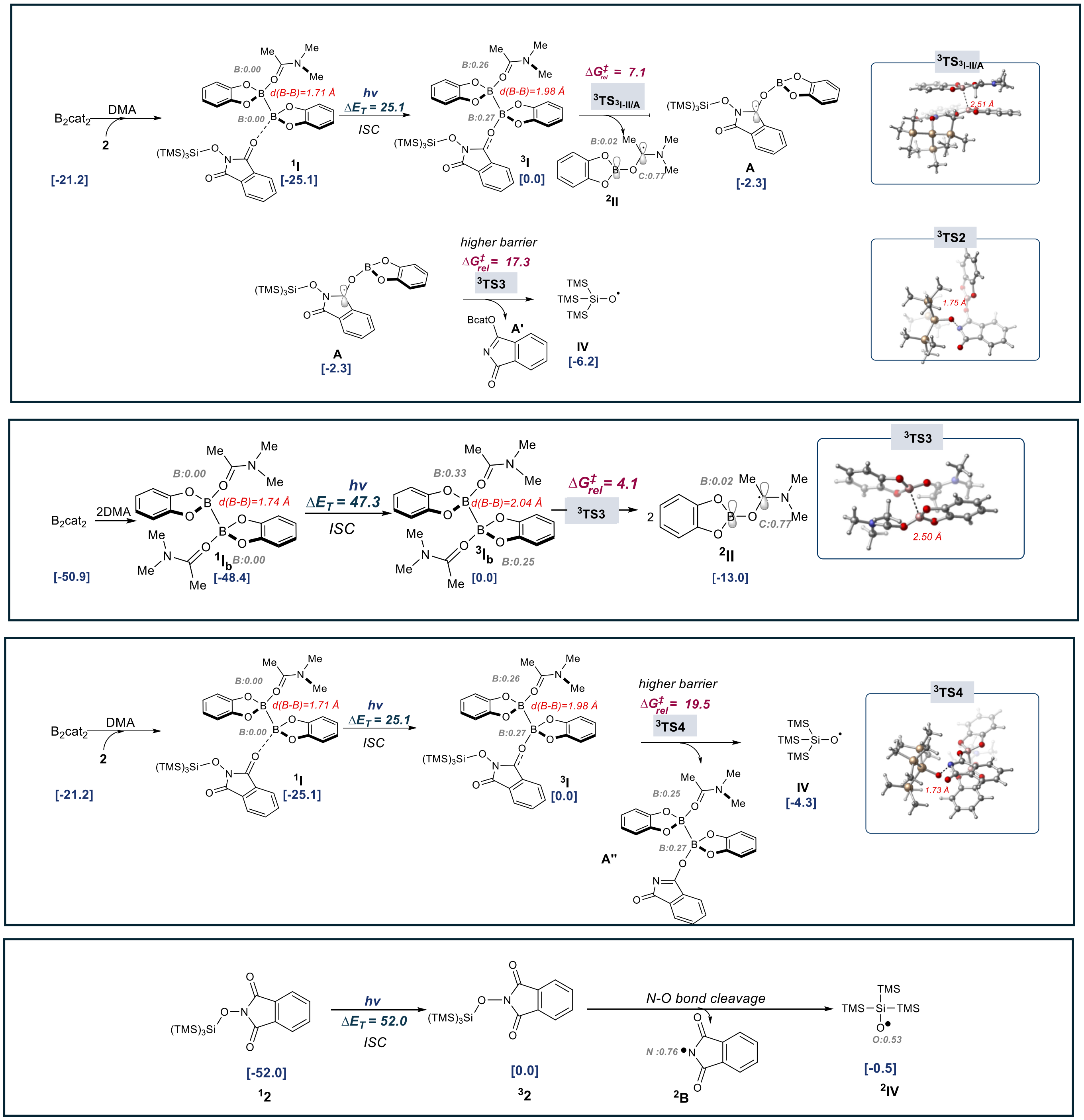

Figure S28: Possible initiation pathways calculated at [uB3LYP-D3/def2-svp-CPCM(DMA)] and Gibbs free energies are given in kcal/mol.

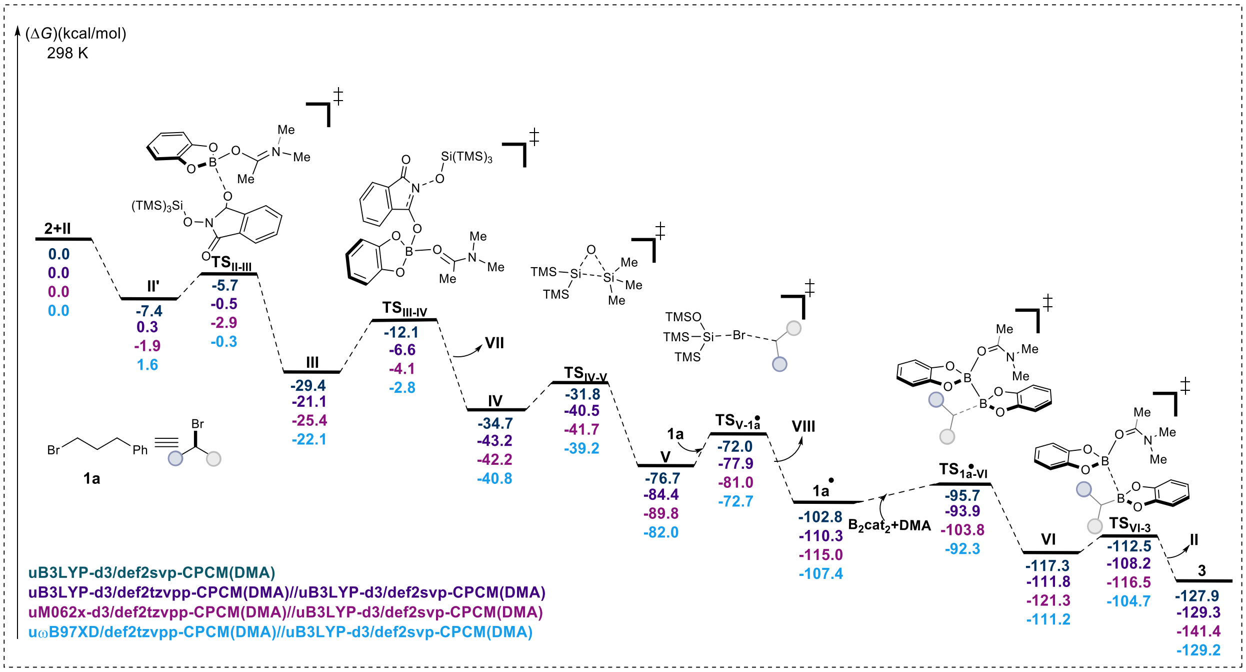

Figure S29: Method comparison on the energetics for the model system. Optimizations were carried out using uB3LYP-D3/def2-svp-CPCM(DMA) level of theory followed by single point energy calculations with chosen functionals (uB3LYP-D3, uM06-2X-D3, uωB97XD) with the def2TZVPP basis set.

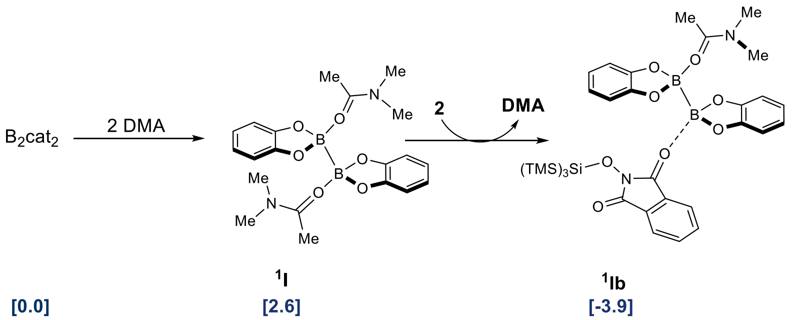

Figure S30: It is plausible for the formation of complex ^{1}I from 2DMA•B2cat2, but it is thermodynamically less favorable. Optimizations were carried out using uB3LYP-D3/def2-svp-CPCM(DMA) level of theory.

Table S5. Cartesian coordinates (xyz format) and energies of all the structures involved in each reaction mechanism studied calculated at the CPCM(DMA)- uB3LYP-d3/def2-svp level of theory.

B_2Cat_2

E(scf) = -812.319594066 a.u.

ν_{min} = 23.3132 cm^{-1}

C	5.716978	-5.604585	0.563644	C	4.318099	-5.607647	0.558377

C	3.580707	-4.527976	0.091611
C	4.314031	-3.426079	-0.377670
C	5.718547	-3.423010	-0.372394
C	6.453131	-4.521700	0.102404
H	2.489717	-4.542671	0.092615
H	3.778132	-2.552262	-0.755166
H	6.253445	-2.546859	-0.745880
H	7.544137	-4.531633	0.111587
C	4.321235	-11.012188	2.878734
C	5.720118	-11.009094	2.884017
C	3.585100	-12.094960	3.340254
C	6.457526	-12.088619	3.351079
C	4.319707	-13.193571	3.815225
H	2.494094	-12.085073	3.331032
C	5.724217	-13.190471	3.820516
H	7.548517	-12.073922	3.350062
H	3.784814	-14.069661	4.188859
H	6.260141	-14.064203	4.198173
O	6.158955	-6.799220	1.078078
O	3.877493	-6.804216	1.069482
O	6.160709	-9.812666	2.372554
O	3.879234	-9.817713	2.363929
B	5.018641	-7.530704	1.386639
B	5.019542	-9.086266	2.055247

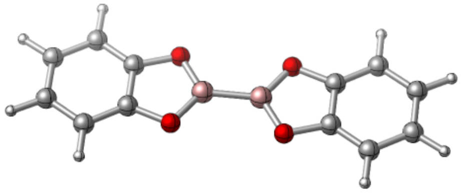

Zero-point correction= 0.187877 (Hartree/Particle)
Thermal correction to Energy= 0.200554
Thermal correction to Enthalpy= 0.201498
Thermal correction to Gibbs Free Energy= 0.146445
Sum of electronic and zero-point Energies= -812.131717
Sum of electronic and thermal Energies= -812.119040
Sum of electronic and thermal Enthalpies= -812.118096
Sum of electronic and thermal Free Energies= -812.173150

UB3LYP-D3/def2TZVPP
E(scf)= -813.2193302
UM062X-D3/def2TZVPP
E(scf)= -812.8933453
uωB97XD/def2TZVPP
E(scf)= -812.911834242

DMA
E(scf) = -287.637078749 a.u.
ν_{min} = 57.3610 cm^{-1}

C	4.631749	-8.827050	-1.562788	H	6.964598	-8.595794	-0.691579
O	4.739675	-8.966654	-0.344884	H	7.721843	-9.194018	-2.203143
N	5.711582	-8.566856	-2.362640	H	7.457214	-7.443141	-1.973884
C	5.665893	-8.386654	-3.803301	C	3.268587	-8.941650	-2.230741
H	4.651687	-8.498243	-4.198190	H	2.988532	-8.011622	-2.749479
H	6.032784	-7.382255	-4.079551	H	3.242834	-9.759063	-2.968078
H	6.311759	-9.128528	-4.305139	H	2.531410	-9.147417	-1.445465
C	7.035268	-8.443623	-1.773822				

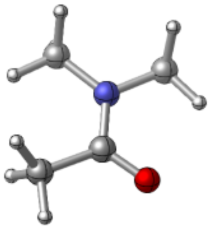

Zero-point correction= 0.129511 (Hartree/Particle)
Thermal correction to Energy= 0.137181
Thermal correction to Enthalpy= 0.138125
Thermal correction to Gibbs Free Energy= 0.097670
Sum of electronic and zero-point Energies= -287.507568
Sum of electronic and thermal Energies= -287.499898
Sum of electronic and thermal Enthalpies= -287.498954
Sum of electronic and thermal Free Energies= -287.539408

UB3LYP-D3/def2TZVPP
E(scf)= -287.9694322
UM062X-D3/def2TZVPP
E(scf)= -287.8205737
uωB97XD/def2TZVPP
E(scf)= -287.863087397

$^{1}I_{b}$
E(scf) = -1387.63425251 a.u.
ν_{min} = 9.5386 cm^{-1}

C	7.649671	-6.281758	-1.303332
C	6.275381	-6.070704	-1.552562
C	5.847763	-5.113800	-2.464370
C	6.831566	-4.354248	-3.133800
C	8.191594	-4.561381	-2.886103
C	8.621348	-5.536402	-1.960056
H	4.781995	-4.961573	-2.650768
H	6.520992	-3.593773	-3.855156
H	8.936827	-3.961160	-3.414811
H	9.681699	-5.707901	-1.760267
C	6.312604	-10.734131	2.228137
C	7.204554	-11.157542	1.218583
C	6.176912	-11.440364	3.416451
C	7.973367	-12.304365	1.378891
C	6.955258	-12.606542	3.582128
H	5.488742	-11.094418	4.191354
C	7.834852	-13.030118	2.581753
H	8.663195	-12.624042	0.594330
H	6.866537	-13.182556	4.506941
H	8.428476	-13.936073	2.730041
O	7.813523	-7.276181	-0.402422
O	5.531561	-6.924621	-0.814224
O	7.189871	-10.290809	0.182966
O	5.707724	-9.583554	1.859782
B	6.463070	-7.626519	0.101157
C	3.764234	-9.288502	-0.595033
O	4.928142	-9.793610	-0.487517
N	3.066539	-9.622248	-1.677827
C	3.690185	-10.509396	-2.664313
H	3.991143	-11.456158	-2.193769
H	2.968382	-10.712439	-3.463575
H	4.586897	-10.036918	-3.091965
C	1.721045	-9.141807	-1.976681
H	1.363849	-8.447061	-1.211652
H	1.724168	-8.618927	-2.945403
H	1.022877	-9.991123	-2.037786
B	6.122517	-9.300926	0.449453
C	3.182914	-8.377659	0.450530
H	3.060013	-7.369599	0.031192
H	2.205368	-8.748129	0.790833
H	3.860058	-8.324296	1.305879
C	6.770609	-7.122937	2.610910
O	6.265353	-6.811381	1.486283
N	6.143936	-6.746213	3.725579
C	6.658912	-7.081167	5.053136
H	6.462041	-8.137914	5.296727

H	7.737241	-6.892128	5.125624	H	4.859379	-5.147361	4.209378
H	6.153246	-6.450689	5.794668	C	8.074445	-7.863042	2.716790
C	4.827436	-6.112292	3.680839	H	8.839691	-7.212243	3.168756
H	4.527380	-5.948776	2.641919	H	7.966852	-8.754485	3.350258
H	4.088643	-6.762430	4.175835	H	8.414568	-8.158190	1.720720

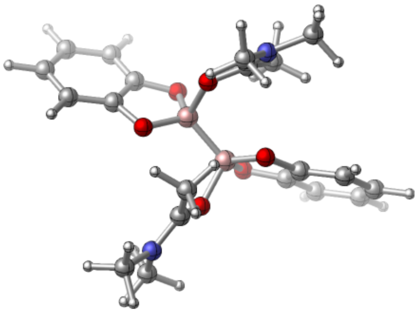

Zero-point correction= 0.450638 (Hartree/Particle)
Thermal correction to Energy= 0.480792
Thermal correction to Enthalpy= 0.481737
Thermal correction to Gibbs Free Energy= 0.386357
Sum of electronic and zero-point Energies= -1387.183615
Sum of electronic and thermal Energies= -1387.153460
Sum of electronic and thermal Enthalpies= -1387.152516
Sum of electronic and thermal Free Energies= -1387.247896

UB3LYP-D3/def2TZVPP
E(scf)= -1389.176859
UM062X-D3/def2TZVPP
E(scf)= -1388.56871
uωB97XD/def2TZVPP
E(scf)= -1388.66012925

$^{3}I_{b}$

E(scf) = -1387.55884719 a.u.

ν_{min} = 18.1931 cm^{-1}

C	6.842370	-6.563083	-1.396894	C	6.905289	-10.634001	1.868835
C	5.505523	-6.174585	-1.171519	C	5.367794	-10.964934	3.734310
C	4.733995	-5.606302	-2.177712	C	7.864895	-11.463146	2.433074
C	5.341460	-5.420674	-3.434696	C	6.335779	-11.809371	4.312201
C	6.672838	-5.795149	-3.657408	H	4.412342	-10.765919	4.222784
C	7.447517	-6.377252	-2.635981	C	7.559704	-12.051297	3.676171
H	3.697060	-5.318341	-1.993557	H	8.816231	-11.641540	1.928333
H	4.761346	-4.976967	-4.247276	H	6.125407	-12.281038	5.275143
H	7.118897	-5.640212	-4.642707	H	8.292785	-12.709107	4.149452
H	8.482859	-6.681585	-2.801010	O	7.363584	-7.105930	-0.276493
C	5.679263	-10.383713	2.513265	O	5.144353	-6.473590	0.100519

O	6.959705	-9.954457	0.695116	H	1.940731	-9.095823	-0.465697
O	4.932349	-9.530810	1.762823	H	3.129653	-8.735540	0.817978
B	6.272107	-7.179532	0.705653	C	7.254260	-7.612748	2.916427
C	3.970718	-8.901706	-1.135721	O	6.524486	-6.827799	2.037320
O	5.127196	-9.272788	-0.698640	N	6.900173	-7.349781	4.253151
N	3.837814	-8.871622	-2.449213	C	7.677081	-8.008872	5.288714
C	4.980425	-9.229499	-3.300099	H	7.542643	-9.111281	5.288846
H	5.426314	-10.172324	-2.959726	H	8.749019	-7.792479	5.182322
H	4.625101	-9.341756	-4.330272	H	7.360240	-7.630367	6.272329
H	5.740768	-8.436579	-3.258140	C	5.476084	-7.292436	4.555830
C	2.636603	-8.407678	-3.139904	H	4.947022	-6.736408	3.773352
H	1.866118	-8.082229	-2.436455	H	5.021990	-8.300733	4.630836
H	2.907255	-7.556148	-3.781865	H	5.324458	-6.774545	5.516095
H	2.234057	-9.217024	-3.766859	C	8.696969	-7.828317	2.569392
B	5.757538	-9.161318	0.632784	H	9.323876	-6.932587	2.777976
C	2.848027	-8.527504	-0.216652	H	9.126194	-8.679134	3.117171
H	2.630765	-7.454296	-0.317319	H	8.778113	-8.050719	1.498048

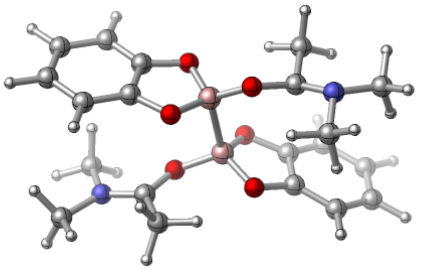

Zero-point correction= 0.449683 (Hartree/Particle)
Thermal correction to Energy= 0.479360
Thermal correction to Enthalpy= 0.480304
Thermal correction to Gibbs Free Energy= 0.388032
Sum of electronic and zero-point Energies= -1387.109164
Sum of electronic and thermal Energies= -1387.079487
Sum of electronic and thermal Enthalpies= -1387.078543
Sum of electronic and thermal Free Energies= -1387.170815

UB3LYP-D3/def2TZVPP
E(scf)= -1389.100171
UM062X-D3/def2TZVPP
E(scf)= -1388.487657
uωB97XD/def2TZVPP
E(scf)= -1388.58064883

^{3}TS3
E(scf) = -1387.54936777 a.u.
ν_{min} = -596.3685 cm^{-1}

C	6.073008	-6.668956	-1.346173	C	5.977790	-9.673042	-3.163210
C	4.842388	-6.375187	-0.735133	H	6.694444	-9.919711	-2.371637
C	3.878916	-5.608614	-1.373879	H	5.832299	-10.571025	-3.800364
C	4.193006	-5.120389	-2.658099	H	6.411858	-8.874238	-3.782419
C	5.423851	-5.403315	-3.260226	C	3.796521	-8.644489	-3.538373
C	6.393161	-6.190359	-2.606933	H	3.245145	-9.423264	-4.107053
H	2.922576	-5.394479	-0.893145	H	3.070484	-7.988197	-3.041206
H	3.458213	-4.512367	-3.191313	H	4.351608	-8.023614	-4.254577
H	5.639475	-5.011787	-4.257339	B	5.335382	-10.050776	0.755056
H	7.353791	-6.424184	-3.069151	C	2.879143	-9.540551	-0.921431
C	4.752764	-10.326247	2.882342	H	2.106338	-9.545382	-1.702141
C	6.086944	-10.738328	2.732824	H	2.592879	-10.274786	-0.156922
C	4.112388	-10.364049	4.113464	H	2.873939	-8.544037	-0.433419
C	6.832159	-11.200519	3.807685	C	7.757225	-7.987450	2.432375
C	4.858782	-10.836722	5.209560	O	6.582547	-7.675551	1.934665
H	3.074633	-10.042186	4.216699	N	7.814563	-8.115063	3.776515
C	6.190492	-11.246031	5.059972	C	9.000589	-8.611858	4.456523
H	7.869926	-11.512269	3.680157	H	9.545686	-9.334638	3.839076
H	4.388895	-10.881385	6.194835	H	9.683087	-7.788920	4.736201
H	6.745836	-11.604338	5.929860	H	8.686901	-9.129347	5.374231
O	6.822714	-7.449607	-0.514128	C	6.748948	-7.636225	4.647166
O	4.788555	-6.956944	0.498060	H	7.159390	-6.913817	5.372826
O	6.467365	-10.589190	1.430013	H	5.964265	-7.148217	4.061410
O	4.257942	-9.930042	1.674531	H	6.306810	-8.480182	5.199554
B	6.024712	-7.653412	0.652457	C	8.900878	-8.371358	1.553285
C	4.221571	-9.894562	-1.479628	H	9.859396	-8.016865	1.956254
O	5.218617	-10.255832	-0.589239	H	8.952173	-9.471609	1.450722
N	4.732459	-9.201373	-2.581325	H	8.759868	-7.950300	0.552859

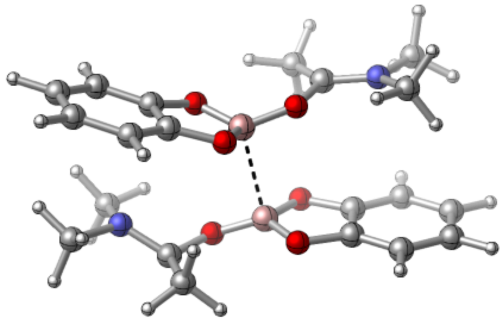

Zero-point correction= 0.447477 (Hartree/Particle)
Thermal correction to Energy= 0.477353
Thermal correction to Enthalpy= 0.478297
Thermal correction to Gibbs Free Energy= 0.385053
Sum of electronic and zero-point Energies= -1387.101891
Sum of electronic and thermal Energies= -1387.072015

Sum of electronic and thermal Enthalpies= -1387.071071
Sum of electronic and thermal Free Energies= -1387.164315

UB3LYP-D3/def2TZVPP
E(scf)= -1389.08913
UM062X-D3/def2TZVPP
E(scf)= -1388.471364
uωB97XD/def2TZVPP
E(scf)= -1388.56356068

II_complex
E(scf) = -1387.58016677 a.u.
ν_{min} = 18.9956 cm^{-1}

C	5.858496	-5.973745	-1.422467	C	6.656568	-9.456734	-2.431331
C	4.560344	-6.164263	-0.929563	H	7.237169	-9.801048	-1.569540
C	3.451857	-5.624757	-1.562256	H	6.710166	-10.231966	-3.223376
C	3.690313	-4.879215	-2.731674	H	7.117121	-8.533692	-2.812755
C	4.987834	-4.694201	-3.227473	C	4.485047	-8.640753	-3.133604
C	6.106871	-5.243046	-2.573068	H	4.257686	-9.409430	-3.901095
H	2.445399	-5.784516	-1.172135	H	3.543097	-8.215059	-2.769127
H	2.843713	-4.439743	-3.264100	H	5.044006	-7.825618	-3.613395
H	5.137450	-4.112695	-4.140028	B	5.356785	-10.572917	1.139205
H	7.122249	-5.109028	-2.949618	C	3.200408	-9.964772	-0.862634
C	4.620948	-10.353184	3.199469	H	2.577209	-9.969983	-1.767741
C	5.869611	-10.988346	3.237936	H	2.881788	-10.807364	-0.233586
C	3.917184	-10.049364	4.354224	H	2.991532	-9.031499	-0.302525
C	6.464813	-11.357585	4.433223	C	7.737483	-8.137296	1.809023
C	4.515107	-10.413125	5.575293	O	6.397170	-7.994954	1.394585
H	2.947839	-9.550012	4.309709	N	7.786364	-8.506870	3.149587
C	5.760684	-11.054647	5.613222	C	9.003615	-9.086072	3.680529
H	7.439302	-11.847489	4.450386	H	9.343712	-9.928655	3.063563
H	3.995764	-10.189541	6.510008	H	9.832193	-8.352047	3.761688
H	6.198304	-11.323388	6.577475	H	8.799027	-9.476380	4.687888
O	6.751102	-6.622552	-0.602805	C	7.018622	-7.724775	4.106487
O	4.614610	-6.941723	0.199418	H	7.572050	-6.826851	4.450267
O	6.332578	-11.148299	1.956819	H	6.073978	-7.400214	3.654562
O	4.276420	-10.112946	1.891584	H	6.779775	-8.349001	4.980675
B	5.966796	-7.237732	0.371750	C	8.641577	-8.797848	0.814860
C	4.651003	-10.112080	-1.200319	H	9.698456	-8.679883	1.092459
O	5.504960	-10.556407	-0.196210	H	8.427154	-9.881769	0.717454
N	5.280628	-9.174549	-2.036969	H	8.503358	-8.335557	-0.171235

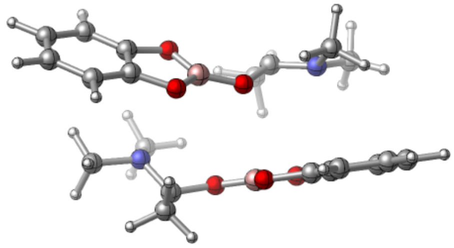

Zero-point correction= 0.450390 (Hartree/Particle)
Thermal correction to Energy= 0.479950
Thermal correction to Enthalpy= 0.480894
Thermal correction to Gibbs Free Energy= 0.388560
Sum of electronic and zero-point Energies= -1387.129777
Sum of electronic and thermal Energies= -1387.100217
Sum of electronic and thermal Enthalpies= -1387.099272
Sum of electronic and thermal Free Energies= -1387.191607

UB3LYP-D3/def2TZVPP
E(scf)= -1389.121563
UM062X-D3/def2TZVPP
E(scf)= -1388.512015
uωB97XD/def2TZVPP
E(scf)= -1388.60203454

II

E(scf) = -693.776354317 a.u.

ν_{min} = 26.3165 cm^{-1}

C	6.331315	-5.740340	-1.688121	O	6.886893	-6.792997	1.613178
C	5.063571	-6.193550	-1.297494	N	8.293540	-6.330035	3.385570
C	3.974601	-6.150063	-2.154321	C	9.573881	-6.467434	4.057439
C	4.202921	-5.627890	-3.440905	H	9.423688	-6.346012	5.140342
C	5.470647	-5.175350	-3.831171	H	10.004777	-7.463938	3.891455
C	6.568620	-5.224354	-2.952354	H	10.314601	-5.707140	3.732329
H	2.992209	-6.505352	-1.838874	C	7.493709	-5.205967	3.857239
H	3.372078	-5.575388	-4.148266	H	6.509958	-5.212420	3.371539
H	5.612743	-4.775764	-4.837896	H	7.342975	-5.287699	4.944523
H	7.560714	-4.875386	-3.243568	H	7.980508	-4.231731	3.645328
O	7.209443	-5.903413	-0.641774	C	9.181718	-7.594683	1.404969
O	5.126796	-6.648726	-0.002026	H	10.227213	-7.292387	1.558396
B	6.452278	-6.461070	0.387180	H	9.057942	-8.621849	1.809588
C	8.225971	-6.621500	2.023154	H	9.006357	-7.639647	0.320460

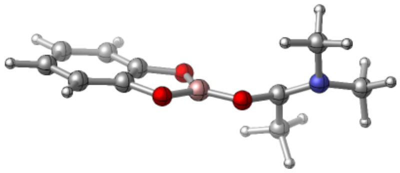

Zero-point correction= 0.224072 (Hartree/Particle)
Thermal correction to Energy= 0.238207
Thermal correction to Enthalpy= 0.239151
Thermal correction to Gibbs Free Energy= 0.181276
Sum of electronic and zero-point Energies= -693.552282
Sum of electronic and thermal Energies= -693.538148
Sum of electronic and thermal Enthalpies= -693.537204
Sum of electronic and thermal Free Energies= -693.595078

UB3LYP-D3/def2TZVPP
E(scf)= -694.5524038
UM062X-D3/def2TZVPP
E(scf)= -694.2463356
uωB97XD/def2TZVPP
E(scf)= -694.291926609

2
E(scf) = -2104.09568408 a.u.
ν_{min} = 20.1146 cm^{-1}

C	3.563028	-10.628734	2.406928	C	2.730558	-7.112372	-2.467984
C	4.925898	-10.796542	2.692921	H	2.618452	-7.064541	-1.373339
C	2.584263	-11.309863	3.116295	H	2.283072	-6.206249	-2.910561
C	5.355812	-11.649917	3.698702	H	2.163188	-7.987586	-2.822774
C	3.009675	-12.175473	4.139542	C	5.455102	-5.638550	-2.547116
H	1.525757	-11.177021	2.884234	H	6.518197	-5.692395	-2.833120
C	4.371281	-12.342288	4.425803	H	4.994902	-4.797651	-3.093743
H	6.418770	-11.777682	3.912510	H	5.403779	-5.416884	-1.468560
H	2.267609	-12.728171	4.720050	C	4.730974	-7.666197	-4.783909
H	4.672024	-13.022679	5.225633	H	4.181348	-8.587049	-5.038650
N	4.752502	-9.342276	0.935180	H	4.321200	-6.844454	-5.395743
C	5.716111	-9.951625	1.750066	H	5.786342	-7.809188	-5.066073
O	6.914143	-9.802941	1.650358	C	8.517533	-7.638845	-0.608215
C	3.430472	-9.672796	1.268145	H	9.620592	-7.609436	-0.608843
O	2.440563	-9.261515	0.705269	H	8.144559	-6.654575	-0.934784
O	5.053689	-8.426272	-0.014421	H	8.168937	-7.821826	0.419803
Si	5.510917	-8.990891	-1.643653	C	8.590797	-10.684796	-1.173718
Si	4.586285	-11.126049	-2.094767	H	8.294436	-10.893529	-0.134455
Si	7.878375	-9.020794	-1.732416	H	8.236260	-11.510172	-1.812085
Si	4.558556	-7.261609	-2.941200	H	9.693029	-10.663859	-1.225890

C	8.392533	-8.696326	-3.531363	C	5.393642	-11.674710	-3.722091
H	8.050738	-7.704728	-3.870173	H	6.486506	-11.770895	-3.617459
H	9.491357	-8.729038	-3.627757	H	4.993530	-12.656363	-4.028421
H	7.969207	-9.452953	-4.212331	H	5.191251	-10.955880	-4.532670
C	5.019436	-12.375552	-0.733944	C	2.707286	-11.051481	-2.310691
H	4.428856	-12.207961	0.179868	H	2.439436	-10.431272	-3.181401
H	4.800989	-13.397654	-1.087865	H	2.300957	-12.064466	-2.473370
H	6.086484	-12.326563	-0.465670	H	2.225938	-10.619238	-1.419687

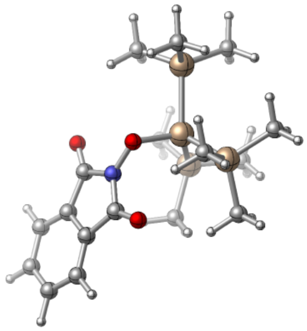

Zero-point correction= 0.443700 (Hartree/Particle)
Thermal correction to Energy= 0.479133
Thermal correction to Enthalpy= 0.480077
Thermal correction to Gibbs Free Energy= 0.376978
Sum of electronic and zero-point Energies= -2103.651984
Sum of electronic and thermal Energies= -2103.616551
Sum of electronic and thermal Enthalpies= -2103.615607
Sum of electronic and thermal Free Energies= -2103.718706

UB3LYP-D3/def2TZVPP
E(scf)= -2105.57963114
UM062X-D3/def2TZVPP
E(scf)= -2104.93653679
uωB97XD/def2TZVPP
E(scf)= -2105.13586042

II’
E(scf) = -2797.91532267 a.u.
ν_{min} = 24.2131 cm^{-1}

C	3.929618	-2.703347	-2.822956	H	5.444168	-5.283625	-1.151755
C	4.082206	-3.885714	-2.090494	H	7.442842	-3.946064	-1.877274
C	5.330959	-4.362962	-1.723955	H	7.169084	-1.867174	-3.194441
C	6.438909	-3.603485	-2.137555	H	4.873486	-1.040237	-3.829219
C	6.283797	-2.426195	-2.883135	O	2.584636	-2.465056	-3.015466
C	5.011688	-1.948659	-3.242116	O	2.835511	-4.419373	-1.829809

B	1.954102	-3.550952	-2.444115
O	0.592277	-3.707688	-2.268735
C	-0.415154	-2.923829	-2.617226
N	-1.617789	-3.411155	-2.412826
C	-0.154413	-1.512123	-3.006396
H	-1.068214	-0.964561	-3.246173
H	0.355203	-1.017574	-2.163681
H	0.526168	-1.472212	-3.866969
C	-1.819478	-4.762124	-1.882458
H	-2.762698	-4.777728	-1.323018
H	-1.871157	-5.485590	-2.709443
H	-0.998801	-5.037476	-1.214438
C	-2.798754	-2.717380	-2.944503
H	-3.005711	-1.795313	-2.383172
H	-2.636818	-2.495323	-4.008035
H	-3.661177	-3.385274	-2.849330
C	2.086689	-1.897255	-6.857918
C	0.696487	-2.002034	-6.519688
C	2.542170	-0.859939	-7.685884
C	-0.225845	-1.062260	-7.019041
C	1.620292	0.064591	-8.171411
H	3.603540	-0.785764	-7.938655
C	0.240308	-0.038463	-7.836583
H	-1.285768	-1.140814	-6.762520
H	1.955213	0.880388	-8.817329
H	-0.460905	0.701902	-8.231125
N	1.799934	-3.706384	-5.556906
C	0.509516	-3.149660	-5.665050
O	-0.497916	-3.592377	-5.069885
C	2.813599	-2.974360	-6.206398
O	4.016127	-3.267855	-6.184095
O	2.079917	-4.707914	-4.672511
Si	2.260967	-6.353278	-5.325313
Si	2.133157	-6.338948	-7.696441
Si	0.484002	-7.572980	-4.349037
Si	4.357077	-7.184814	-4.605234
C	5.748499	-5.958066	-4.972940
H	5.489604	-4.942112	-4.645203
H	6.677940	-6.272324	-4.468358
H	5.935107	-5.919726	-6.057954
C	4.289729	-7.615210	-2.757232
H	3.699686	-8.534014	-2.606445
H	5.304765	-7.792940	-2.363108
H	3.820992	-6.813302	-2.168755
C	4.681234	-8.790695	-5.56959
H	4.785599	-8.59517	-6.649094
H	5.616569	-9.25964	-5.218315
H	3.864901	-9.518107	-5.431862
C	0.546774	-7.33967	-2.467276
H	-0.381015	-7.706195	-1.996102
H	1.39363	-7.900791	-2.042949
H	0.679155	-6.281948	-2.200852
C	-1.164404	-6.945511	-5.039182
H	-1.201501	-5.845964	-4.977999
H	-1.270753	-7.229242	-6.099504
H	-2.016232	-7.374063	-4.483404
C	0.681477	-9.423616	-4.72237
H	1.61927	-9.815315	-4.295614
H	-0.154803	-9.988661	-4.275626
H	0.689579	-9.624895	-5.804863
C	0.733215	-5.24407	-8.352265
H	0.985773	-4.176868	-8.265061
H	0.55493	-5.471792	-9.417408
H	-0.203726	-5.416749	-7.799686
C	1.777017	-8.134169	-8.206589
H	0.763395	-8.438792	-7.900018
H	1.841461	-8.22728	-9.304386
H	2.496016	-8.840619	-7.762083
C	3.781173	-5.792953	-8.452368
H	4.57874	-6.513299	-8.206598
H	3.700192	-5.734272	-9.551421
H	4.078218	-4.80916	-8.057769

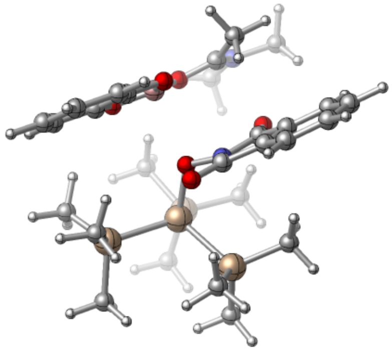

Zero-point correction= 0.671955 (Hartree/Particle)
Thermal correction to Energy= 0.721910
Thermal correction to Enthalpy= 0.722854
Thermal correction to Gibbs Free Energy= 0.589818
Sum of electronic and zero-point Energies= -2797.243368
Sum of electronic and thermal Energies= -2797.193413
Sum of electronic and thermal Enthalpies= -2797.192469
Sum of electronic and thermal Free Energies= -2797.325505

UB3LYP-D3/def2TZVPP
E(scf)= -2800.163048
UM062X-D3/def2TZVPP
E(scf)= -2799.217401
uωB97XD/def2TZVPP
E(scf)= -2799.45675803

$TS_{II\text{-}III}$
E(scf) = -2797.91093162 a.u.
ν_{min} = -62.7435 cm^{-1}

C	3.454664	-2.367654	-2.718625
C	3.615454	-3.604285	-2.084167
C	4.855645	-4.205967	-1.956863
C	5.946599	-3.522257	-2.522426
C	5.780239	-2.295006	-3.179825
C	4.517051	-1.685892	-3.286728
H	4.972495	-5.167050	-1.455430
H	6.942666	-3.965103	-2.455504
H	6.648528	-1.798127	-3.618180
H	4.370367	-0.734161	-3.799075
O	2.120497	-2.015840	-2.681974
O	2.381947	-4.056162	-1.663432
B	1.490465	-3.096806	-2.094763
O	0.159975	-3.167565	-1.702101
C	-0.847455	-2.559258	-2.297140
N	-1.964988	-3.224273	-2.396689
C	-0.679890	-1.146617	-2.732921
H	-1.618255	-0.661310	-3.011708
H	-0.201345	-0.589139	-1.915282
H	0.011105	-1.131161	-3.587802
C	-2.085731	-4.626853	-1.977019
H	-2.965344	-4.723749	-1.326531
H	-2.219672	-5.249675	-2.872676
H	-1.190110	-4.948447	-1.442637
C	-3.154603	-2.641021	-3.041188
H	-3.513419	-1.768498	-2.479691
H	-2.925078	-2.356310	-4.076583
H	-3.943027	-3.400267	-3.052714

C	2.326050	-1.986246	-6.816873	C	4.725585	-9.228778	-5.818275
C	1.097428	-2.144961	-6.085208	H	4.820202	-8.993426	-6.890706
C	2.465472	-0.953096	-7.757198	H	5.615661	-9.806643	-5.515832
C	0.019283	-1.269992	-6.325575	H	3.840887	-9.873175	-5.688045
C	1.395909	-0.087817	-7.972155	C	0.743664	-6.975198	-2.489599
H	3.402177	-0.840195	-8.310353	H	-0.152809	-7.395896	-2.002740
C	0.174194	-0.250665	-7.259019	H	1.634349	-7.424252	-2.022179
H	-0.928561	-1.399690	-5.797460	H	0.774542	-5.893450	-2.305034
H	1.487600	0.722041	-8.700697	C	-0.896857	-6.651717	-5.100667
H	-0.655413	0.433688	-7.455884	H	-0.916486	-5.556464	-4.992413
N	2.512565	-3.778283	-5.481867	H	-0.974095	-6.902921	-6.171059
C	1.237363	-3.272390	-5.200273	H	-1.773155	-7.083527	-4.586574
O	0.497889	-3.739417	-4.293091	C	0.769473	-9.207978	-4.605551
C	3.252779	-3.031909	-6.422502	H	1.668105	-9.643803	-4.138627
O	4.408293	-3.301154	-6.787137	H	-0.114330	-9.681943	-4.144427
O	3.038853	-4.843878	-4.817510	H	0.779895	-9.476747	-5.673734
Si	2.639106	-6.455220	-5.409629	C	1.064894	-5.182957	-8.326449
Si	2.362244	-6.446229	-7.765536	H	1.414068	-4.150270	-8.176688
Si	0.707548	-7.322668	-4.349810	H	0.849609	-5.322206	-9.400143
Si	4.580778	-7.647711	-4.779728	H	0.123185	-5.306361	-7.768461
C	6.064814	-6.515748	-5.100781	C	1.744141	-8.180936	-8.235168
H	5.939574	-5.563576	-4.562273	H	0.736008	-8.366481	-7.830645
H	7.006050	-6.987768	-4.771266	H	1.689609	-8.272460	-9.333648
H	6.147702	-6.283369	-6.174826	H	2.414016	-8.971038	-7.859983
C	4.514851	-8.125584	-2.944968	C	4.006283	-6.099626	-8.636781
H	3.702143	-8.845499	-2.755356	H	4.754333	-6.871106	-8.390562
H	5.464367	-8.590705	-2.629251	H	3.871449	-6.089097	-9.732161
H	4.336553	-7.242354	-2.312024	H	4.394959	-5.121968	-8.311370

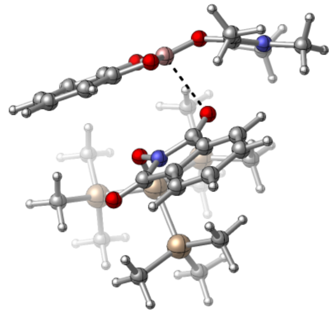

Zero-point correction= 0.671020 (Hartree/Particle)
Thermal correction to Energy= 0.720743
Thermal correction to Enthalpy= 0.721687
Thermal correction to Gibbs Free Energy= 0.588086
Sum of electronic and zero-point Energies= -2797.239911
Sum of electronic and thermal Energies= -2797.190189
Sum of electronic and thermal Enthalpies= -2797.189244

Sum of electronic and thermal Free Energies= -2797.322846
UB3LYP-D3/def2TZVPP
E(scf)= -2800.162593
UM062X-D3/def2TZVPP
E(scf)= -2799.217247
uωB97XD/def2TZVPP
E(scf)= -2799.45808715

III

E(scf) = -2797.94586913 a.u.

ν_{min} = 15.1597 cm^{-1}

C	5.625825	-11.552768	2.801826	C	4.990751	-6.185885	-2.914048
C	7.001106	-11.305027	3.112446	H	5.680157	-5.371300	-2.638631
C	4.819881	-12.309095	3.676723	H	4.762120	-6.090951	-3.989293
C	7.569599	-11.813225	4.284526	H	4.053410	-6.041633	-2.353053
C	5.402296	-12.806957	4.837910	C	7.310121	-8.108843	-3.617674
H	3.774735	-12.505157	3.430136	H	7.045987	-8.119959	-4.689091
C	6.765206	-12.564230	5.143698	H	8.029693	-7.290958	-3.451340
H	8.620232	-11.620772	4.515747	H	7.813933	-9.059683	-3.379021
H	4.799506	-13.400206	5.530312	C	4.523036	-9.260275	-2.976513
H	7.188115	-12.972358	6.064836	H	5.027937	-10.237750	-2.959563
N	6.506901	-10.274262	1.167429	H	3.683414	-9.307904	-2.266686
C	7.590224	-10.490784	2.048583	H	4.118780	-9.106073	-3.991687
O	8.729554	-10.053460	1.880769	C	8.202233	-5.090699	-0.565001
C	5.347310	-10.901635	1.570708	H	8.105915	-5.174149	-1.660046
O	6.634217	-9.695989	-0.051622	H	7.338702	-4.517693	-0.190993
C	1.735729	-12.849990	0.550467	H	9.116156	-4.511322	-0.348042
C	1.396003	-11.792130	-0.316021	C	8.536707	-6.623204	2.113831
C	0.765980	-13.697795	1.067673	H	8.623946	-7.621711	2.570107
C	0.081462	-11.555814	-0.690584	H	9.456610	-6.055262	2.336208
C	-0.573567	-13.460906	0.695432	H	7.687831	-6.095263	2.577812
H	1.040530	-14.516282	1.736423	C	9.753514	-7.781886	-0.485373
C	-0.909360	-12.411685	-0.166048	H	9.764422	-8.783269	-0.028889
H	-0.166599	-10.734225	-1.365814	H	9.657455	-7.888813	-1.577695
H	-1.359241	-14.110656	1.089062	H	10.712466	-7.280509	-0.268395
H	-1.954753	-12.248803	-0.439776	C	4.641791	-5.321571	1.072869
O	4.219027	-10.716995	0.921390	H	4.646260	-4.917559	0.047377
B	3.628306	-11.719138	0.034970	H	3.786173	-4.876431	1.609289
O	3.079575	-12.881887	0.744783	H	5.565509	-4.990074	1.574057
O	2.516178	-11.121421	-0.695399	C	2.812067	-7.693051	0.339860
Si	6.300443	-7.964371	-0.255895	H	2.673217	-7.227293	-0.649469
Si	4.487304	-7.216545	1.078708	H	2.732303	-8.783557	0.221993
Si	5.755631	-7.884130	-2.555632	H	1.996090	-7.344184	0.996272
Si	8.304245	-6.806824	0.242882	C	4.615149	-7.822687	2.870654

H	3.910692	-7.254378	3.502158	H	7.120543	-14.191973	-3.753432
H	4.361251	-8.889821	2.954617	C	7.088609	-12.197136	-2.176062
H	5.630650	-7.676853	3.272515	H	7.803438	-12.866471	-1.672465
C	4.766018	-12.866349	-1.918078	H	7.562554	-11.789271	-3.079835
O	4.773420	-12.134029	-0.862219	H	6.815531	-11.376607	-1.504976
N	5.902611	-12.963402	-2.585940	C	3.500388	-13.535476	-2.348491
C	6.095017	-13.800943	-3.773520	H	2.765020	-12.743636	-2.551261
H	5.408494	-14.652292	-3.786881	H	3.602811	-14.163629	-3.235340
H	5.961604	-13.201728	-4.687275	H	3.116368	-14.130751	-1.508565

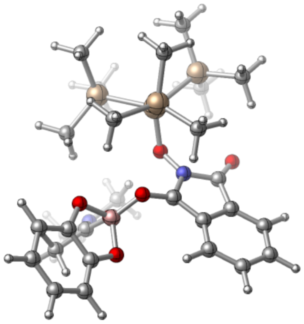

Zero-point correction= 0.671041 (Hartree/Particle)
Thermal correction to Energy= 0.721379
Thermal correction to Enthalpy= 0.722323
Thermal correction to Gibbs Free Energy= 0.585233
Sum of electronic and zero-point Energies= -2797.274829
Sum of electronic and thermal Energies= -2797.224490
Sum of electronic and thermal Enthalpies= -2797.223546
Sum of electronic and thermal Free Energies= -2797.360636

UB3LYP-D3/def2TZVPP
E(scf)= -2800.19257
UM062X-D3/def2TZVPP
E(scf)= -2799.217401
uωB97XD/def2TZVPP
E(scf)= -2799.49002234

TS_{III-IV}
E(scf) = -2797.91499203 a.u.
ν_{min} = -770.6524 cm^{-1}

C	6.000585	-12.052199	2.506299	C	7.758298	-13.864375	3.700447
C	7.340902	-11.696733	2.782608	H	9.264573	-12.286987	3.571356
C	5.519983	-13.321418	2.851246	H	6.077289	-15.219944	3.709670
C	8.232238	-12.582624	3.371184	H	8.429519	-14.590968	4.163292
C	6.421891	-14.217438	3.443242	N	6.234396	-9.845542	1.831643
H	4.485716	-13.602502	2.649465	C	7.519591	-10.294649	2.329647

O	8.512688	-9.608341	2.342073
C	5.369230	-10.882827	1.917901
O	6.514220	-9.505352	0.180345
C	1.431246	-12.623294	0.901478
C	1.280969	-11.541744	0.011528
C	0.341464	-13.382728	1.304233
C	0.040338	-11.190618	-0.500054
C	-0.924052	-13.029833	0.791336
H	0.469108	-14.220842	1.992534
C	-1.071870	-11.956186	-0.092673
H	-0.060602	-10.351357	-1.191279
H	-1.801181	-13.608391	1.091763
H	-2.063312	-11.703338	-0.476618
O	4.131237	-10.750255	1.494188
B	3.472150	-11.682895	0.574084
O	2.738934	-12.768305	1.239650
O	2.488558	-10.972132	-0.235941
Si	6.238112	-7.856052	-0.203557
Si	4.266253	-6.935022	0.739812
Si	5.952182	-8.049059	-2.547659
Si	8.127471	-6.537027	0.338984
C	5.223957	-6.438294	-3.240928
H	5.876311	-5.579546	-3.013211
H	5.116298	-6.505261	-4.337258
H	4.230229	-6.230530	-2.812857
C	7.604175	-8.401112	-3.412871
H	7.452941	-8.555980	-4.495188
H	8.308512	-7.563205	-3.282961
H	8.074623	-9.308087	-2.998720
C	4.774095	-9.498499	-2.888514
H	5.290610	-10.448423	-2.682794
H	3.875785	-9.461285	-2.252637
H	4.457551	-9.507387	-3.945526
C	8.079621	-4.938593	-0.690953
H	8.124632	-5.164583	-1.769263
H	7.159139	-4.362808	-0.504664
H	8.941493	-4.294881	-0.443544
C	8.147680	-6.095431	2.184002
H	8.188898	-7.020136	2.781032
H	9.028634	-5.478839	2.433336
H	7.243776	-5.534146	2.472479
C	9.709565	-7.491178	-0.090108
H	9.760313	-8.410385	0.513078
H	9.729093	-7.770451	-1.156102
H	10.604399	-6.880669	0.122430
C	4.392382	-5.049286	0.514594
H	4.540670	-4.779039	-0.543798
H	3.464692	-4.563046	0.862733
H	5.232084	-4.630240	1.092558
C	2.698824	-7.531130	-0.143809
H	2.707400	-7.240701	-1.207047
H	2.604859	-8.626138	-0.089140
H	1.807367	-7.077651	0.323985
C	4.113008	-7.319613	2.589292
H	3.254051	-6.776199	3.020378
H	3.959729	-8.399133	2.735602
H	5.022332	-7.024108	3.136713
C	4.620960	-12.836703	-1.383831
O	4.613343	-12.232804	-0.250385
N	5.814719	-13.015118	-1.920311
C	6.083037	-13.662956	-3.203032
H	5.164726	-14.008744	-3.683250
H	6.580863	-12.945213	-3.871794
H	6.749795	-14.523124	-3.044467
C	6.990908	-12.516874	-1.183201
H	7.115143	-13.083917	-0.248971
H	7.876595	-12.649601	-1.813747
H	6.860480	-11.455972	-0.924230
C	3.335608	-13.268605	-2.012804
H	2.808705	-12.367523	-2.360186
H	3.458803	-13.963783	-2.846351
H	2.711729	-13.736063	-1.239769

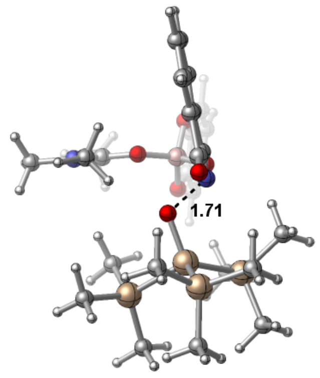

Zero-point correction= 0.668111 (Hartree/Particle)
Thermal correction to Energy= 0.718545
Thermal correction to Enthalpy= 0.719489
Thermal correction to Gibbs Free Energy= 0.581847
Sum of electronic and zero-point Energies= -2797.246881
Sum of electronic and thermal Energies= -2797.196447
Sum of electronic and thermal Enthalpies= -2797.195503
Sum of electronic and thermal Free Energies= -2797.333145

UB3LYP-D3/def2TZVPP
E(scf)= -2800.166192
UM062X-D3/def2TZVPP
E(scf)= -2799.212938
uωB97XD/def2TZVPP
E(scf)= -2799.45584829

IV
E(scf) = -1591.93624173 a.u.
ν_{min} = 21.3135 cm^{-1}

O	6.570407	-8.841406	-0.025284	H	3.852922	-7.417329	-2.047508
Si	6.999702	-7.294847	-0.282503	H	4.263566	-7.248350	-3.778333
Si	5.555392	-6.453309	1.535846	C	10.371495	-6.858574	-1.162243
Si	6.142337	-6.424783	-2.333899	H	10.258060	-7.793786	-1.733933
Si	9.252903	-6.842779	0.369485	H	10.136701	-6.017294	-1.833807
C	5.600147	-4.614212	-2.197064	H	11.428001	-6.766775	-0.855916
H	6.410723	-3.978754	-1.804700	C	9.447663	-5.176577	1.251134
H	5.318167	-4.231182	-3.192913	H	8.916347	-5.167955	2.216217
H	4.726911	-4.506936	-1.534084	H	10.515367	-4.981812	1.451097
C	7.469954	-6.568899	-3.681209	H	9.059743	-4.347850	0.636767
H	7.046877	-6.272302	-4.656678	C	9.744727	-8.255083	1.532021
H	8.328327	-5.912422	-3.467227	H	9.136164	-8.242226	2.450282
H	7.843667	-7.602043	-3.767563	H	9.588530	-9.227679	1.038620
C	4.663761	-7.518967	-2.786357	H	10.806880	-8.174837	1.819856
H	4.967680	-8.577796	-2.808931	C	5.696665	-4.574634	1.644238

H	5.371352	-4.089458	0.712293
H	5.044202	-4.222768	2.463038
H	6.726227	-4.255998	1.863318
C	3.802994	-6.991403	1.103875
H	3.439000	-6.479989	0.198988
H	3.770205	-8.077376	0.929018
H	3.121099	-6.744009	1.935769
C	6.174314	-7.289304	3.106080
H	7.191058	-6.954117	3.364072
H	5.506993	-7.042899	3.949909
H	6.190071	-8.381579	2.972811

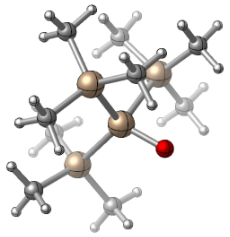

Zero-point correction= 0.335010 (Hartree/Particle)
Thermal correction to Energy= 0.362611
Thermal correction to Enthalpy= 0.363555
Thermal correction to Gibbs Free Energy= 0.276671
Sum of electronic and zero-point Energies= -1591.601232
Sum of electronic and thermal Energies= -1591.573631
Sum of electronic and thermal Enthalpies= -1591.572687
Sum of electronic and thermal Free Energies= -1591.659570

UB3LYP-D3/def2TZVPP
E(scf)= -1592.867964
UM062X-D3/def2TZVPP
E(scf)= -1592.42562
uωB97XD/def2TZVPP
E(scf)= -1592.60962286

VII
E(scf) = -1205.98761517 a.u.
ν_{min} = 13.0196 cm^{-1}

C	6.084427	-11.935183	2.153031
C	7.307385	-11.527334	2.699763
C	5.885082	-13.259804	1.774060
C	8.374317	-12.397336	2.857560
C	6.962474	-14.152058	1.926624
H	4.929595	-13.611748	1.390174
C	8.190518	-13.731248	2.450669
H	9.319272	-12.054687	3.285244
H	6.833702	-15.195982	1.631092
H	9.008073	-14.448794	2.552922
N	5.858706	-9.655410	2.686312
C	7.148536	-10.070873	3.055009
O	7.988646	-9.362016	3.572085
C	5.245836	-10.681689	2.153039
C	1.488713	-12.663346	1.316674
C	1.046779	-11.489433	0.678316
C	0.595156	-13.612059	1.791335
C	-0.304435	-11.231306	0.496207
C	-0.779894	-13.354208	1.614623
H	0.952408	-14.520579	2.280380
C	-1.220213	-12.187974	0.980423
H	-0.636057	-10.319507	-0.004663

H	-1.510002	-14.080622	1.979903	H	5.125615	-13.495986	-3.908009
H	-2.291372	-12.011635	0.854999	H	6.530608	-14.185571	-3.048902
O	4.036890	-10.557116	1.688177	C	6.654921	-12.187224	-1.558911
B	3.304642	-11.432029	0.753513	H	7.311270	-12.884327	-1.016706
O	2.848707	-12.687335	1.350156	H	7.147053	-11.874534	-2.490385
O	2.113413	-10.740510	0.288113	H	6.461574	-11.312732	-0.932667
C	4.265931	-12.571625	-1.283521	C	2.978203	-13.244291	-1.643011
O	4.308557	-11.685379	-0.350563	H	2.154083	-12.537639	-1.494851
N	5.396540	-12.864485	-1.896637	H	2.961438	-13.604358	-2.676449
C	5.480039	-13.891348	-2.943974	H	2.819918	-14.095209	-0.961934
H	4.902188	-14.782428	-2.671395				

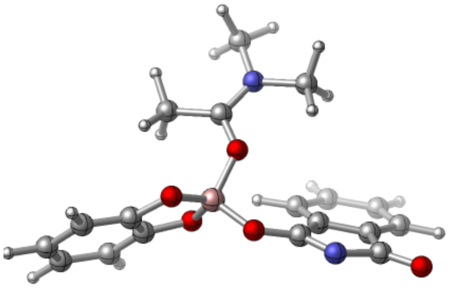

Zero-point correction= 0.332293 (Hartree/Particle)
Thermal correction to Energy= 0.354983
Thermal correction to Enthalpy= 0.355927
Thermal correction to Gibbs Free Energy= 0.278129
Sum of electronic and zero-point Energies= -1205.655323
Sum of electronic and thermal Energies= -1205.632632
Sum of electronic and thermal Enthalpies= -1205.631688
Sum of electronic and thermal Free Energies= -1205.709486

UB3LYP-D3/def2TZVPP
E(scf)= -1207.329534
UM062X-D3/def2TZVPP
E(scf)= -1206.821042
uωB97XD/def2TZVPP
E(scf)= -1206.87974595

$TS_{IV\text{-}V}$
E(scf) = -1591.93299553 a.u.
ν_{min} = -192.5975 cm^{-1}

O	7.106415	-9.085132	-0.398076	Si	9.753664	-6.804549	-0.354239
Si	7.530275	-7.538781	-0.735240	C	5.487519	-4.868962	-1.676405
Si	6.224084	-7.782436	1.328433	H	6.247590	-4.200587	-1.239657
Si	6.292596	-6.412846	-2.426174	H	4.940840	-4.305669	-2.452008

H	4.773446	-5.138373	-0.881562	C	10.454193	-7.635294	1.196180
C	7.438085	-5.891282	-3.844705	H	9.913881	-7.304847	2.097885
H	6.858636	-5.382321	-4.634261	H	10.367830	-8.731795	1.127474
H	8.215666	-5.194413	-3.491298	H	11.520105	-7.379879	1.322667
H	7.939986	-6.763435	-4.294250	C	6.242715	-6.053449	2.141672
C	4.967862	-7.607707	-3.059320	H	5.834024	-5.284720	1.467669
H	5.432127	-8.513537	-3.481959	H	5.623684	-6.083354	3.057026
H	4.297245	-7.920610	-2.243478	H	7.265607	-5.758828	2.425839
H	4.357505	-7.132503	-3.846124	C	4.447660	-8.211213	0.873906
C	10.840591	-7.213276	-1.852971	H	4.018501	-7.459695	0.192091
H	10.898761	-8.301657	-2.016538	H	4.398707	-9.194557	0.384779
H	10.441356	-6.749628	-2.769563	H	3.826319	-8.231725	1.785762
H	11.865077	-6.834602	-1.693608	C	6.981526	-8.995046	2.551786
C	9.680193	-4.925111	-0.113777	H	8.054442	-8.791212	2.693273
H	9.051244	-4.661324	0.751956	H	6.483887	-8.895281	3.531683
H	10.692433	-4.521931	0.060982	H	6.872366	-10.028563	2.193534
H	9.263903	-4.423502	-1.002783				

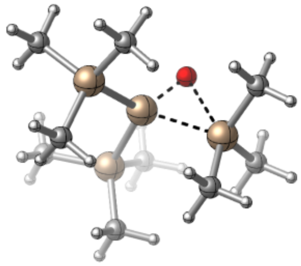

Zero-point correction= 0.335046 (Hartree/Particle)
Thermal correction to Energy= 0.361743
Thermal correction to Enthalpy= 0.362687
Thermal correction to Gibbs Free Energy= 0.278087
Sum of electronic and zero-point Energies= -1591.597949
Sum of electronic and thermal Energies= -1591.571252
Sum of electronic and thermal Enthalpies= -1591.570308
Sum of electronic and thermal Free Energies= -1591.654909
UB3LYP-D3/def2TZVPP
E(scf)= -1592.865014
UM062X-D3/def2TZVPP
E(scf)= -1592.426186
uωB97XD/def2TZVPP
E(scf)= -1592.60839836

V

E(scf) = -1592.00399167 a.u.

ν_{min} = 19.1560 cm^{-1}

Si	5.728586	-9.007217	-1.322573	Si	3.402864	-9.417523	-1.535188

Si	7.146897	-10.219991	-2.775049
O	6.087598	-7.380699	-1.615364
Si	5.727703	-5.792717	-1.200315
C	7.236993	-4.764119	-1.637885
H	7.071257	-3.700219	-1.399346
H	8.121388	-5.107094	-1.075873
H	7.464410	-4.841053	-2.714049
C	4.235488	-5.230509	-2.199985
H	4.415943	-5.365173	-3.279277
H	3.333291	-5.803181	-1.931688
H	4.024459	-4.163204	-2.019352
C	5.365509	-5.709437	0.644406
H	6.237470	-6.047025	1.228756
H	5.125412	-4.678227	0.952971
H	4.509300	-6.351699	0.908383
C	2.793757	-8.850507	-3.243049
H	1.704161	-9.003855	-3.333914
H	3.004664	-7.781250	-3.403360
H	3.290583	-9.419004	-4.045747
C	2.423401	-8.493311	-0.199158
H	2.556336	-7.402683	-0.282635
H	1.346123	-8.710617	-0.300316
H	2.743441	-8.797964	0.810458
C	3.110242	-11.282309	-1.353786
H	3.468229	-11.651974	-0.379113
H	2.032462	-11.506522	-1.430537
H	3.631546	-11.845188	-2.145215
C	6.604372	-9.945418	-4.575199
H	7.304363	-10.440900	-5.270617
H	5.596390	-10.354971	-4.751232
H	6.582334	-8.870170	-4.817024
C	8.912871	-9.564567	-2.563459
H	9.293126	-9.765263	-1.548560
H	9.594929	-10.040397	-3.288781
H	8.940656	-8.474899	-2.727985
C	7.079375	-12.071054	-2.375204
H	7.409406	-12.267851	-1.342181
H	6.056165	-12.465523	-2.486780
H	7.737289	-12.634470	-3.058883

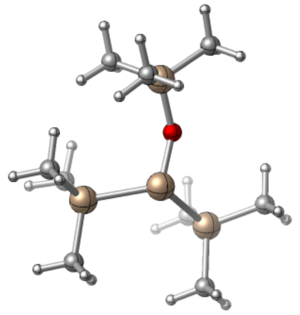

Zero-point correction= 0.335985 (Hartree/Particle)
Thermal correction to Energy= 0.363141
Thermal correction to Enthalpy= 0.364085
Thermal correction to Gibbs Free Energy= 0.277458
Sum of electronic and zero-point Energies= -1591.668007
Sum of electronic and thermal Energies= -1591.640851
Sum of electronic and thermal Enthalpies= -1591.639906
Sum of electronic and thermal Free Energies= -1591.726533
UB3LYP-D3/def2TZVPP
E(scf)= -1592.93435
UM062X-D3/def2TZVPP
E(scf)= -1592.502257
uωB97XD/def2TZVPP
E(scf)= -1592.67604967

1a
E(scf) = -2923.23364179 a.u.
ν_{min} = 27.0597 cm^{-1}

C	-1.680826	-0.298341	-0.006712	C	1.949655	0.908931	0.001403
C	-0.282217	-0.296605	0.013070	H	2.334317	1.791490	0.539394
C	0.437702	0.909511	0.031942	H	2.333462	0.020474	0.530212
C	-0.281071	2.116439	0.027292	C	2.497975	0.916085	-1.439832
C	-1.679686	2.119721	0.007566	H	2.119892	0.033696	-1.981528
C	-2.384974	0.911087	-0.009844	H	2.120905	1.804467	-1.972354
H	-2.222833	-1.247772	-0.016877	C	4.016423	0.915260	-1.452388
H	0.261509	-1.246061	0.017147	H	4.436772	0.017892	-0.979299
H	0.263546	3.065270	0.042538	H	4.437833	1.807184	-0.970036
H	-2.220798	3.069717	0.008617	Br	4.736823	0.924384	-3.299965
H	-3.477939	0.911677	-0.022806				

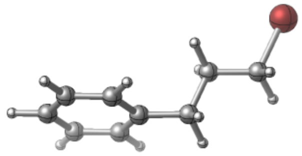

Zero-point correction= 0.175998 (Hartree/Particle)
Thermal correction to Energy= 0.185732
Thermal correction to Enthalpy= 0.186676
Thermal correction to Gibbs Free Energy= 0.137933
Sum of electronic and zero-point Energies= -2923.057644
Sum of electronic and thermal Energies= -2923.047910
Sum of electronic and thermal Enthalpies= -2923.046966
Sum of electronic and thermal Free Energies= -2923.095709

UB3LYP-D3/def2TZVPP
E(scf)= -2923.931155
UM062X-D3/def2TZVPP
E(scf)= -2923.757015
uωB97XD/def2TZVPP
E(scf)= -2923.823604

TS$_{V\text{-}1a^{\cdot}}$
E(scf) = -4515.24581422 a.u.

ν_{min} = -120.2731 cm^{-1}

Si	4.917812	-9.498556	-1.766124	H	3.984763	-9.500997	-5.366587
Si	3.119517	-8.077485	-1.209881	H	5.769040	-9.468047	-5.350837
Si	4.891921	-11.006119	-3.570808	C	6.457493	-12.060748	-3.411291
O	6.426349	-8.768124	-1.682441	H	6.459370	-12.607985	-2.454796
Si	7.448972	-7.860448	-0.696023	H	6.523288	-12.793428	-4.233614
C	9.191728	-8.148884	-1.331547	H	7.357545	-11.425093	-3.441995
H	9.924746	-7.573072	-0.742245	C	3.347333	-12.089078	-3.405587
H	9.460970	-9.215918	-1.264006	H	3.331589	-12.595164	-2.426899
H	9.281636	-7.838638	-2.385684	H	2.427325	-11.488800	-3.494115
C	6.968706	-6.048803	-0.860124	H	3.330650	-12.858923	-4.195748
H	7.009715	-5.727817	-1.914091	C	7.033580	-10.406890	7.425020
H	5.945511	-5.872585	-0.490610	C	6.213714	-11.087035	6.518428
H	7.652848	-5.407130	-0.280119	C	6.756768	-11.995166	5.594334
C	7.269004	-8.445250	1.082058	C	8.146544	-12.201213	5.599736
H	7.499601	-9.519269	1.166264	C	8.970557	-11.523444	6.504137
H	7.951174	-7.888053	1.745794	C	8.416290	-10.622728	7.420827
H	6.239013	-8.298587	1.444618	H	6.590644	-9.707508	8.139218
C	3.084394	-6.524075	-2.297369	H	5.133375	-10.913961	6.528542
H	2.249178	-5.865969	-2.000556	H	8.586833	-12.904945	4.886891
H	4.021842	-5.953413	-2.198186	H	10.049300	-11.701287	6.494930
H	2.955439	-6.788079	-3.359565	H	9.058294	-10.093853	8.130001
C	3.268362	-7.569738	0.610119	C	5.876375	-12.686247	4.577879
H	4.164658	-6.953635	0.786275	H	6.286423	-13.682478	4.342310
H	2.386698	-6.979874	0.914034	H	4.870181	-12.846024	4.999702
H	3.334536	-8.460350	1.255141	C	5.753362	-11.869775	3.271912
C	1.532927	-9.083627	-1.457431	H	5.346631	-10.871809	3.506540
H	1.560607	-10.013202	-0.866113	H	6.760364	-11.707262	2.852349
H	0.655826	-8.496627	-1.135716	C	4.876209	-12.553904	2.249473
H	1.392919	-9.354713	-2.516459	H	3.827639	-12.655852	2.558484
C	4.887680	-10.121994	-5.248712	H	5.271517	-13.515061	1.895520
H	4.909271	-10.858633	-6.070851	Br	4.779900	-11.362174	0.496749

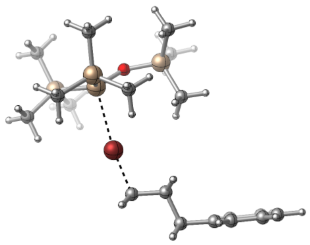

Zero-point correction= 0.511099 (Hartree/Particle)
Thermal correction to Energy= 0.549874
Thermal correction to Enthalpy= 0.550818
Thermal correction to Gibbs Free Energy= 0.431047
Sum of electronic and zero-point Energies= -4514.734715

Sum of electronic and thermal Energies= -4514.695940
Sum of electronic and thermal Enthalpies= -4514.694996
Sum of electronic and thermal Free Energies= -4514.814767

UB3LYP-D3/def2TZVPP
E(scf)= -4516.870721
UM062X-D3/def2TZVPP
E(scf)= -4516.260881
uωB97XD/def2TZVPP
E(scf)= -4516.50045072

1a·
E(scf) = -349.288662574 a.u.
ν_{min} = 42.2801 cm^{-1}

C	-1.702531	-0.298164	-0.027117	H	-3.498210	0.912302	-0.100340
C	-0.305225	-0.296659	0.036737	C	1.926057	0.908040	0.091455
C	0.415395	0.908985	0.078728	H	2.299907	1.790691	0.635607
C	-0.303794	2.115878	0.050132	H	2.298900	0.018775	0.625429
C	-1.701094	2.119746	-0.013697	C	2.527731	0.915966	-1.347383
C	-2.406163	0.911390	-0.052872	H	2.132832	0.032651	-1.879940
H	-2.244296	-1.247496	-0.054054	H	2.133841	1.805797	-1.869739
H	0.237688	-1.246439	0.058404	C	4.017171	0.915235	-1.367490
H	0.240245	3.064714	0.082350	H	4.575935	-0.021559	-1.279759
H	-2.241736	3.069959	-0.030078	H	4.576996	1.850312	-1.268866

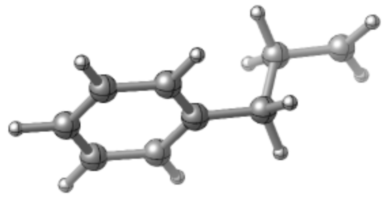

Zero-point correction= 0.169914 (Hartree/Particle)
Thermal correction to Energy= 0.178974
Thermal correction to Enthalpy= 0.179918
Thermal correction to Gibbs Free Energy= 0.134370
Sum of electronic and zero-point Energies= -349.118749
Sum of electronic and thermal Energies= -349.109689
Sum of electronic and thermal Enthalpies= -349.108745
Sum of electronic and thermal Free Energies= -349.154293

UB3LYP-D3/def2TZVPP
E(scf)= -349.6723034
UM062X-D3/def2TZVPP
E(scf)= -349.4855001

uωB97XD/def2TZVPP
E(scf)= -349.530512104

VIII
E(scf) = -4165.98588858 a.u.
ν_{min} = 19.2372 cm^{-1}

Si	5.659741	-8.992155	-1.475394	C	2.458159	-8.484888	-0.185958
Si	3.340325	-9.442489	-1.559993	H	2.533918	-7.397229	-0.343166
Si	7.156882	-10.203694	-2.836161	H	1.387811	-8.751098	-0.161875
O	6.000687	-7.378299	-1.693859	H	2.896441	-8.721208	0.796903
Si	5.723974	-5.787909	-1.203491	C	3.099476	-11.303348	-1.314219
C	7.292167	-4.835667	-1.598694	H	3.533949	-11.629457	-0.355420
H	7.188239	-3.773034	-1.322986	H	2.025584	-11.555979	-1.310497
H	8.150384	-5.251185	-1.045233	H	3.583223	-11.876630	-2.121824
H	7.520700	-4.888831	-2.675948	C	6.656711	-9.957197	-4.645954
C	4.266684	-5.124991	-2.189057	H	7.365777	-10.471729	-5.316630
H	4.453329	-5.217433	-3.271676	H	5.649513	-10.362957	-4.835852
H	3.341867	-5.676459	-1.954073	H	6.649612	-8.887015	-4.909759
H	4.092571	-4.060062	-1.961494	C	8.882194	-9.492926	-2.522505
C	5.358602	-5.767973	0.639765	H	9.167286	-9.619731	-1.465560
H	6.194598	-6.206797	1.207353	H	9.636326	-9.998776	-3.148893
H	5.201263	-4.734959	0.992492	H	8.903348	-8.415847	-2.754895
H	4.452528	-6.350695	0.870708	C	7.089843	-12.035589	-2.370852
C	2.694472	-8.904831	-3.256987	H	7.325590	-12.173142	-1.303310
H	1.608906	-9.086440	-3.333183	H	6.087822	-12.455405	-2.556247
H	2.875518	-7.830636	-3.423812	H	7.818652	-12.613295	-2.964212
H	3.193658	-9.464706	-4.064685	Br	6.274077	-9.535670	0.713141

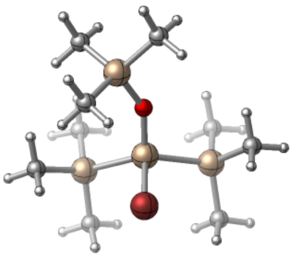

Zero-point correction= 0.337783 (Hartree/Particle)
Thermal correction to Energy= 0.366807
Thermal correction to Enthalpy= 0.367751
Thermal correction to Gibbs Free Energy= 0.276388
Sum of electronic and zero-point Energies= -4165.648106
Sum of electronic and thermal Energies= -4165.619081
Sum of electronic and thermal Enthalpies= -4165.618137

Sum of electronic and thermal Free Energies= -4165.709500

UB3LYP-D3/def2TZVPP
E(scf)= -4167.229747
UM062X-D3/def2TZVPP
E(scf)= -4166.809381
uωB97XD/def2TZVPP
E(scf)= -4167.00504345

TS$_{1a^{\cdot}-VI}$
E(scf) = -1449.27798041 a.u.
ν_{min} = -85.1280 cm^{-1}

C	8.376242	-5.170310	-1.239819	H	5.722848	-4.403013	4.583436
C	7.227967	-5.471644	-2.001489	H	4.322536	-5.497704	4.744314
C	6.936166	-4.776784	-3.168219	C	3.965333	-5.440028	2.265050
C	7.827265	-3.757647	-3.566807	H	4.060501	-5.473162	1.176121
C	8.965119	-3.459535	-2.811305	H	3.287144	-6.241314	2.597483
C	9.257311	-4.169157	-1.626837	H	3.543414	-4.470001	2.567306
H	6.044597	-5.018914	-3.751081	C	7.688938	-6.048764	2.853308
H	7.620691	-3.194888	-4.480866	H	7.850266	-5.239716	3.578125
H	9.640990	-2.665340	-3.138871	H	7.725040	-7.011627	3.385388
H	10.143548	-3.945769	-1.028580	H	8.483437	-6.030596	2.102341
C	7.917196	-10.190814	1.554515	C	3.438584	-14.430923	1.481784
C	8.182326	-10.510629	0.215539	C	3.405994	-13.038054	1.362123
C	8.113160	-11.106569	2.579803	C	3.396694	-12.421142	0.099395
C	8.653901	-11.762136	-0.157924	C	3.426747	-13.238877	-1.042315
C	8.592676	-12.376192	2.212461	C	3.458721	-14.632845	-0.927824
H	7.896277	-10.847634	3.617669	C	3.465254	-15.234214	0.335687
C	8.857164	-12.696951	0.872364	H	3.441064	-14.891418	2.473423
H	8.848908	-12.002503	-1.204518	H	3.385472	-12.416315	2.262348
H	8.759815	-13.127663	2.987718	H	3.422904	-12.775738	-2.033581
H	9.226620	-13.694160	0.621826	H	3.477238	-15.251708	-1.828975
O	8.449601	-5.992013	-0.164034	H	3.488961	-16.323197	0.427080
O	6.543234	-6.492530	-1.433962	C	3.430845	-10.915762	-0.021869
O	7.879782	-9.434545	-0.568632	H	2.969998	-10.598843	-0.971234
O	7.444299	-8.911655	1.620003	H	2.845435	-10.456108	0.791297
B	7.187046	-6.770307	-0.140037	C	4.888406	-10.374318	0.041492
B	7.374737	-8.432295	0.288740	H	5.328576	-10.723238	0.991002
C	6.356167	-5.915179	2.173447	H	5.468186	-10.848101	-0.767211
O	6.216198	-6.085394	0.918671	C	4.975551	-8.890729	-0.056307
N	5.277877	-5.611261	2.890723	H	4.635725	-8.282523	0.786785
C	5.337818	-5.405945	4.339980	H	4.951793	-8.404398	-1.034755
H	5.965817	-6.163579	4.824911				

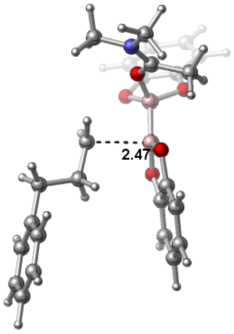

Zero-point correction= 0.491251 (Hartree/Particle)
Thermal correction to Energy= 0.522328
Thermal correction to Enthalpy= 0.523273
Thermal correction to Gibbs Free Energy= 0.422348
Sum of electronic and zero-point Energies= -1448.786729
Sum of electronic and thermal Energies= -1448.755652
Sum of electronic and thermal Enthalpies= -1448.754708
Sum of electronic and thermal Free Energies= -1448.855633
UB3LYP-D3/def2TZVPP
E(scf)= -1450.878858
UM062X-D3/def2TZVPP
E(scf)= -1450.225283
uωB97XD/def2TZVPP
E(scf)= -1450.32520492

VI

E(scf) = -1449.31613345 a.u.

ν_{min} =11.6875 cm^{-1}

C	8.465494	-6.047723	-1.092686
C	7.301407	-5.914465	-1.875059
C	7.369555	-5.565531	-3.217837
C	8.649023	-5.349708	-3.766469
C	9.805167	-5.486287	-2.988384
C	9.729955	-5.842698	-1.627549
H	6.461942	-5.466700	-3.816573
H	8.737437	-5.074643	-4.820372
H	10.784670	-5.317306	-3.442408
H	10.622928	-5.958025	-1.010259
C	8.291006	-9.355917	1.355601
C	8.535630	-9.470727	-0.029518
C	9.273624	-9.655168	2.292917
C	9.772244	-9.884184	-0.513393
C	10.524877	-10.079757	1.807169
H	9.073691	-9.562189	3.362284
C	10.769143	-10.191147	0.431692
H	9.952980	-9.960384	-1.587441
H	11.318756	-10.323658	2.517490
H	11.751642	-10.519842	0.083781
O	8.129977	-6.394947	0.176208
O	6.202313	-6.178406	-1.122900
O	7.433810	-9.119934	-0.726573
O	7.027302	-8.924645	1.567053
B	6.701054	-6.657263	0.147256
B	6.468177	-8.607500	0.245064
C	6.083170	-6.207034	2.544043
O	5.909750	-6.126568	1.270017
N	5.010761	-6.115134	3.314299
C	5.125157	-6.123134	4.776779

H	5.670198	-7.010806	5.124963	C	2.399961	-15.014061	0.128319
H	5.639092	-5.218907	5.135515	H	2.076860	-14.661395	2.239765
H	4.116512	-6.145728	5.203385	H	2.457715	-12.211442	2.078071
C	3.660783	-5.971753	2.762011	H	3.146855	-12.591789	-2.159542
H	3.695890	-5.967156	1.669511	H	2.766796	-15.042313	-2.005099
H	3.039657	-6.811923	3.106024	H	2.230657	-16.091769	0.197496
H	3.218583	-5.029780	3.119331	C	3.119015	-10.745961	-0.137326
C	7.438209	-6.359514	3.161740	H	2.811863	-10.360660	-1.124770
H	7.631912	-5.525259	3.852913	H	2.518562	-10.203881	0.613503
H	7.482937	-7.295374	3.736796	C	4.606904	-10.414777	0.084558
H	8.207914	-6.379488	2.387096	H	4.916572	-10.809130	1.068618
C	2.314244	-14.211546	1.271811	H	5.209840	-10.961426	-0.661955
C	2.528218	-12.832282	1.179696	C	4.919394	-8.913273	0.005642
C	2.829342	-12.226228	-0.052113	H	4.312778	-8.370456	0.751708
C	2.913669	-13.045015	-1.191223	H	4.618023	-8.515753	-0.978956
C	2.700523	-14.424851	-1.105112				

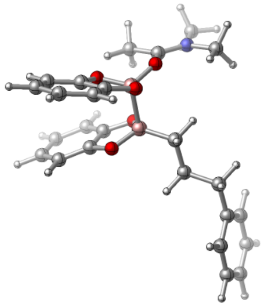

Zero-point correction= 0.494541 (Hartree/Particle)
Thermal correction to Energy= 0.525154
Thermal correction to Enthalpy= 0.526098
Thermal correction to Gibbs Free Energy= 0.427319
Sum of electronic and zero-point Energies= -1448.821593
Sum of electronic and thermal Energies= -1448.790979
Sum of electronic and thermal Enthalpies= -1448.790035
Sum of electronic and thermal Free Energies= -1448.888815

UB3LYP-D3/def2TZVPP
E(scf)= -1450.878858
UM062X-D3/def2TZVPP
E(scf)= -1450.225283
uωB97XD/def2TZVPP
E(scf)= -1450.36077531

3
E(scf) = -1450.225283 a.u.
ν_{min} = 5.1936 cm^{-1}

C	6.093140	-8.741866	3.136700	C	5.469654	-9.985323	3.292741

C	6.484841	-7.975070	4.224416
C	5.206658	-10.524318	4.543530
C	6.223526	-8.510228	5.498101
H	6.970310	-7.007038	4.090241
C	5.598567	-9.756449	5.654276
H	4.719995	-11.495025	4.651521
H	6.514489	-7.941055	6.383992
H	5.411312	-10.140840	6.659545
O	5.202100	-10.504692	2.048946
O	6.220215	-8.475322	1.794915
B	5.665633	-9.566842	1.130590
C	2.823606	-14.150115	-3.444727
C	3.222944	-12.870031	-3.046678
C	4.583900	-12.538862	-2.933432
C	5.535733	-13.529597	-3.227864
C	5.141978	-14.811372	-3.626533
C	3.782908	-15.126589	-3.736303
H	1.759271	-14.385365	-3.530542
H	2.466892	-12.111343	-2.822699
H	6.600361	-13.290285	-3.146509
H	5.899372	-15.566102	-3.855109
H	3.473016	-16.126755	-4.050163
C	5.010676	-11.171835	-2.450502
H	5.982638	-10.903915	-2.899050
H	4.286230	-10.412450	-2.791777
C	5.129768	-11.091601	-0.918753
H	4.159973	-11.364539	-0.468162
H	5.846474	-11.857454	-0.574459
C	5.564875	-9.708051	-0.420012
H	4.856370	-8.936320	-0.779604
H	6.536867	-9.423122	-0.863431

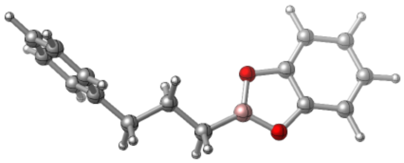

Zero-point correction= 0.268941 (Hartree/Particle)
Thermal correction to Energy= 0.283889
Thermal correction to Enthalpy= 0.284833
Thermal correction to Gibbs Free Energy= 0.221879
Sum of electronic and zero-point Energies= -755.265162
Sum of electronic and thermal Energies= -755.250215
Sum of electronic and thermal Enthalpies= -755.249271
Sum of electronic and thermal Free Energies= -755.312225

UB3LYP-D3/def2TZVPP
E(scf)= -756.3642047
UM062X-D3/def2TZVPP
E(scf)= -756.0202999
uωB97XD/def2TZVPP
E(scf)= -756.07334444

TS$_{VI-3}$
E(scf) = -1449.30884871 a.u.
ν_{min} = -594.9578 cm^{-1}

C	6.299867	-6.201929	-1.873361
C	5.090342	-5.786079	-1.289935
C	4.012671	-5.380284	-2.064458
C	4.184119	-5.401600	-3.462632

C	5.389261	-5.816550	-4.041898
C	6.477795	-6.229138	-3.248127
H	3.076399	-5.062926	-1.601880
H	3.357000	-5.089487	-4.104739
H	5.490299	-5.824339	-5.129769
H	7.421010	-6.559816	-3.686603
C	5.343372	-8.764791	3.068518
C	6.700718	-9.103804	2.955462
C	4.722839	-8.644701	4.306205
C	7.492982	-9.333913	4.071796
C	5.516178	-8.881763	5.443741
H	3.666808	-8.379945	4.382763
C	6.872112	-9.220136	5.329581
H	8.548457	-9.590624	3.970210
H	5.064635	-8.798064	6.435106
H	7.461844	-9.394907	6.232453
O	7.179783	-6.561918	-0.890600
O	5.180189	-5.874894	0.068840
O	7.047069	-9.139681	1.635874
O	4.813676	-8.602316	1.824222
B	6.457094	-6.440032	0.327525
B	5.878193	-8.792132	0.904221
C	8.240055	-6.392574	2.078960
O	7.045247	-6.199143	1.557043
N	8.311757	-6.318560	3.429023
C	9.551866	-6.579563	4.142127
H	9.306539	-6.946232	5.148798
H	10.148444	-7.351872	3.643362
H	10.162942	-5.663875	4.244459
C	7.235135	-5.759746	4.235304
H	6.356895	-5.555228	3.616450
H	6.952806	-6.476720	5.021592
H	7.566170	-4.820756	4.713124
C	9.390334	-6.858119	1.249715
H	10.323118	-6.348016	1.530160
H	9.544004	-7.946452	1.372559
H	9.190527	-6.666239	0.190882
C	2.685694	-14.274482	-2.129101
C	3.107218	-12.940837	-2.112573
C	4.471476	-12.611655	-2.186731
C	5.403837	-13.659485	-2.274673
C	4.987849	-14.994873	-2.291579
C	3.625609	-15.307643	-2.218665
H	1.618890	-14.508048	-2.074900
H	2.366111	-12.138646	-2.044411
H	6.470598	-13.423038	-2.333983
H	5.730023	-15.794471	-2.364945
H	3.298191	-16.350443	-2.234377
C	4.926835	-11.173240	-2.110760
H	5.856894	-11.043074	-2.690066
H	4.171584	-10.515754	-2.574446
C	5.173101	-10.701577	-0.665759
H	4.245653	-10.835195	-0.081871
H	5.925523	-11.360235	-0.197167
C	5.637741	-9.233703	-0.586102
H	4.878583	-8.590275	-1.061470
H	6.562981	-9.105488	-1.169975

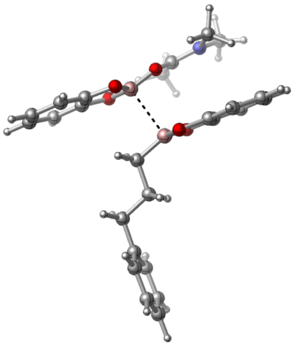

Zero-point correction= 0.492932 (Hartree/Particle)
Thermal correction to Energy= 0.523584
Thermal correction to Enthalpy= 0.524528
Thermal correction to Gibbs Free Energy= 0.426523
Sum of electronic and zero-point Energies= -1448.815917
Sum of electronic and thermal Energies= -1448.785265

Sum of electronic and thermal Enthalpies= -1448.784320
Sum of electronic and thermal Free Energies= -1448.882326
UB3LYP-D3/def2TZVPP
E(scf)= -1450.905789
UM062X-D3/def2TZVPP
E(scf)= -1450.249776
uωB97XD/def2TZVPP
E(scf)= -1450.3490686

$TS_{1a^{\cdot}-VI'}$
E(scf) = -1161.62115491 a.u.
ν_{min} = -109.0628 cm^{-1}

C	7.553431	-5.635187	-2.357968	O	6.520931	-8.400609	1.612064
C	6.579482	-5.040570	-1.547468	B	7.162972	-6.970356	-0.639005
C	6.012060	-3.813037	-1.859858	B	7.175274	-8.319083	0.368888
C	6.464540	-3.193708	-3.037239	C	3.150828	-14.155489	0.780793
C	7.441479	-3.790010	-3.849976	C	3.221479	-12.837742	0.318270
C	8.009785	-5.032570	-3.521980	C	3.909182	-12.524791	-0.866494
H	5.253973	-3.359352	-1.219441	C	4.530582	-13.567059	-1.573817
H	6.045442	-2.226683	-3.324582	C	4.462014	-14.886866	-1.115196
H	7.768590	-3.278471	-4.758079	C	3.771739	-15.185617	0.064816
H	8.770269	-5.505933	-4.145068	H	2.607261	-14.379564	1.702539
C	6.951656	-9.558433	2.195066	H	2.734352	-12.037166	0.883112
C	7.891638	-10.172815	1.354338	H	5.072800	-13.340356	-2.496654
C	6.567286	-10.125087	3.403761	H	4.948266	-15.685089	-1.682373
C	8.489613	-11.382731	1.682518	H	3.716133	-16.216460	0.424001
C	7.167848	-11.347863	3.745446	C	4.037490	-11.091205	-1.325974
H	5.830902	-9.640202	4.046598	H	4.160940	-11.050140	-2.419892
C	8.108308	-11.963240	2.903238	H	3.121631	-10.529540	-1.080788
H	9.214546	-11.853835	1.016819	C	5.253404	-10.393243	-0.650007
H	6.894501	-11.830677	4.686451	H	5.112045	-10.462715	0.440725
H	8.552287	-12.915728	3.201545	H	6.161707	-10.966239	-0.894334
O	7.924657	-6.832786	-1.798293	C	5.404515	-8.970037	-1.071076
O	6.334129	-5.861652	-0.474890	H	4.664805	-8.243619	-0.717507
O	8.060521	-9.404536	0.237666	H	5.815469	-8.769956	-2.066854

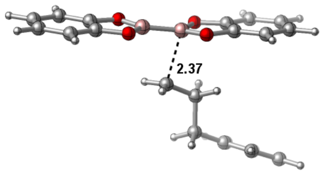

Zero-point correction= 0.360198 (Hartree/Particle)
Thermal correction to Energy= 0.382461

Thermal correction to Enthalpy= 0.383405
Thermal correction to Gibbs Free Energy= 0.302843
Sum of electronic and zero-point Energies= -1161.260957
Sum of electronic and thermal Energies= -1161.238694
Sum of electronic and thermal Enthalpies= -1161.237750
Sum of electronic and thermal Free Energies= -1161.318312

VI'

E(scf) = -1161.63799273 a.u.

ν_{min} = 9.8626 cm^{-1}

C	7.671068	-5.506540	-2.137752	O	6.111238	-8.668048	1.177305
C	6.570125	-5.012648	-1.429962	B	6.829970	-7.152168	-0.916023
C	6.154510	-3.693528	-1.544027	B	6.515144	-8.730602	-0.280092
C	6.897049	-2.872722	-2.410658	C	2.359107	-14.433378	0.520114
C	8.001371	-3.368187	-3.120664	C	2.620520	-13.163312	-0.004708
C	8.412476	-4.706668	-2.995809	C	3.622631	-12.971544	-0.970952
H	5.293992	-3.319889	-0.986531	C	4.360609	-14.090234	-1.394006
H	6.606170	-1.826744	-2.532430	C	4.103605	-15.362353	-0.872428
H	8.555143	-2.701169	-3.785436	C	3.100724	-15.538727	0.087867
H	9.269555	-5.103588	-3.542406	H	1.571505	-14.560965	1.267748
C	6.977954	-9.359279	1.880237	H	2.035446	-12.304249	0.337450
C	7.992235	-9.907484	1.026481	H	5.145206	-13.960626	-2.145681
C	6.992742	-9.586296	3.266674	H	4.686619	-16.219970	-1.219037
C	9.036246	-10.690704	1.546565	H	2.896522	-16.532428	0.494974
C	8.029510	-10.364868	3.766018	C	3.942886	-11.590264	-1.493228
H	6.216305	-9.165238	3.906657	H	4.286432	-11.656080	-2.539661
C	9.036176	-10.908754	2.918734	H	3.030070	-10.970675	-1.498026
H	9.802145	-11.102965	0.888402	C	5.024747	-10.879129	-0.657093
H	8.078795	-10.568998	4.837812	H	4.682817	-10.825844	0.391053
H	9.827953	-11.513861	3.365718	H	5.933085	-11.506272	-0.648494
O	7.839917	-6.833538	-1.822971	C	5.362129	-9.476482	-1.168765
O	6.042584	-6.027778	-0.668925	H	4.459835	-8.839694	-1.167342
O	7.773809	-9.566919	-0.222188	H	5.707285	-9.523638	-2.216877

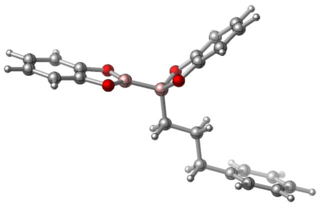

Zero-point correction= 0.361922 (Hartree/Particle)
Thermal correction to Energy= 0.384060
Thermal correction to Enthalpy= 0.385004
Thermal correction to Gibbs Free Energy= 0.304024
Sum of electronic and zero-point Energies= -1161.276071

Sum of electronic and thermal Energies= -1161.253933
Sum of electronic and thermal Enthalpies= -1161.252989
Sum of electronic and thermal Free Energies= -1161.333969

TS$_{VI'\text{-}3}$
E(scf) = -1449.27798041 a.u.
ν_{min} = -85.1280 cm^{-1}

C	8.376242	-5.170310	-1.239819
C	7.227967	-5.471644	-2.001489
C	6.936166	-4.776784	-3.168219
C	7.827265	-3.757647	-3.566807
C	8.965119	-3.459535	-2.811305
C	9.257311	-4.169157	-1.626837
H	6.044597	-5.018914	-3.751081
H	7.620691	-3.194888	-4.480866
H	9.640990	-2.665340	-3.138871
H	10.143548	-3.945769	-1.028580
C	7.917196	-10.190814	1.554515
C	8.182326	-10.510629	0.215539
C	8.113160	-11.106569	2.579803
C	8.653901	-11.762136	-0.157924
C	8.592676	-12.376192	2.212461
H	7.896277	-10.847634	3.617669
C	8.857164	-12.696951	0.872364
H	8.848908	-12.002503	-1.204518
H	8.759815	-13.127663	2.987718
H	9.226620	-13.694160	0.621826
O	8.449601	-5.992013	-0.164034
O	6.543234	-6.492530	-1.433962
O	7.879782	-9.434545	-0.568632
O	7.444299	-8.911655	1.620003
B	7.187046	-6.770307	-0.140037
B	7.374737	-8.432295	0.288740
C	6.356167	-5.915179	2.173447
O	6.216198	-6.085394	0.918671
N	5.277877	-5.611261	2.890723
C	5.337818	-5.405945	4.339980
H	5.965817	-6.163579	4.824911
H	5.722848	-4.403013	4.583436
H	4.322536	-5.497704	4.744314
C	3.965333	-5.440028	2.265050
H	4.060501	-5.473162	1.176121
H	3.287144	-6.241314	2.597483
H	3.543414	-4.470001	2.567306
C	7.688938	-6.048764	2.853308
H	7.850266	-5.239716	3.578125
H	7.725040	-7.011627	3.385388
H	8.483437	-6.030596	2.102341
C	3.438584	-14.430923	1.481784
C	3.405994	-13.038054	1.362123
C	3.396694	-12.421142	0.099395
C	3.426747	-13.238877	-1.042315
C	3.458721	-14.632845	-0.927824
C	3.465254	-15.234214	0.335687
H	3.441064	-14.891418	2.473423
H	3.385472	-12.416315	2.262348
H	3.422904	-12.775738	-2.033581
H	3.477238	-15.251708	-1.828975
H	3.488961	-16.323197	0.42708
C	3.430845	-10.915762	-0.021869
H	2.969998	-10.598843	-0.971234
H	2.845435	-10.456108	0.791297
C	4.888406	-10.374318	0.041492
H	5.328576	-10.723238	0.991002
H	5.468186	-10.848101	-0.767211
C	4.975551	-8.890729	-0.056307
H	4.635725	-8.282523	0.786785
H	4.951793	-8.404398	-1.034755

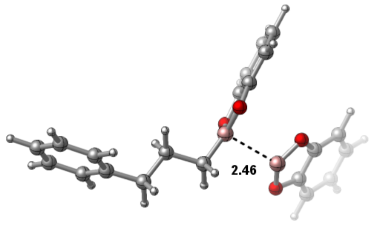

Zero-point correction= 0.491251 (Hartree/Particle)
Thermal correction to Energy= 0.522328
Thermal correction to Enthalpy= 0.523273
Thermal correction to Gibbs Free Energy= 0.422348
Sum of electronic and zero-point Energies= -1448.786729
Sum of electronic and thermal Energies= -1448.755652
Sum of electronic and thermal Enthalpies= -1448.754708
Sum of electronic and thermal Free Energies= -1448.855633

^{1}I'

E(scf) = -1099.97702729 a.u.

ν_{min} = 8.8010 cm^{-1}

C	7.797544	-6.296607	-0.977277
C	6.452619	-6.456746	-1.370206
C	5.925801	-5.743770	-2.439415
C	6.781953	-4.851740	-3.119698
C	8.114808	-4.692927	-2.729017
C	8.645047	-5.420464	-1.642138
H	4.882916	-5.875986	-2.736192
H	6.392614	-4.277507	-3.964404
H	8.759217	-3.995343	-3.270237
H	9.684797	-5.305518	-1.327711
C	6.878706	-11.184376	2.240229
C	7.020897	-11.574086	0.904003
C	6.865217	-12.105809	3.278019
C	7.155906	-12.906418	0.538551
C	7.001992	-13.458393	2.920659
H	6.753478	-11.788765	4.316320
C	7.144043	-13.849856	1.580498
H	7.265747	-13.198899	-0.507099
H	6.997262	-14.219450	3.704446
H	7.247918	-14.910377	1.339508
O	8.076281	-7.110927	0.071378
O	5.840545	-7.375888	-0.584121
O	6.999233	-10.453693	0.112052
O	6.765496	-9.817876	2.289399
B	6.787497	-7.703611	0.490958
B	6.844185	-9.359506	0.967454
C	6.763371	-6.701948	2.887187
O	6.248700	-6.858088	1.731500
N	6.020629	-6.145239	3.839173
C	6.519863	-5.948180	5.202682
H	7.066808	-6.829812	5.559284
H	7.174254	-5.064157	5.262043
H	5.660517	-5.791335	5.865601
C	4.661421	-5.671158	3.571922
H	4.445135	-5.731760	2.501615
H	3.935756	-6.285385	4.127473
H	4.571318	-4.626862	3.906136
C	8.170393	-7.129608	3.196349
H	8.691433	-6.383454	3.810465
H	8.141898	-8.081189	3.749406
H	8.721718	-7.276531	2.263423

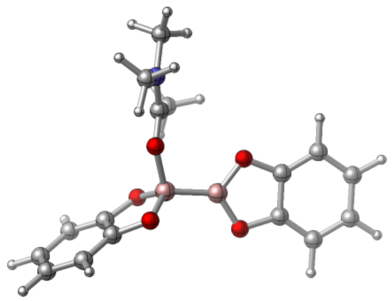

Zero-point correction= 0.319282 (Hartree/Particle)

Thermal correction to Energy= 0.340713
Thermal correction to Enthalpy= 0.341657
Thermal correction to Gibbs Free Energy= 0.265443
Sum of electronic and zero-point Energies= -1099.657745
Sum of electronic and thermal Energies= -1099.636315
Sum of electronic and thermal Enthalpies= -1099.635370
Sum of electronic and thermal Free Energies= -1099.711584

^{3}I'

E(scf) = -1099.87612494 a.u.

ν_{min} = 23.6619 cm^{-1}

C	4.233378	-6.068734	0.776019	O	5.569964	-7.627214	-0.161557
C	5.292275	-6.289312	-0.116700	O	5.840639	-10.697231	0.856710
C	5.897878	-5.246835	-0.805071	O	4.058434	-10.338625	2.365057
C	5.402449	-3.952025	-0.563501	B	4.605408	-8.257778	0.657592
C	4.345580	-3.731288	0.330771	C	3.122826	-9.403129	-0.814915
C	3.737332	-4.795654	1.021654	O	3.566572	-10.464974	-0.036231
H	6.719486	-5.431495	-1.499532	N	3.547702	-9.452395	-2.142681
H	5.851130	-3.103799	-1.086019	C	3.137552	-8.374986	-3.029468
H	3.983447	-2.713553	0.494854	H	2.048880	-8.233757	-3.007417
H	2.912169	-4.636493	1.718237	H	3.619905	-7.410155	-2.770202
C	5.026915	-10.989150	2.946159	H	3.418952	-8.629284	-4.061495
C	6.117033	-11.209569	2.022203	C	4.881559	-9.974173	-2.414553
C	5.095225	-11.451990	4.273696	H	5.058331	-10.883543	-1.829041
C	7.279993	-11.895344	2.421301	H	4.961621	-10.221767	-3.483390
C	6.246049	-12.129930	4.644213	H	5.670220	-9.240875	-2.159847
H	4.268642	-11.279031	4.963799	B	4.489014	-9.968537	0.940229
C	7.323970	-12.348761	3.730190	C	1.728877	-8.937702	-0.501365
H	8.097299	-12.055462	1.717175	H	1.524424	-7.927959	-0.883663
H	6.340864	-12.510691	5.663396	H	0.964767	-9.626515	-0.917042
H	8.205260	-12.889020	4.082572	H	1.605169	-8.911804	0.589726
O	3.832204	-7.266394	1.301094				

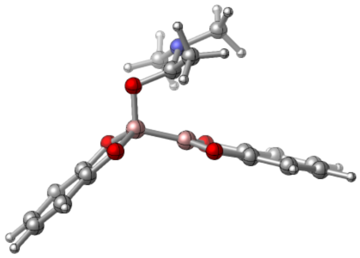

Zero-point correction= 0.316355 (Hartree/Particle)
Thermal correction to Energy= 0.337834
Thermal correction to Enthalpy= 0.338778

Thermal correction to Gibbs Free Energy= 0.263194
Sum of electronic and zero-point Energies= -1099.559770
Sum of electronic and thermal Energies= -1099.538291
Sum of electronic and thermal Enthalpies= -1099.537347
Sum of electronic and thermal Free Energies= -1099.612931

Bcat·

E(scf) = -406.068233178 a.u.

ν_{min} = 235.0290 cm^{-1}

C	5.716644	-5.602674	0.563164
C	4.318427	-5.605736	0.557897
C	3.580179	-4.528505	0.092064
C	4.314478	-3.426363	-0.377628
C	5.718101	-3.423297	-0.372357
C	6.453658	-4.522227	0.102860
H	2.489241	-4.543159	0.093046
H	3.778171	-2.552916	-0.755215
H	6.253410	-2.547515	-0.745932
H	7.544611	-4.532121	0.112020
O	6.161783	-6.808789	1.082185
O	3.874676	-6.813802	1.073560
B	5.018634	-7.511460	1.377664

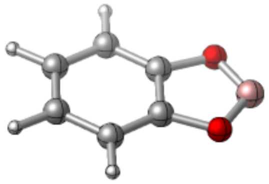

Zero-point correction= 0.092005 (Hartree/Particle)
Thermal correction to Energy= 0.097826
Thermal correction to Enthalpy= 0.098770
Thermal correction to Gibbs Free Energy= 0.061292
Sum of electronic and zero-point Energies= -405.976228
Sum of electronic and thermal Energies= -405.970408
Sum of electronic and thermal Enthalpies= -405.969463
Sum of electronic and thermal Free Energies= -406.006941

^{1}I

E(scf) = -3204.10249857 a.u.

ν_{min} = 4.1129 cm^{-1}

C	7.519617	-11.006893	-1.730894	C	7.689859	-12.063817	-2.615255
C	7.279394	-9.696377	-2.192453	H	7.019084	-8.394401	-3.894961
C	7.213052	-9.412078	-3.549124	H	7.330458	-10.285984	-5.527588
C	7.386380	-10.481106	-4.453535	H	7.738947	-12.596136	-4.714008
C	7.619029	-11.781267	-3.995816	H	7.859371	-13.076289	-2.243729

C	4.821359	-8.386948	2.691473
C	5.070770	-9.670146	3.193217
C	3.885864	-7.541709	3.270874
C	4.393225	-10.170288	4.296615
C	3.192689	-8.036748	4.389662
H	3.695790	-6.546842	2.864760
C	3.440471	-9.323308	4.891189
H	4.592859	-11.173453	4.677801
H	2.443177	-7.406500	4.874179
H	2.881529	-9.677706	5.760540
O	7.503682	-11.037179	-0.375882
O	7.111969	-8.855403	-1.143646
O	6.029272	-10.269146	2.420299
O	5.618334	-8.176875	1.600346
B	7.405664	-9.638597	0.075962
B	6.328553	-9.365936	1.388523
C	9.402336	-8.156447	0.816342
O	8.908654	-9.286521	0.494531
N	10.688080	-8.102127	1.150848
C	11.330228	-6.860992	1.591799
H	11.599652	-6.226553	0.732470
H	10.682562	-6.295330	2.272861
H	12.246660	-7.120917	2.135394
C	11.547009	-9.285707	1.078259
H	11.028193	-10.096636	0.559020
H	12.465732	-9.028178	0.531094
H	11.818831	-9.616075	2.093030
C	8.572800	-6.903116	0.845534
H	8.319248	-6.657940	1.889581
H	9.119172	-6.051685	0.418787
H	7.644894	-7.053085	0.288758
C	4.834911	-12.714052	-0.212818
C	4.842078	-13.528085	-1.354702
C	5.167470	-13.214251	1.037853
C	5.174616	-14.873618	-1.283642
C	5.509703	-14.574939	1.118575
H	5.185809	-12.561782	1.911456
C	5.511444	-15.391643	-0.021244
H	5.177508	-15.499979	-2.177862
H	5.783016	-15.004257	2.085243
H	5.782020	-16.445734	0.073689
N	4.234333	-11.422714	-2.001417
C	4.453873	-12.700846	-2.534987
H	3.964208	-6.145834	-0.547546
H	4.727303	-7.732648	-0.849378
O	4.318420	-13.013189	-3.696848
C	4.433724	-11.339543	-0.622270
O	4.266979	-10.333505	0.035321
O	3.990350	-10.344523	-2.776599
Si	2.360606	-9.637526	-2.831576
Si	2.273311	-7.813294	-1.322687
Si	0.751973	-11.304325	-2.342843
Si	2.400759	-8.964546	-5.095521
C	3.709736	-7.605640	-5.288823
H	4.688939	-7.964044	-4.931273
H	3.815360	-7.312547	-6.347443
H	3.443131	-6.707416	-4.708487
C	2.909403	-10.470480	-6.126688
H	2.098385	-11.215545	-6.160345
H	3.152579	-10.175918	-7.161767
H	3.790144	-10.956798	-5.677921
C	0.703568	-8.323916	-5.641125
H	0.381654	-7.470065	-5.023498
H	0.734356	-7.993653	-6.693500
H	-0.059570	-9.114750	-5.553687
C	0.724920	-12.640379	-3.684911
H	0.029224	-13.451351	-3.409282
H	0.394956	-12.217514	-4.647687
H	1.727575	-13.073353	-3.828767
C	1.085754	-12.110779	-0.657542
H	1.944764	-12.798419	-0.701987
H	1.292981	-11.355921	0.116802
H	0.204959	-12.696574	-0.343354
C	-0.919425	-10.408406	-2.286010
H	-1.136608	-9.906116	-3.242797
H	-1.730869	-11.128666	-2.085266
H	-0.936724	-9.646103	-1.489979
C	1.667374	-8.317964	0.399385
H	2.356739	-9.043902	0.853530
H	1.614712	-7.434389	1.057917
H	0.661837	-8.767153	0.350832
C	1.031238	-6.571563	-2.053020
H	0.037320	-7.028730	-2.190919
H	0.915087	-5.704906	-1.379480
H	1.370638	-6.196856	-3.032530
C	3.984601	-7.015105	-1.227545
H	4.307470	-6.666644	-2.222327

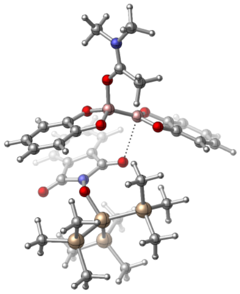

Zero-point correction= 0.764180 (Hartree/Particle)
Thermal correction to Energy= 0.822964
Thermal correction to Enthalpy= 0.823909
Thermal correction to Gibbs Free Energy= 0.665003
Sum of electronic and zero-point Energies= -3203.338319
Sum of electronic and thermal Energies= -3203.279534
Sum of electronic and thermal Enthalpies= -3203.278590
Sum of electronic and thermal Free Energies= -3203.437496

^{3}I

E(scf) = -3204.06864355 a.u.

ν_{min} = 10.4211 cm^{-1}

C	7.404107	-11.274492	-1.748355	B	5.338556	-9.999560	0.827382
C	6.955273	-10.065294	-2.310889	C	7.992569	-7.887452	1.257134
C	6.669038	-9.951165	-3.664719	O	7.931062	-9.130751	0.899920
C	6.849127	-11.098772	-4.454681	N	8.534457	-7.645405	2.435259
C	7.291574	-12.306112	-3.894312	C	8.587005	-6.328042	3.071913
C	7.574538	-12.415363	-2.522196	H	9.624464	-5.962457	3.096475
H	6.303406	-9.012017	-4.082269	H	7.951114	-5.603164	2.558612
H	6.625384	-11.051494	-5.522991	H	8.218117	-6.435584	4.101542
H	7.404338	-13.185523	-4.532614	C	9.055730	-8.742715	3.261768
H	7.894560	-13.355528	-2.070657	H	9.390585	-9.572637	2.632446
C	5.241021	-8.687418	2.654782	H	9.900698	-8.360305	3.848282
C	5.554942	-9.996045	3.070996	H	8.267375	-9.094137	3.943879
C	5.208788	-7.626035	3.550538	C	7.470828	-6.819365	0.352402
C	5.824218	-10.287595	4.403314	H	6.371521	-6.869732	0.358900
C	5.487071	-7.913934	4.899796	H	7.802675	-5.815672	0.627858
H	4.979403	-6.614278	3.211250	H	7.792613	-7.048613	-0.670150
C	5.783296	-9.218800	5.317981	C	4.729162	-12.914211	-0.602576
H	6.066290	-11.304967	4.715785	C	4.421086	-13.658148	-1.789183
H	5.470502	-7.103630	5.632298	C	5.264835	-13.578000	0.521286
H	5.994472	-9.411370	6.372371	C	4.649406	-15.035242	-1.856665
O	7.576975	-11.126352	-0.407692	C	5.487339	-14.948601	0.434794
O	6.835984	-9.122381	-1.339142	H	5.507162	-13.020565	1.424632
O	5.553361	-10.831405	2.005158	C	5.185930	-15.678867	-0.741527
O	5.032736	-8.665542	1.315216	H	4.412730	-15.584338	-2.771396
B	7.086257	-9.808307	-0.090332	H	5.906313	-15.476291	1.295595

H	5.376917	-16.754291	-0.771518	H	-0.553355	-13.366731	-2.891901
N	3.897280	-11.499919	-2.153633	H	-0.380029	-12.122200	-4.161119
C	3.894755	-12.752168	-2.804253	H	1.054069	-13.030671	-3.613396
O	3.510300	-12.940986	-3.959773	C	1.017945	-12.114021	-0.359867
C	4.400213	-11.554810	-0.872773	H	1.851863	-12.804088	-0.561565
O	4.388201	-10.451854	-0.151137	H	1.358703	-11.377379	0.384412
O	3.615578	-10.342909	-2.791616	H	0.192075	-12.697496	0.082138
Si	1.987589	-9.641071	-2.679999	C	-1.172562	-10.308613	-1.602157
Si	2.133896	-7.811249	-1.180339	H	-1.522024	-9.776284	-2.502074
Si	0.428739	-11.267348	-1.951283	H	-1.969779	-11.002691	-1.285286
Si	1.714271	-8.978076	-4.930258	H	-1.031072	-9.564938	-0.801177
C	3.080354	-7.728033	-5.344855	C	1.800391	-8.338416	0.608211
H	4.073031	-8.162923	-5.142803	H	2.497571	-9.131739	0.910576
H	3.039889	-7.444463	-6.410567	H	1.938195	-7.482033	1.290312
H	2.981604	-6.811224	-4.741250	H	0.768305	-8.707233	0.723925
C	1.911489	-10.506047	-6.032454	C	0.816211	-6.538407	-1.686347
H	1.009868	-11.137803	-5.983975	H	-0.192816	-6.982774	-1.689671
H	2.072726	-10.213131	-7.083934	H	0.811522	-5.691532	-0.978506
H	2.761329	-11.117566	-5.690276	H	1.015164	-6.137756	-2.693647
C	0.009496	-8.193328	-5.195587	C	3.853410	-7.039415	-1.354164
H	-0.133385	-7.312465	-4.549641	H	3.990639	-6.626637	-2.367014
H	-0.108637	-7.872236	-6.244717	H	3.986012	-6.221385	-0.625586
H	-0.791934	-8.915667	-4.968393	H	4.634838	-7.790909	-1.176158
C	0.108152	-12.574348	-3.282395				

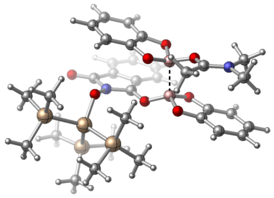

Zero-point correction= 0.764888 (Hartree/Particle)
Thermal correction to Energy= 0.822293
Thermal correction to Enthalpy= 0.823238
Thermal correction to Gibbs Free Energy= 0.671138
Sum of electronic and zero-point Energies= -3203.303755
Sum of electronic and thermal Energies= -3203.246350
Sum of electronic and thermal Enthalpies= -3203.245406
Sum of electronic and thermal Free Energies= -3203.397505

^{3}TS$_{I\text{-}II/A}$
E(scf) = -3204.05507394 a.u.
ν_{min} = -700.3848 cm^{-1}

C	7.489974	-11.276068	-1.808989	C	6.629812	-10.031788	-3.722722
C	7.027707	-10.087707	-2.396254	C	6.707604	-11.227098	-4.459659

C	7.163391	-12.414965	-3.873524	H	6.009019	-15.475882	1.231127
C	7.565032	-12.460915	-2.526048	H	5.345524	-16.766878	-0.788671
H	6.255674	-9.104986	-4.159009	N	3.932187	-11.496194	-2.180028
H	6.388775	-11.229970	-5.504408	C	3.870210	-12.758536	-2.806286
H	7.192587	-13.331110	-4.467661	O	3.438299	-12.954697	-3.941609
H	7.897323	-13.385514	-2.052161	C	4.475809	-11.548748	-0.918403
C	5.273649	-8.766810	2.720507	O	4.569066	-10.402782	-0.260582
C	5.547396	-10.090437	3.093211	O	3.641866	-10.343226	-2.816973
C	5.267390	-7.734352	3.646120	Si	2.012030	-9.637615	-2.691467
C	5.820636	-10.442088	4.406864	Si	2.207032	-7.810567	-1.193493
C	5.541042	-8.078139	4.982570	Si	0.447365	-11.246720	-1.933729
H	5.060614	-6.707261	3.342727	Si	1.717767	-8.979673	-4.940143
C	5.810870	-9.402520	5.354897	C	3.099228	-7.755365	-5.377553
H	6.031503	-11.476390	4.683016	H	4.087033	-8.208260	-5.192581
H	5.545917	-7.293772	5.742746	H	3.045641	-7.470798	-6.442380
H	6.021260	-9.634749	6.401302	H	3.027299	-6.837169	-4.772258
O	7.777937	-11.054576	-0.488564	C	1.869817	-10.516735	-6.036437
O	7.018030	-9.089699	-1.458288	H	0.954550	-11.127422	-5.976422
O	5.485699	-10.893688	1.983739	H	2.027634	-10.232426	-7.090742
O	5.052469	-8.709659	1.370738	H	2.708880	-11.145953	-5.699831
B	7.420198	-9.705315	-0.249675	C	0.023444	-8.164993	-5.177687
B	5.213481	-10.028944	0.908471	H	-0.090922	-7.279379	-4.532482
C	8.009330	-7.769546	1.203178	H	-0.107130	-7.845146	-6.225694
O	7.961278	-9.040128	0.831216	H	-0.787254	-8.871297	-4.933773
N	8.274192	-7.555032	2.515838	C	0.074911	-12.546822	-3.257207
C	8.269739	-6.213725	3.077943	H	-0.601257	-13.320119	-2.853776
H	9.231412	-5.694306	2.910364	H	-0.415065	-12.084266	-4.129407
H	7.458052	-5.607120	2.659053	H	1.003236	-13.028791	-3.600559
H	8.101111	-6.290379	4.160741	C	1.055395	-12.105018	-0.355604
C	8.850797	-8.589070	3.364806	H	1.891107	-12.789582	-0.568428
H	8.765119	-9.569719	2.887999	H	1.395063	-11.374564	0.395303
H	9.917098	-8.377013	3.563664	H	0.235622	-12.697478	0.085814
H	8.310616	-8.617170	4.322444	C	-1.130897	-10.262437	-1.553463
C	7.544706	-6.681309	0.295293	H	-1.489754	-9.726565	-2.447577
H	6.463219	-6.490363	0.432396	H	-1.931824	-10.944664	-1.220662
H	8.088137	-5.744717	0.475209	H	-0.963723	-9.519837	-0.756719
H	7.691473	-6.976208	-0.749011	C	1.857599	-8.337023	0.593875
C	4.784755	-12.909578	-0.631450	H	2.497861	-9.179043	0.890619
C	4.407709	-13.661911	-1.793602	H	2.054531	-7.500634	1.285720
C	5.366226	-13.574533	0.469093	H	0.805047	-8.640715	0.712488
C	4.602639	-15.043032	-1.856587	C	0.925889	-6.499322	-1.690387
C	5.556024	-14.950676	0.386424	H	-0.095047	-6.915370	-1.692560
H	5.666047	-13.019741	1.355441	H	0.947266	-5.655059	-0.979853
C	5.179521	-15.687401	-0.761873	H	1.133969	-6.101616	-2.696977
H	4.311842	-15.594229	-2.754071	C	3.948571	-7.091024	-1.367413

H	4.105202	-6.707526	-2.388993	H	4.712497	-7.853952	-1.163920
H	4.094949	-6.257769	-0.659087				

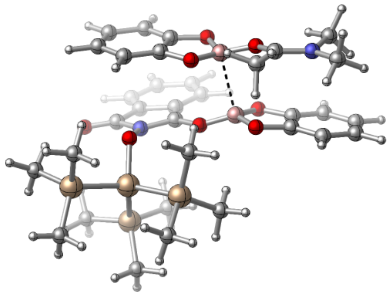

Zero-point correction= 0.762611 (Hartree/Particle)
Thermal correction to Energy= 0.820286
Thermal correction to Enthalpy= 0.821230
Thermal correction to Gibbs Free Energy= 0.668838
Sum of electronic and zero-point Energies= -3203.292463
Sum of electronic and thermal Energies= -3203.234788
Sum of electronic and thermal Enthalpies= -3203.233844
Sum of electronic and thermal Free Energies= -3203.386236

^{2}A

E(scf) = -2510.26664578 a.u.

ν_{min} = 9.9558 cm^{-1}

C	5.509132	-11.079743	2.664685	H	1.655663	-12.360420	-3.340963
C	6.575119	-10.576203	3.479535	C	2.021364	-15.297626	-1.571642
C	4.500006	-11.882664	3.236644	H	3.289397	-15.580845	0.194087
C	6.641105	-10.878587	4.841011	H	0.863707	-14.751362	-3.310929
C	4.582625	-12.171482	4.595460	H	1.663346	-16.329486	-1.579145
H	3.683684	-12.274878	2.629867	O	5.017131	-10.602304	0.229980
C	5.641236	-11.680170	5.395346	B	4.275690	-11.627282	-0.272743
H	7.458416	-10.486419	5.450741	O	3.476175	-11.449429	-1.383595
H	3.811323	-12.793514	5.056070	O	4.221594	-12.914437	0.223394
H	5.671649	-11.930135	6.458269	Si	7.006486	-7.489272	-0.051588
N	6.852080	-9.781588	1.384947	Si	4.945547	-6.830267	0.917221
C	7.456362	-9.740854	2.662134	Si	6.897795	-7.547511	-2.410420
O	8.477335	-9.109615	2.925073	Si	8.811900	-6.212285	0.790116
C	5.729803	-10.567718	1.358256	C	6.132497	-5.931858	-3.039892
O	7.370348	-9.204532	0.278919	H	6.724656	-5.059286	-2.719303
C	2.893970	-12.682390	-1.596055	H	6.091920	-5.930779	-4.142505
C	3.346177	-13.576158	-0.617546	H	5.105184	-5.804431	-2.661514
C	2.000673	-13.065678	-2.583481	C	8.634625	-7.760465	-3.138669
C	2.928793	-14.896115	-0.574875	H	8.587210	-7.833129	-4.238775
C	1.568527	-14.403678	-2.552402	H	9.282919	-6.907491	-2.879860

H	9.110106	-8.677652	-2.754273	H	10.562793	-7.499838	-0.468899
C	5.821469	-9.029054	-2.892914	H	11.267171	-6.698641	0.966513
H	6.243084	-9.956411	-2.474161	C	4.924467	-4.940040	0.740770
H	4.797879	-8.925023	-2.500973	H	5.016269	-4.634097	-0.313972
H	5.766696	-9.133203	-3.989974	H	3.974102	-4.537397	1.130895
C	8.914162	-4.626123	-0.249775	H	5.748174	-4.472279	1.303542
H	9.089429	-4.855845	-1.313485	C	3.450909	-7.549140	0.003372
H	7.981820	-4.042048	-0.178044	H	3.445112	-7.230752	-1.051747
H	9.742749	-3.987778	0.102067	H	3.457381	-8.648813	0.026726
C	8.571962	-5.753575	2.611298	H	2.516430	-7.195335	0.471576
H	8.489414	-6.670498	3.214456	C	4.858053	-7.288822	2.754022
H	9.433675	-5.167417	2.974702	H	3.994106	-6.786581	3.222070
H	7.662498	-5.149488	2.760609	H	4.735825	-8.373332	2.898553
C	10.385456	-7.243727	0.588286	H	5.769569	-6.974284	3.286314
H	10.272155	-8.176558	1.162202				

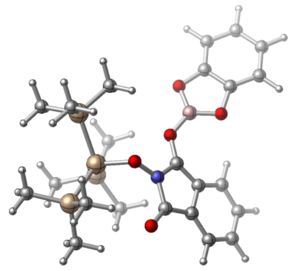

Zero-point correction= 0.538164 (Hartree/Particle)
Thermal correction to Energy= 0.580537
Thermal correction to Enthalpy= 0.581481
Thermal correction to Gibbs Free Energy= 0.460484
Sum of electronic and zero-point Energies= -2509.728482
Sum of electronic and thermal Energies= -2509.686109
Sum of electronic and thermal Enthalpies= -2509.685165
Sum of electronic and thermal Free Energies= -2509.806161

^{2}A'

E(scf) = -918.309170589 a.u.
ν_{min} = 25.1859 cm^{-1}

C	4.169396	-10.878562	2.724652	H	3.819367	-12.733285	5.529946
C	5.555670	-10.677002	2.769789	H	6.268865	-12.391845	5.595256
C	3.514109	-11.610068	3.707885	N	4.687685	-9.530063	0.874819
C	6.338888	-11.205923	3.782400	C	5.894861	-9.818020	1.581821
C	4.301679	-12.150862	4.741824	O	6.985799	-9.419250	1.250012
H	2.436728	-11.774397	3.691886	C	3.737833	-10.131302	1.507272
C	5.687435	-11.957051	4.779097	C	-0.535352	-11.785371	0.969794
H	7.418070	-11.040541	3.806265	C	0.370131	-12.827785	1.199965

C	-1.895653	-12.010572	0.833222	H	-3.385778	-13.573104	0.839296
C	-0.035810	-14.147717	1.307313	H	-1.783249	-15.415028	1.248000
C	-2.322070	-13.346329	0.939614	O	2.472559	-10.009818	1.100857
H	-2.591869	-11.190157	0.653556	B	1.474454	-10.943606	1.115055
C	-1.414292	-14.389904	1.171117	O	0.157906	-10.591756	0.913632
H	0.681533	-14.949548	1.487720	O	1.645171	-12.300525	1.289878

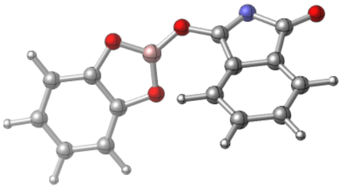

Zero-point correction= 0.199837 (Hartree/Particle)
Thermal correction to Energy= 0.214108
Thermal correction to Enthalpy= 0.215052
Thermal correction to Gibbs Free Energy= 0.156486
Sum of electronic and zero-point Energies= -918.109333
Sum of electronic and thermal Energies= -918.095063
Sum of electronic and thermal Enthalpies= -918.094119
Sum of electronic and thermal Free Energies= -918.152685

^{2}A''

E(scf) = -1612.10673538 a.u.

ν_{min} = 11.1060 cm^{-1}

C	7.678617	-11.660854	-1.705824	H	7.277361	-9.591917	6.410070
C	7.048220	-10.546156	-2.288503	O	7.993031	-11.389773	-0.411291
C	6.585830	-10.571822	-3.597936	O	6.960695	-9.539702	-1.377246
C	6.778208	-11.762328	-4.320408	O	6.254436	-11.139547	2.186081
C	7.406176	-12.874136	-3.739121	O	5.531533	-9.019842	1.540962
C	7.867109	-12.841850	-2.412311	B	7.430311	-10.095165	-0.127892
H	6.087035	-9.704514	-4.033753	B	5.833305	-10.351580	1.043168
H	6.424586	-11.822774	-5.352309	C	8.322300	-8.027378	1.007698
H	7.529858	-13.788765	-4.323556	O	8.326208	-9.283118	0.696614
H	8.331226	-13.708462	-1.939537	N	9.012156	-7.685486	2.078311
C	5.934832	-8.997383	2.837782	C	9.063157	-6.323235	2.609630
C	6.375569	-10.276964	3.225361	H	10.074781	-5.910211	2.479318
C	5.979536	-7.917853	3.711570	H	8.335342	-5.669179	2.123257
C	6.858899	-10.523337	4.504758	H	8.827810	-6.365079	3.682557
C	6.472618	-8.159016	5.007280	C	9.730243	-8.684997	2.878379
H	5.644336	-6.927129	3.399298	H	9.976729	-9.560024	2.270195
C	6.899408	-9.436347	5.397060	H	10.652699	-8.226605	3.257818
H	7.198645	-11.519658	4.792808	H	9.100452	-8.991681	3.726955
H	6.521670	-7.333140	5.720581	C	7.574803	-7.015807	0.194065

H	6.690896	-6.676082	0.754733	C	6.073706	-15.925517	-0.545062
H	8.208021	-6.146234	-0.026638	H	4.670436	-16.041489	-2.218587
H	7.238275	-7.469299	-0.741226	H	7.362878	-15.525869	1.137909
C	5.260665	-13.281560	-0.271664	H	6.410266	-16.960709	-0.639881
C	4.716757	-14.101550	-1.268940	N	3.726615	-11.961640	-1.467951
C	6.211864	-13.772920	0.615824	C	3.732463	-13.252429	-2.028429
C	5.103022	-15.421675	-1.430032	O	3.052748	-13.602911	-2.972033
C	6.612387	-15.112980	0.459537	C	4.592420	-11.954163	-0.488430
H	6.642095	-13.147206	1.394242	O	4.787717	-10.860073	0.195860

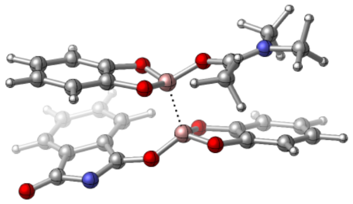

Zero-point correction= 0.425375 (Hartree/Particle)
Thermal correction to Energy= 0.455427
Thermal correction to Enthalpy= 0.456371
Thermal correction to Gibbs Free Energy= 0.361874
Sum of electronic and zero-point Energies= -1611.681360
Sum of electronic and thermal Energies= -1611.651309
Sum of electronic and thermal Enthalpies= -1611.650364
Sum of electronic and thermal Free Energies= -1611.744862

^{3}TS2

E(scf) = -2510.23646233 a.u.

ν_{min} = -685.3255 cm^{-1}

C	5.682388	-11.080251	3.309237	C	3.989655	-12.313660	-0.919190
C	6.989223	-10.733087	3.723738	C	2.012569	-11.773651	-2.236561
C	5.037789	-12.201309	3.837612	C	4.353711	-13.361739	-1.747844
C	7.688787	-11.495450	4.647155	C	2.369730	-12.836414	-3.085984
C	5.747092	-12.973931	4.769888	H	1.129535	-11.158326	-2.414787
H	4.025205	-12.473093	3.534162	C	3.512750	-13.612423	-2.846968
C	7.049777	-12.633810	5.170749	H	5.250422	-13.952234	-1.554529
H	8.698750	-11.216644	4.955050	H	1.741432	-13.058491	-3.951398
H	5.274747	-13.863185	5.193934	H	3.759726	-14.428891	-3.529186
H	7.568993	-13.259575	5.899595	O	4.106554	-9.986334	1.712826
N	6.196648	-9.103047	2.204258	B	3.861283	-10.763700	0.619081
C	7.359084	-9.488648	2.999591	O	2.756060	-10.563862	-0.184309
O	8.388041	-8.864650	3.016893	O	4.633319	-11.831413	0.203056
C	5.275265	-10.051056	2.376814	Si	6.690809	-7.603005	-0.187369
O	6.707604	-9.162000	0.533491	Si	4.683735	-6.392909	0.182646
C	2.847880	-11.539629	-1.156955	Si	6.784222	-8.267017	-2.463427

Si	8.584300	-6.313754	0.414874	H	9.227831	-4.874196	2.367717
C	6.350074	-6.791275	-3.574982	H	7.463491	-4.817433	2.098787
H	7.043200	-5.951030	-3.407903	C	10.127707	-7.413719	0.463983
H	6.413843	-7.081711	-4.637786	H	10.011904	-8.188591	1.236521
H	5.327091	-6.430722	-3.381161	H	10.293045	-7.908982	-0.506507
C	8.514649	-8.898314	-2.912785	H	11.023258	-6.813256	0.700571
H	8.532973	-9.276883	-3.949319	C	4.996072	-4.611818	-0.404688
H	9.264209	-8.094489	-2.830054	H	5.315861	-4.583980	-1.459091
H	8.818862	-9.717700	-2.241290	H	4.071477	-4.016219	-0.313316
C	5.527283	-9.663957	-2.703481	H	5.776092	-4.121684	0.200111
H	5.797968	-10.523074	-2.070805	C	3.228157	-7.093122	-0.812504
H	4.513754	-9.342798	-2.416156	H	3.437541	-7.067601	-1.894133
H	5.496706	-10.003777	-3.752579	H	3.003894	-8.133939	-0.534960
C	8.834908	-4.977265	-0.914841	H	2.324044	-6.487132	-0.628454
H	9.050530	-5.433166	-1.895316	C	4.212151	-6.356020	2.018167
H	7.943744	-4.339523	-1.026780	H	3.346772	-5.688090	2.171912
H	9.687546	-4.328864	-0.648586	H	3.943759	-7.363765	2.368967
C	8.344507	-5.480072	2.101321	H	5.047630	-5.990297	2.636179
H	8.201362	-6.247166	2.878351				

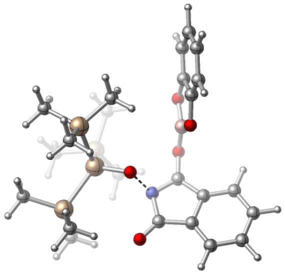

Zero-point correction= 0.535338 (Hartree/Particle)
Thermal correction to Energy= 0.577712
Thermal correction to Enthalpy= 0.578657
Thermal correction to Gibbs Free Energy= 0.457880
Sum of electronic and zero-point Energies= -2509.701124
Sum of electronic and thermal Energies= -2509.658750
Sum of electronic and thermal Enthalpies= -2509.657806
Sum of electronic and thermal Free Energies= -2509.778583

^{3}TS4

E(scf) = -3204.03403058 a.u.

ν_{min} = -805.2526 cm^{-1}

C	7.711772	-11.688992	-1.663376	C	7.084359	-10.590052	-2.275862

C	6.708542	-10.618679	-3.612081	C	6.010849	-15.890422	-0.642690
C	6.980439	-11.795054	-4.329341	H	4.539905	-15.978949	-2.256856
C	7.597305	-12.896420	-3.716102	H	7.345009	-15.512389	1.011564
C	7.971664	-12.861836	-2.362408	H	6.346388	-16.922402	-0.765701
H	6.203745	-9.769273	-4.069968	N	3.544618	-11.969838	-1.309084
H	6.694071	-11.854409	-5.382084	C	3.586002	-13.250740	-1.973143
H	7.783587	-13.802333	-4.297807	O	2.885509	-13.554449	-2.908782
H	8.432499	-13.717367	-1.866915	C	4.534746	-11.935277	-0.393551
C	5.964243	-9.009151	2.904793	O	4.684884	-10.841854	0.324628
C	6.397148	-10.298156	3.271302	O	3.761583	-10.833544	-2.588877
C	6.041079	-7.937733	3.786489	Si	2.384129	-9.827935	-2.752505
C	6.904247	-10.560468	4.538873	Si	2.330501	-8.157361	-1.076444
C	6.556713	-8.195987	5.069999	Si	0.335775	-11.013902	-2.881285
H	5.712743	-6.940695	3.487219	Si	2.848968	-8.870393	-4.872947
C	6.975543	-9.482169	5.439825	C	4.151290	-7.493016	-4.752425
H	7.237038	-11.563391	4.812036	H	5.053065	-7.823910	-4.213357
H	6.630618	-7.377212	5.789379	H	4.454716	-7.162268	-5.760679
H	7.371767	-9.651362	6.443688	H	3.744275	-6.619992	-4.216832
O	7.956896	-11.412734	-0.356023	C	3.491215	-10.249576	-6.006036
O	6.920785	-9.589963	-1.367972	H	2.715911	-11.017707	-6.158918
O	6.238591	-11.149883	2.230369	H	3.785262	-9.852200	-6.992518
O	5.527384	-9.014760	1.621265	H	4.364022	-10.742745	-5.550406
B	7.371543	-10.121061	-0.100657	C	1.271120	-8.120007	-5.618359
B	5.785415	-10.348006	1.101060	H	0.845301	-7.349687	-4.954630
C	8.284304	-8.022697	0.972936	H	1.493161	-7.647614	-6.590818
O	8.272340	-9.287642	0.707873	H	0.500766	-8.890601	-5.781626
N	8.969624	-7.651990	2.038081	C	0.239314	-12.038983	-4.474804
C	9.013382	-6.277245	2.537762	H	-0.710152	-12.600275	-4.519562
H	10.002689	-5.835695	2.343414	H	0.299171	-11.398380	-5.369666
H	8.236480	-5.656597	2.083910	H	1.073266	-12.756693	-4.499577
H	8.839448	-6.304111	3.622465	C	0.065041	-12.159390	-1.393235
C	9.687977	-8.630622	2.862558	H	0.781034	-12.993811	-1.414587
H	9.937707	-9.518769	2.274975	H	0.194044	-11.616562	-0.443106
H	10.609108	-8.161172	3.232068	H	-0.957443	-12.575208	-1.417598
H	9.058952	-8.919970	3.717586	C	-1.072058	-9.733025	-2.889469
C	7.554336	-7.036785	0.116196	H	-0.930051	-8.969073	-3.670564
H	6.579169	-6.816501	0.576807	H	-2.038338	-10.234036	-3.073097
H	8.118889	-6.104308	-0.000194	H	-1.139369	-9.217080	-1.917765
H	7.364046	-7.479472	-0.864951	C	1.799024	-8.877389	0.596080
C	5.208003	-13.228219	-0.296082	H	2.494108	-9.678099	0.892551
C	4.620250	-14.055317	-1.282072	H	1.809302	-8.099526	1.379315
C	6.181211	-13.757112	0.561497	H	0.781324	-9.297902	0.541675
C	5.009028	-15.371623	-1.479575	C	1.104940	-6.796401	-1.591265
C	6.573155	-15.088706	0.363921	H	0.081490	-7.187909	-1.702285
H	6.635220	-13.149104	1.339856	H	1.083004	-5.993295	-0.834197

H	1.399748	-6.345567	-2.553555
C	4.052976	-7.383079	-0.917684
H	4.316000	-6.822868	-1.828807
H	4.090168	-6.689433	-0.060360
H	4.811898	-8.160924	-0.760275

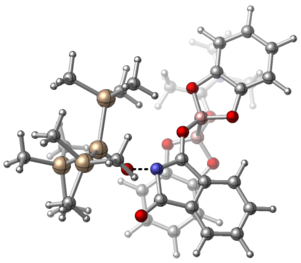

Zero-point correction= 0.761576 (Hartree/Particle)
Thermal correction to Energy= 0.819291
Thermal correction to Enthalpy= 0.820235
Thermal correction to Gibbs Free Energy= 0.667672
Sum of electronic and zero-point Energies= -3203.272455
Sum of electronic and thermal Energies= -3203.214740
Sum of electronic and thermal Enthalpies= -3203.213795
Sum of electronic and thermal Free Energies= -3203.366359

^{3}**2**

E(scf) = -2104.00694652 a.u.

ν_{min} = 10.2884 cm^{-1}

C	3.616394	-10.037351	2.869681
C	4.837461	-10.781129	2.638982
C	2.921768	-10.180006	4.091428
C	5.340085	-11.646985	3.639075
C	3.431014	-11.033273	5.053495
H	1.999637	-9.619436	4.262256
C	4.642980	-11.767368	4.825964
H	6.262402	-12.205523	3.463481
H	2.907911	-11.155309	6.004874
H	5.014688	-12.431778	5.609720
N	4.394949	-9.536235	0.813413
C	5.360239	-10.487249	1.346054
O	6.344207	-10.829275	0.681216
C	3.294684	-9.225748	1.733460
O	2.393648	-8.447673	1.435848
O	4.455094	-8.988414	-0.345325
Si	5.453397	-9.300405	-1.842169
Si	4.689843	-11.411794	-2.654868
Si	7.808478	-9.145598	-1.527684
Si	4.499745	-7.437589	-2.967188
C	2.637281	-7.463983	-2.631404
H	2.451832	-7.538448	-1.547867
H	2.165831	-6.539996	-3.007085
H	2.151398	-8.322628	-3.120222
C	5.230130	-5.853904	-2.226812
H	6.315772	-5.781208	-2.397473
H	4.752565	-4.968398	-2.680096
H	5.052388	-5.819757	-1.139459
C	4.868631	-7.539162	-4.819736
H	4.417316	-8.441056	-5.264342
H	4.454057	-6.658171	-5.338507
H	5.953585	-7.568845	-5.009551
C	8.171184	-8.107905	0.007458
H	9.256514	-7.927613	0.091915
H	7.662980	-7.131538	-0.042275
H	7.831006	-8.637465	0.909826
C	8.646513	-10.834918	-1.452991
H	8.324650	-11.385926	-0.559409
H	8.407908	-11.436423	-2.344849
H	9.741239	-10.698945	-1.420541
C	8.417484	-8.249049	-3.087006
H	7.998301	-7.234538	-3.173311

H	9.517212	-8.161537	-3.052724	H	6.699566	-11.728580	-4.135754
H	8.151926	-8.809349	-3.998621	H	5.271180	-12.595184	-4.762070
C	5.085383	-12.800851	-1.443063	H	5.415114	-10.831161	-4.991759
H	4.519994	-12.682307	-0.506012	C	2.827061	-11.314303	-2.955700
H	4.805709	-13.766364	-1.898944	H	2.589513	-10.589919	-3.750383
H	6.155636	-12.817055	-1.192294	H	2.450528	-12.303281	-3.268774
C	5.611200	-11.657322	-4.290006	H	2.289922	-11.015148	-2.041471

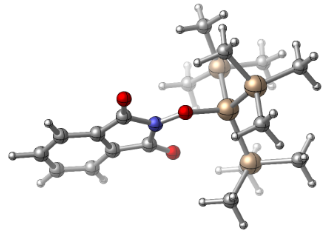

Zero-point correction= 0.440654 (Hartree/Particle)
Thermal correction to Energy= 0.476708
Thermal correction to Enthalpy= 0.477653
Thermal correction to Gibbs Free Energy= 0.371044
Sum of electronic and zero-point Energies= -2103.566293
Sum of electronic and thermal Energies= -2103.530238
Sum of electronic and thermal Enthalpies= -2103.529294
Sum of electronic and thermal Free Energies= -2103.635902

^{3}B

E(scf) = -512.044016499 a.u.
ν_{min} = 27.1600 cm^{-1}

C	3.630448	-10.063606	2.898126
C	4.815609	-10.781794	2.674419
C	2.921329	-10.179232	4.091735
C	5.339017	-11.643895	3.635515
C	3.440227	-11.044291	5.061758
H	2.000297	-9.617648	4.258330
C	4.631871	-11.766056	4.836941
H	6.261281	-12.198833	3.454248
H	2.916571	-11.167071	6.012410
H	5.004921	-12.431681	5.618481
N	4.412086	-9.473671	0.740255
C	5.304275	-10.426582	1.314664
O	6.286577	-10.837122	0.732222
C	3.363491	-9.250458	1.680840
O	2.434068	-8.502397	1.459000

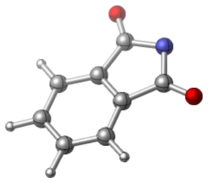

Zero-point correction= 0.101914 (Hartree/Particle)
Thermal correction to Energy= 0.109930
Thermal correction to Enthalpy= 0.110875
Thermal correction to Gibbs Free Energy= 0.066947
Sum of electronic and zero-point Energies= -511.942103
Sum of electronic and thermal Energies= -511.934086
Sum of electronic and thermal Enthalpies= -511.933142
Sum of electronic and thermal Free Energies= -511.977069

Selected NMR

^{1}H NMR of 4-hydroxy-4-methylpentyl 4-(trifluoromethyl)benzoate **(S1f)** ($CDCl_3$, 500 MHz)

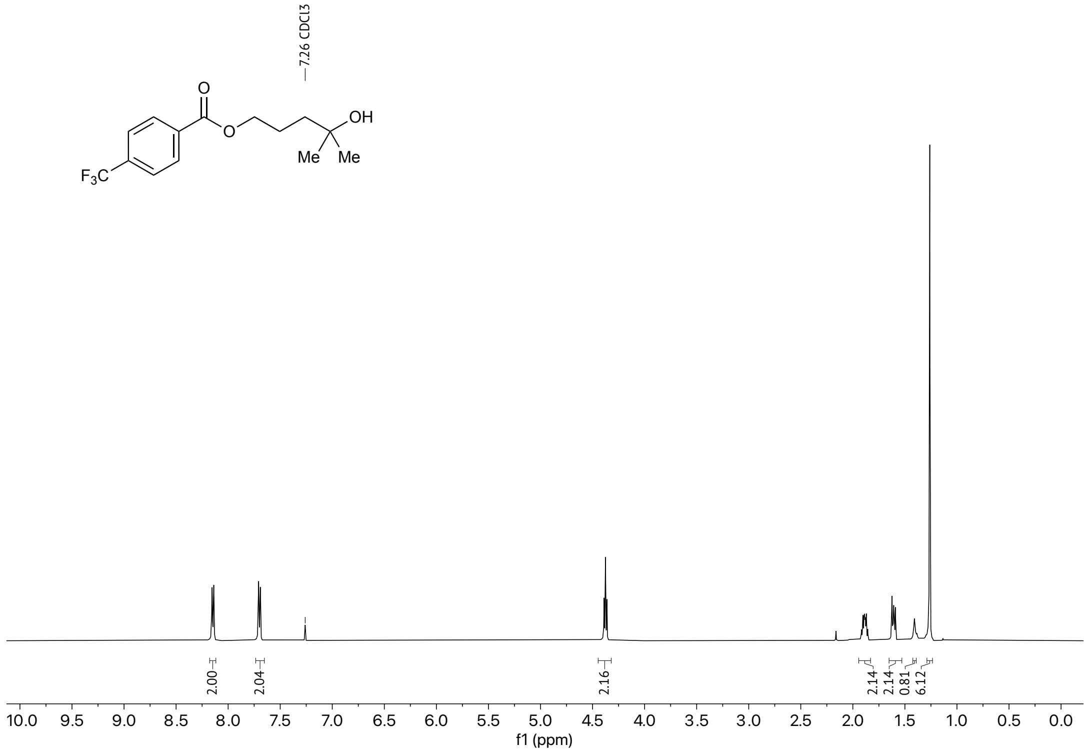

^{13}C NMR of 4-hydroxy-4-methylpentyl 4-(trifluoromethyl)benzoate **(S1f)** ($CDCl_3$, 126 MHz)

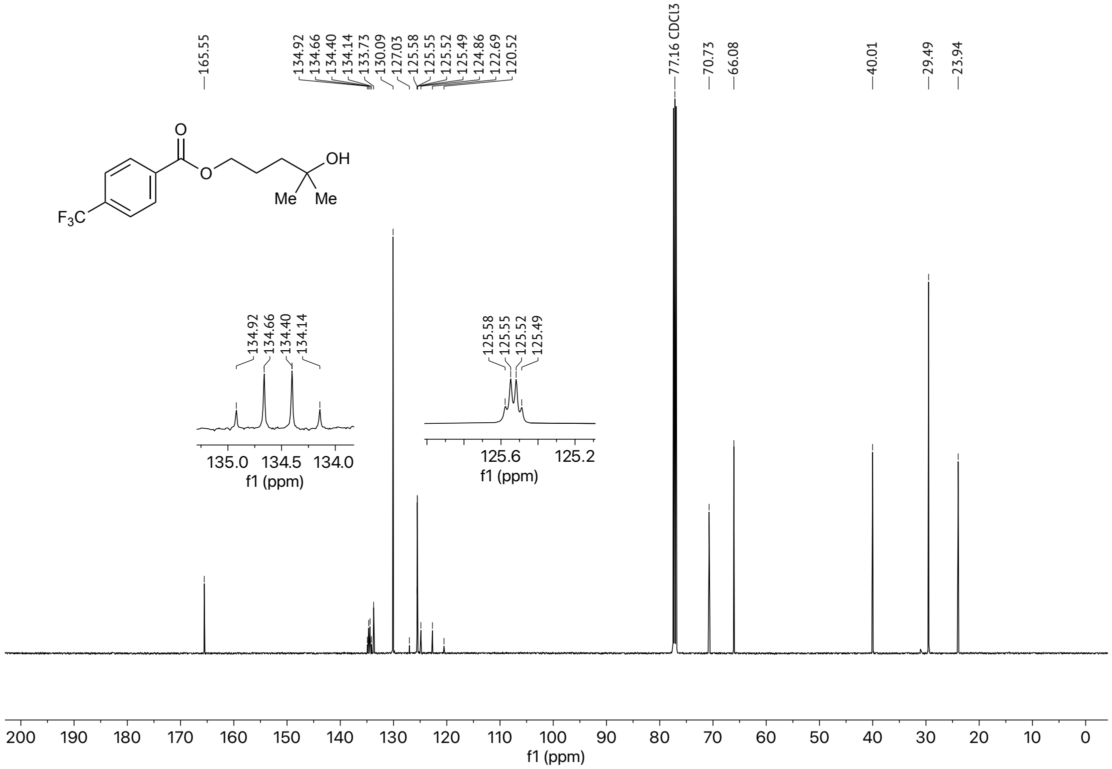

^{19}F NMR of 4-hydroxy-4-methylpentyl 4-(trifluoromethyl)benzoate **(S1f)** ($CDCl_3$, 470 MHz)

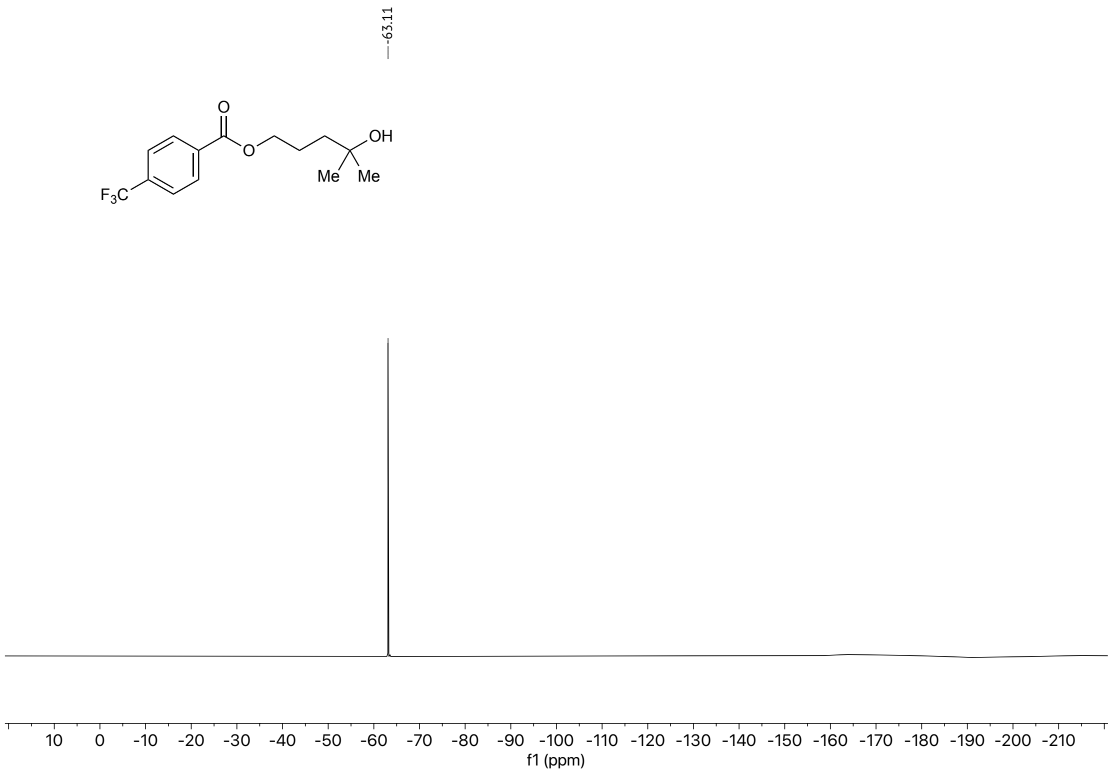

^{1}H NMR **(S1h)** ($CDCl_3$, 500 MHz)

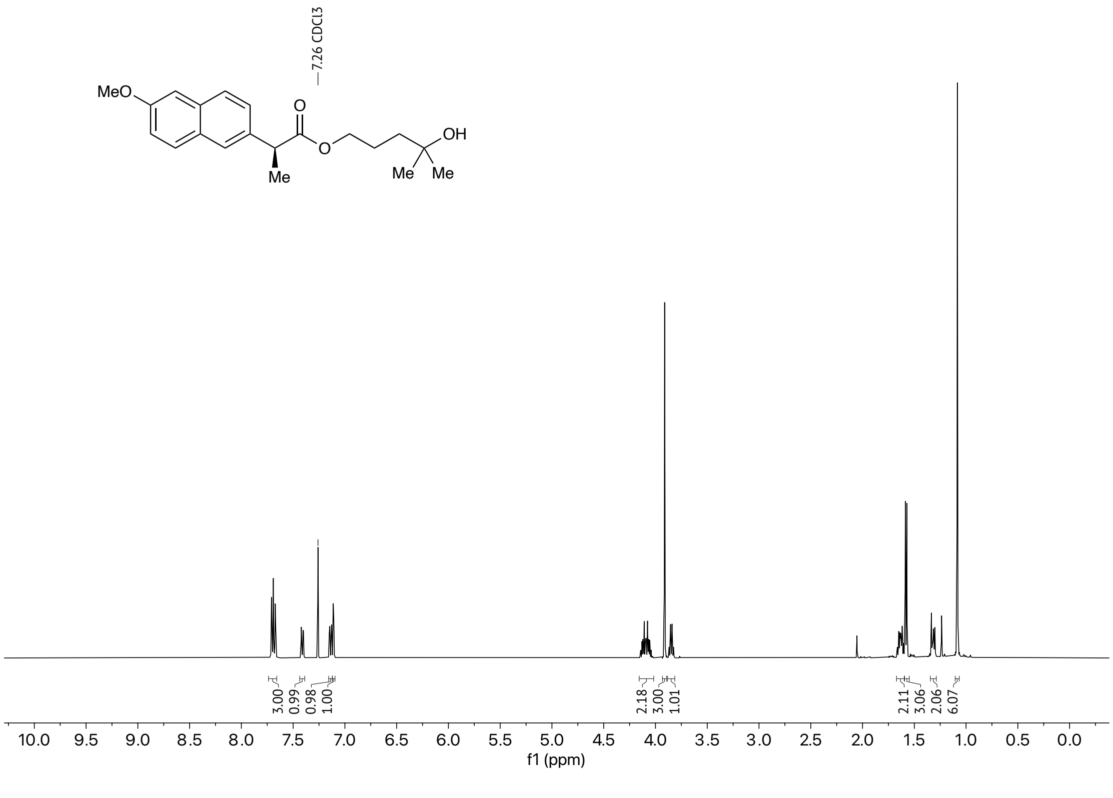

^{13}C NMR of **(S1h)** ($CDCl_3$, 126 MHz)

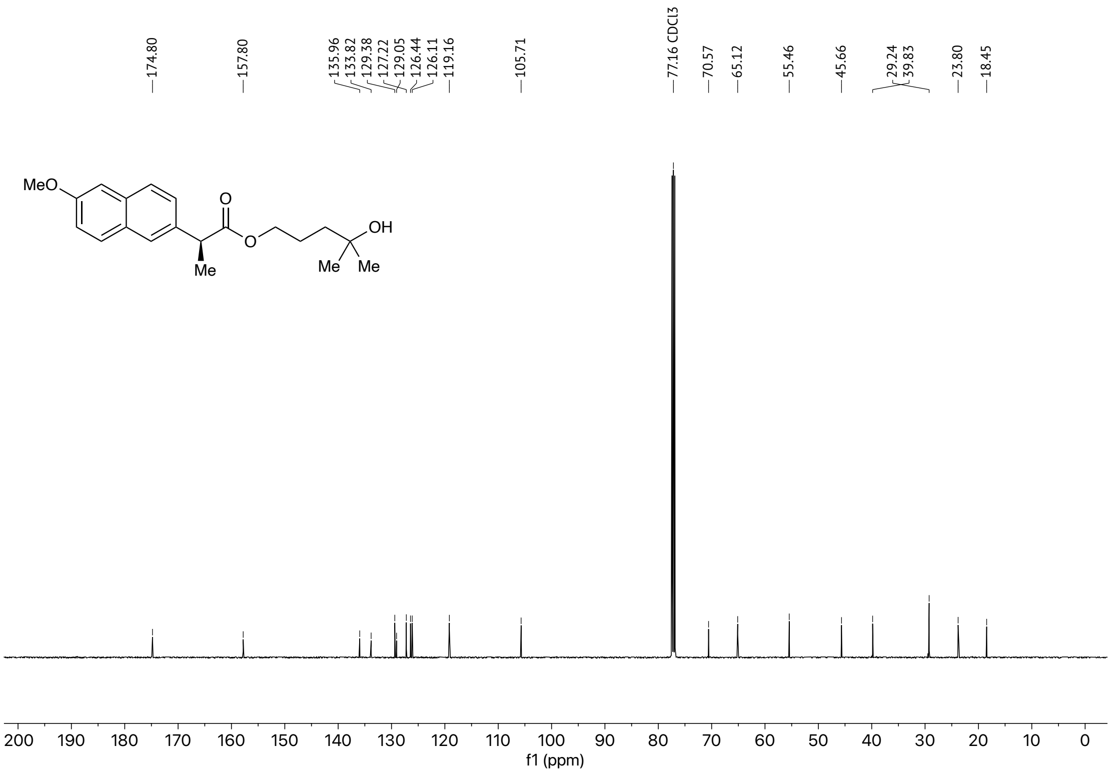

^{1}H NMR of 2-(3-bromopropyl)-4,5-diphenyloxazole (**S2h)** ($CDCl_3$, 500 MHz)

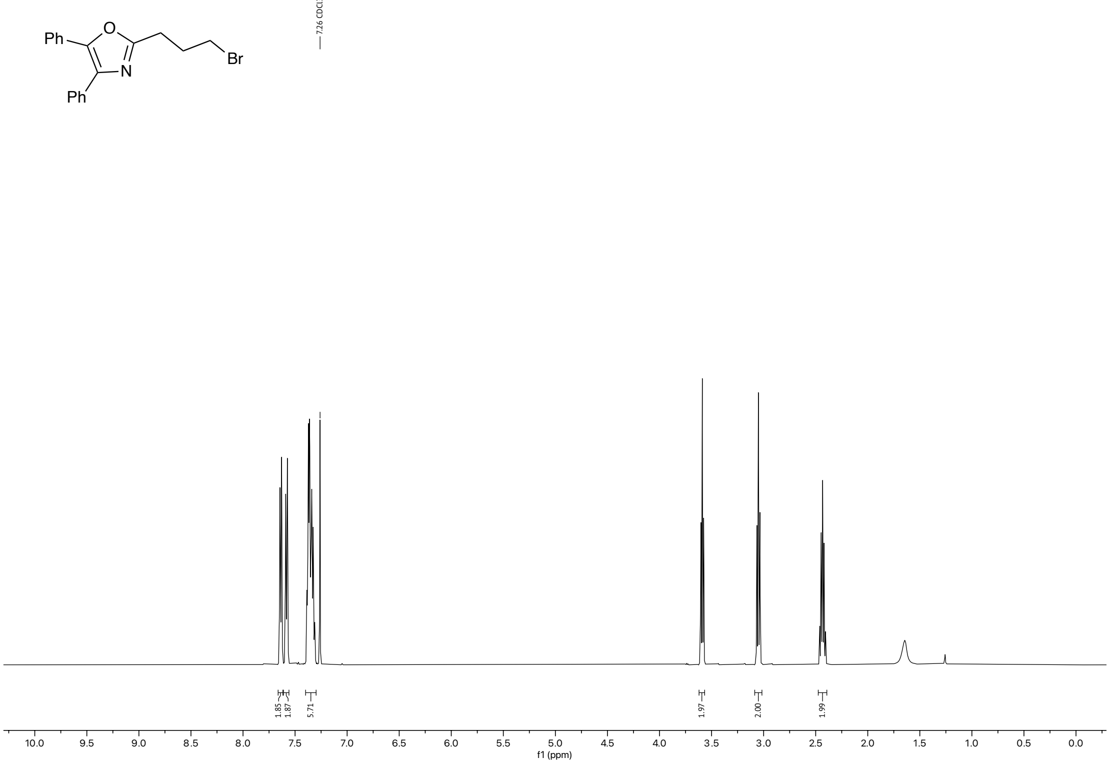

^{13}C NMR of 2-(3-bromopropyl)-4,5-diphenyloxazole (**S2h**) ($CDCl_3$, 126 MHz)

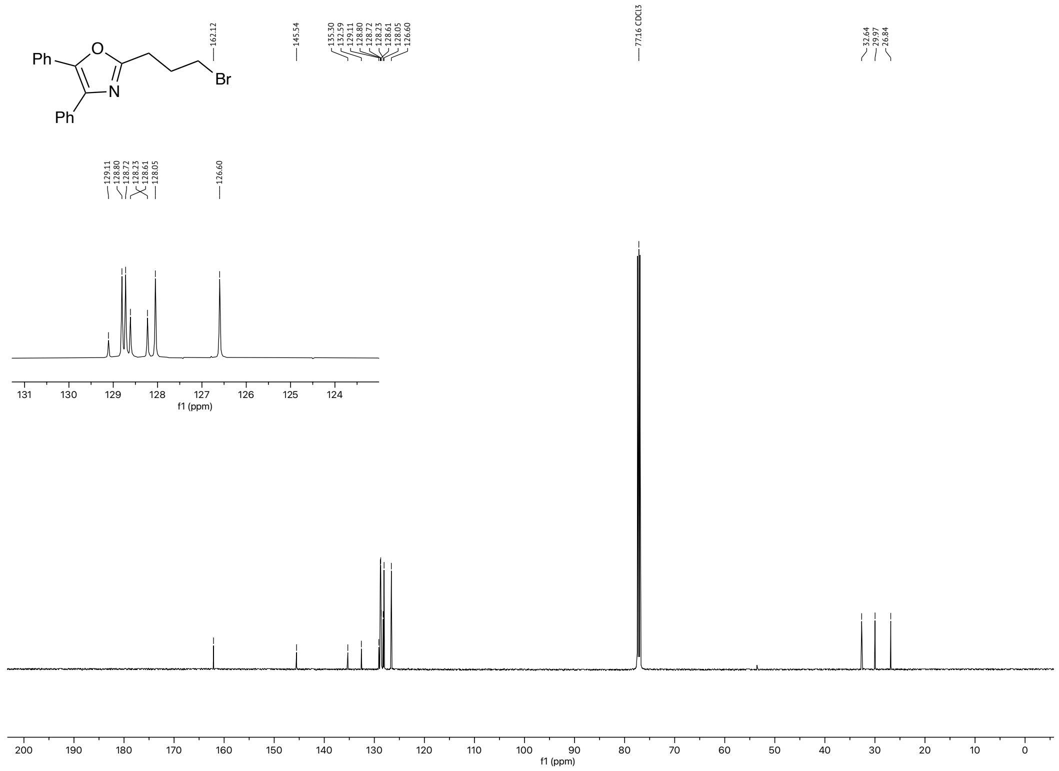

^{1}H NMR of *tert*-butyl 4-bromoazepane-1-carboxylate **(S2o)** ($CDCl_3$, 500 MHz)

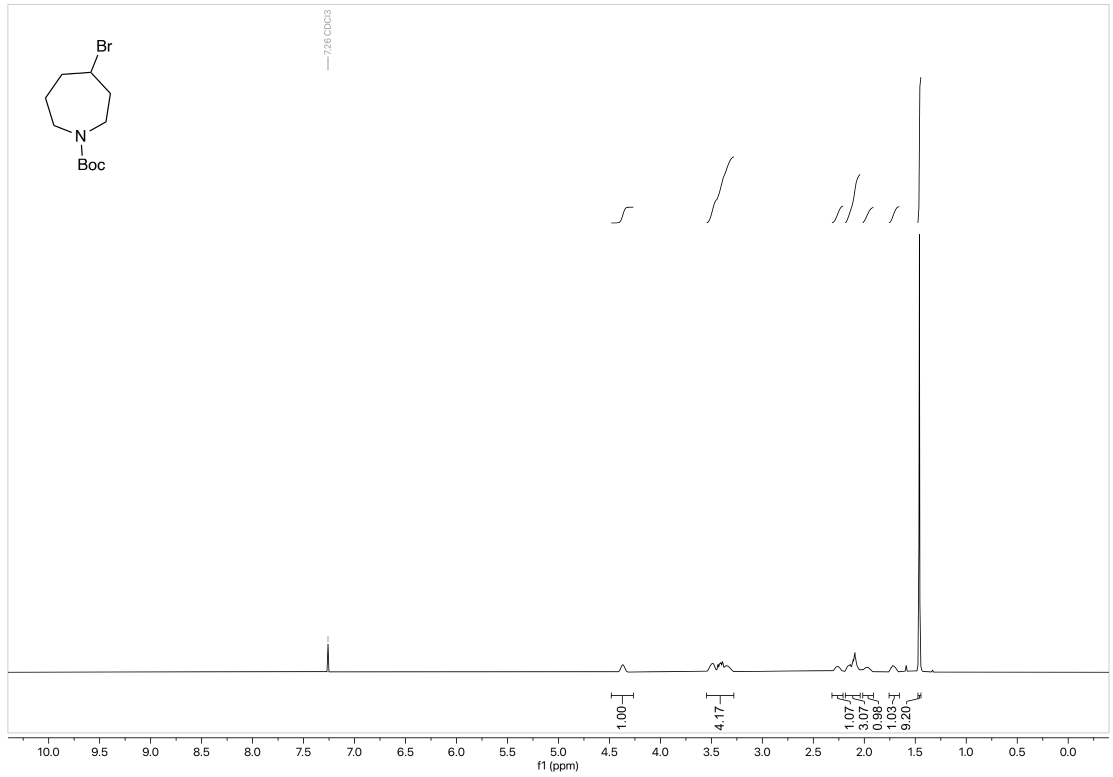

^{13}C NMR of *tert*-butyl 4-bromoazepane-1-carboxylate **(S2o)** ($CDCl_3$, 126 MHz)

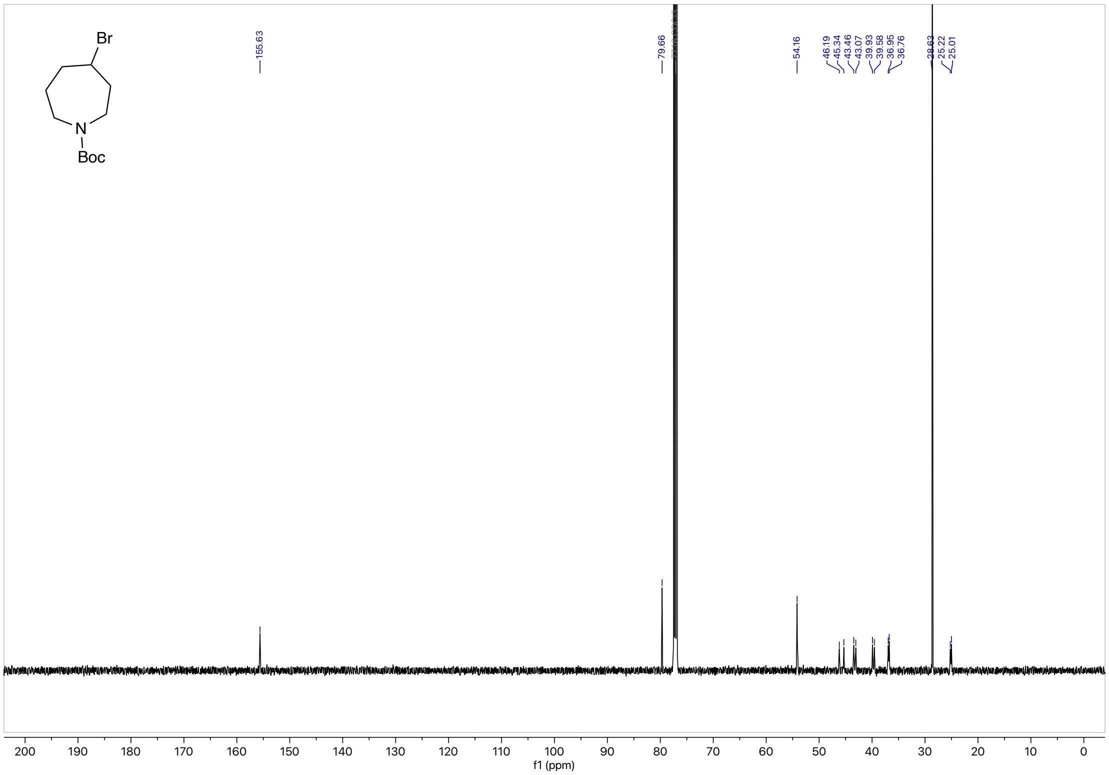

^{1}H NMR of **2-(4-bromopiperidin-1-yl)pyrimidine (S2t)** ($CDCl_3$, 500 MHz)

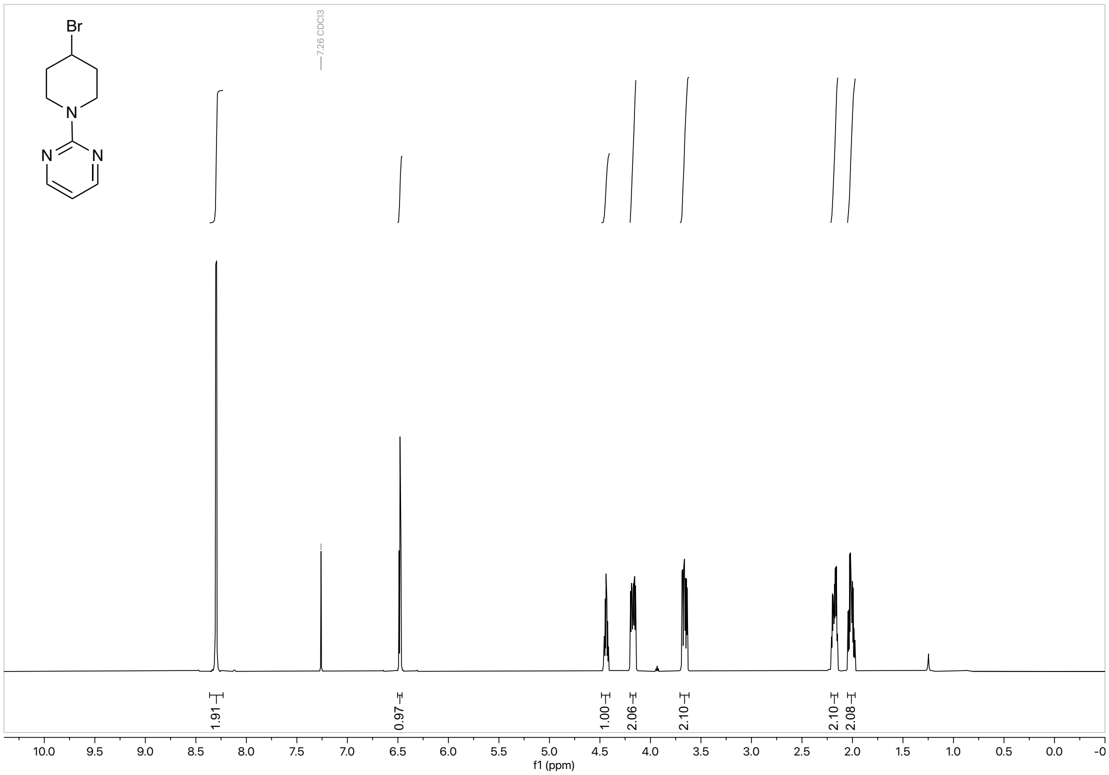

^{13}C NMR of (**S2t**) ($CDCl_3$, 126 MHz)

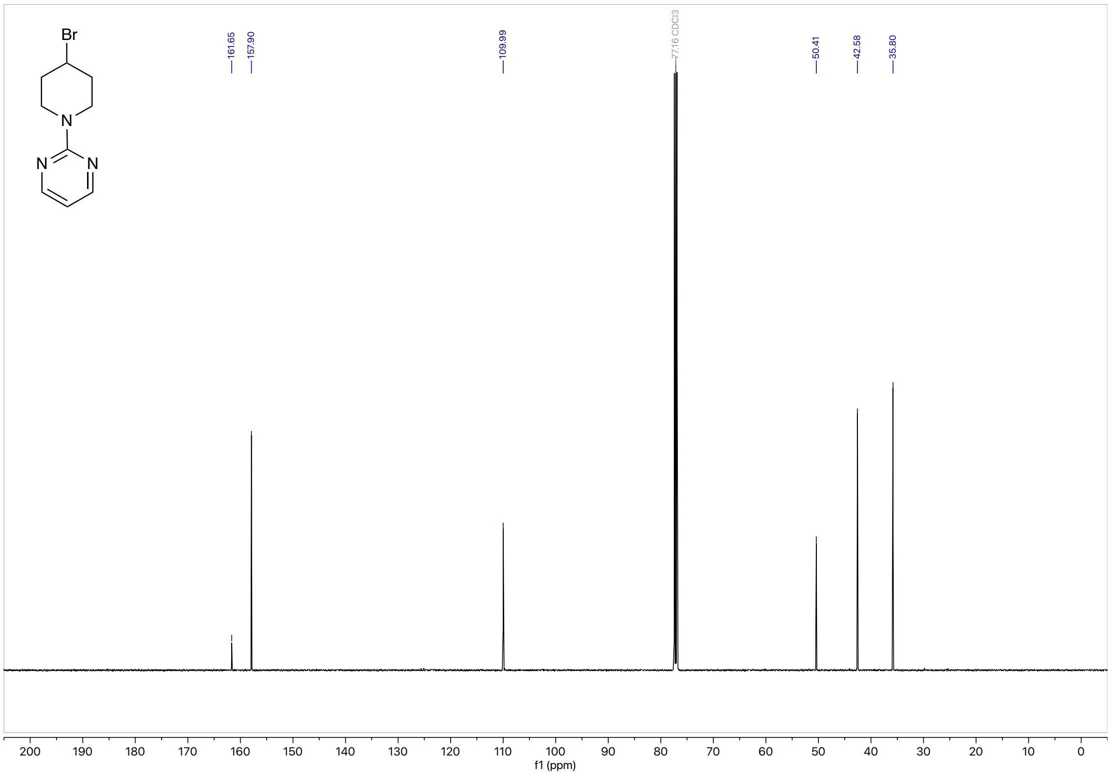

^{1}H NMR of (4-bromopiperidin-1-yl)(2-(trifluoromethyl)pyridin-3-yl)methanone (**S2u**) ($CDCl_3$, 500 MHz)

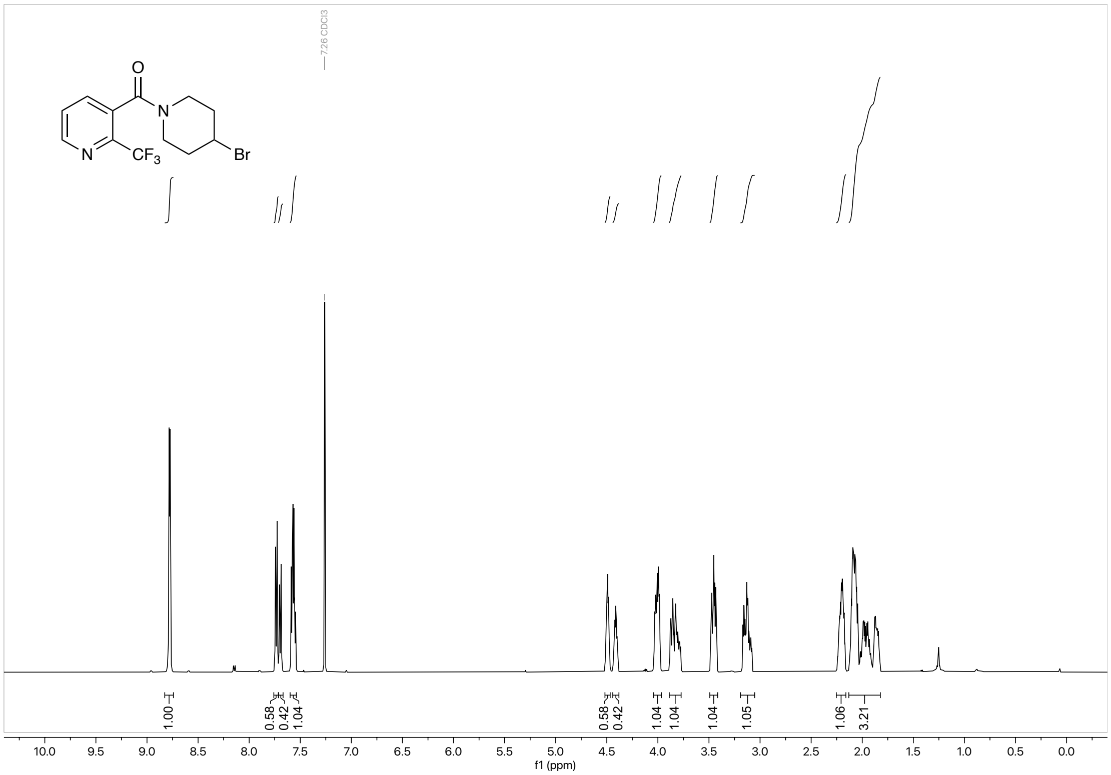

^{13}C NMR of (4-bromopiperidin-1-yl)(2-(trifluoromethyl)pyridin-3-yl)methanone (**S2u**) ($CDCl_3$, 126 MHz)

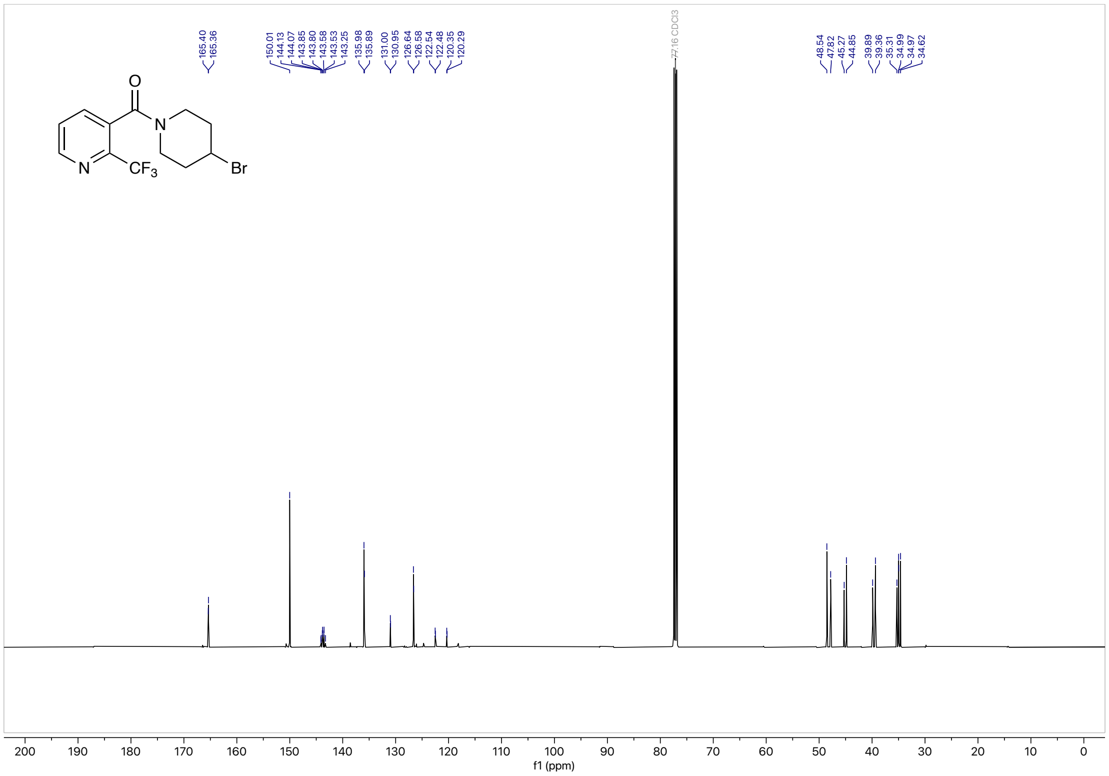

^{19}F NMR of (**S2u**) ($CDCl_3$, 470 MHz)

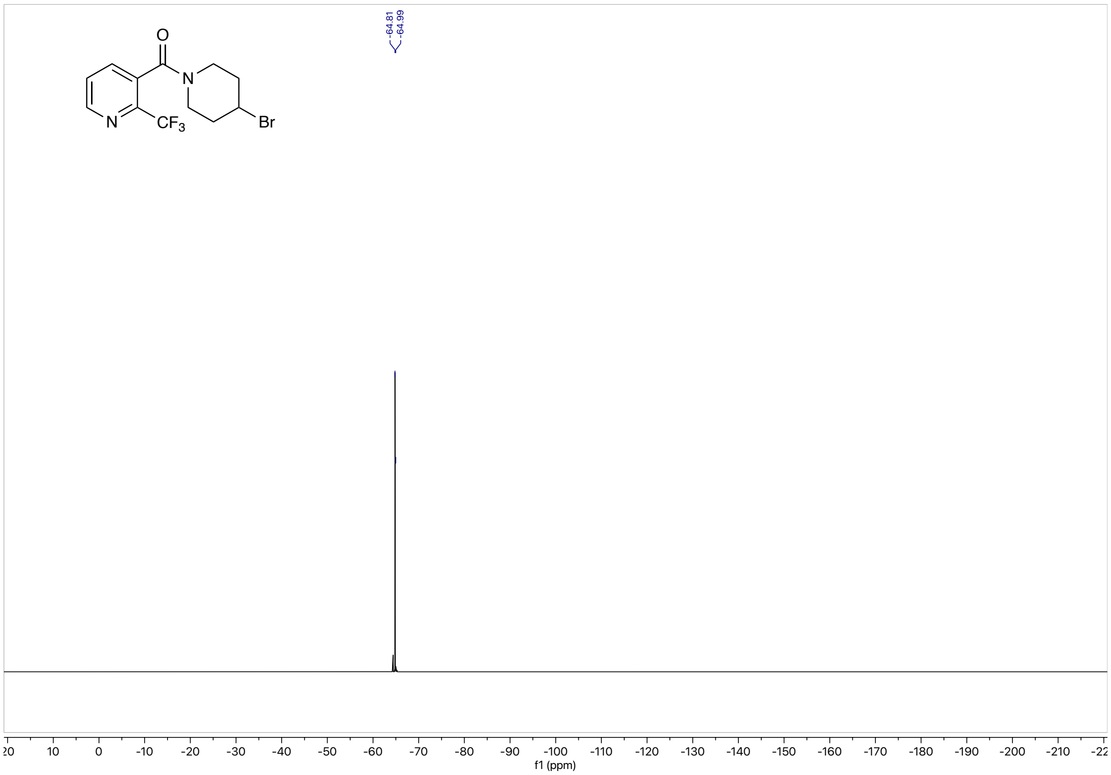

^1H NMR of 4-bromo-4-methylpentyl 4-(trifluoromethyl)benzoate **(S2z)** (500 MHz, $CDCl_3$)

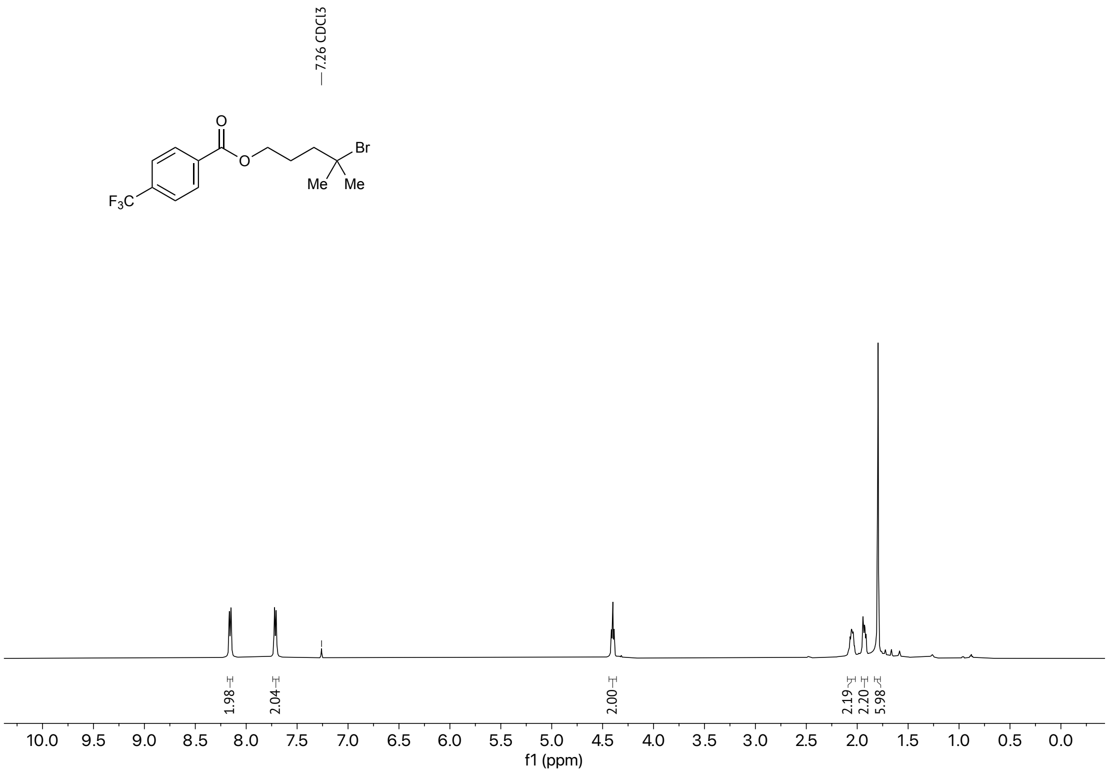

^{13}C NMR of 4-bromo-4-methylpentyl 4-(trifluoromethyl)benzoate **(S2z)** (126 MHz, $CDCl_3$)

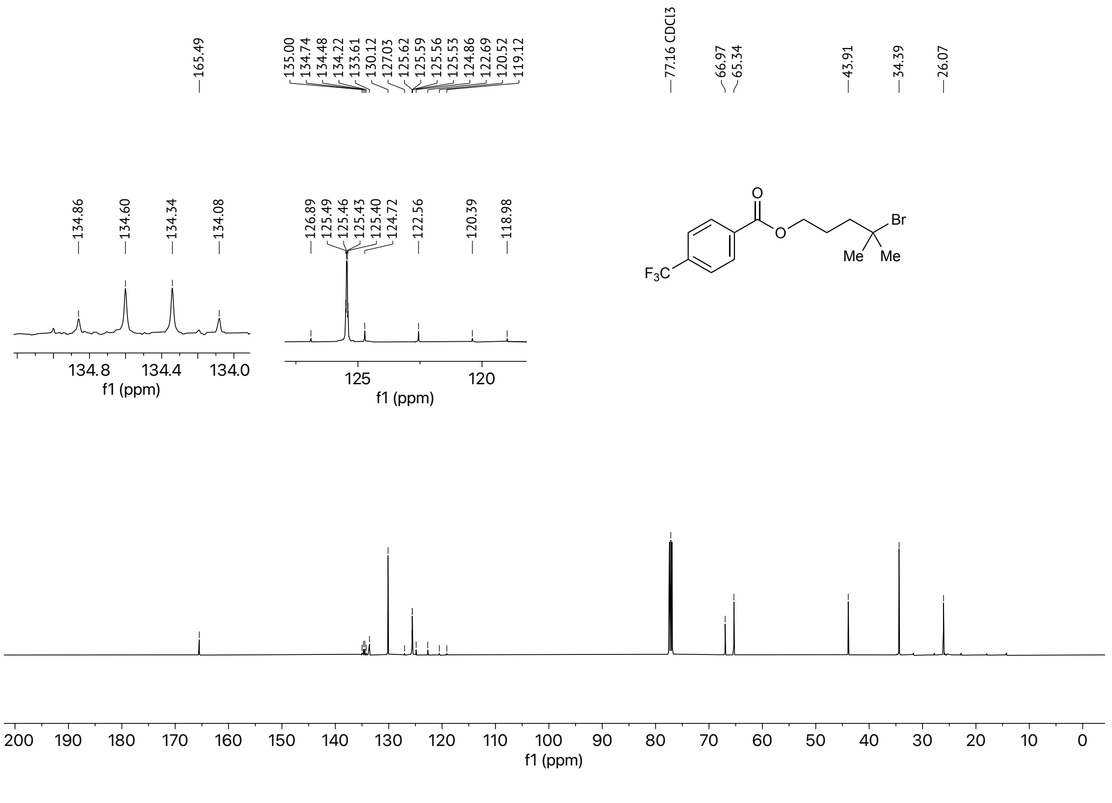

^{19}F NMR of 4-bromo-4-methylpentyl 4-(trifluoromethyl)benzoate **(S2z)** (470 MHz, $CDCl_3$)

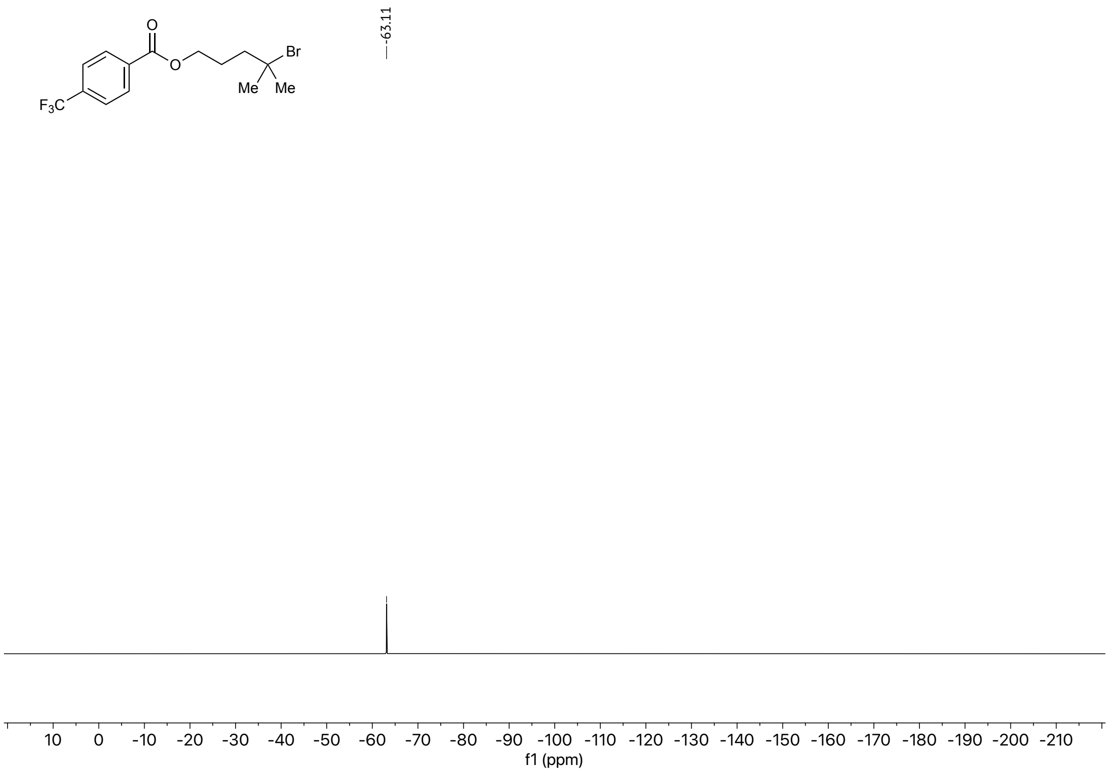

^{1}H NMR of 4-(3-bromo-3-methylbutyl)-2-methoxyphenol (**S2aa**) ($CDCl_3$, 500 MHz)

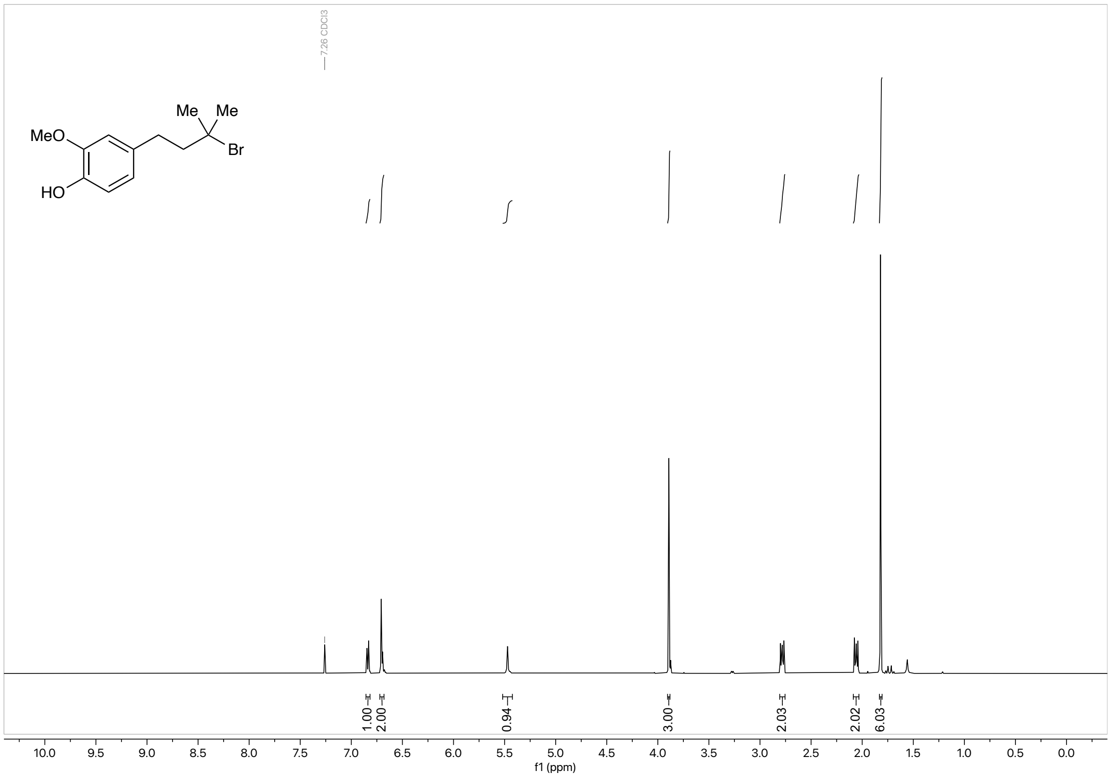

^{13}C NMR of 4-(3-bromo-3-methylbutyl)-2-methoxyphenol (**S2aa**) ($CDCl_3$, 126 MHz)

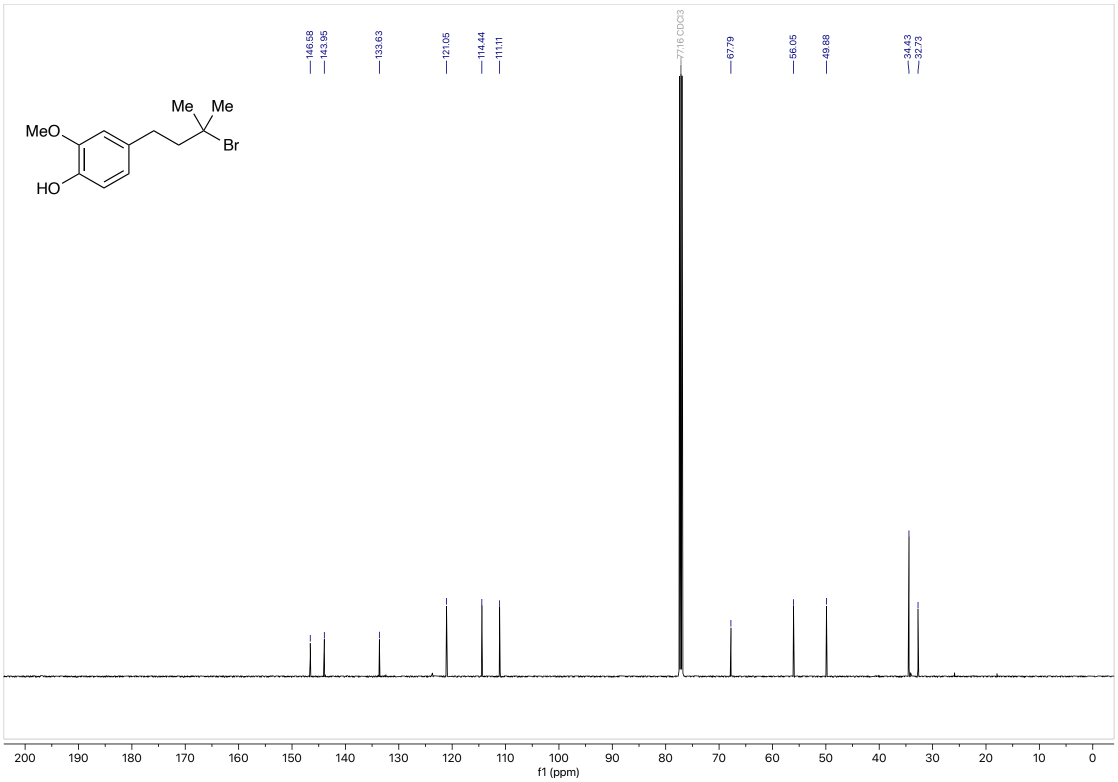

^1H NMR of 4-bromo-4-methylpentyl (*S*)-2-(6-methoxynaphthalen-2-yl)propanoate (**S2ab**) ($CDCl_3$, 500 MHz)

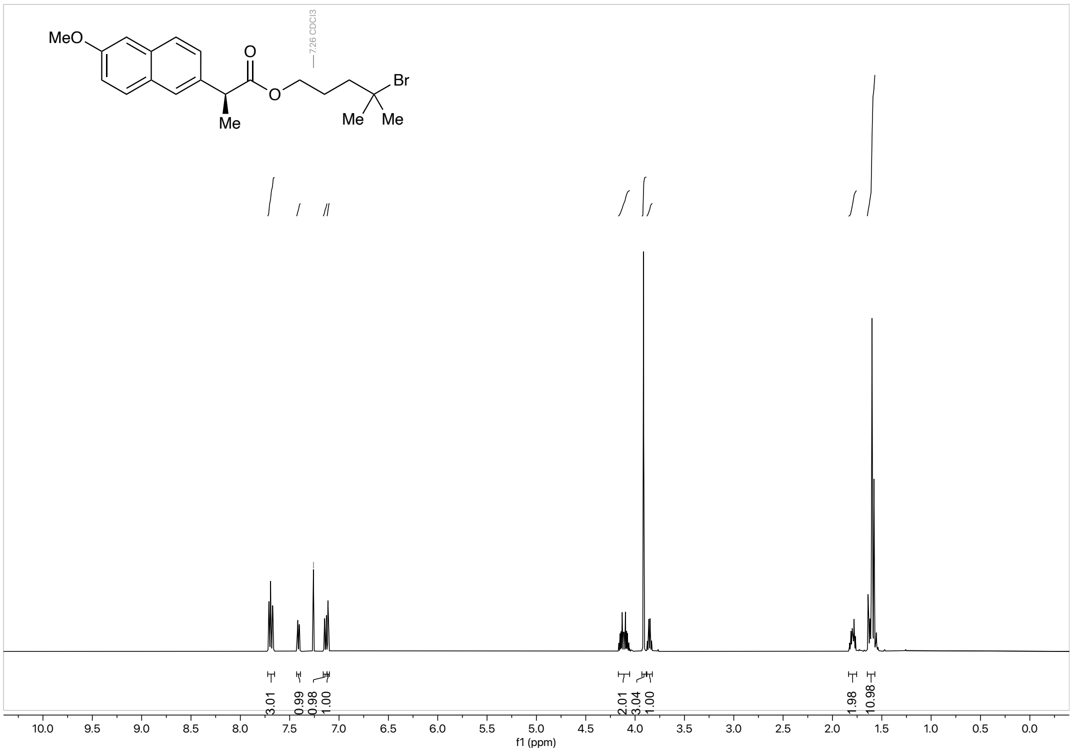

^{13}C NMR **S2ab** ($CDCl_3$, 126 MHz)

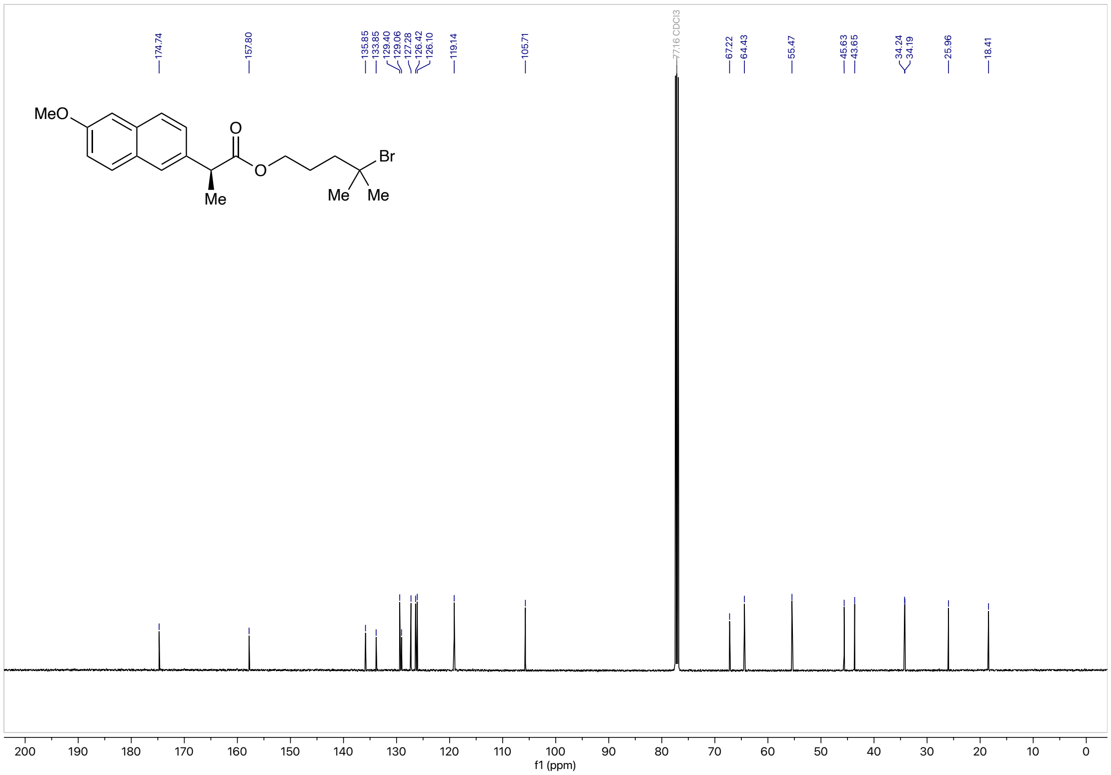

^{1}H NMR of 4,4,5,5-tetraethyl-2-(3-phenylpropyl)-1,3,2-dioxaborolane (**3a**) (500 MHz, $CDCl_3$)

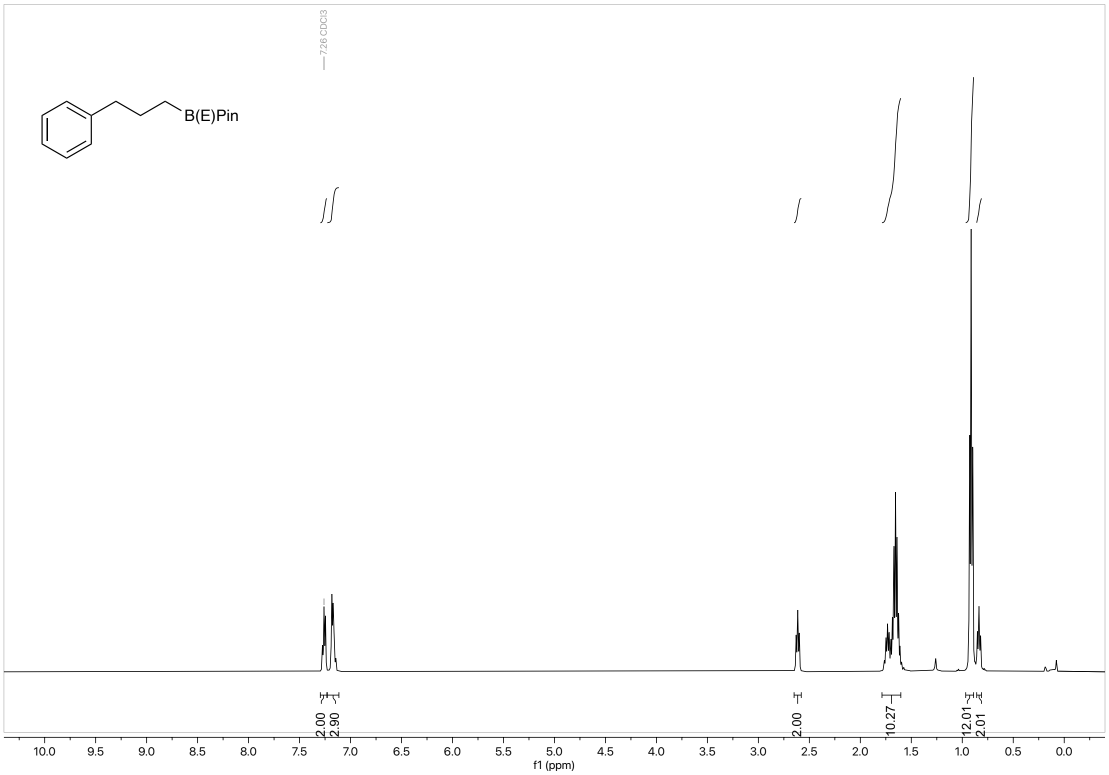

^{13}C NMR of (**3a**) (126 MHz, $CDCl_3$)

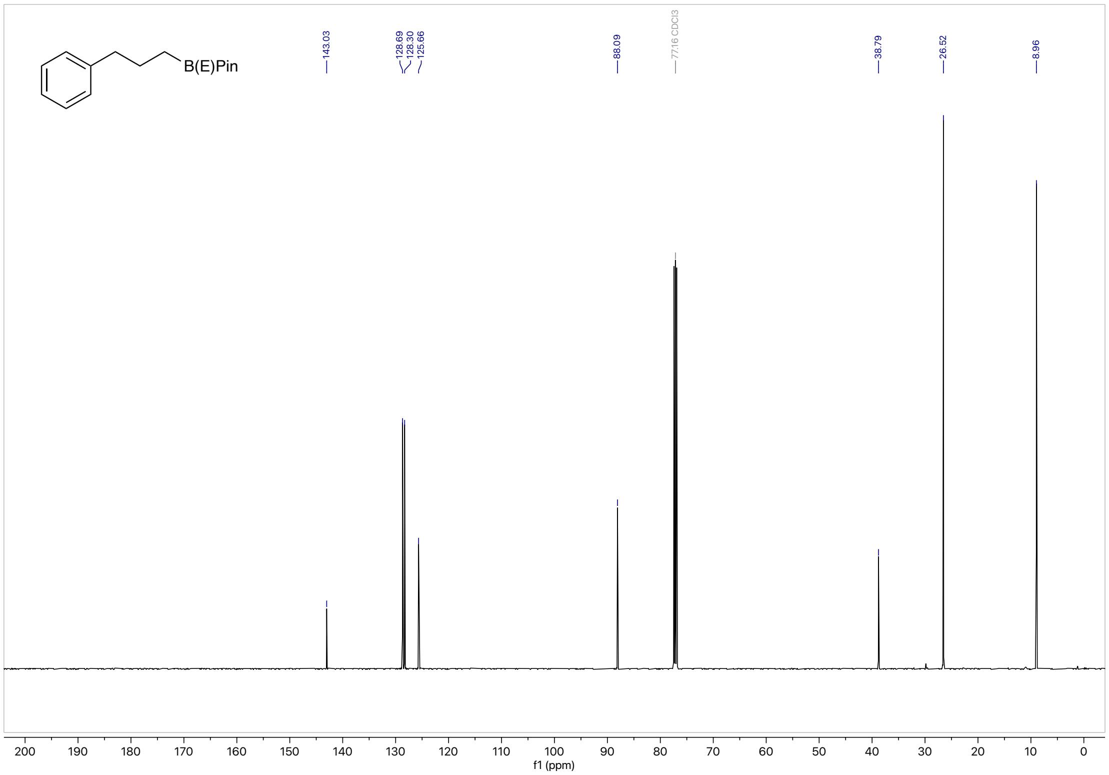

^{11}B NMR of (**3a**) (160 MHz, $CDCl_3$)

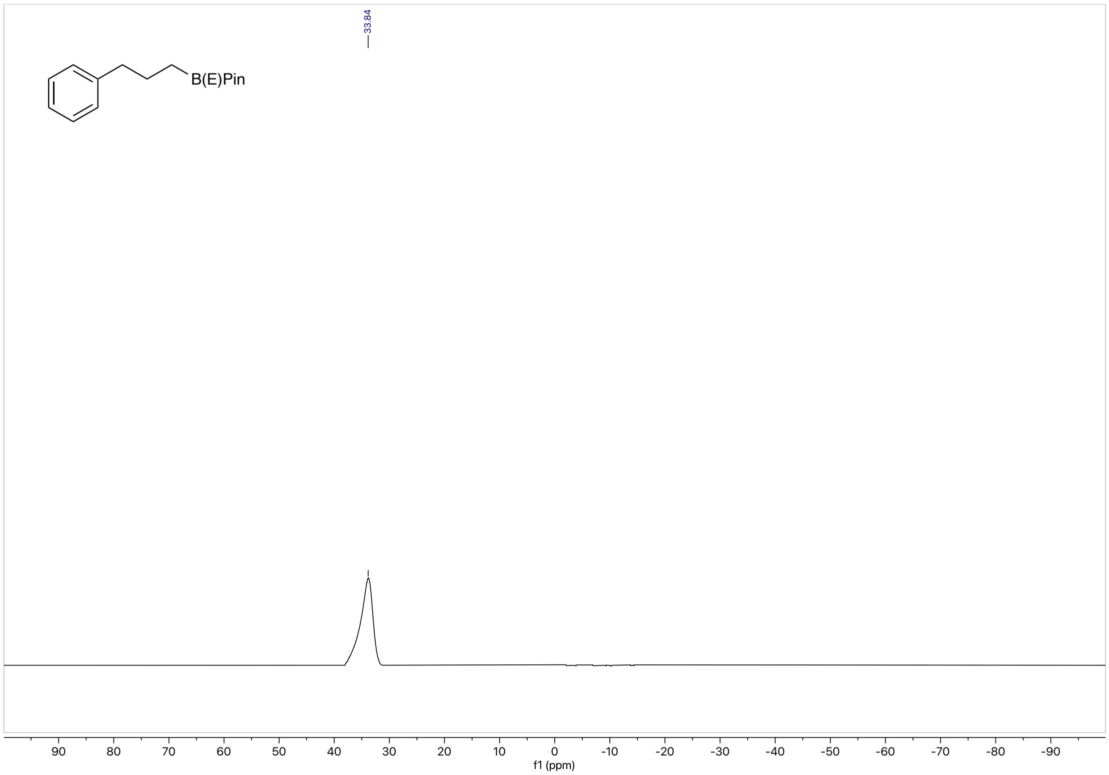

^{1}H NMR of ethyl 7-(4,4,5,5-tetraethyl-1,3,2-dioxaborolan-2-yl)heptanoate (**3b**) (500 MHz, $CDCl_3$)

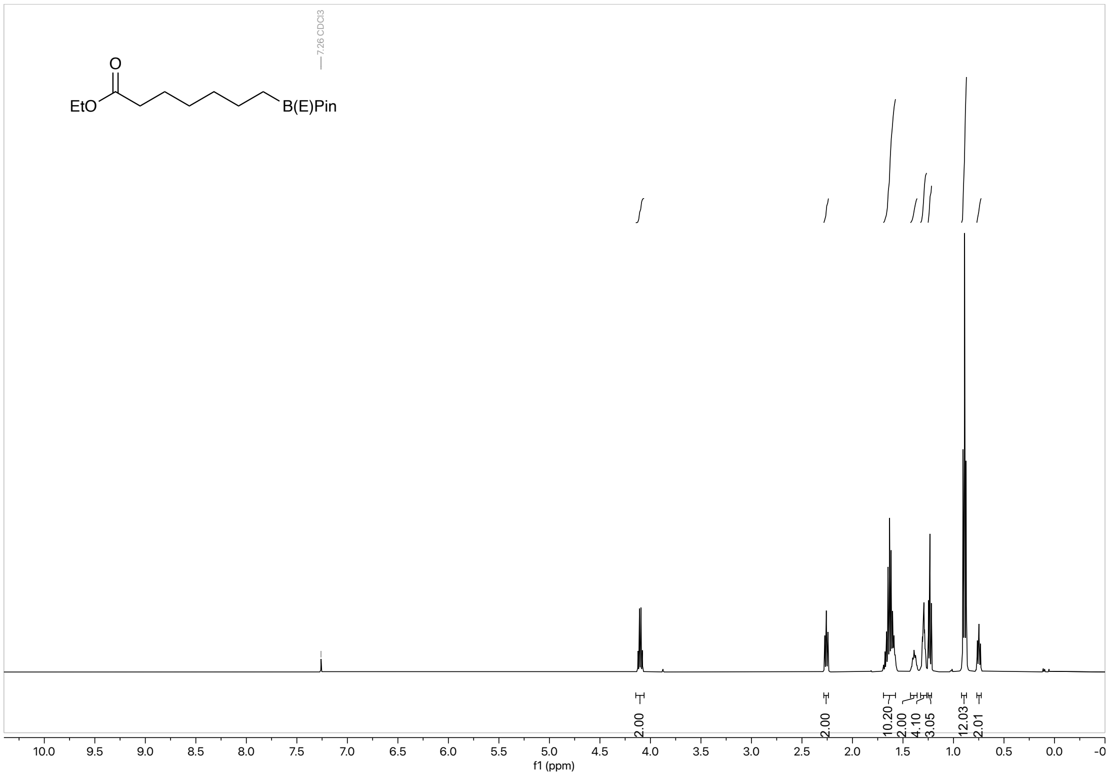

^{13}C NMR of (**3b**) (126 MHz, $CDCl_3$)

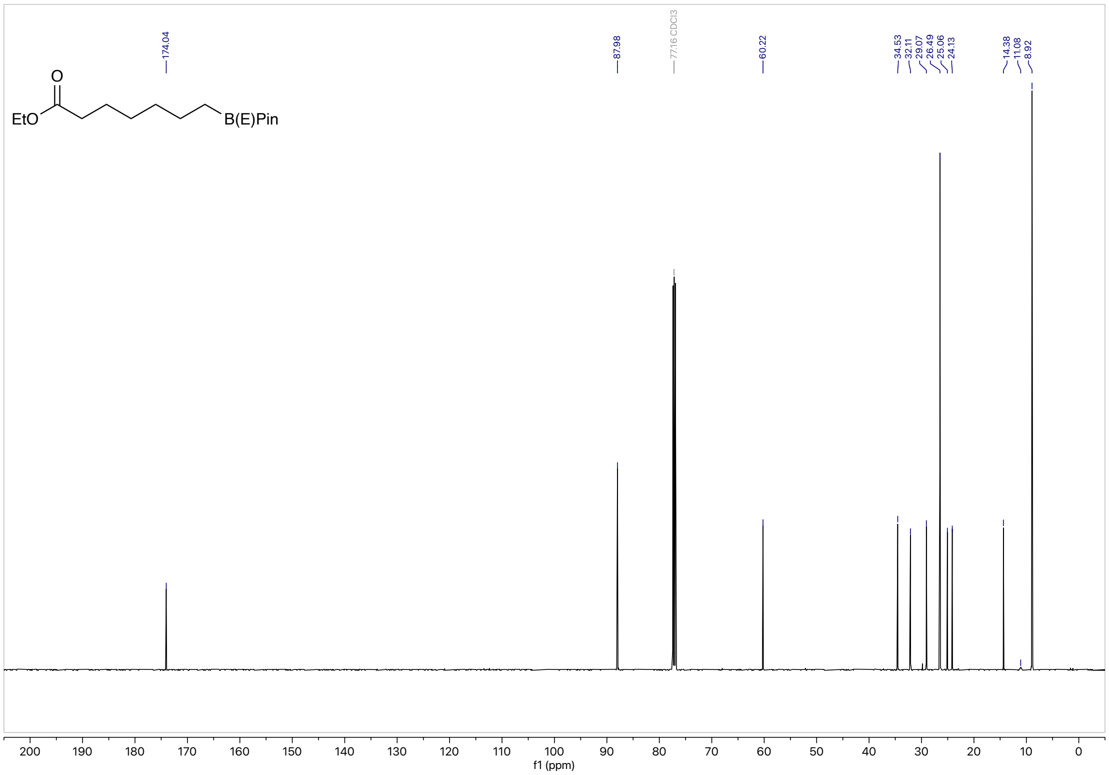

^{11}B NMR of (**3b**) (160 MHz, $CDCl_3$)

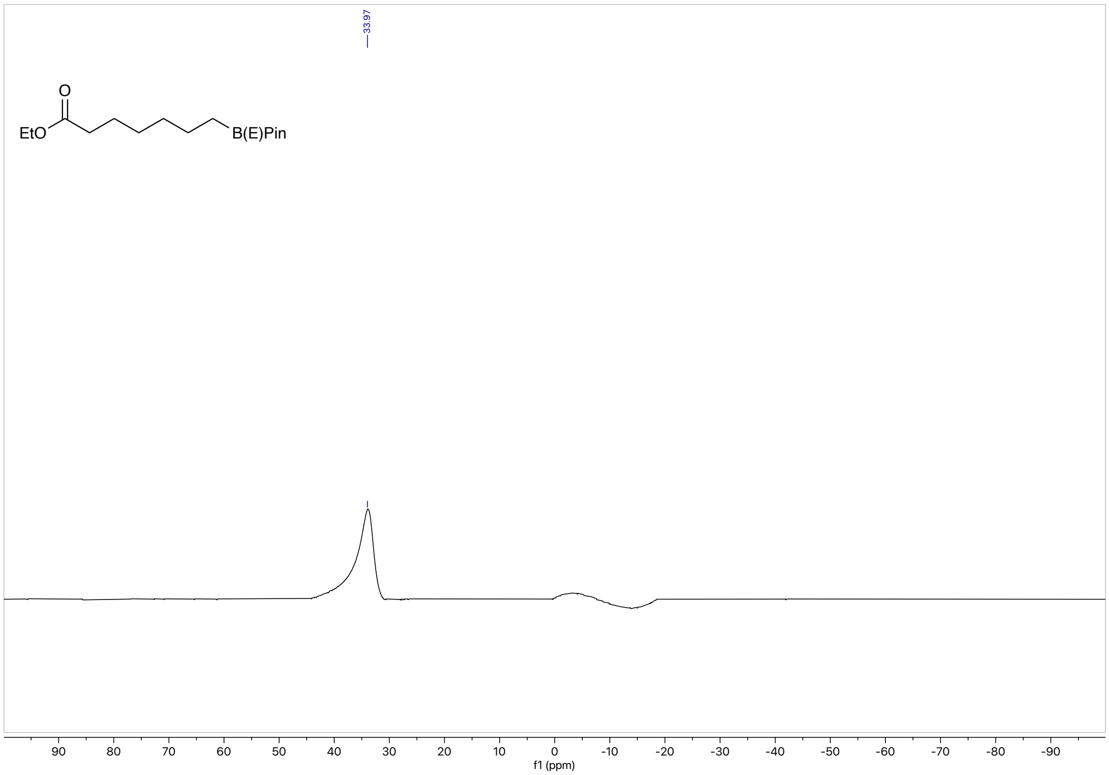

^{1}H NMR of 6-methyl-2-(4,4,4-trifluorobutyl)-1,3,6,2-dioxazaborocane-4,8-dione **(3c)** (500 MHz, CD_3CN)

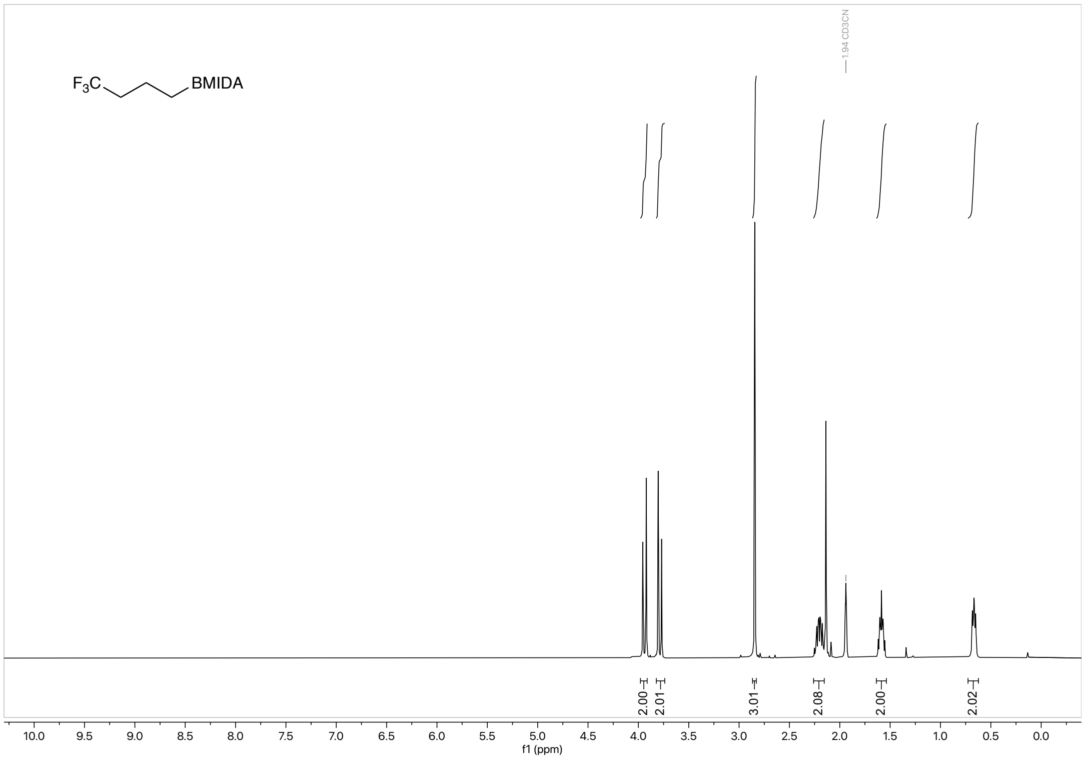

^{13}C NMR of **(3c)** (126 MHz, CD_3CN)

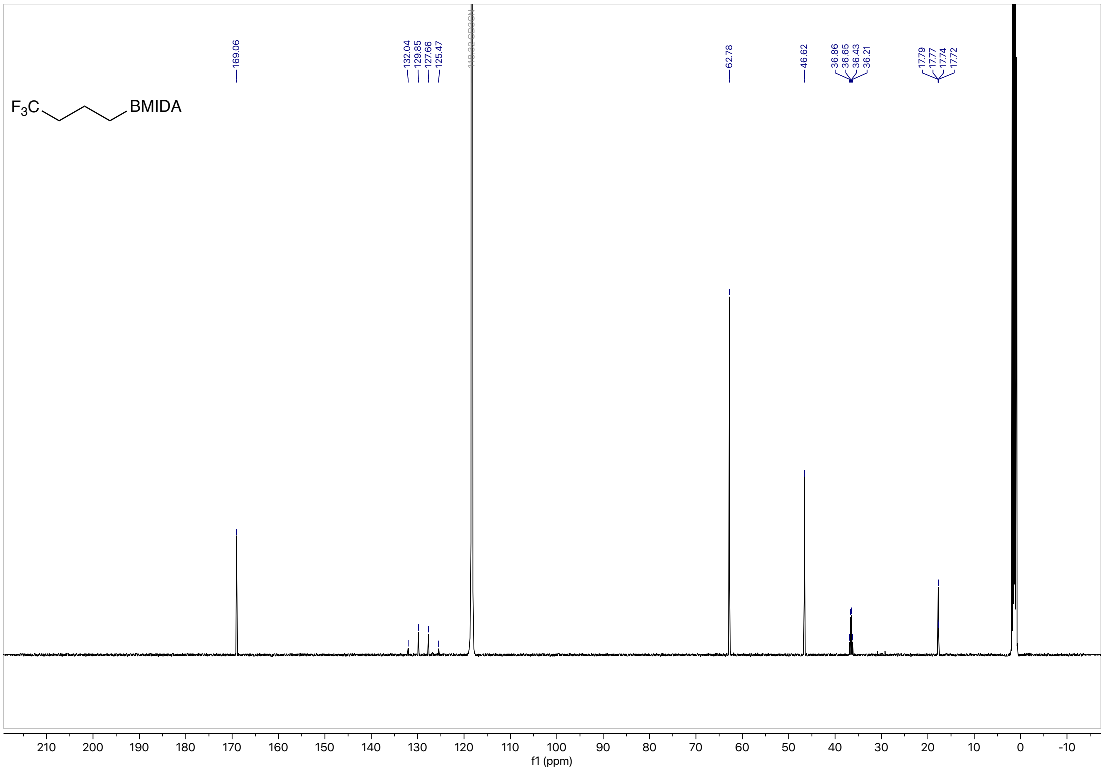

^{19}F NMR of **(3c)** (470 MHz, CD_3CN)

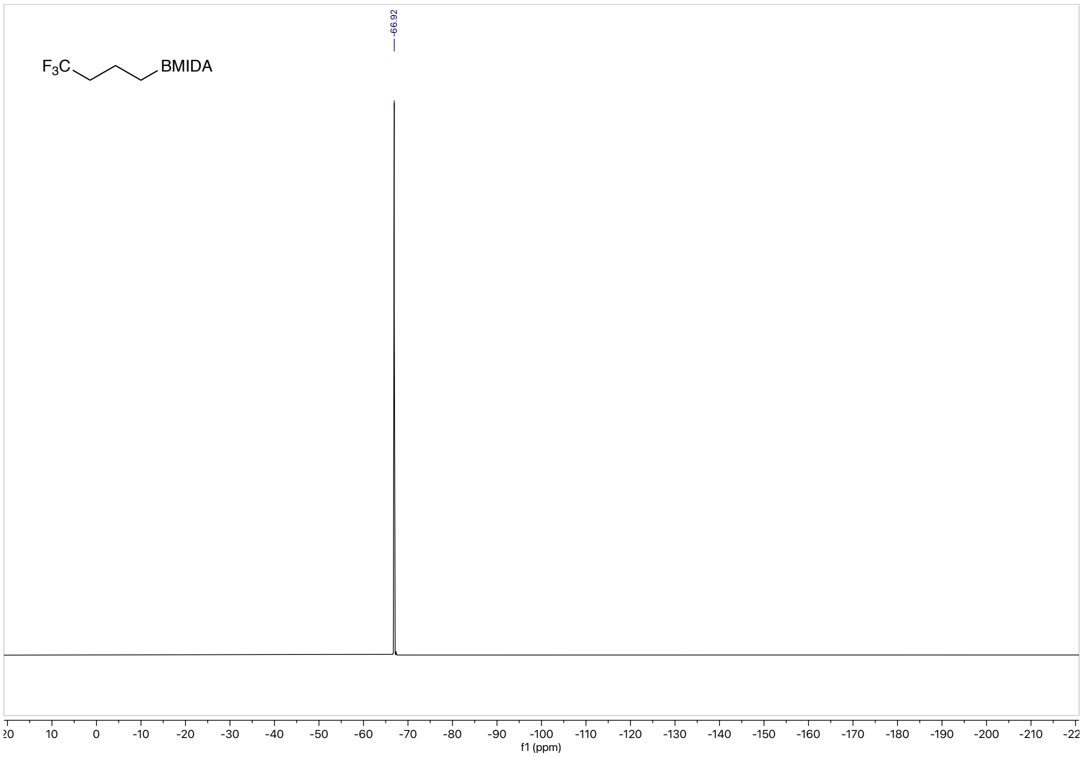

^{11}B NMR of **(3c)** (160 MHz, CD_3CN)

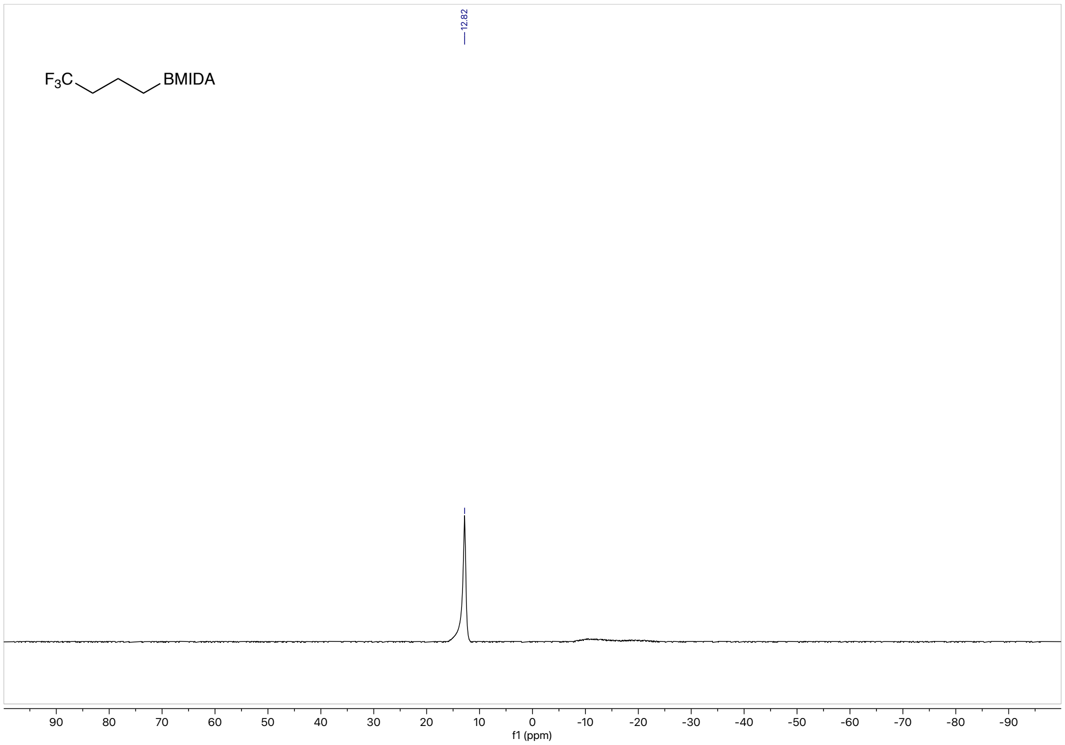

^{1}H NMR of 2-(2-(1,3-dioxan-2-yl)ethyl)-4,4,5,5-tetraethyl-1,3,2-dioxaborolane (**3d**) (500 MHz,$CDCl_3$)

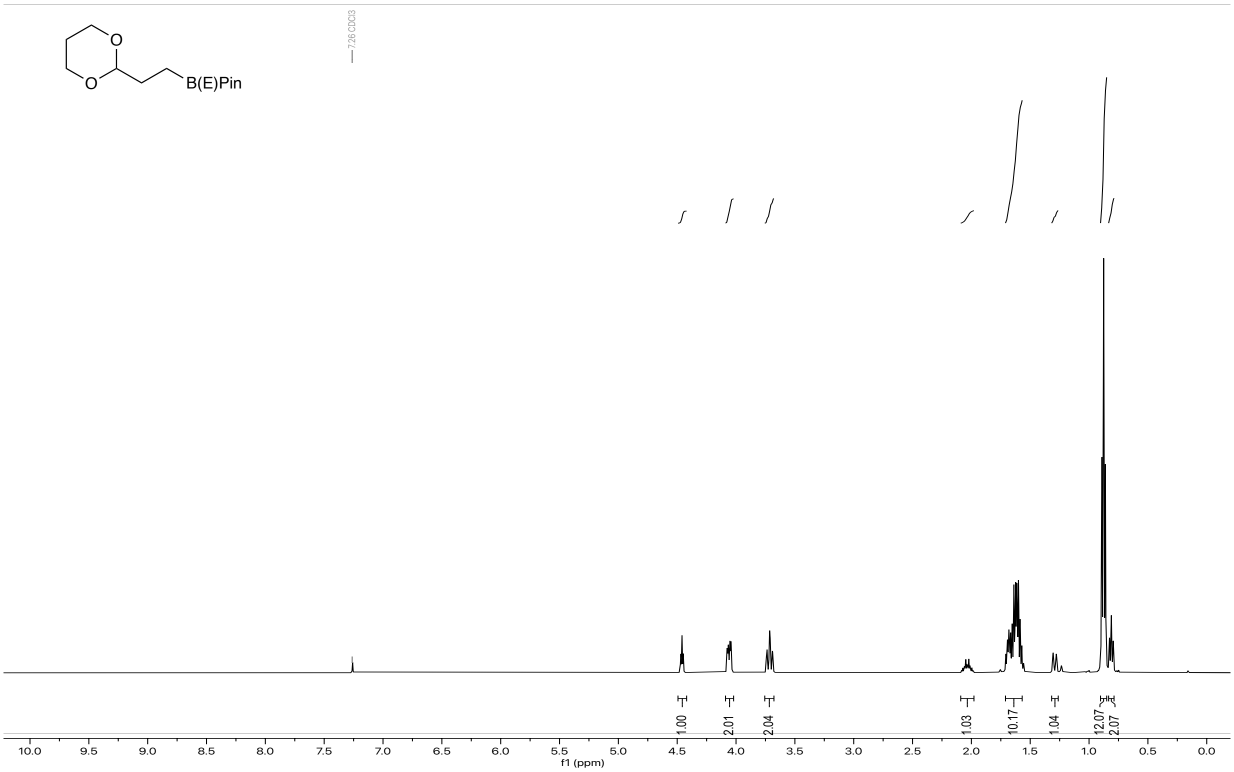

^{13}C NMR of (**3d**) (126 MHz, $CDCl_3$)

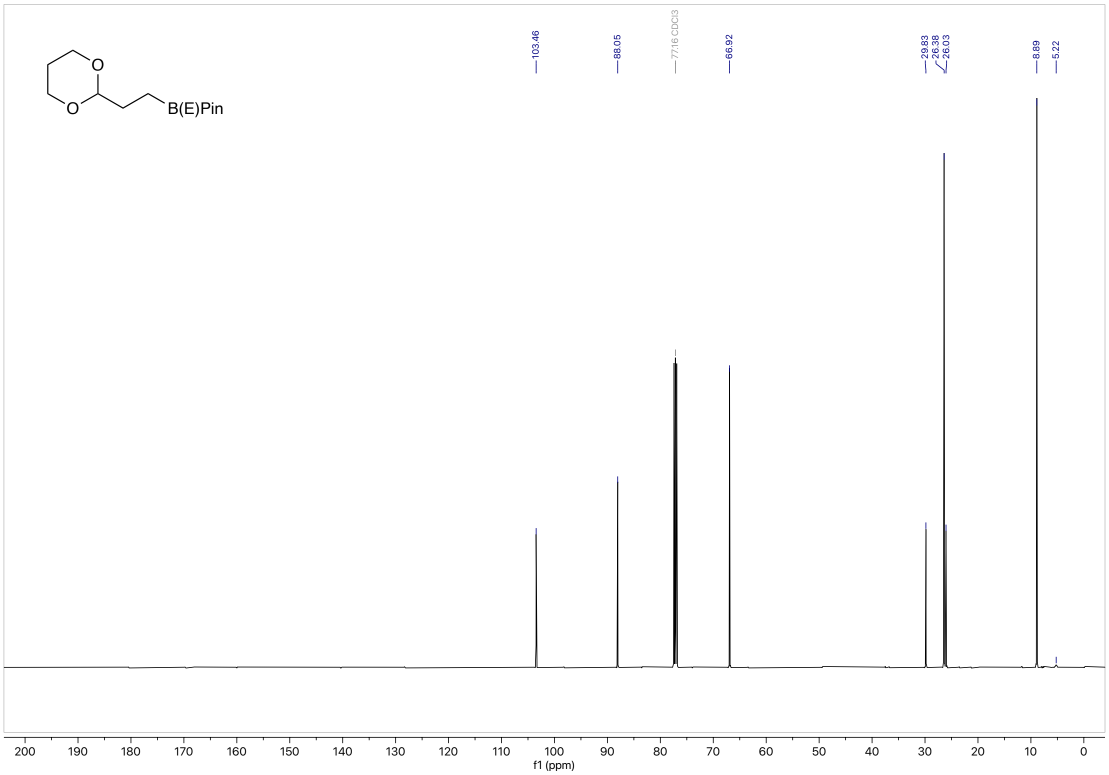

^{11}B NMR of (**3d**) (160 MHz, $CDCl_3$)

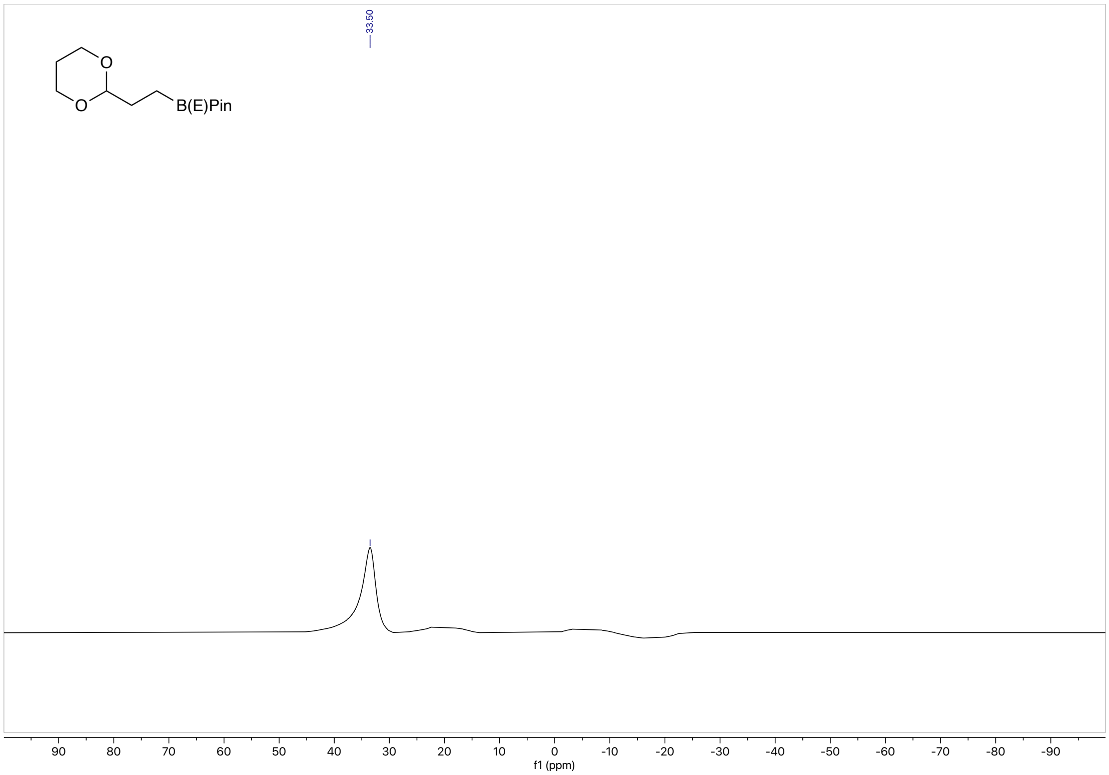

^{1}H NMR of 6-methyl-2-(4-((2-oxo-1,2,3,4-tetrahydroquinolin-7-yl)oxy)butyl)-1,3,6,2-dioxazaborocane-4,8-dione (**3e)** (500 MHz, DMSO)

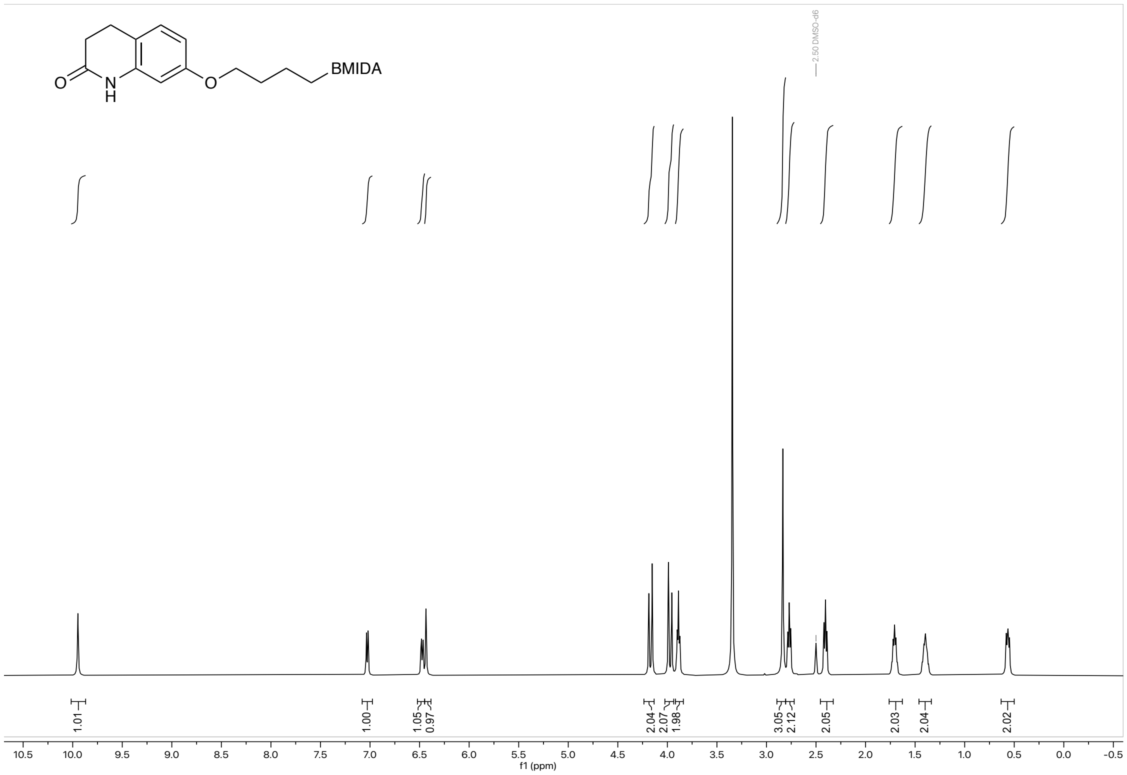

^{13}C NMR of (**3e**) (126 MHz, DMSO)

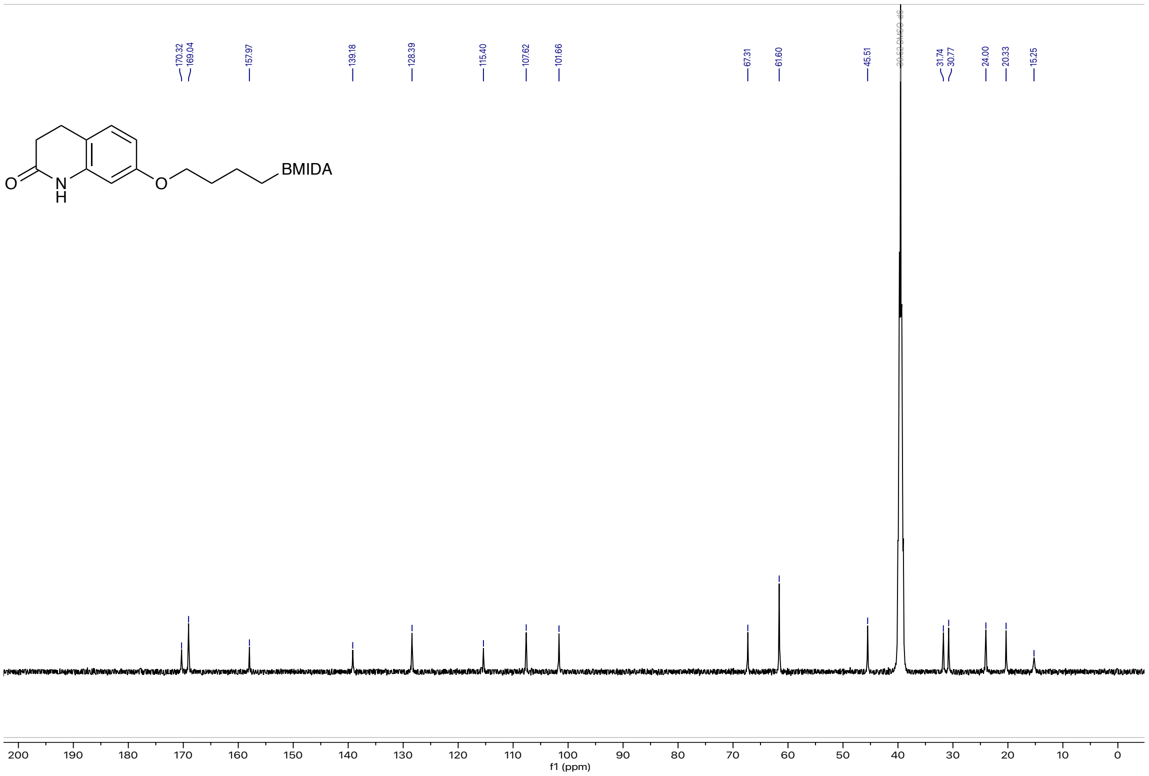

^{11}B NMR of (**3e**) (160 MHz, DMSO)

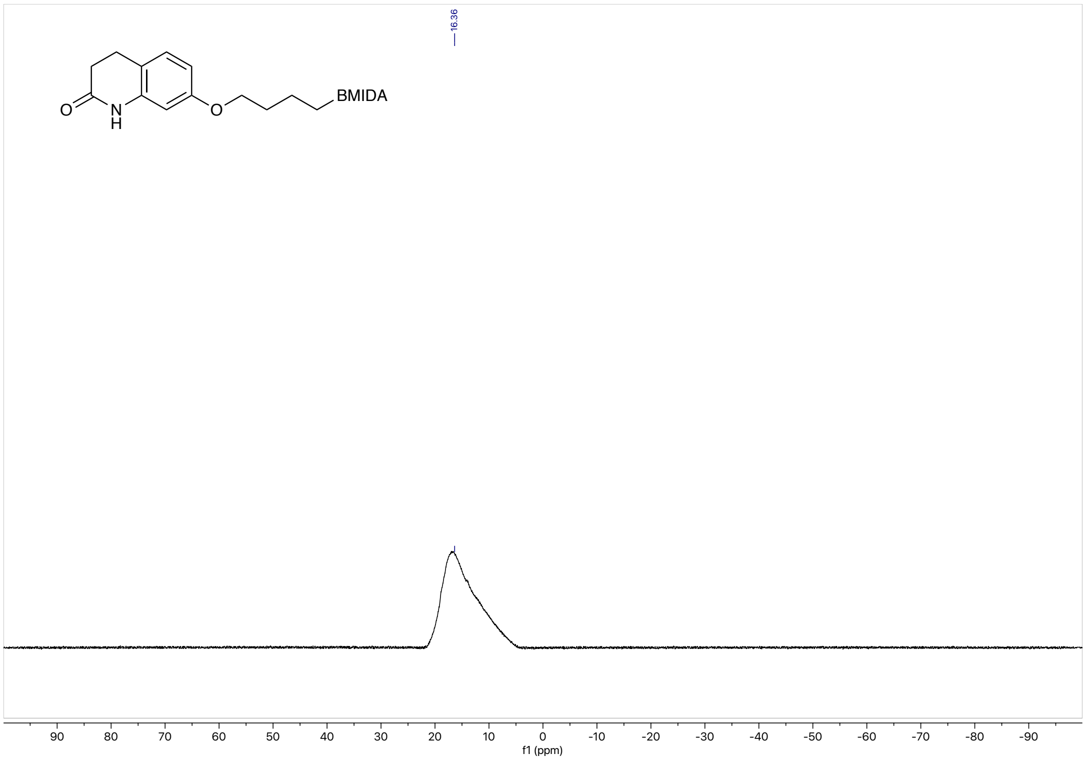

^{1}H NMR of 9-(2-(4,4,5,5-tetraethyl-1,3,2-dioxaborolan-2-yl)ethyl)-9*H*-carbazole (**3f**) (500 MHz, $CDCl_3$)

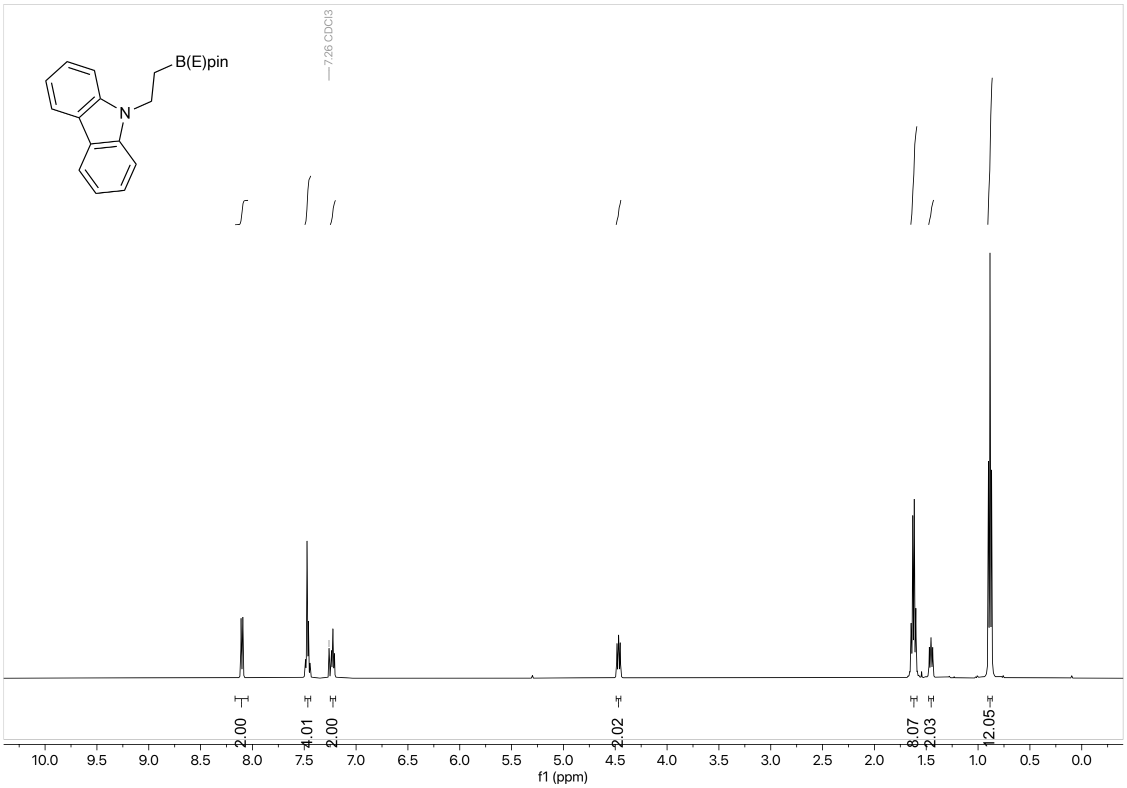

[13] C NMR of (**3f**) (126 MHz, $CDCl_3$)

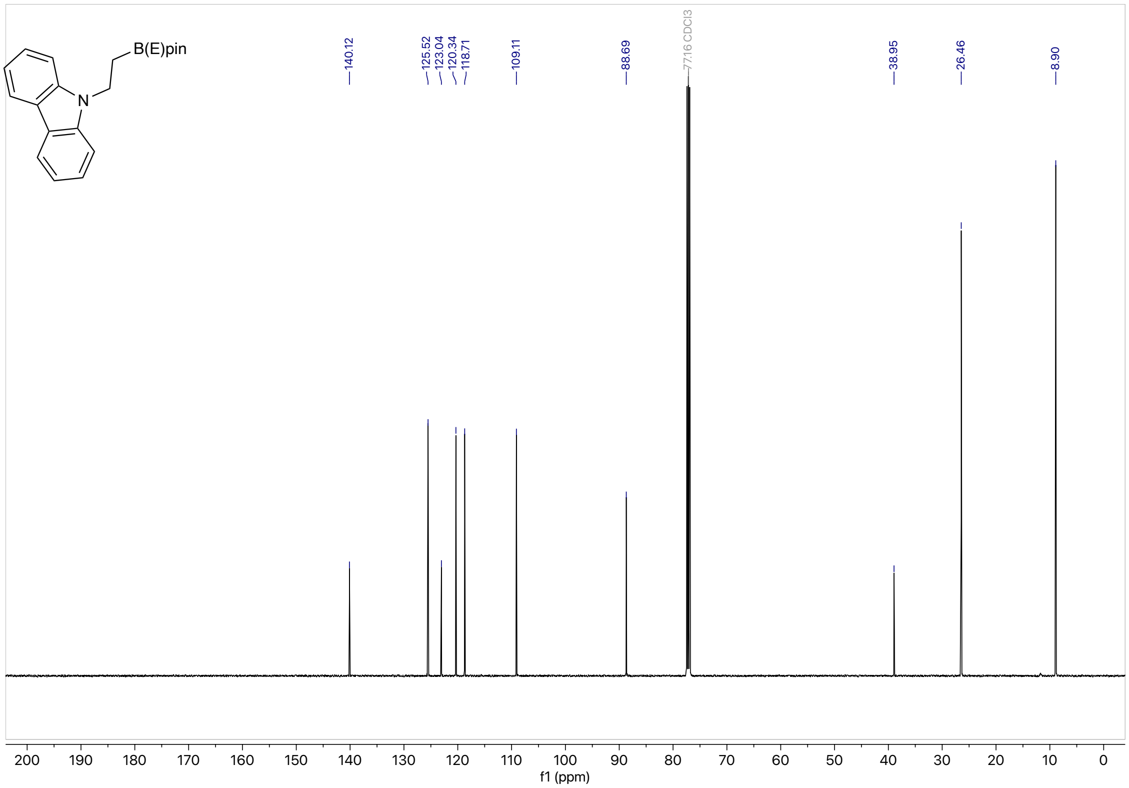

[11] B NMR of (**3f**) (160 MHz, $CDCl_3$)

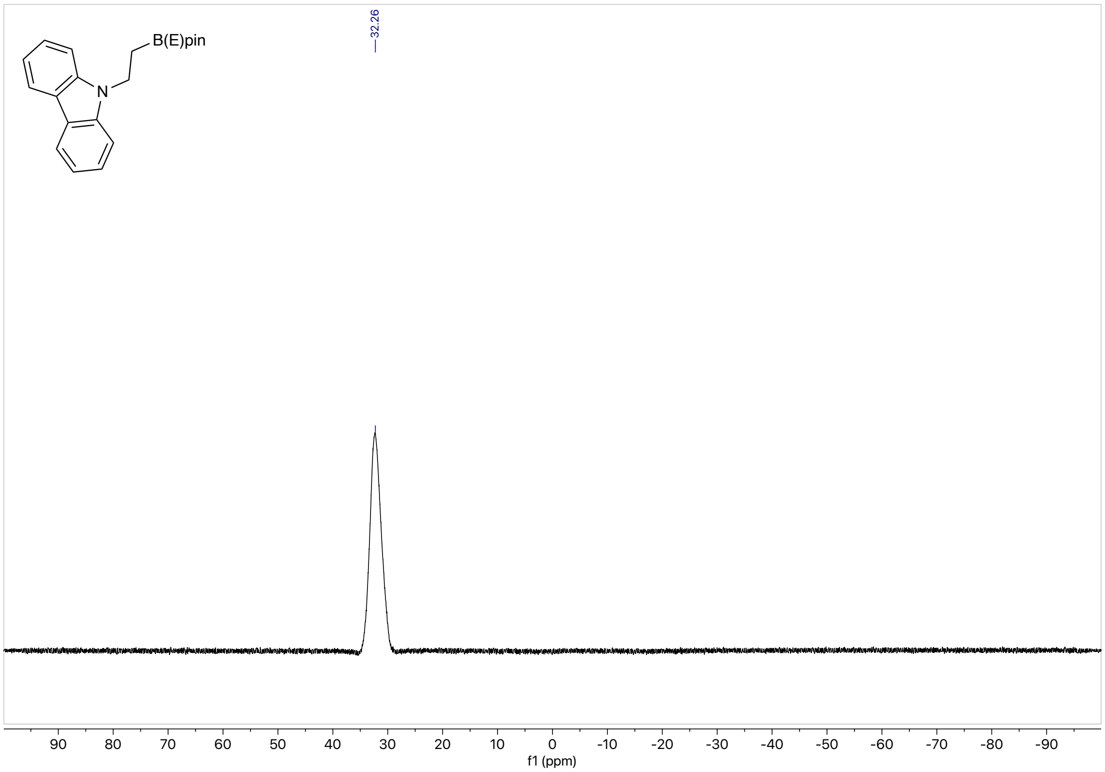

^{1}H NMR of 3-(2-(4,4,5,5-tetramethyl-1,3,2-dioxaborolan-2-yl)ethyl)-1*H*-indole (**3g**) (500 MHz, $CDCl_3$)

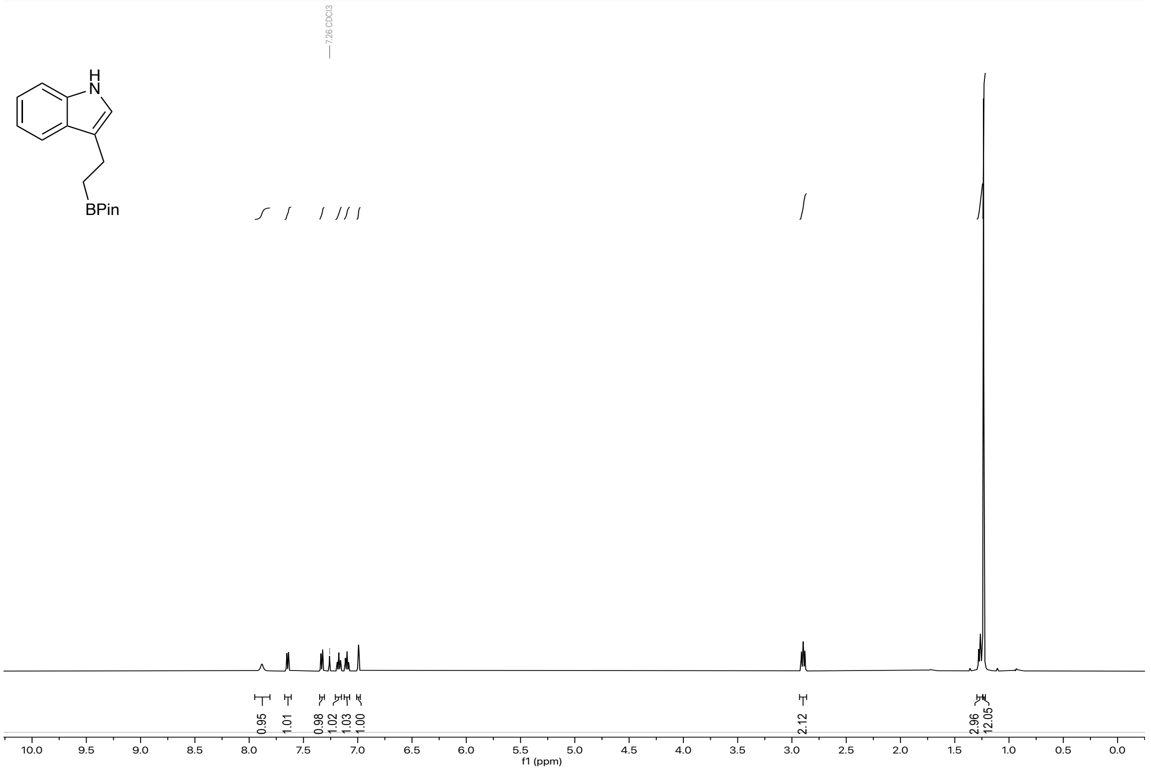

^{13}C NMR of (**3g**) (126 MHz, $CDCl_3$)

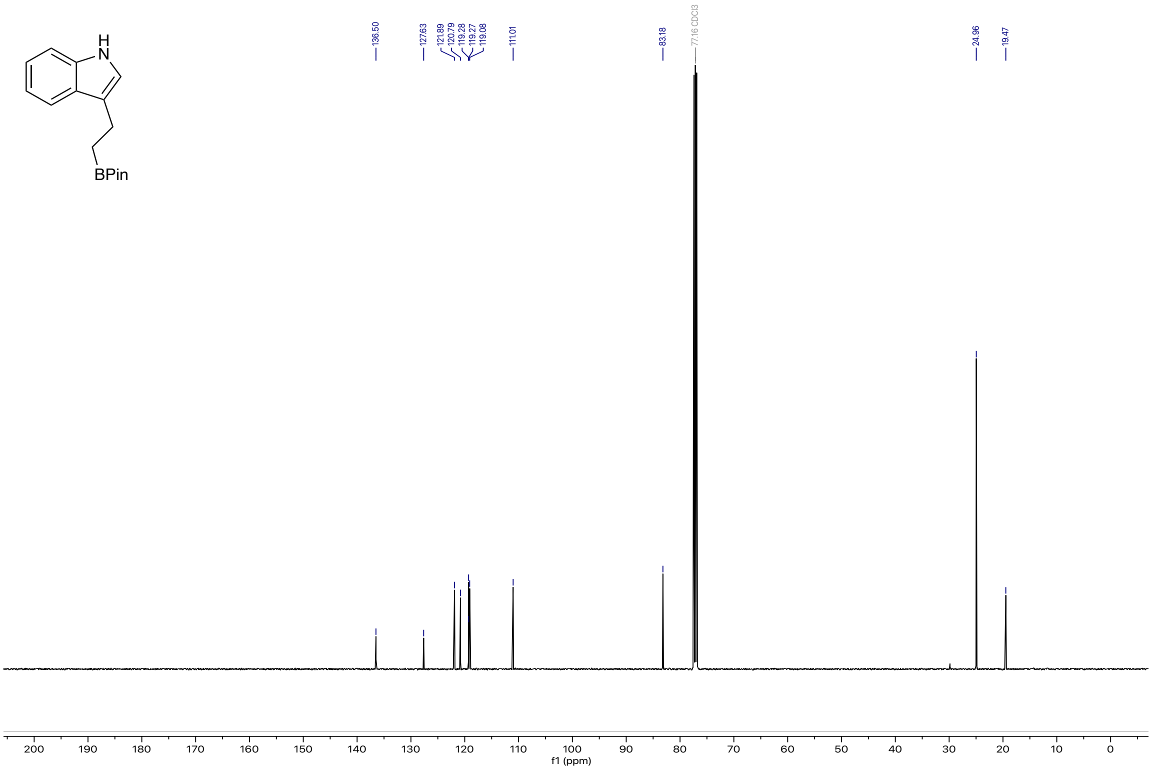

^{1}H NMR of 2-(3-(4,5-diphenyloxazol-2-yl)propyl)-6-methyl-1,3,6,2-dioxazaborocane-4,8-dione (**3h**) (500 MHz, CD_3CN)

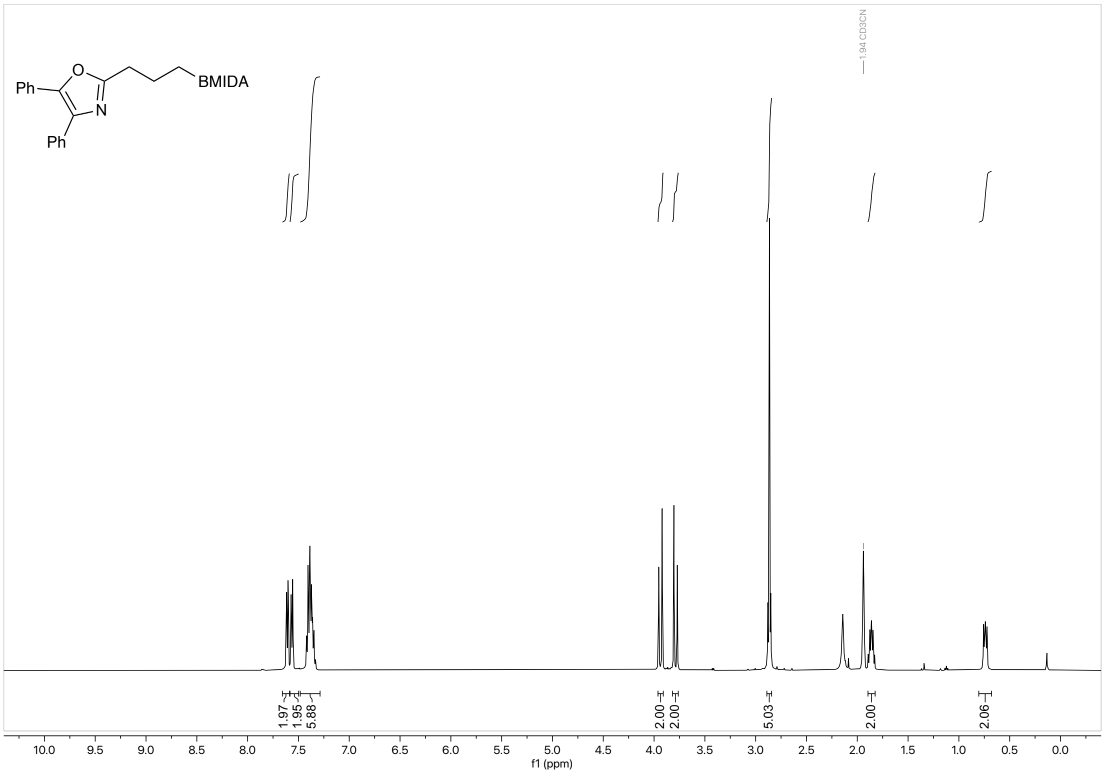

^{13}C NMR of (**3h**) (126MHz, CD_3CN)

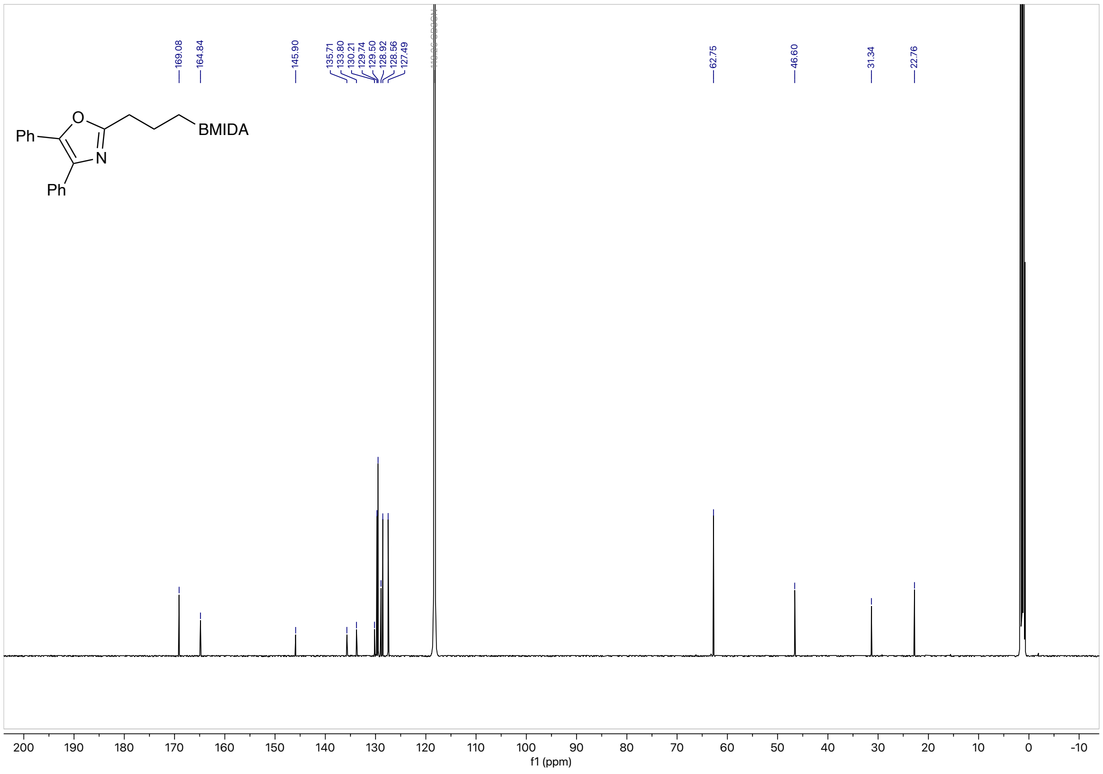

^{11}B NMR of (**3h**) (160MHz, CD_3CN)

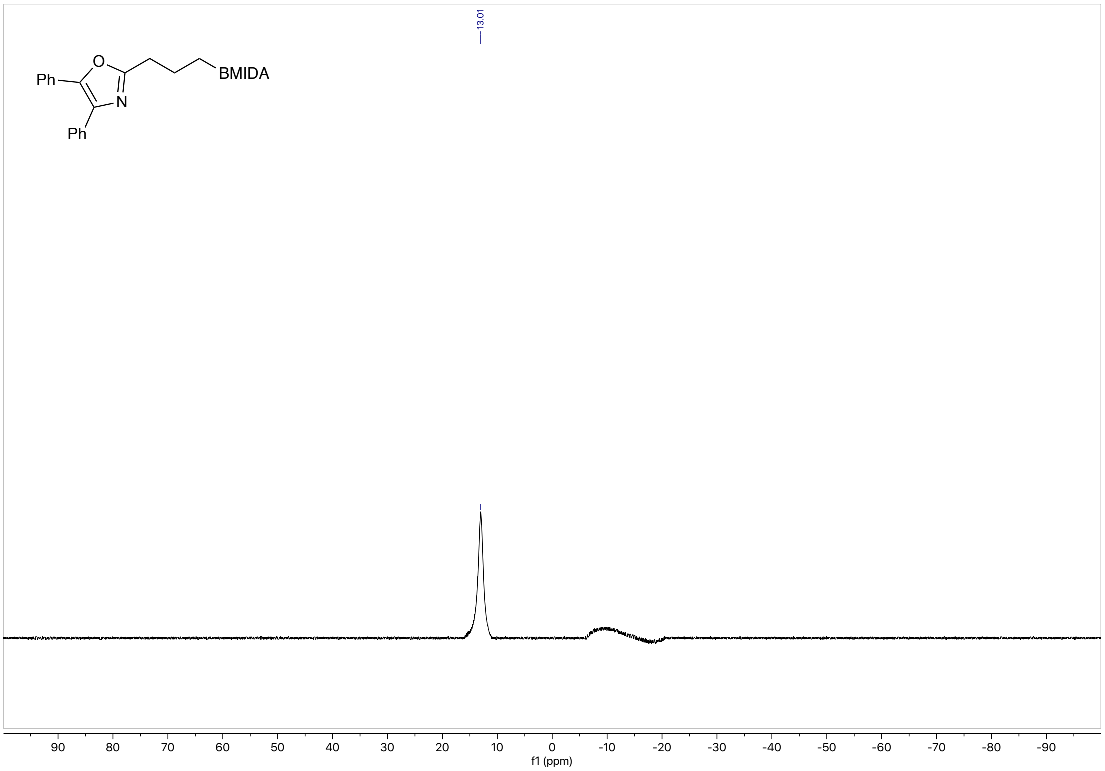

^{1}H NMR benzyl-6-methyl-1,3,6,2-dioxazaborocane-4,8-dione (**3i**) (500 MHz, CD_3CN)

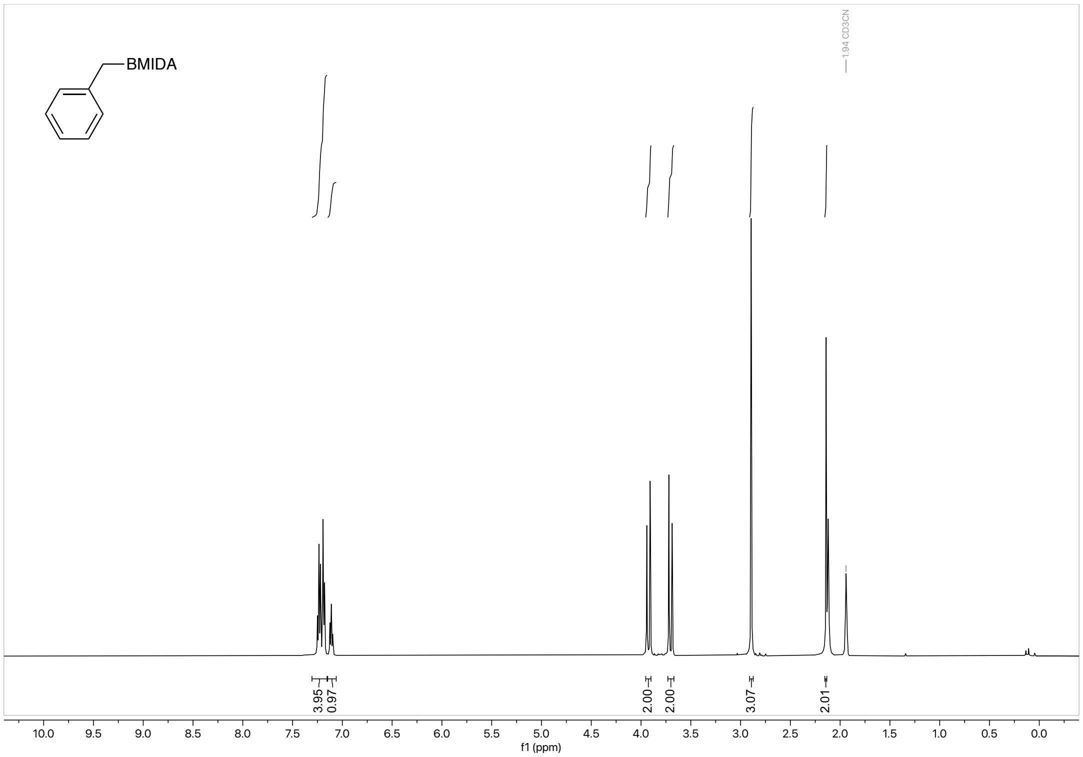

^{13}C NMR benzyl-6-methyl-1,3,6,2-dioxazaborocane-4,8-dione (**3i**) (126 MHz, CD_3CN)

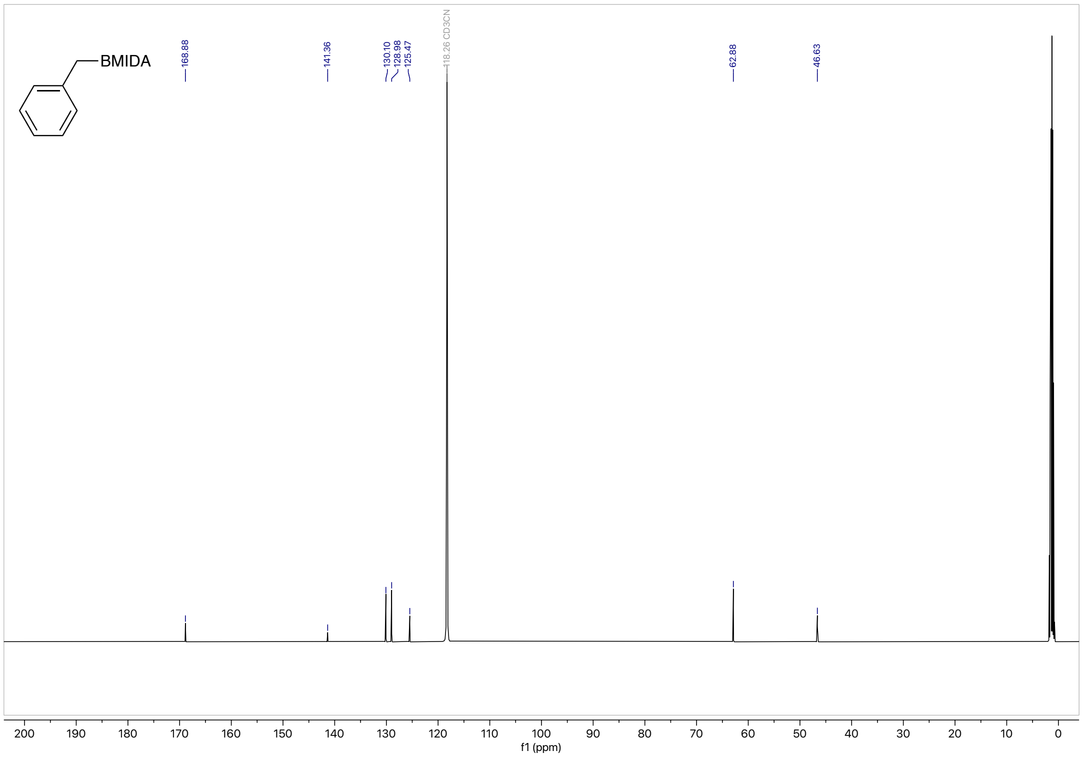

^{1}H NMR of 2-(2,4-difluorobenzyl)-6-methyl-1,3,6,2-dioxazaborocane-4,8-dione (**3j**) (500 MHz, DMSO)

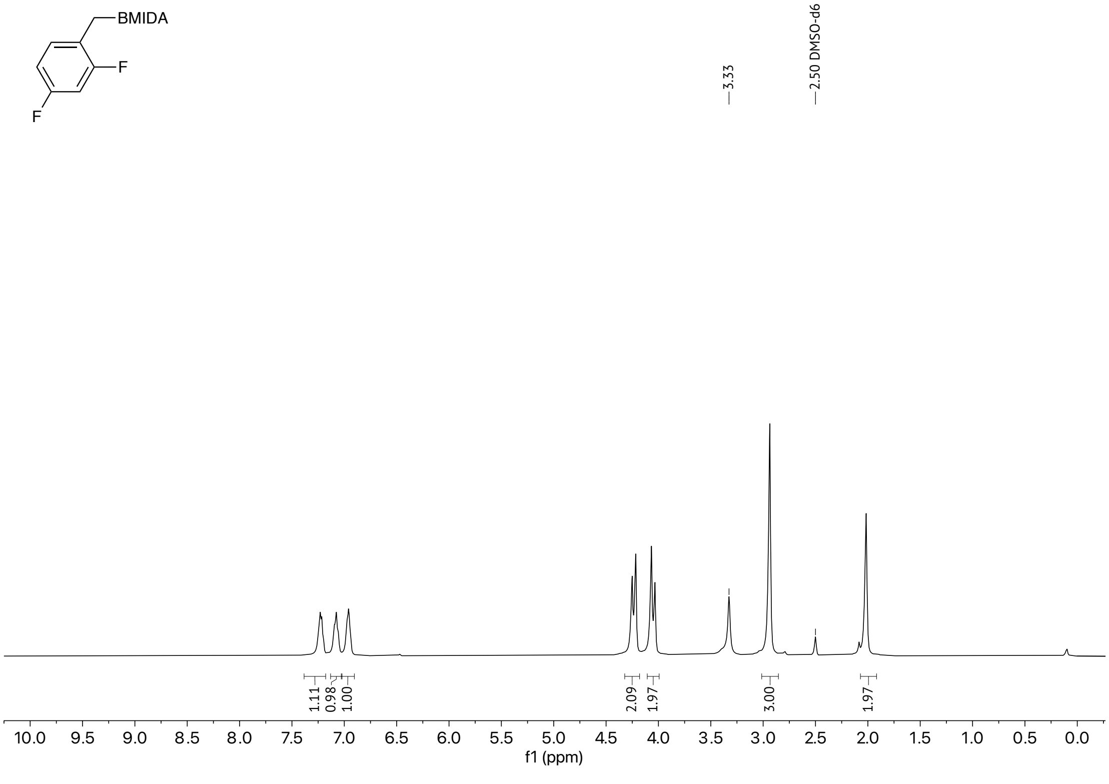

^{13}C NMR of (**3j**) (126 MHz, DMSO$_{d6}$)

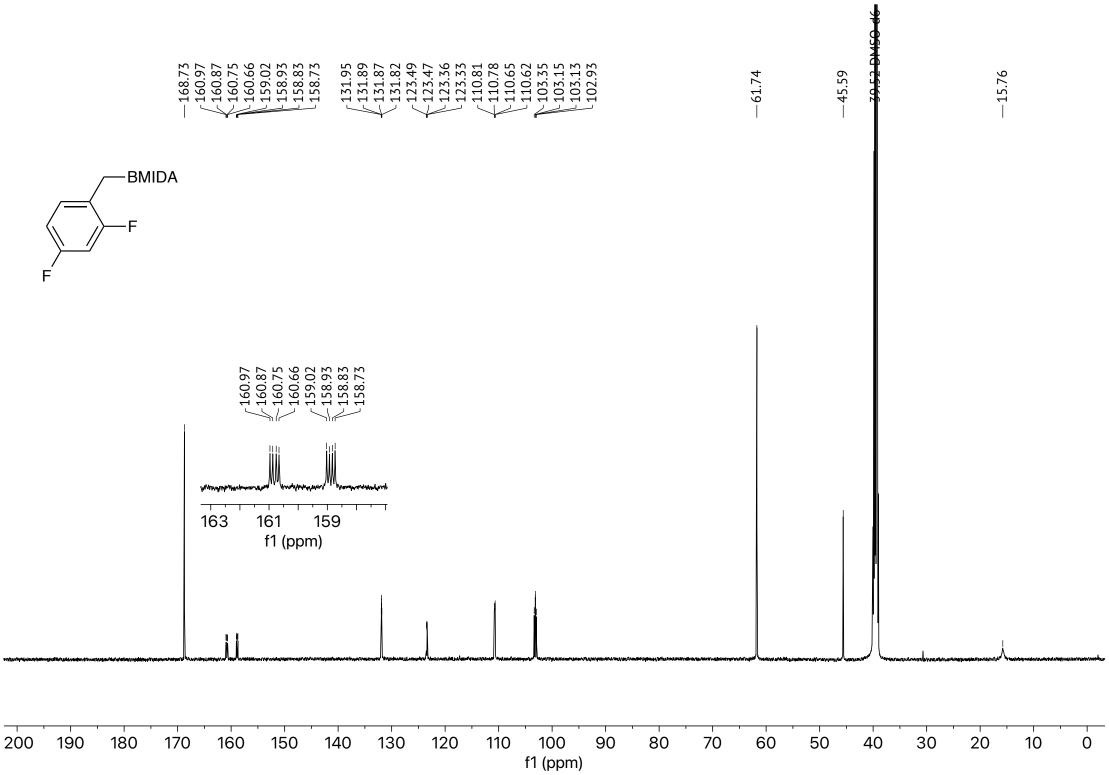

^{19}F NMR of (**3j**) (470 MHz, DMSO$_{d6}$)

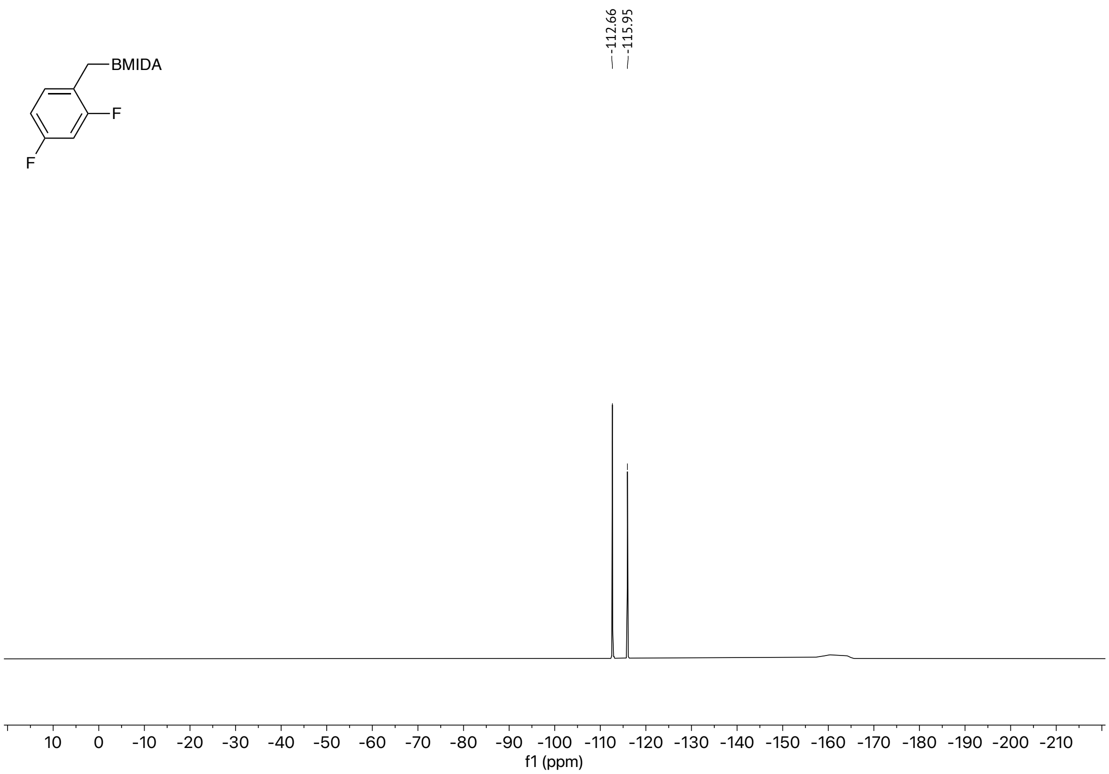

^{11}B NMR of (**3j**) (160 MHz, DMSO$_{d6}$)

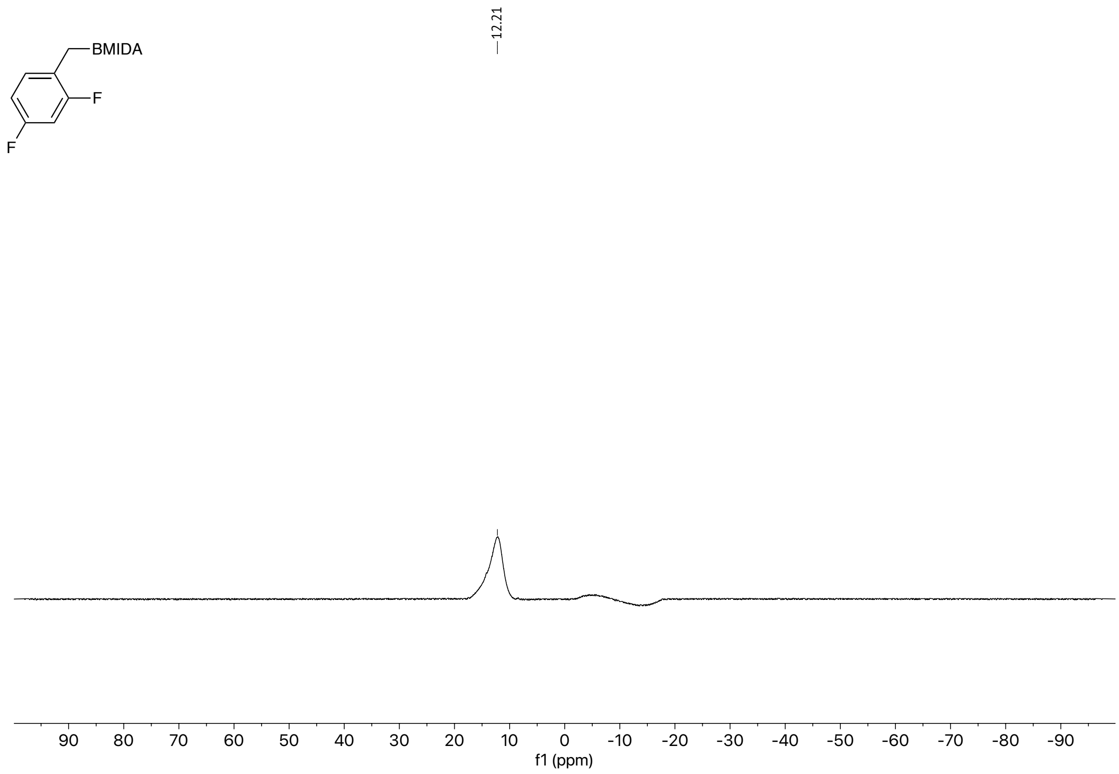

^{1}H NMR of 4-((6-methyl-4,8-dioxo-1,3,6,2-dioxazaborocan-2-yl)methyl)-N,N-dipropylbenzenesulfonamide (**3k)** (500 MHz, CD_3CN)

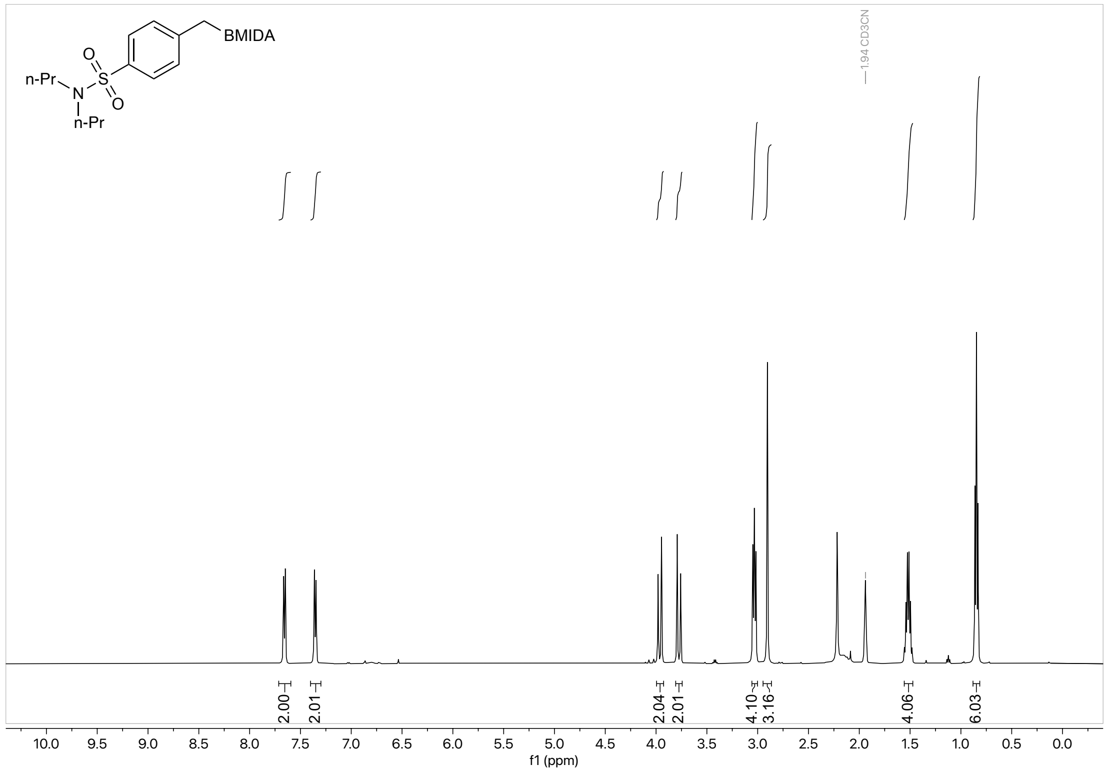

^{13}C NMR of (**3k**) (126 MHz, CD_3CN)

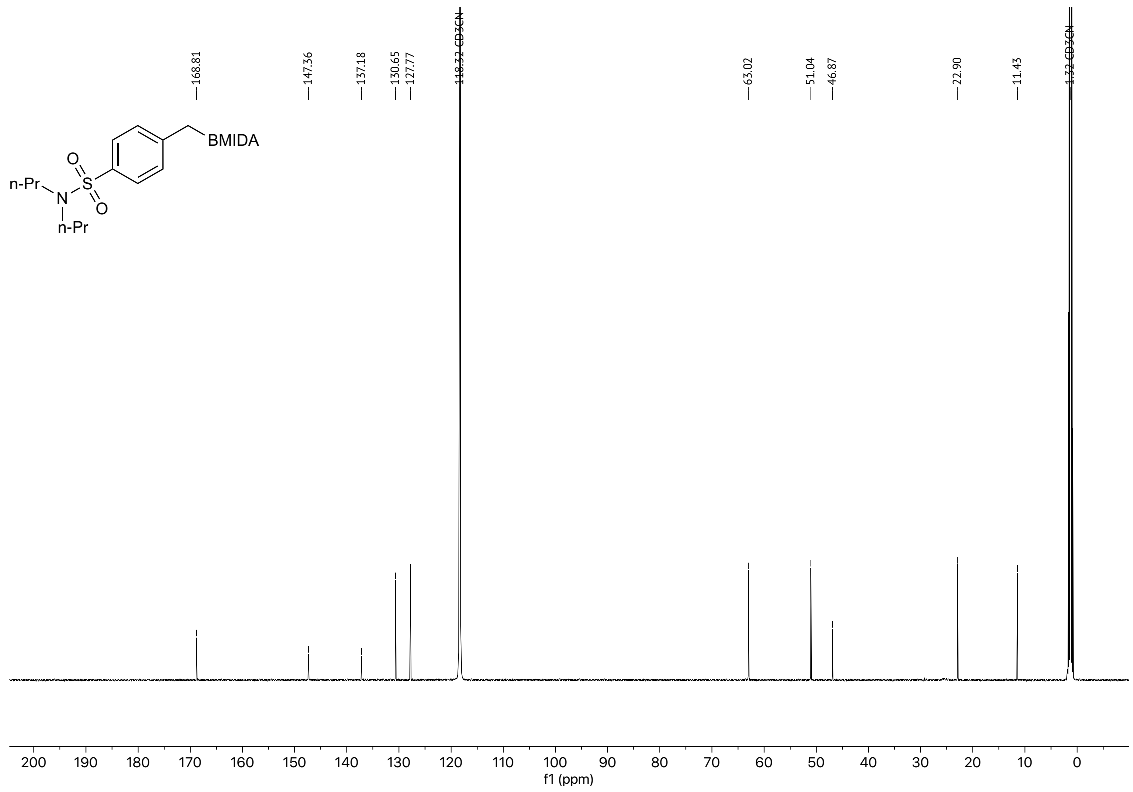

^{11}B NMR of (**3k**) (160 MHz, CD_3CN)

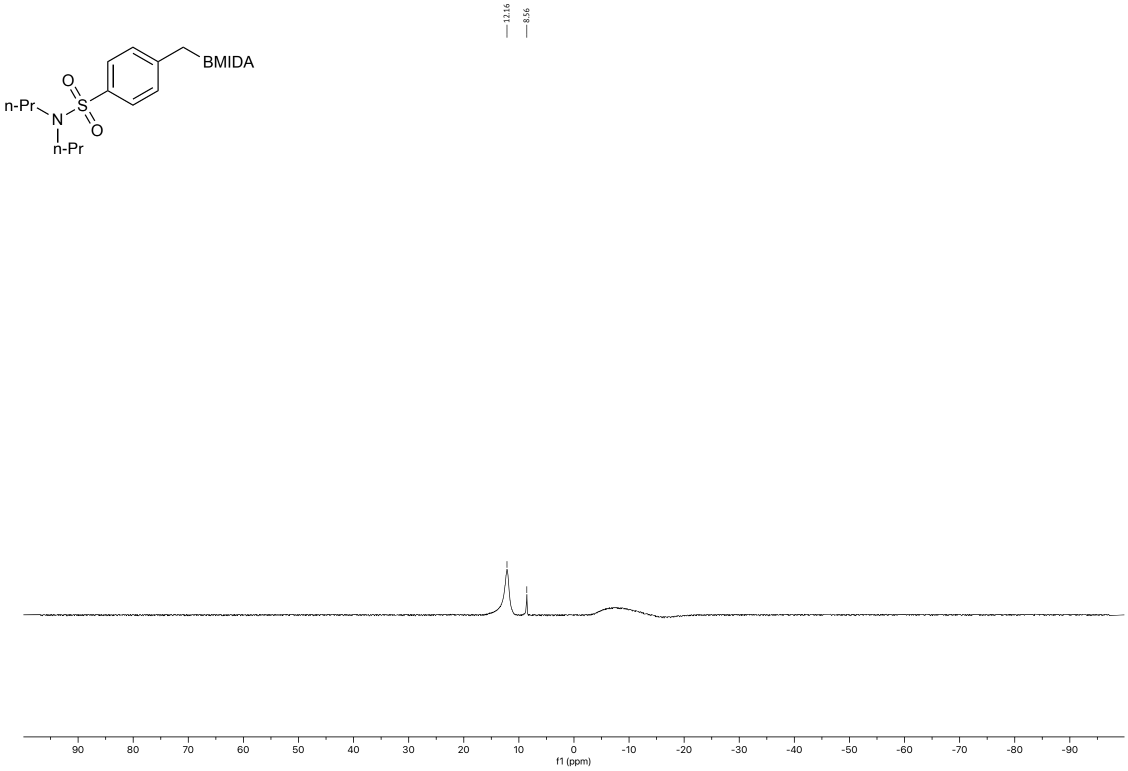

^{1}H NMR of *tert*-butyl 3-(6-methyl-4,8-dioxo-1,3,6,2-dioxazaborocan-2-yl)azetidine-1-carboxylate (**3l**) (500 MHz, CD_3CN)

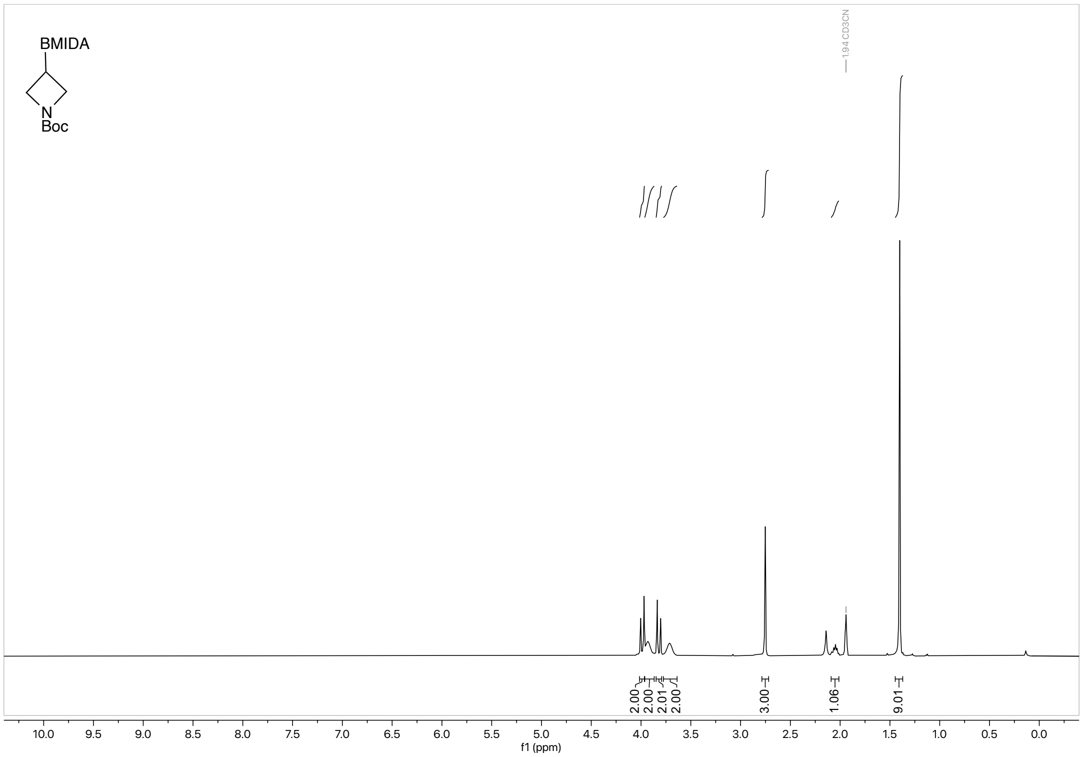

^{13}C NMR of (**3l**) (126 MHz, CD_3CN)

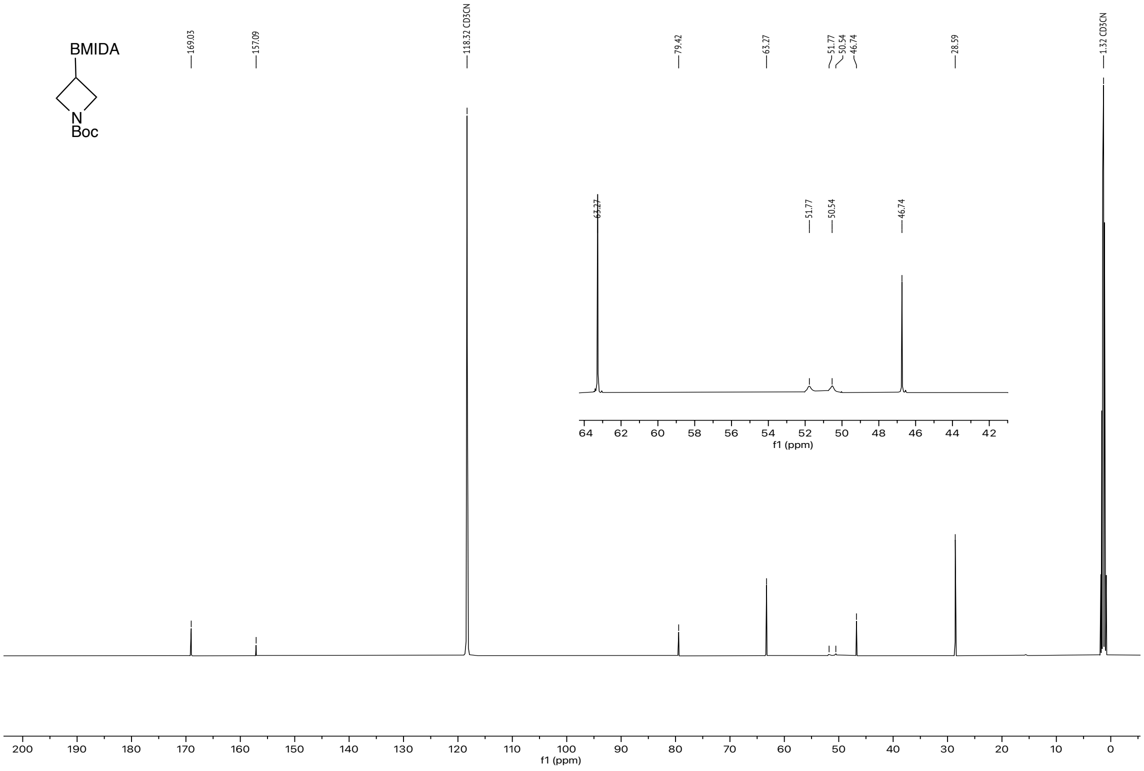

^{11}B NMR of (**3l**) (160 MHz, CD_3CN)

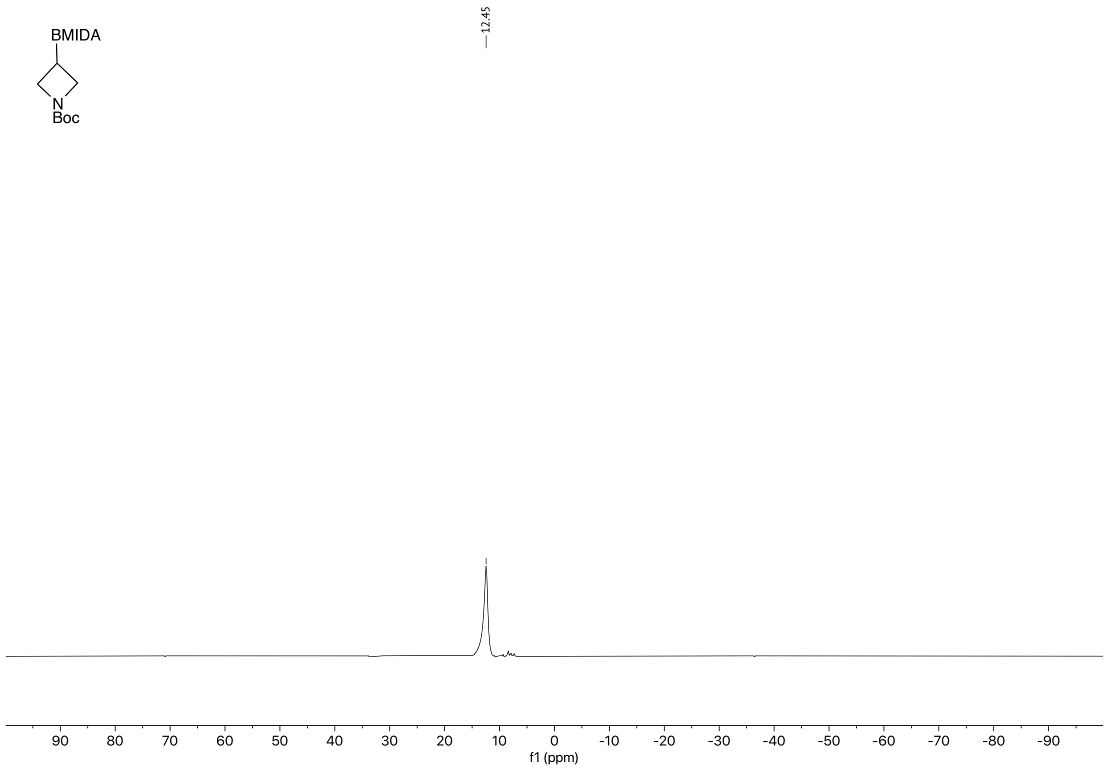

^{1}H NMR *of tert*-butyl 3-(4,4,5,5-tetraethyl-1,3,2-dioxaborolan-2-yl)pyrrolidine-1-carboxylate (**3m**) (500 MHz, $CDCl_3$)

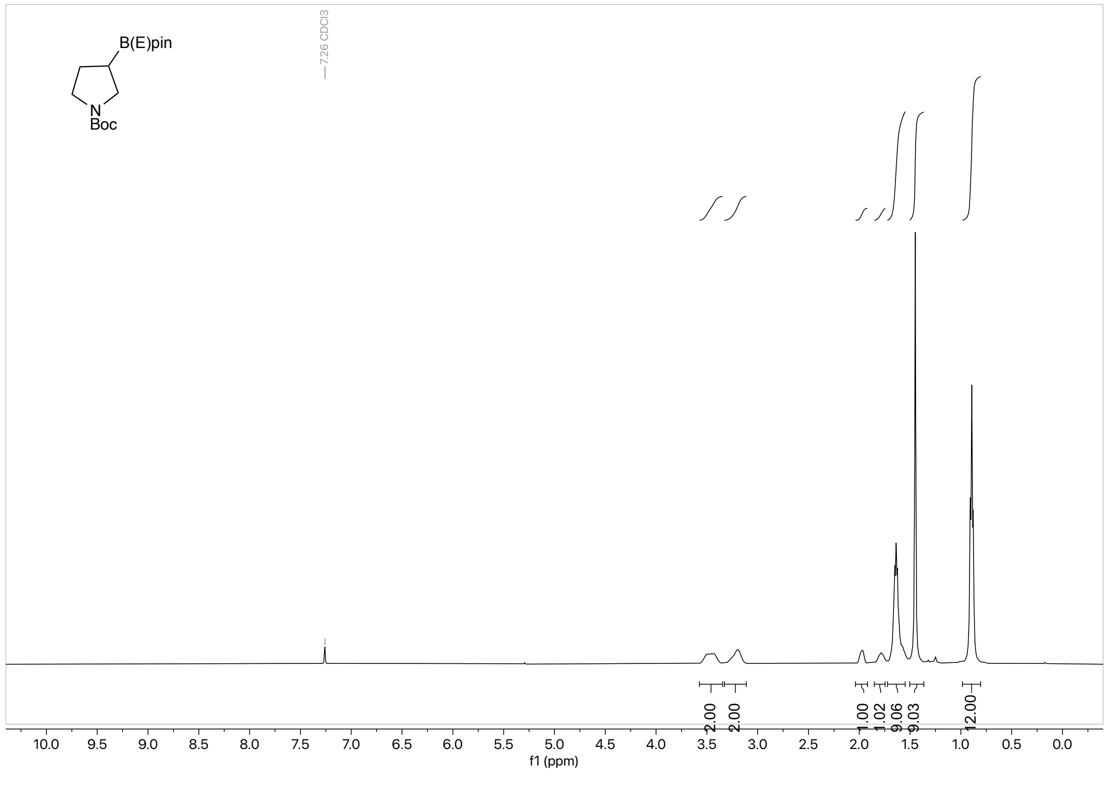

^{13}C NMR (**3m**) (126 MHz, $CDCl_3$)

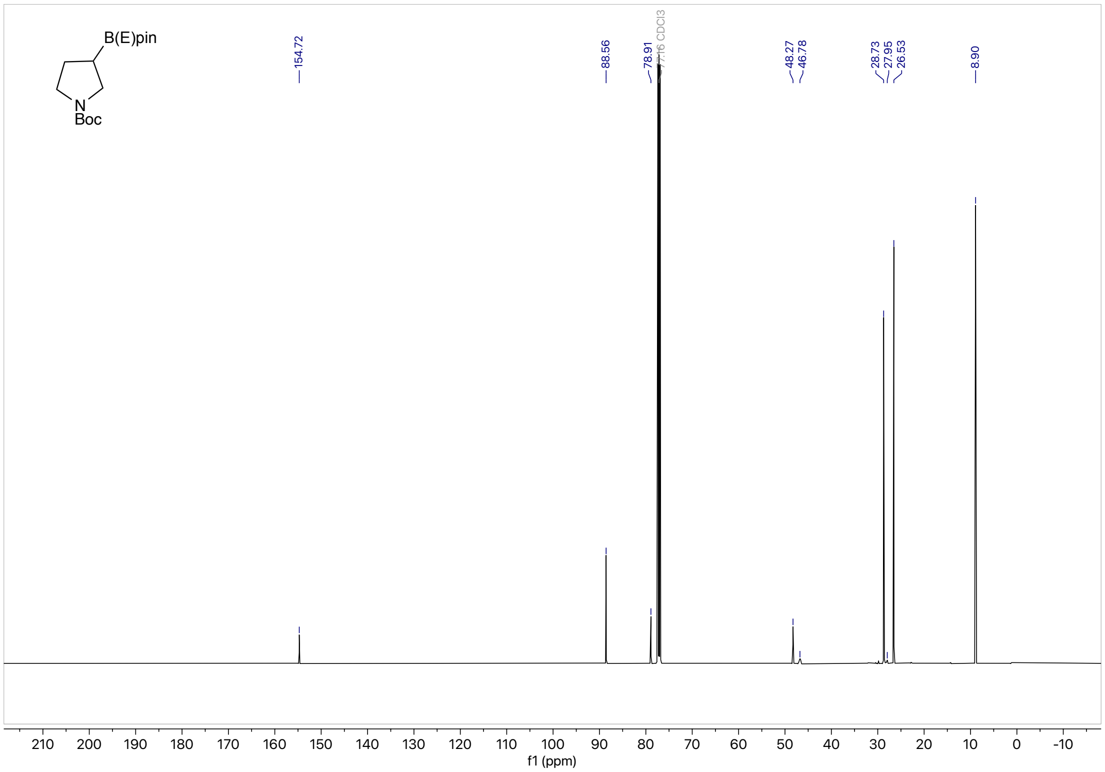

^{11}B NMR of (**3m**) (160 MHz, CDCl3)

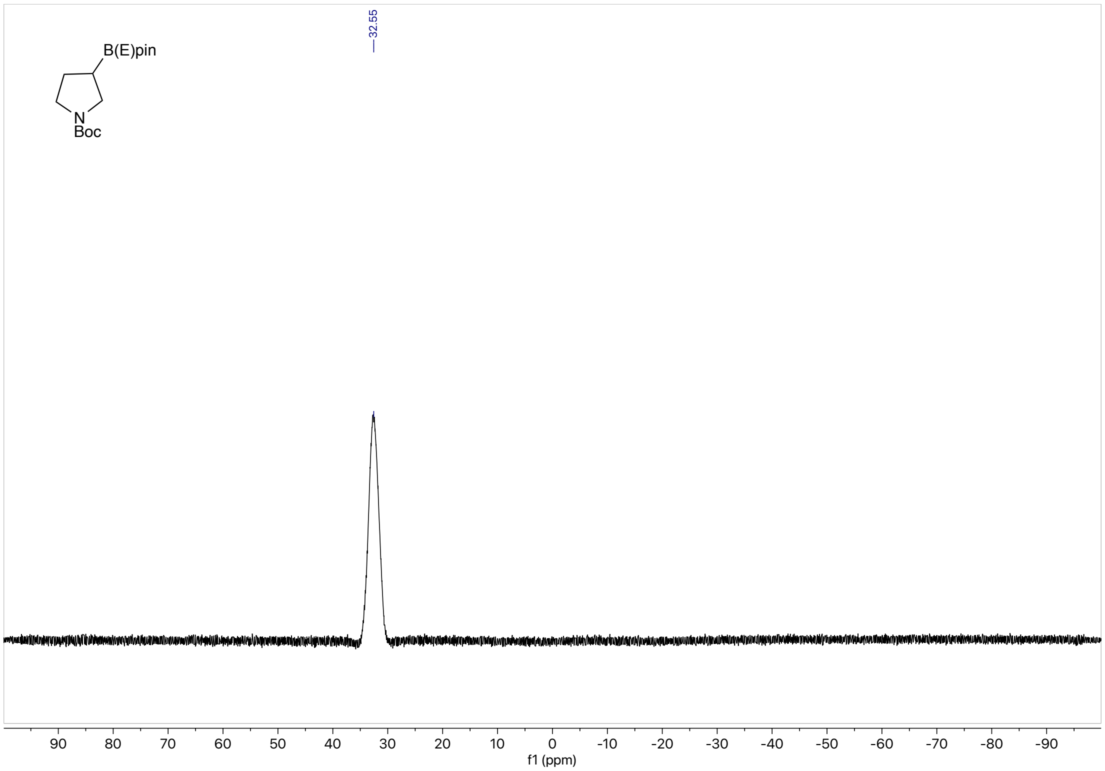

^{1}H NMR of *tert*-butyl 4-(4,4,5,5-tetraethyl-1,3,2-dioxaborolan-2-yl)piperidine-1-carboxylate (**3n)** (500 MHz, $CDCl_3$)

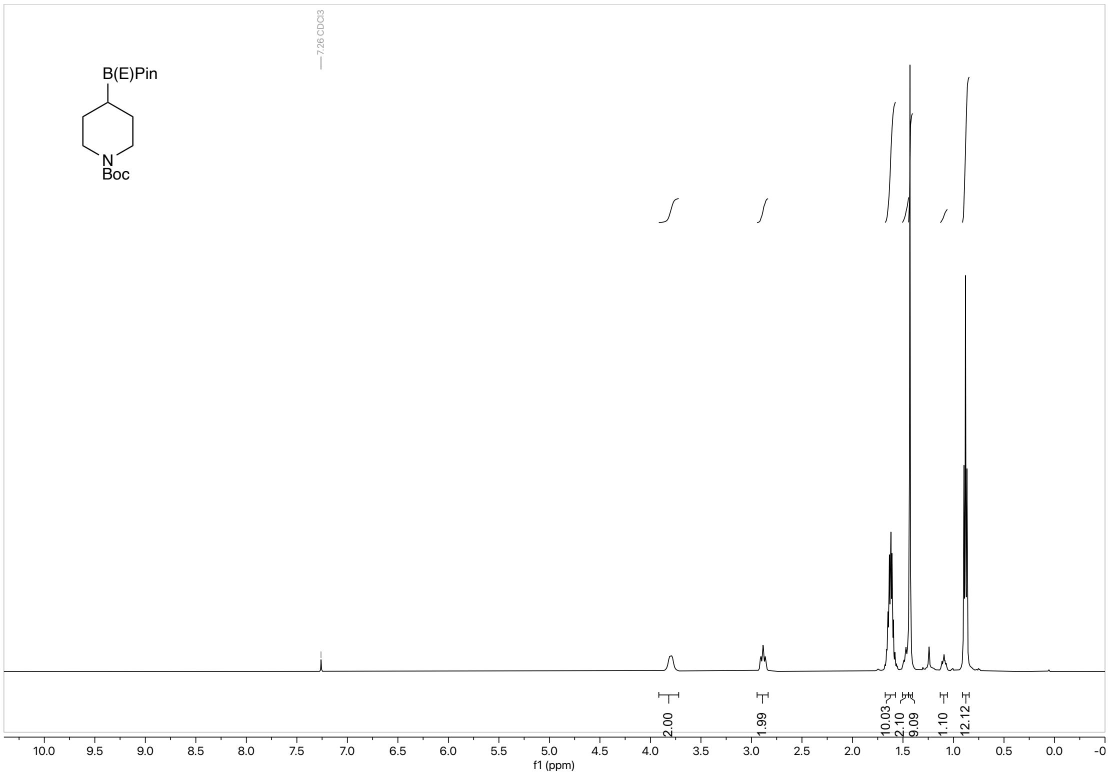

^{13}C NMR of (**3n)** (126 MHz, $CDCl_3$)

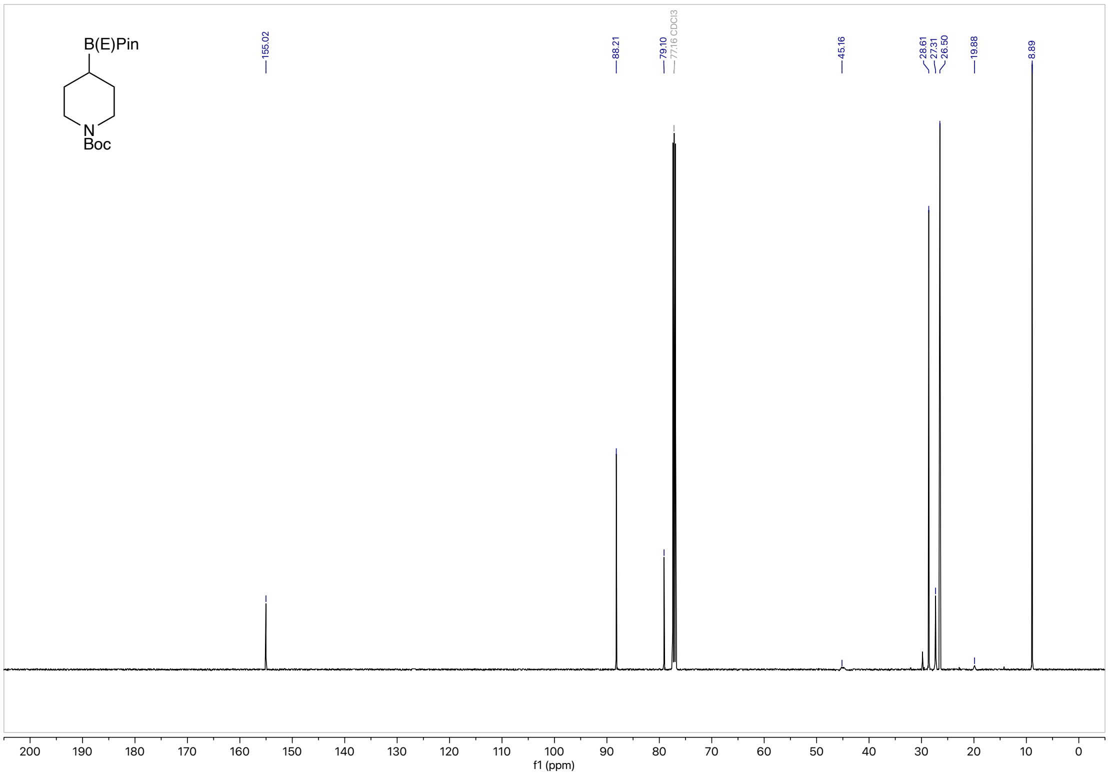

^{11}B NMR of (**3n**) (160 MHz, $CDCl_3$)

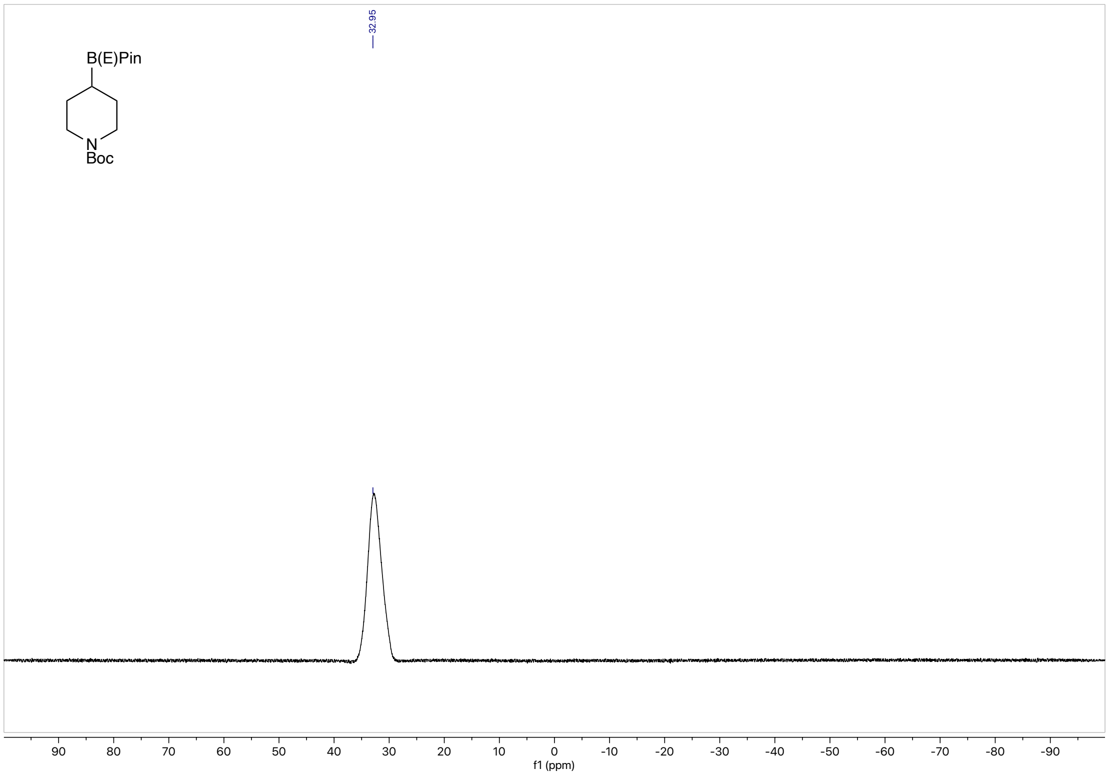

^{1}H NMR of *tert*-butyl 3-(4,4,5,5-tetraethyl-1,3,2-dioxaborolan-2-yl)azepane-1-carboxylate (**3o)** (500 MHz, $CDCl_3$)

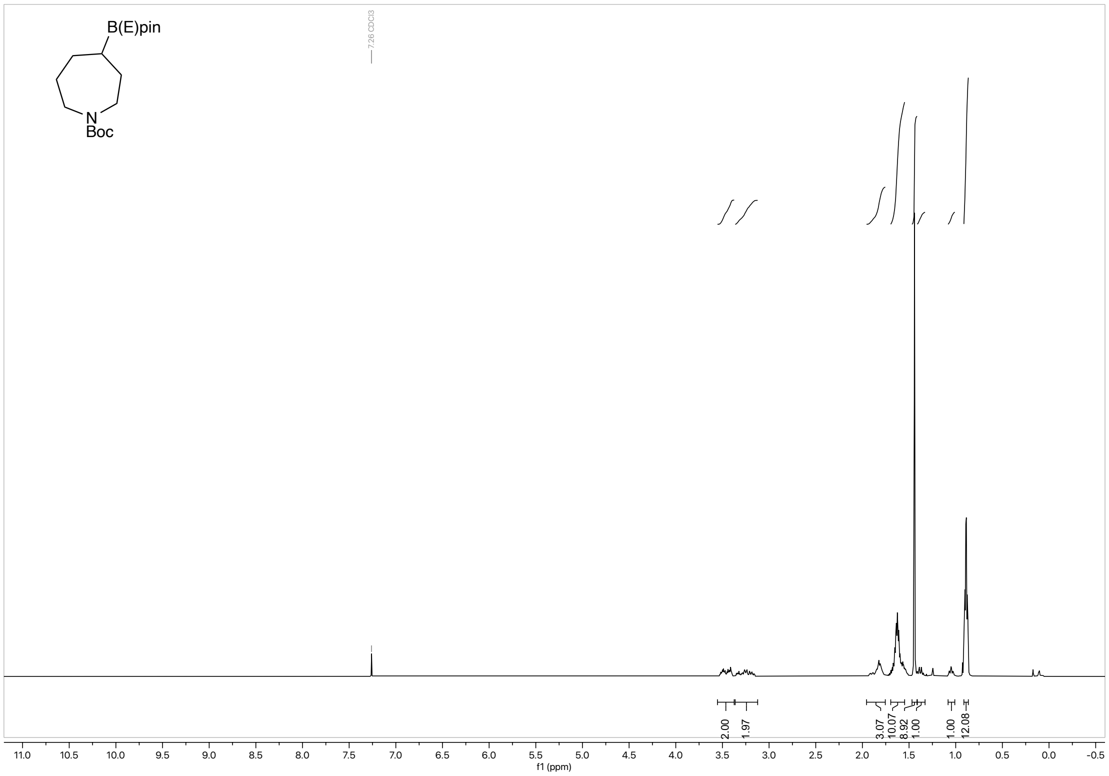

^{13}C NMR of (**3o**) (126 MHz, $CDCl_3$)

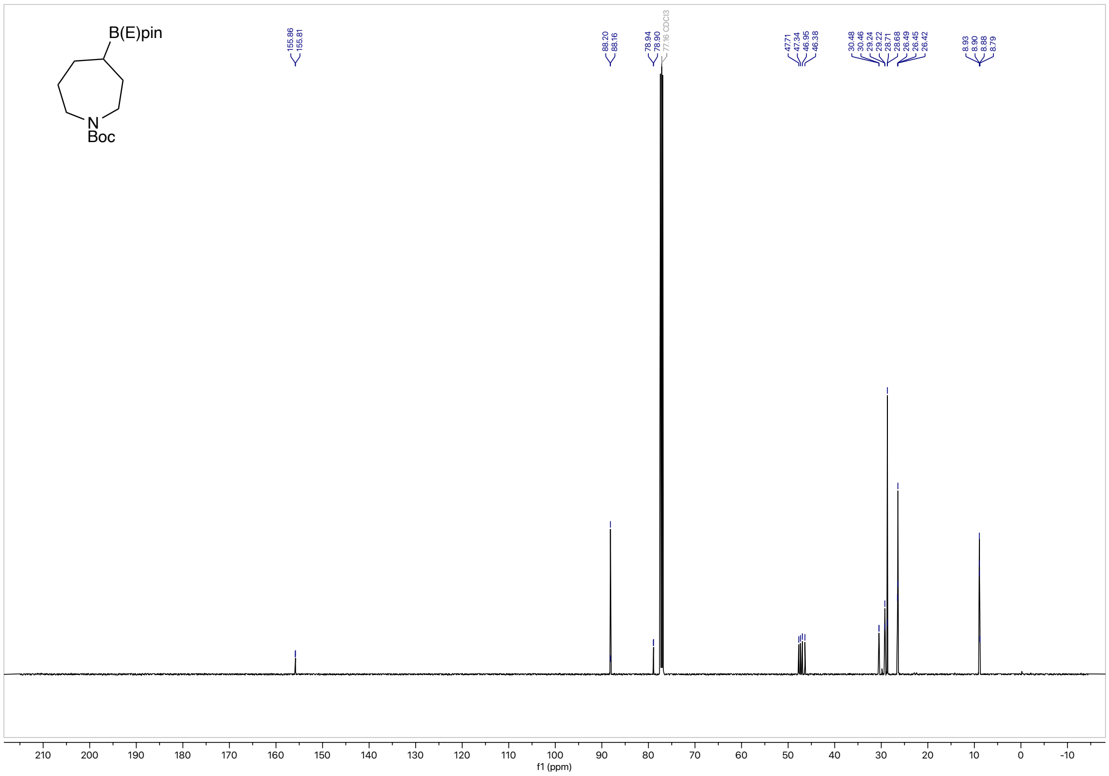

^{11}B NMR of (**3o**) (160 MHz, $CDCl_3$)

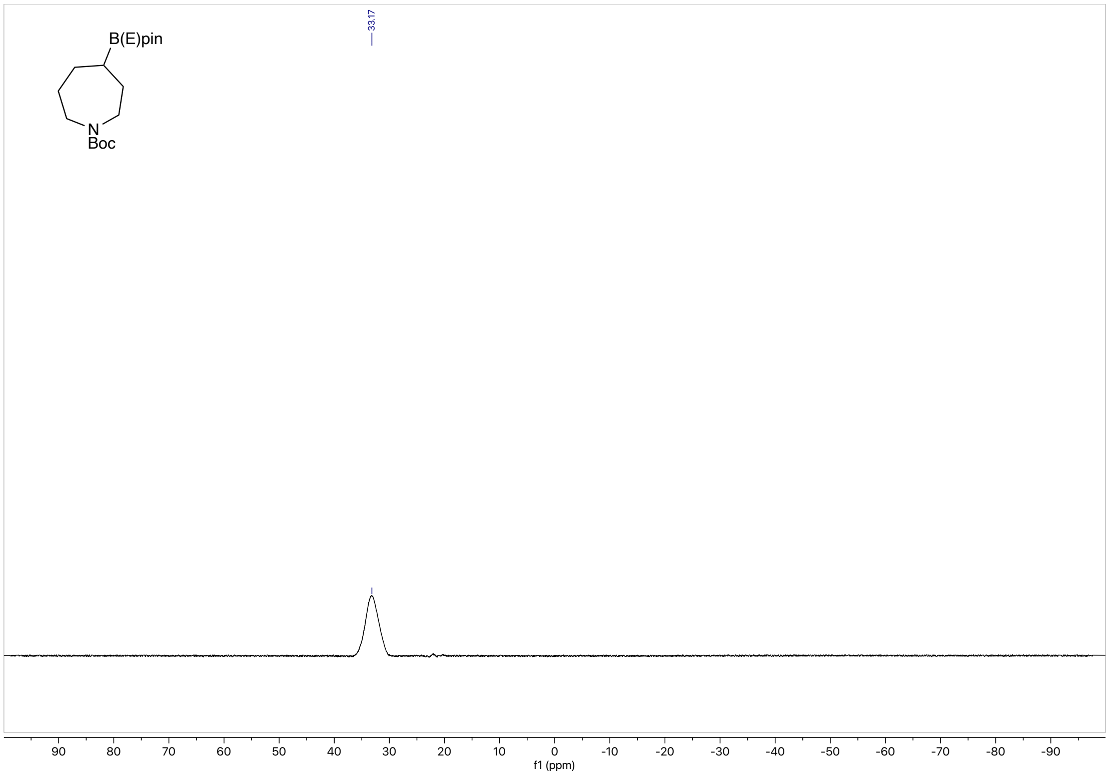

^{1}H NMR of 6-methyl-2-(tetrahydrothiophen-3-yl)-1,3,6,2-dioxazaborocane-4,8-dione (**3p**) (500 MHz, DMSO)

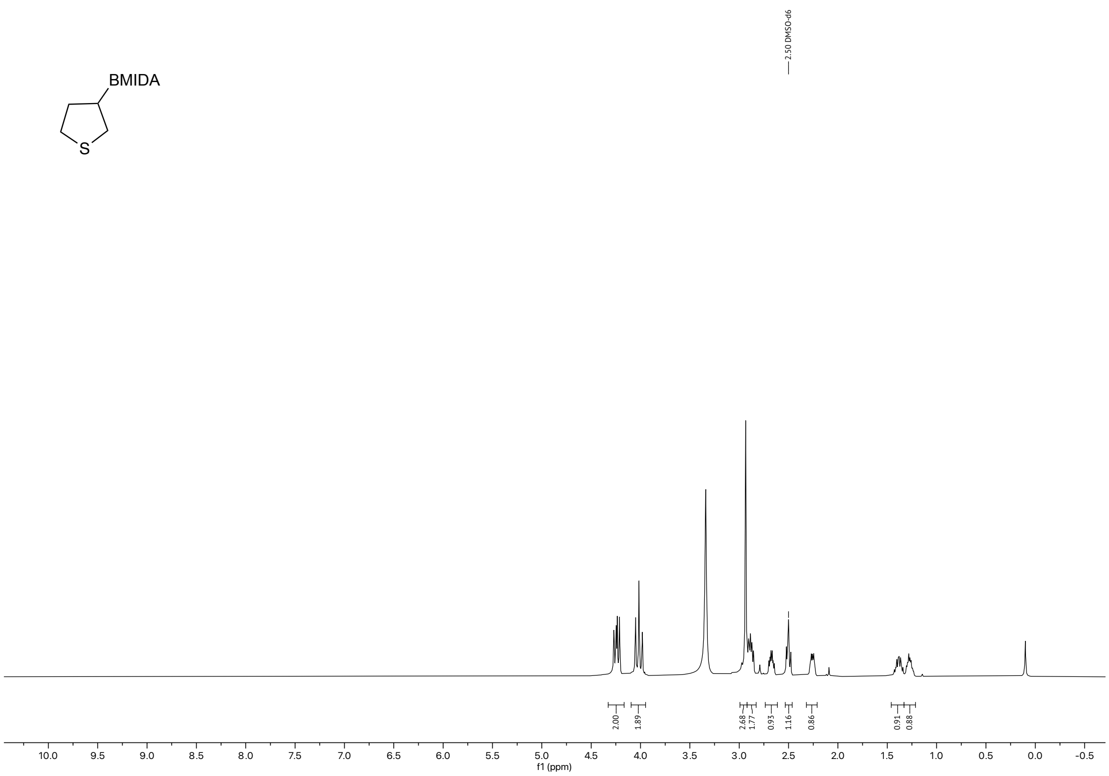

^{13}C NMR of (**3p**) (126 MHz, DMSO)

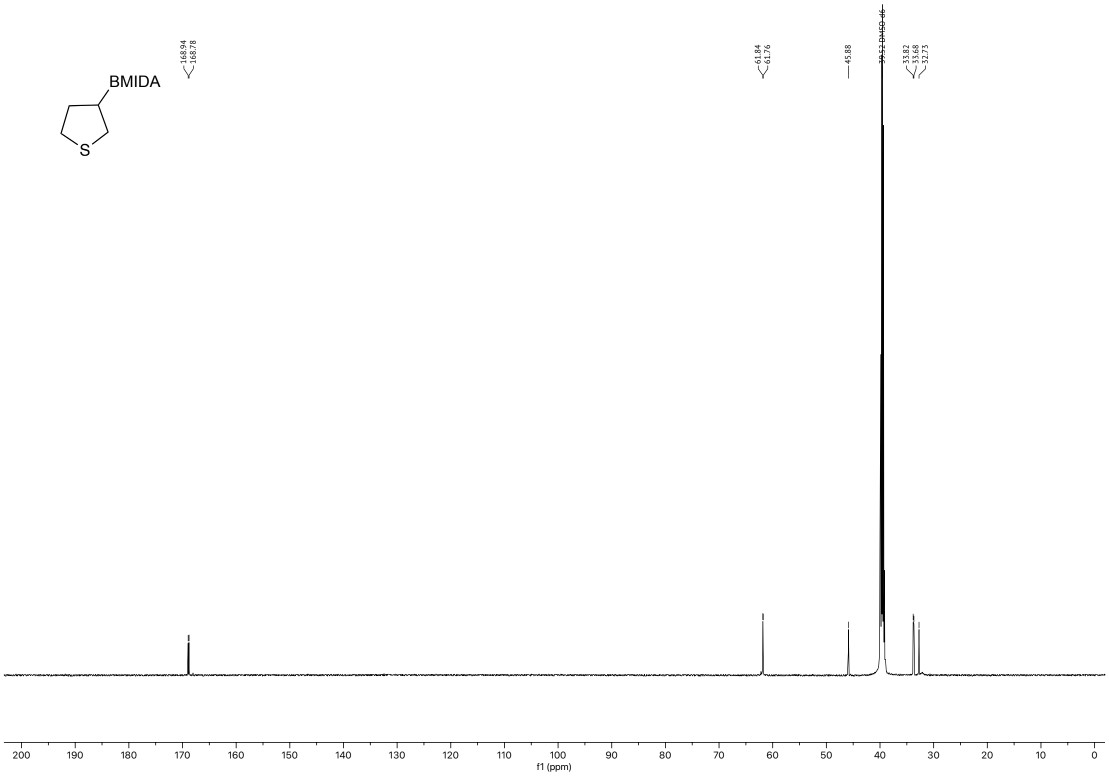

^{11}B NMR of (**3p**) (160 MHz, DMSO)

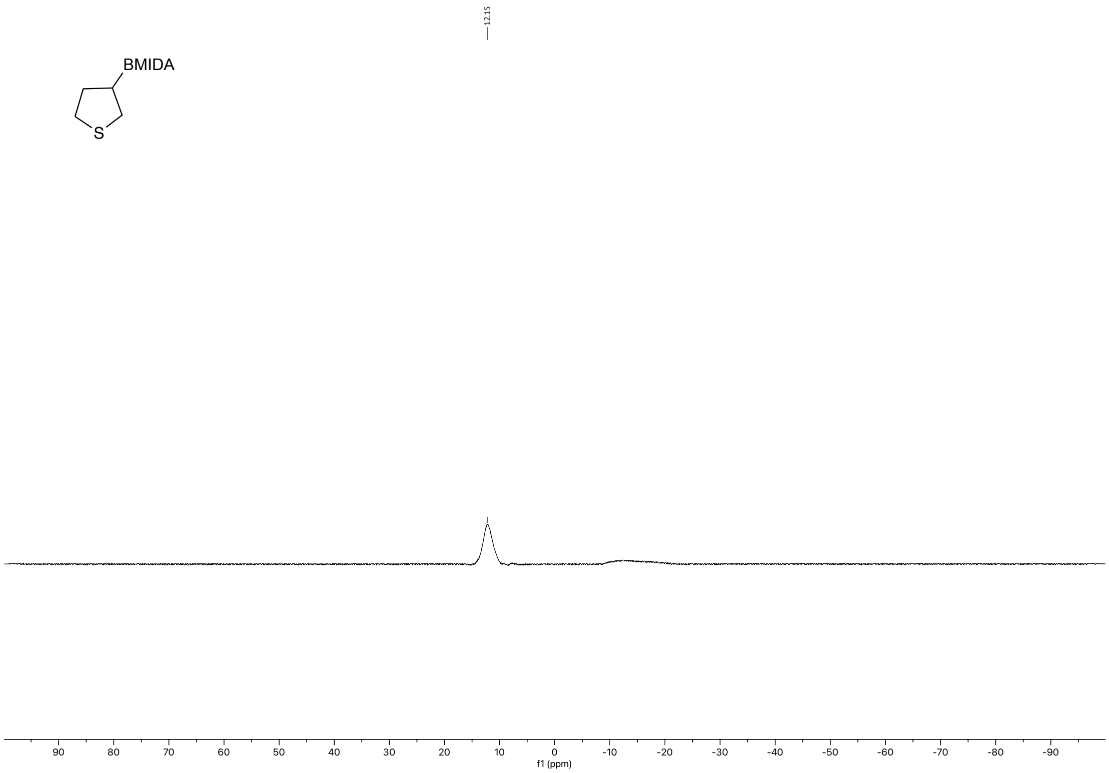

^{1}H NMR of 4,4,5,5-tetraethyl-2-(tetrahydro-2H-pyran-4-yl)-1,3,2-dioxaborolane **(3q)** (500 MHz, $CDCl_3$)

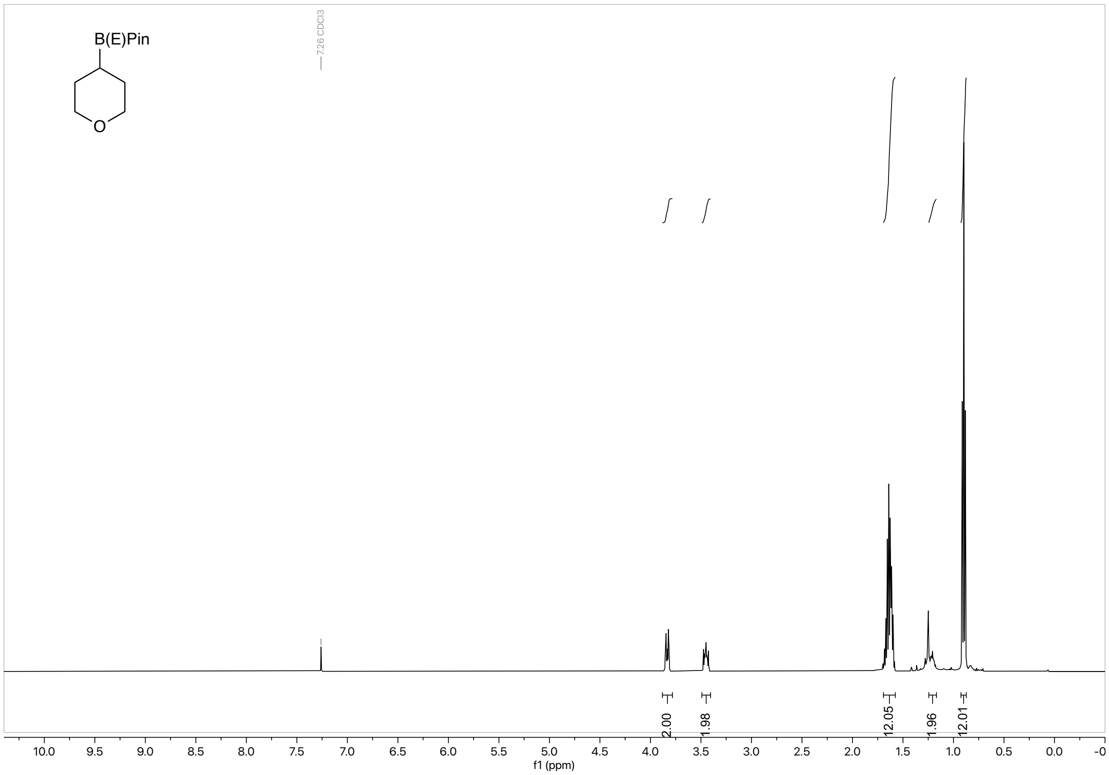

^{13}C NMR of **(3q)** (126 MHz, $CDCl_3$)

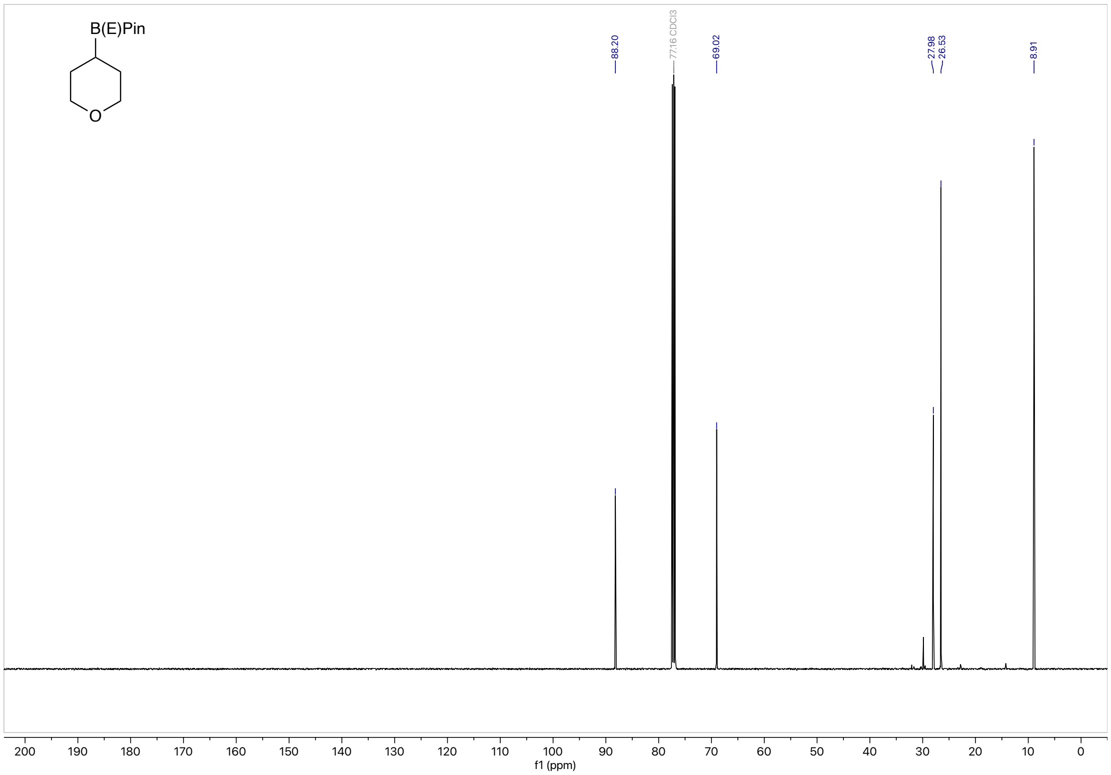

^{11}B NMR of **(3q)** (160 MHz, $CDCl_3$)

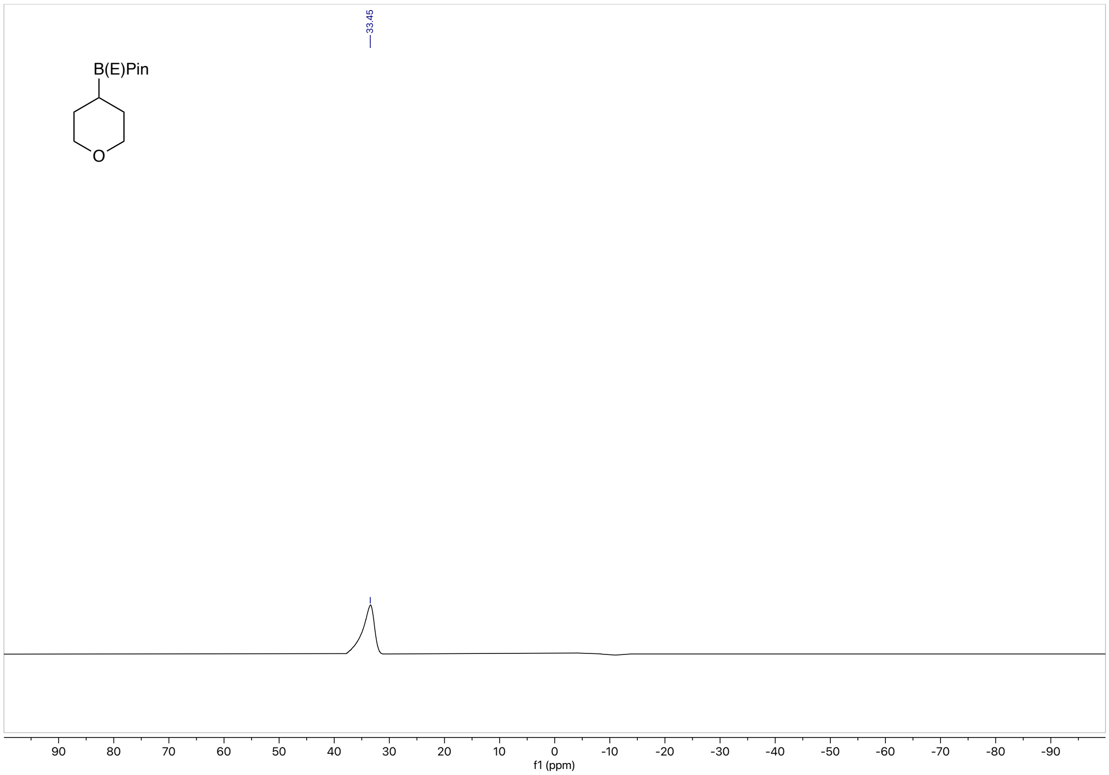

^{1}H NMR of 6-methyl-2-(1-phenylpropan-2-yl)-1,3,6,2-dioxazaborocane-4,8-dione **(3r)** (500 MHz, DMSO)

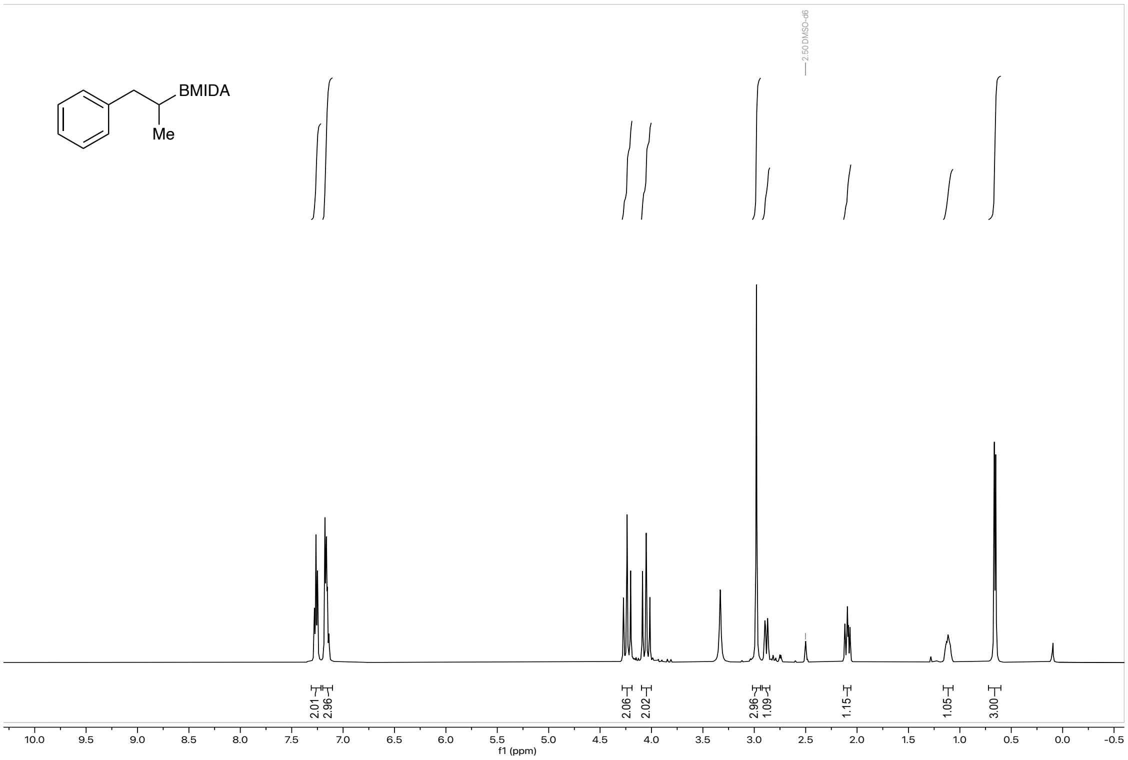

^{13}C NMR of **(3r)** (126 MHz, DMSO)

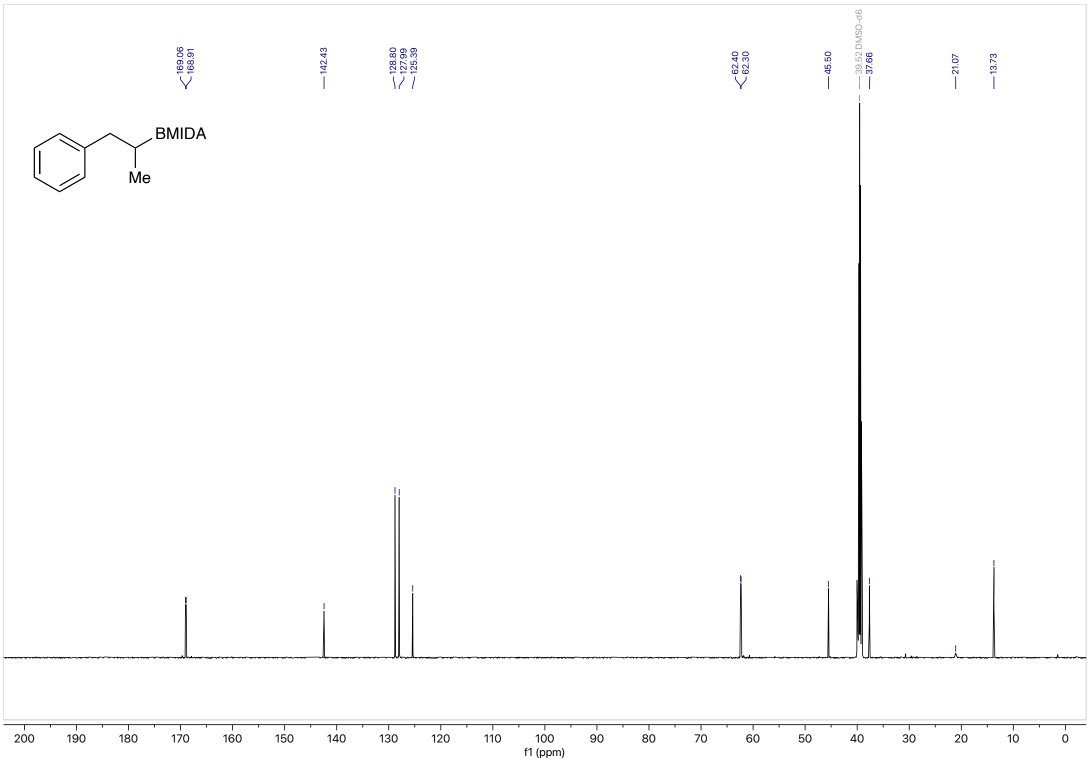

^{11}B NMR of **(3r)** (160 MHz, DMSO)

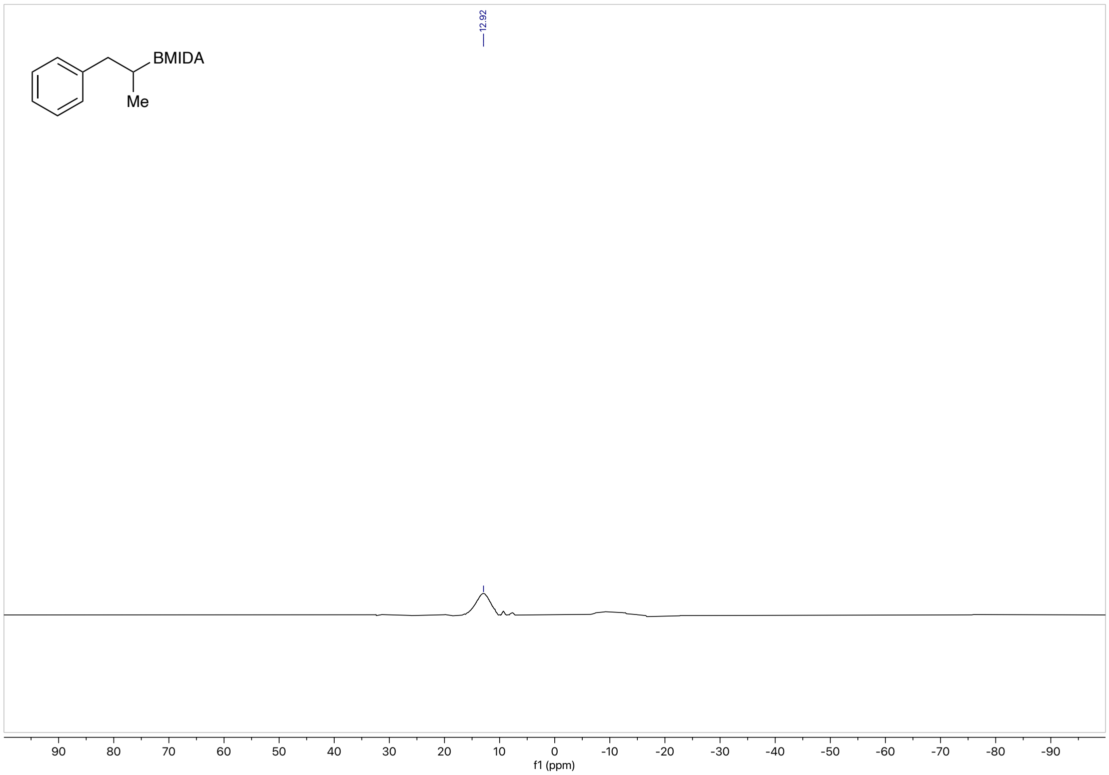

^{1}H NMR of 2-(2,3-dihydro-1*H*-inden-2-yl)-6-methyl-1,3,6,2-dioxazaborocane-4,8-dione **(3s)** (500 MHz, DMSO **)**

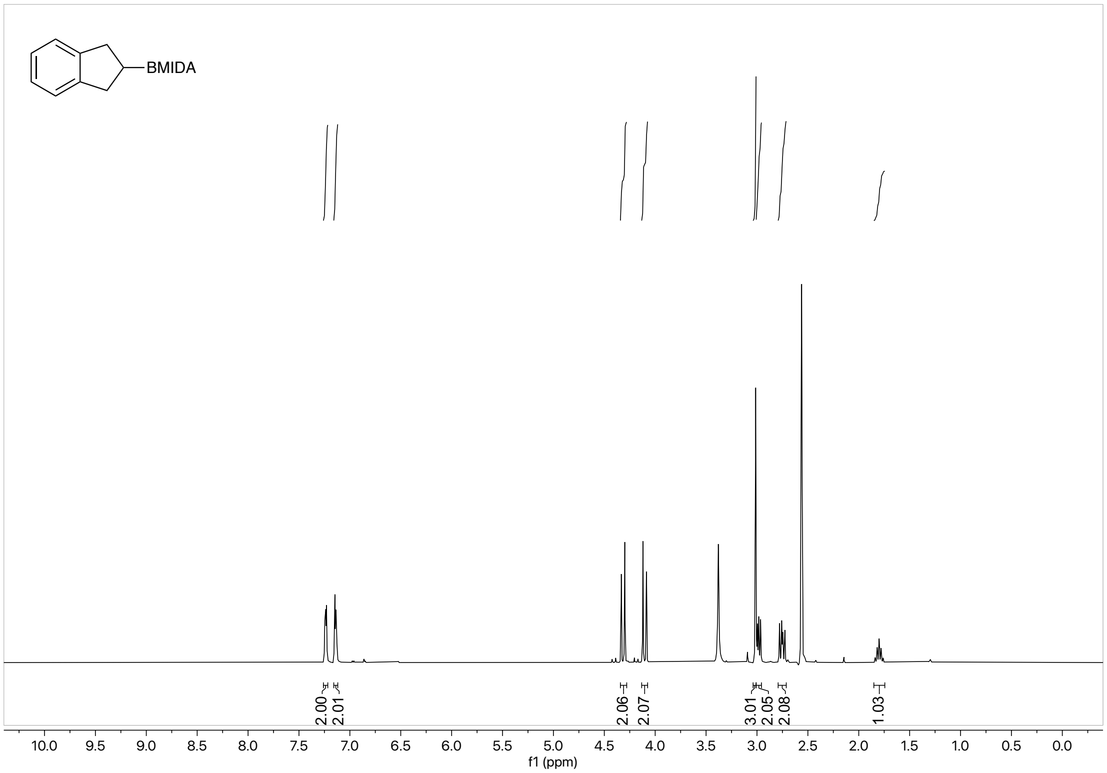

^{13}C NMR of **(3s)** (126 MHz, DMSO**)**

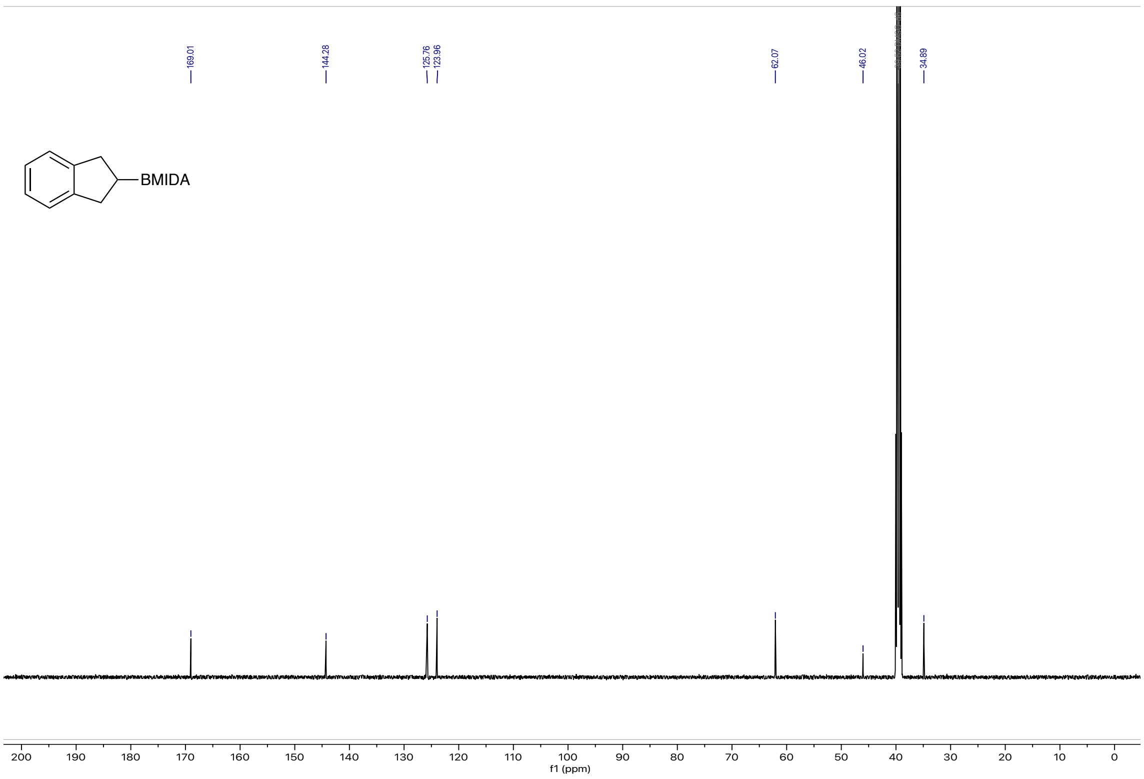

^{11}B NMR of **(3s)** (160 MHz, DMSO**)**

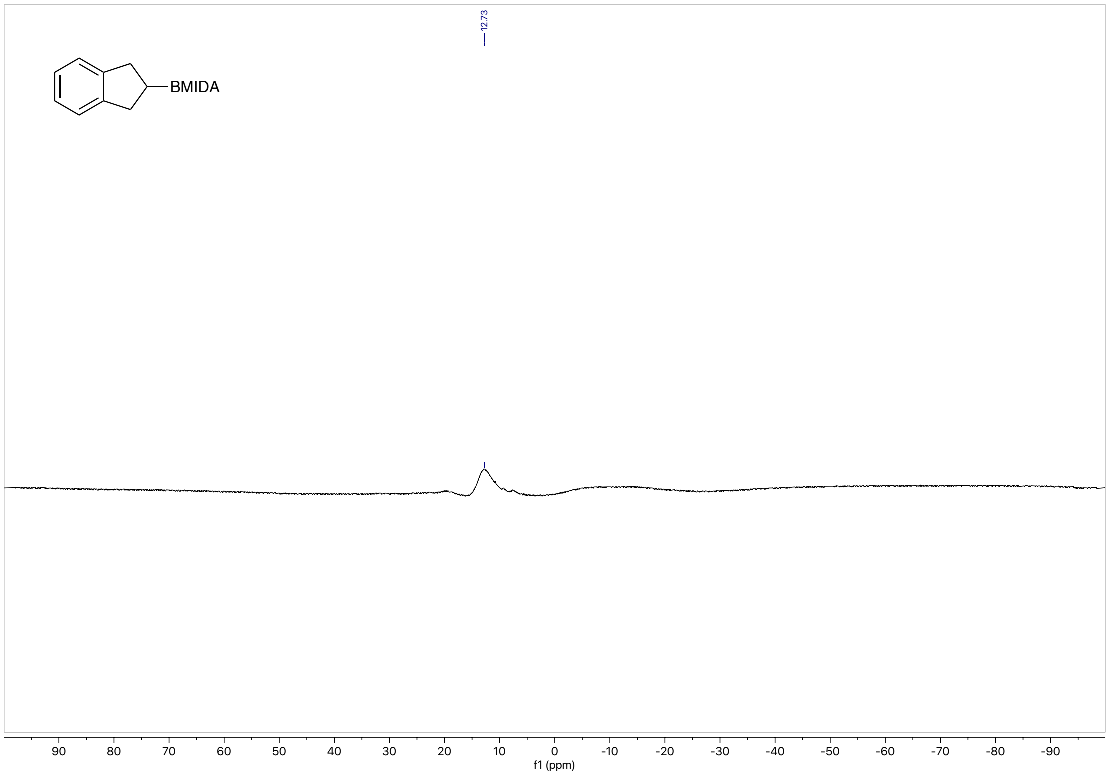

^{1}H NMR of 6-methyl-2-(1-(pyrimidin-2-yl)piperidin-4-yl)-1,3,6,2-dioxazaborocane-4,8-dione **(3t)** (500 MHz, CD_3CN)

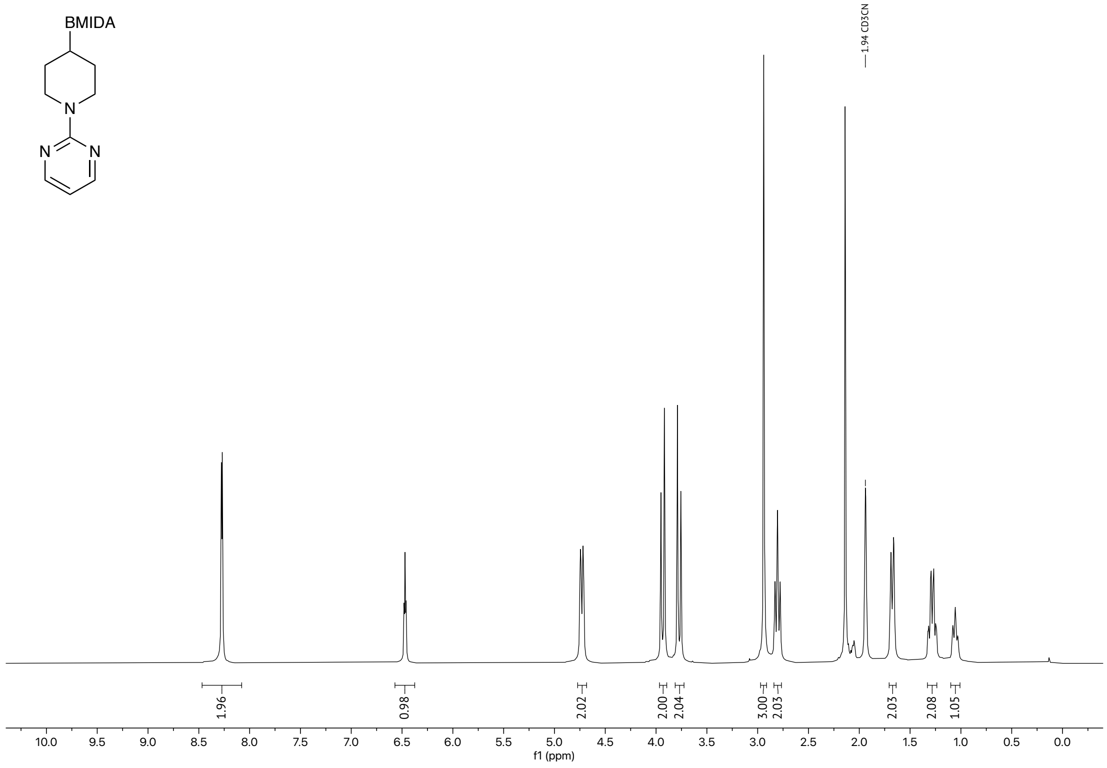

^{13}C NMR of **(3t)** (126 MHz, CD_3CN)

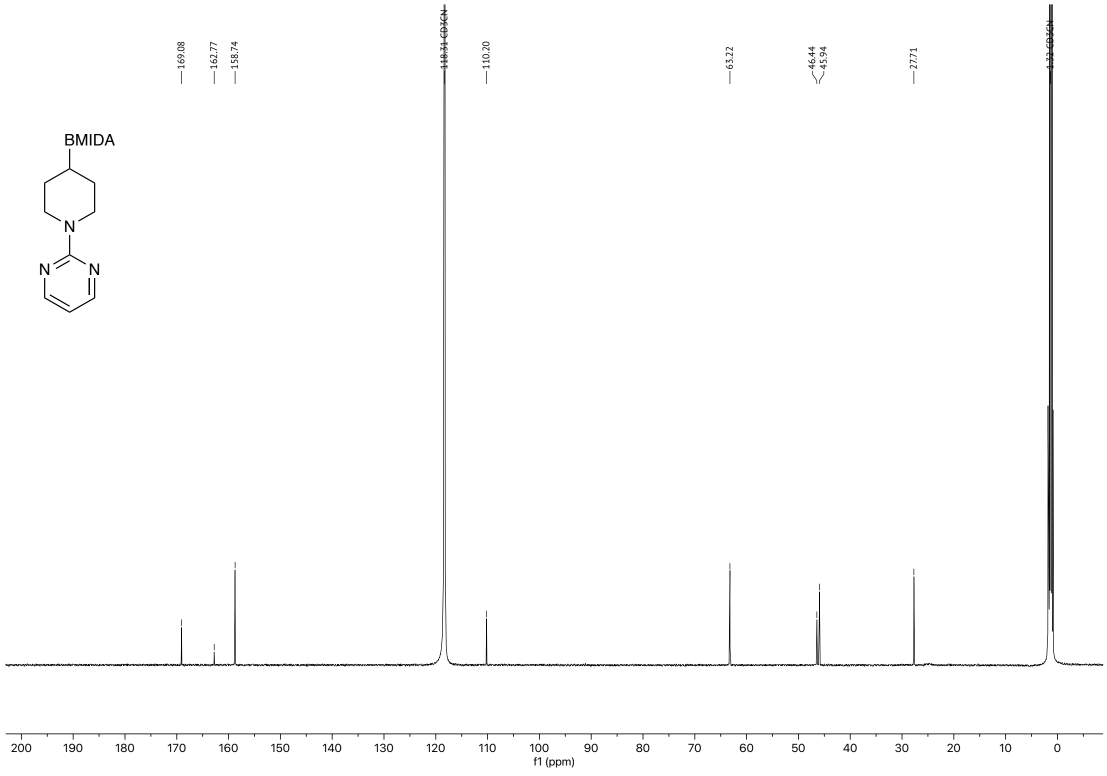

^{11}B NMR of **(3t)** (160MHz, CD_3CN)

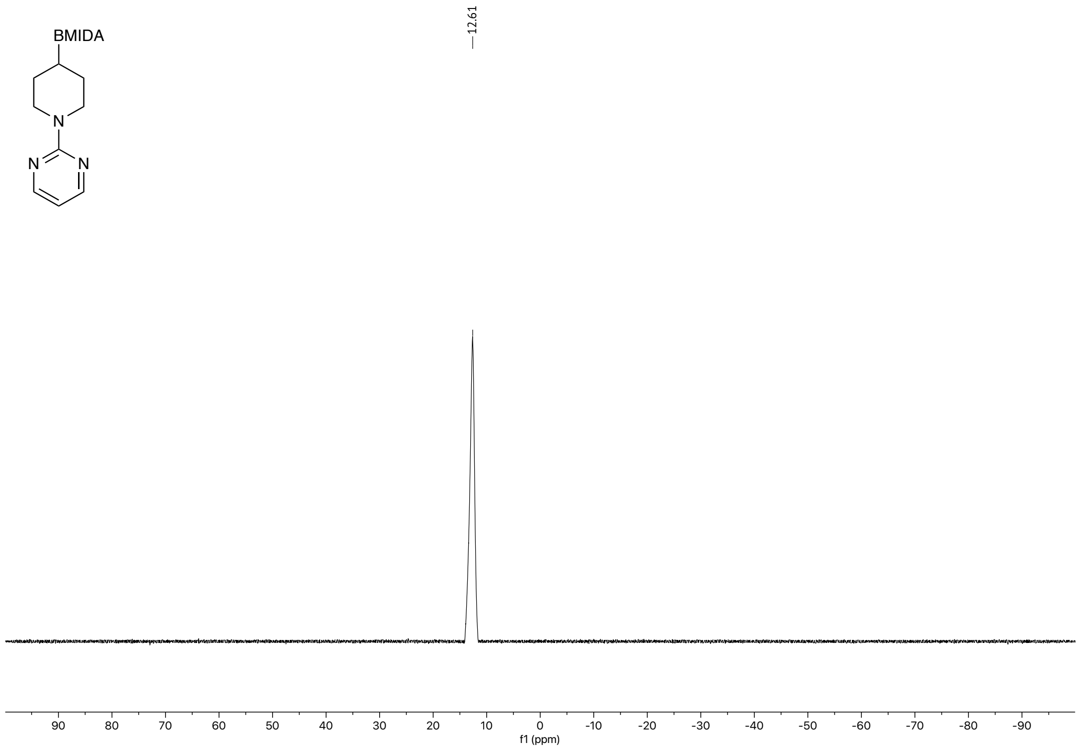

^{1}H NMR of (4-(4,4,5,5-tetraethyl-1,3,2-dioxaborolan-2-yl)piperidin-1-yl)(2-(trizfluoromethyl)pyridin-3-yl)methanone **(3u)** (160 MHz, $CDCl_3$)

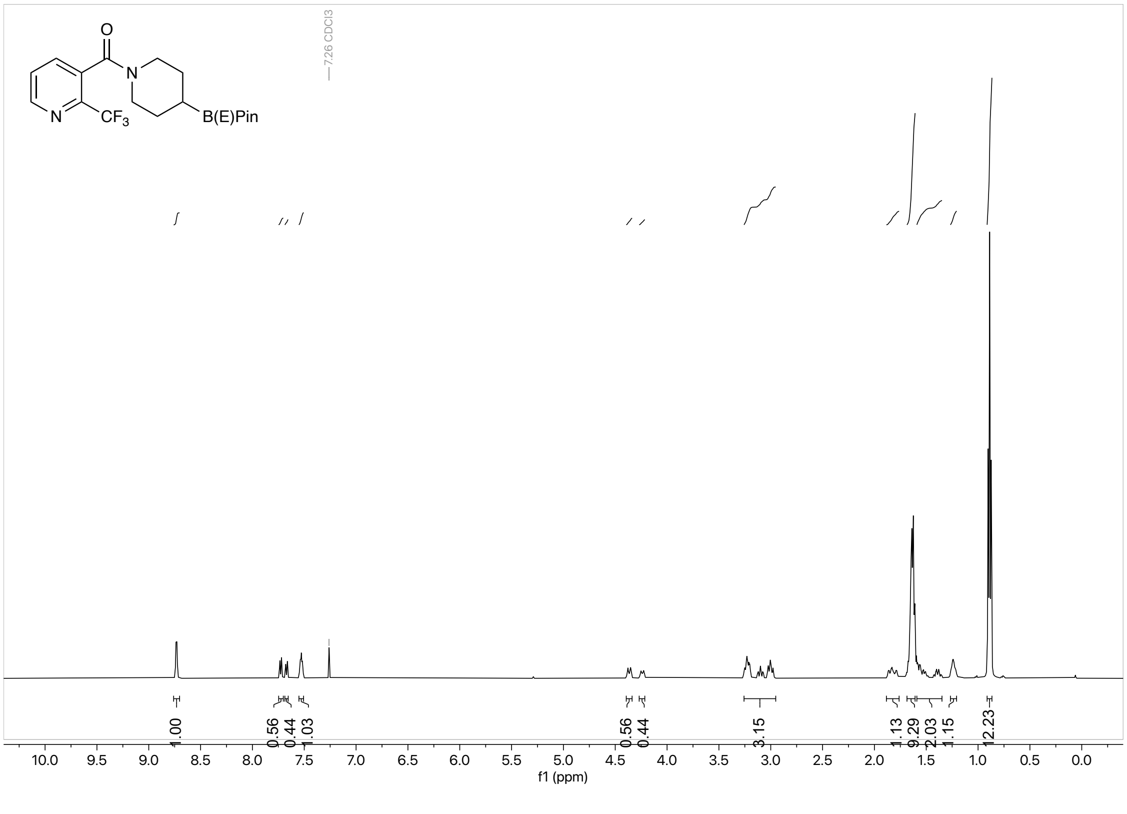

^{13}C NMR of **(3u)** (160 MHz, $CDCl_3$)

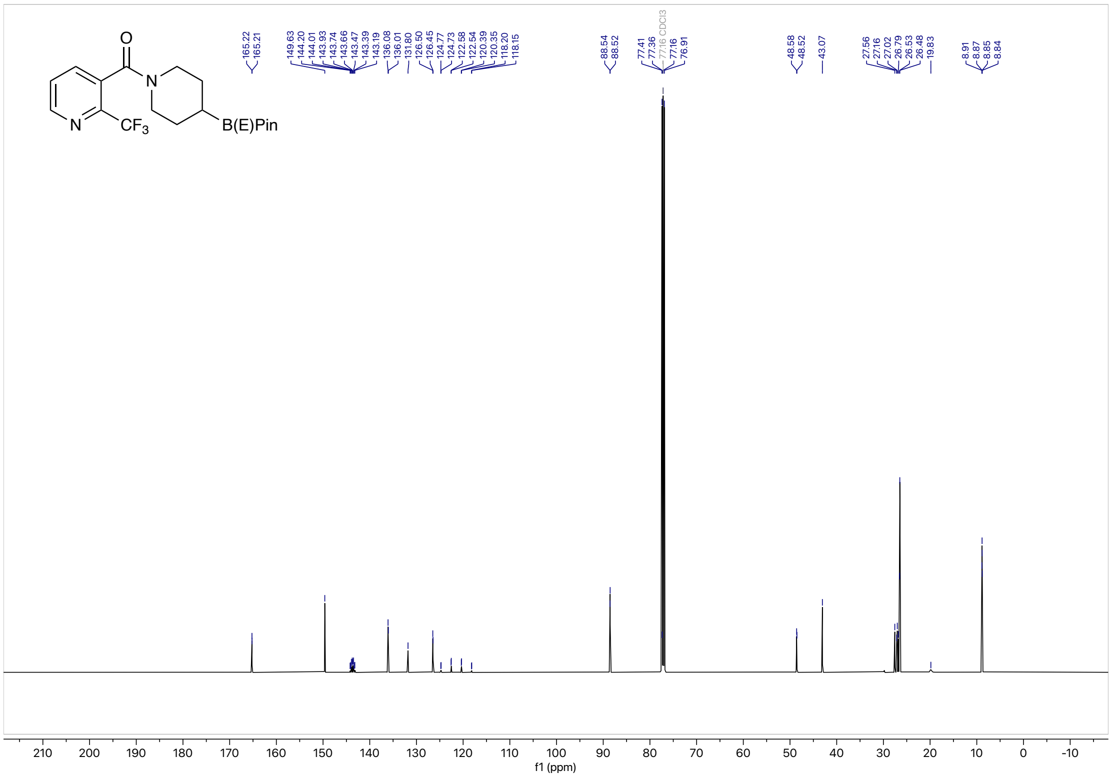

^{19}F NMR of **(3u)** (160 MHz, $CDCl_3$)

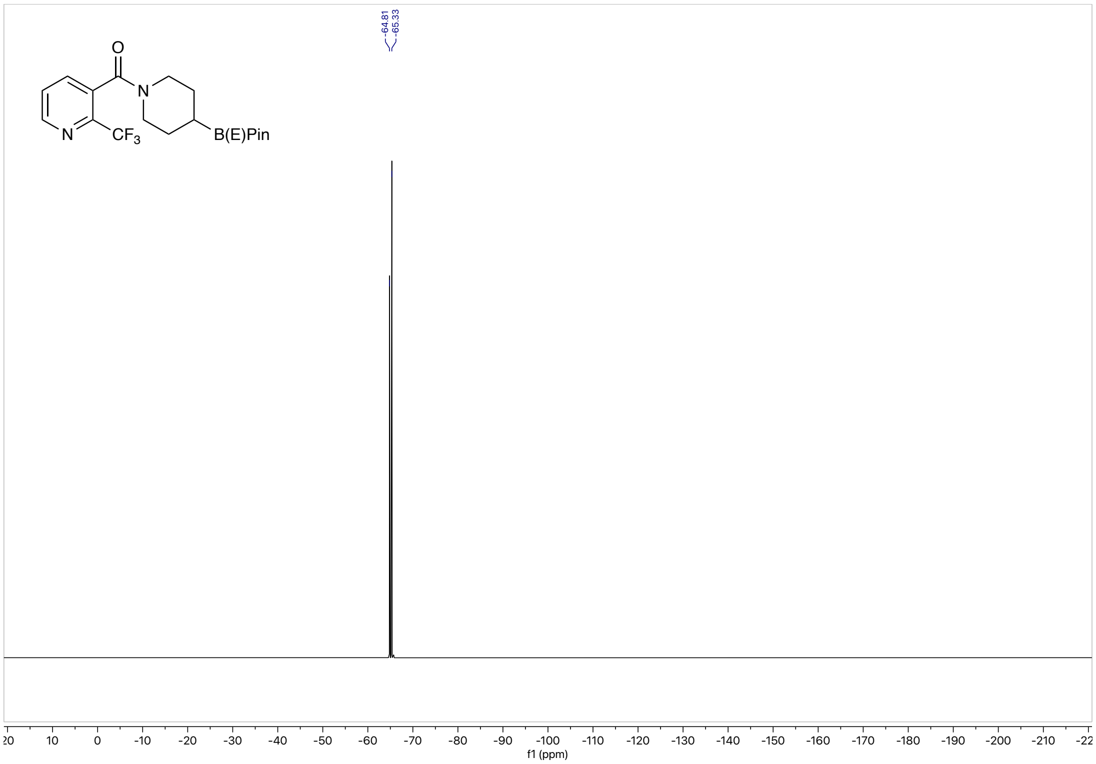

^{11}B NMR of **(3u)** (160 MHz, $CDCl_3$)

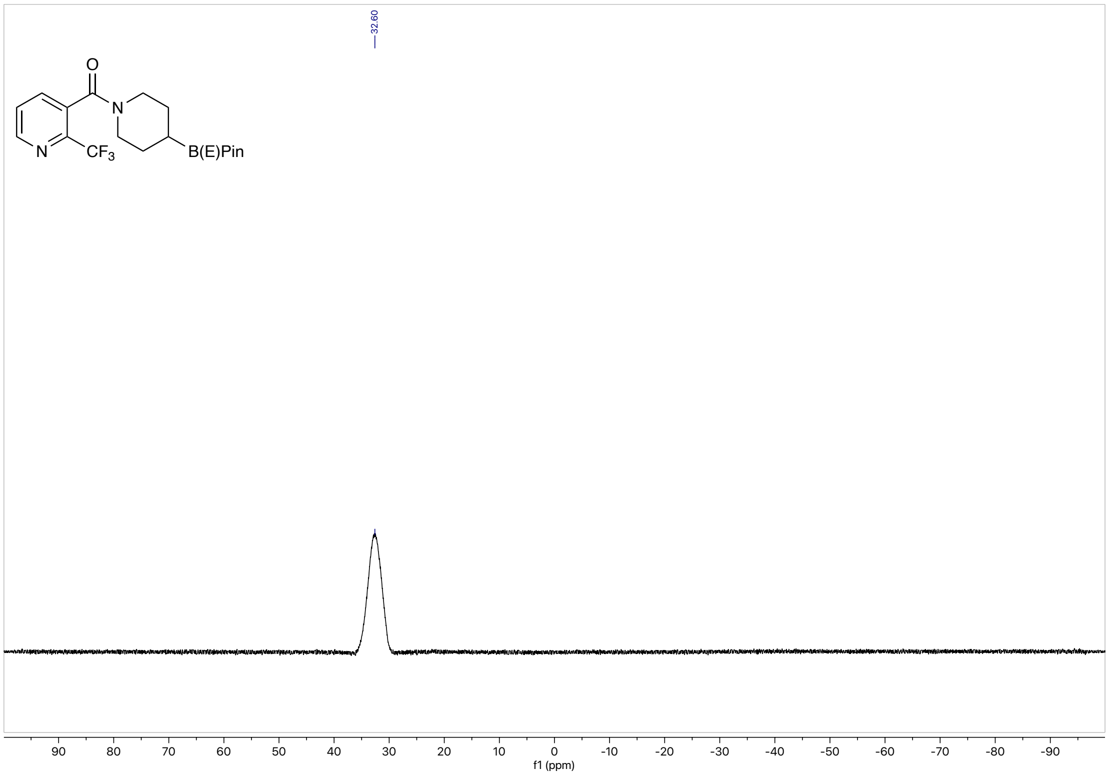

^{1}H NMR of 1,3-dimethyl-7-(2-(4,4,5,5-tetraethyl-1,3,2-dioxaborolan-2-yl)propyl)-3,7-dihydro-1H-purine-2,6-dione **(3v)** (500 MHz, $CDCl_3$)

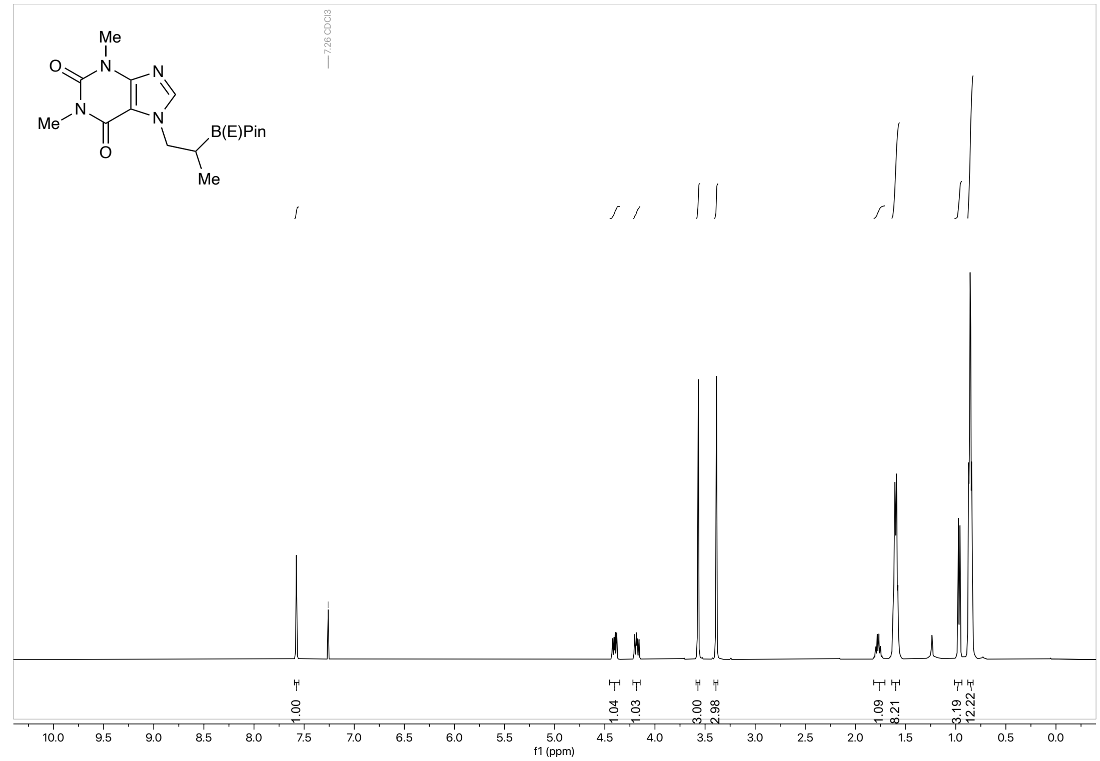

^{13}C NMR of 1,3-dimethyl-7-(2-(4,4,5,5-tetraethyl-1,3,2-dioxaborolan-2-yl)propyl)-3,7-dihydro-1H-purine-2,6-dione **(3v)** (126 MHz, $CDCl_3$)

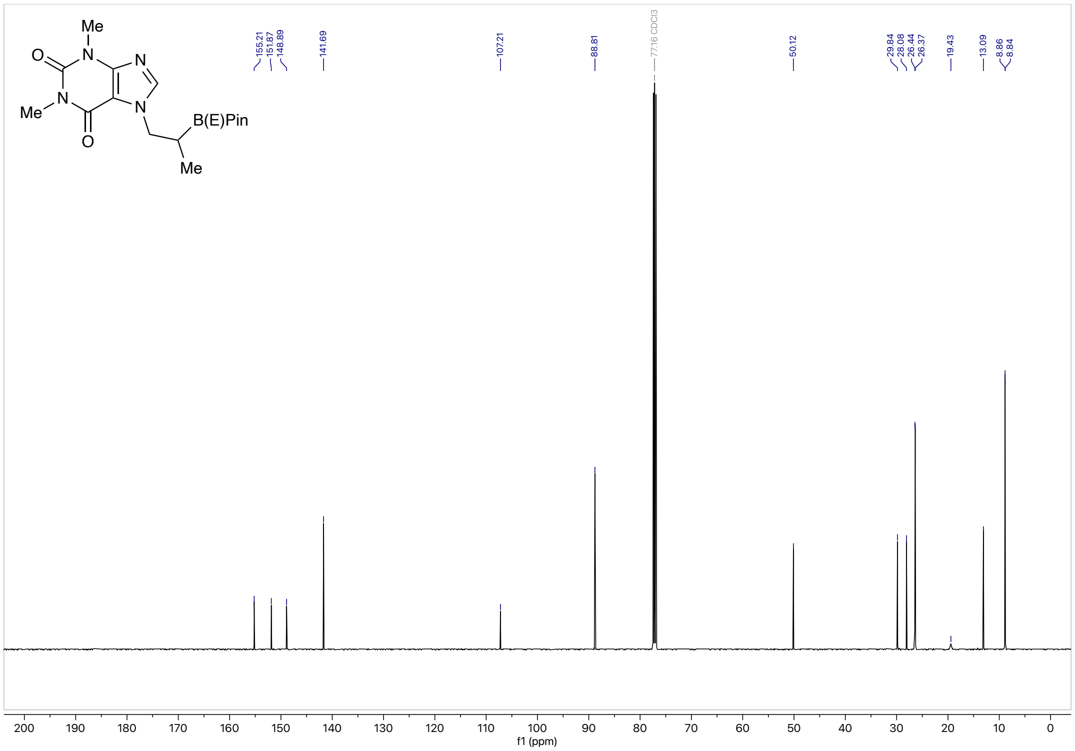

^{11}B NMR of **(3v)** (160 MHz, $CDCl_3$)

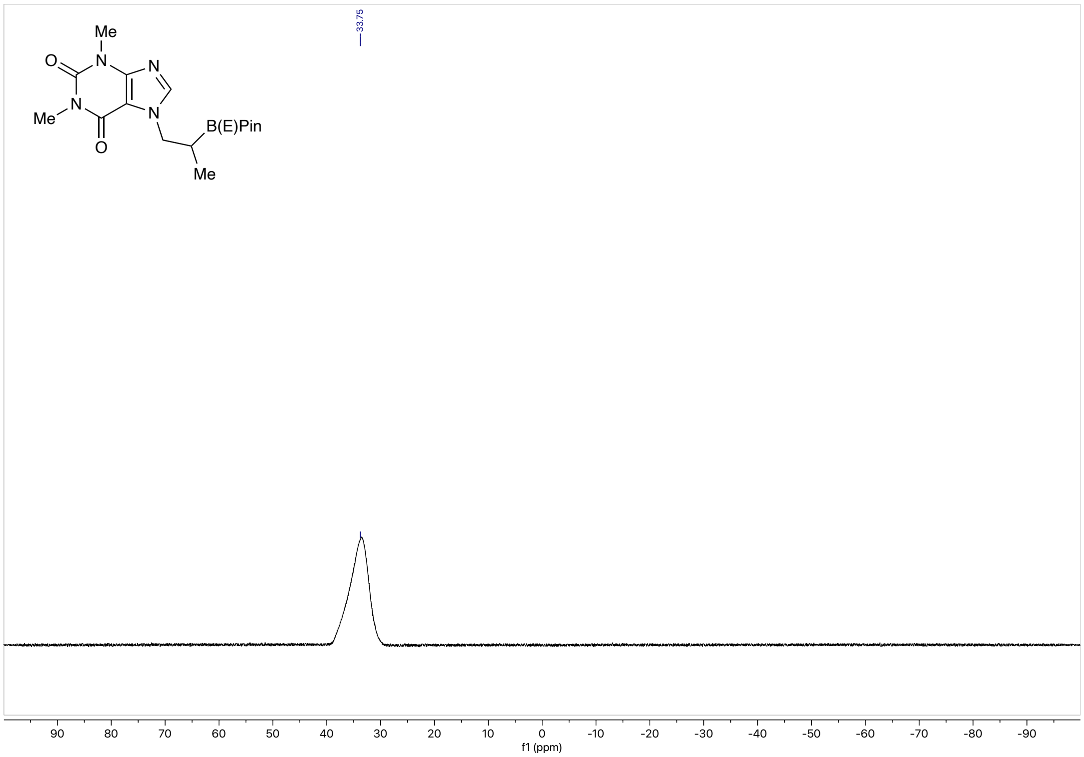

^{1}H NMR of 2-(adamantan-1-yl)-4,4,5,5-tetraethyl-1,3,2-dioxaborolane (**3w)** (500 MHz, $CDCl_3$)

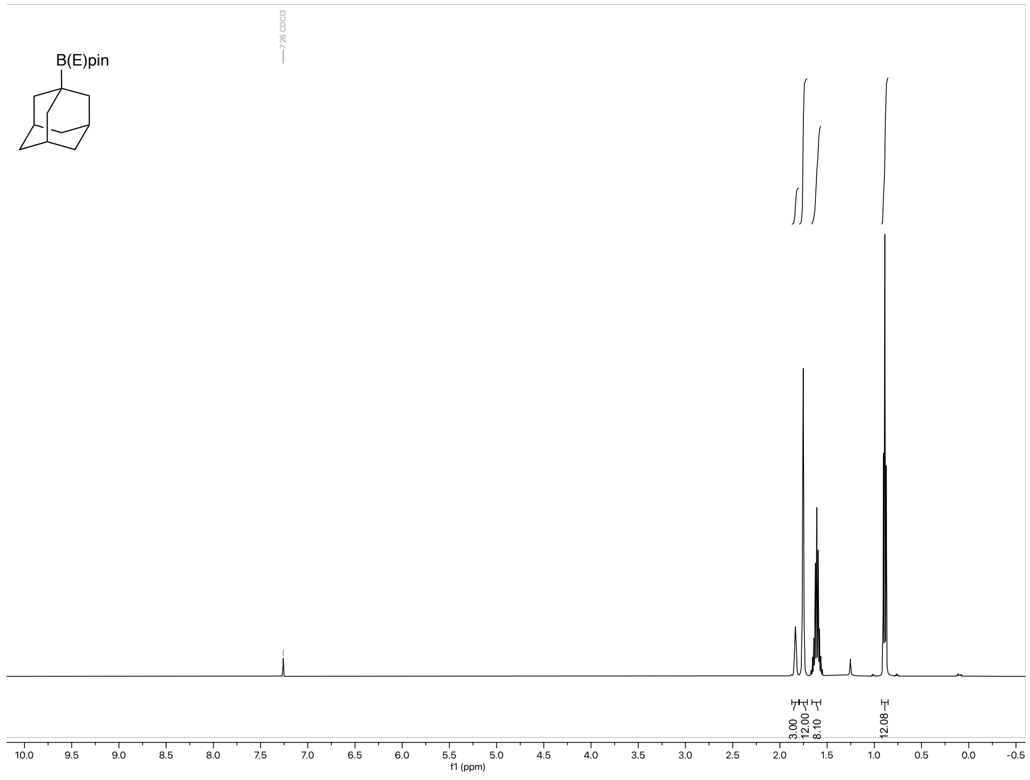

^{13}C NMR of 2-(adamantan-1-yl)-4,4,5,5-tetraethyl-1,3,2-dioxaborolane (**3w**) (126 MHz, $CDCl_3$)

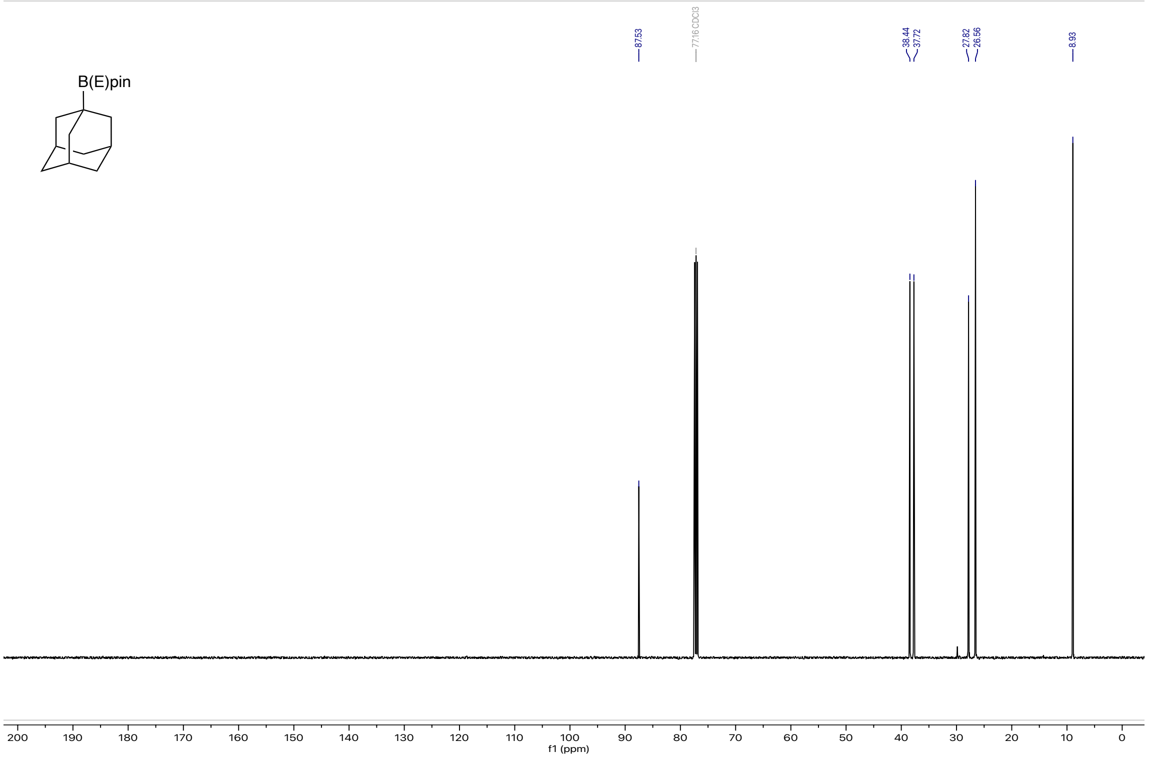

^{11}B NMR of (**3w**) (160 MHz, $CDCl_3$)

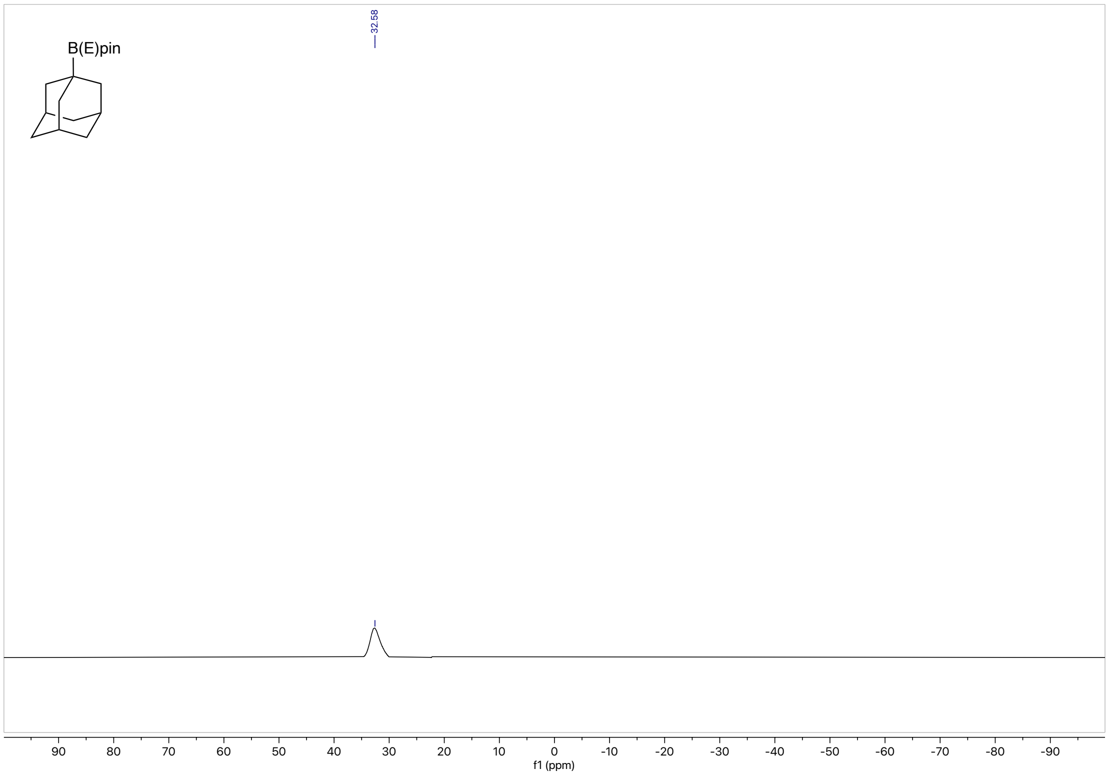

^{1}H NMR of 4,4,5,5-tetraethyl-2-(4-methyltetrahydro-2*H*-pyran-4-yl)-1,3,2-dioxaborolane (**3x**) (500 MHz, $CDCl_3$)

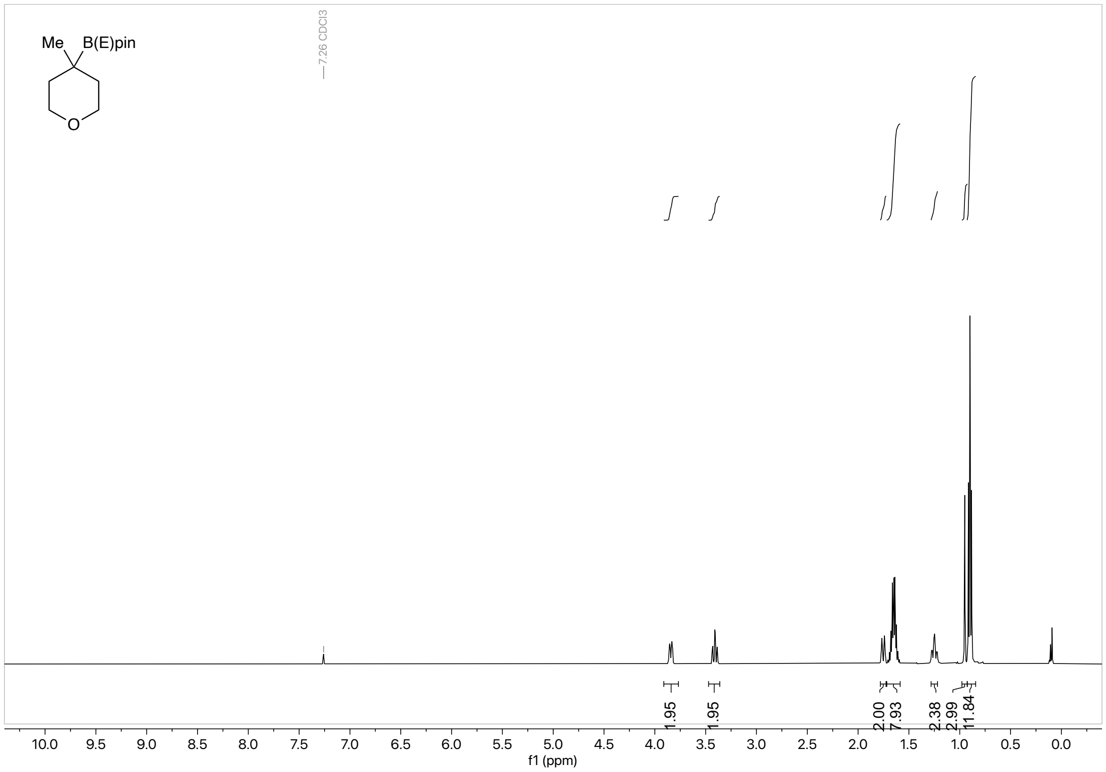

^{13}C NMR of 4,4,5,5-tetraethyl-2-(4-methyltetrahydro-2*H*-pyran-4-yl)-1,3,2-dioxaborolane (**3x**) (126 MHz, $CDCl_3$)

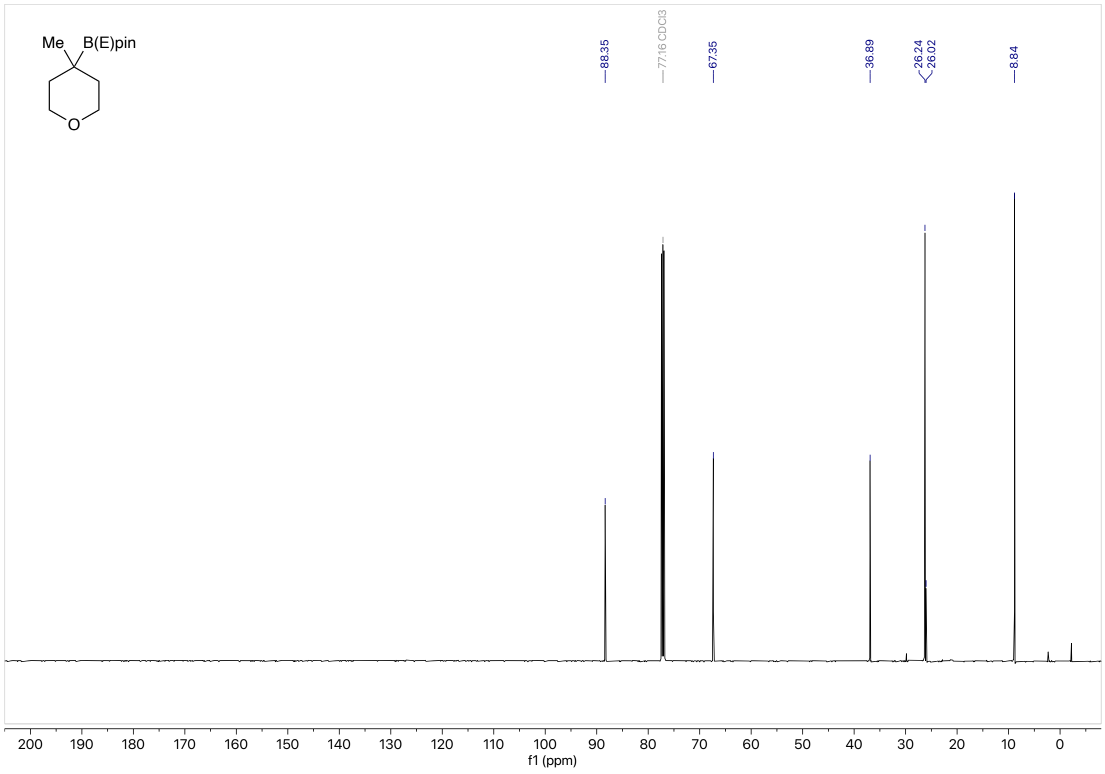

^{11}B NMR of (**3x**) (160 MHz, $CDCl_3$)

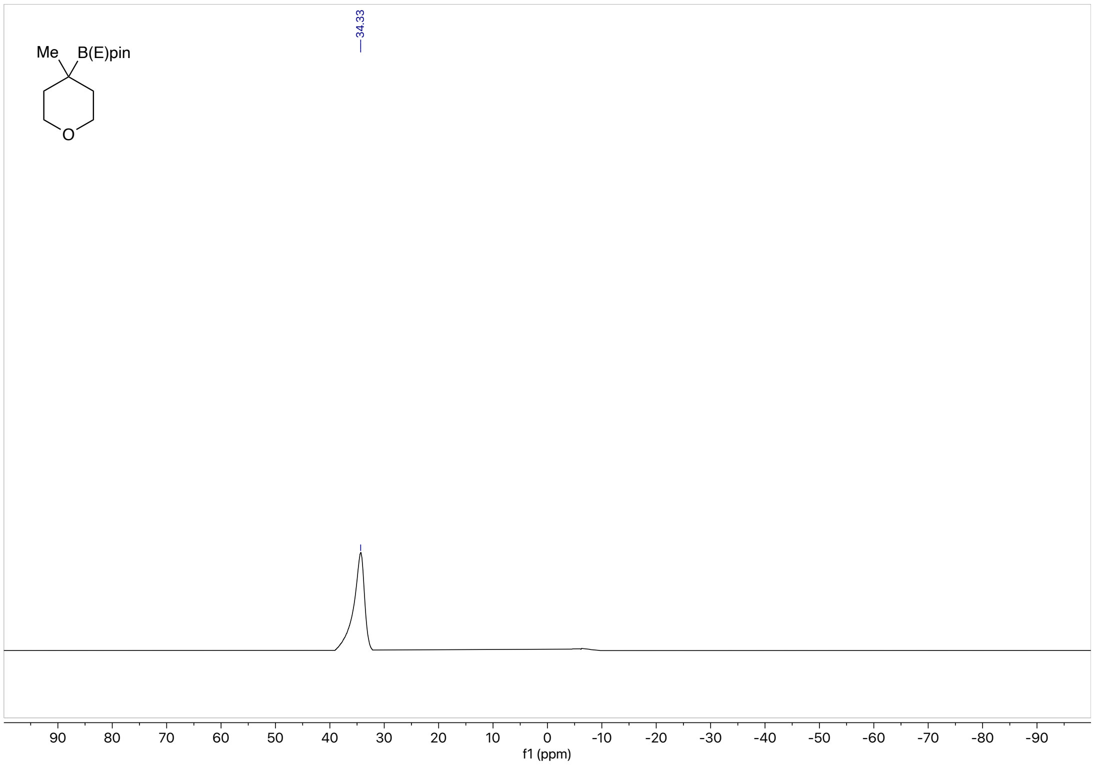

^{1}H NMR of 4,4,5,5-tetraethyl-2-(4-methyltetrahydro-2*H*-pyran-4-yl)-1,3,2-dioxaborolane (**3y**) (500 MHz, $CDCl_3$)

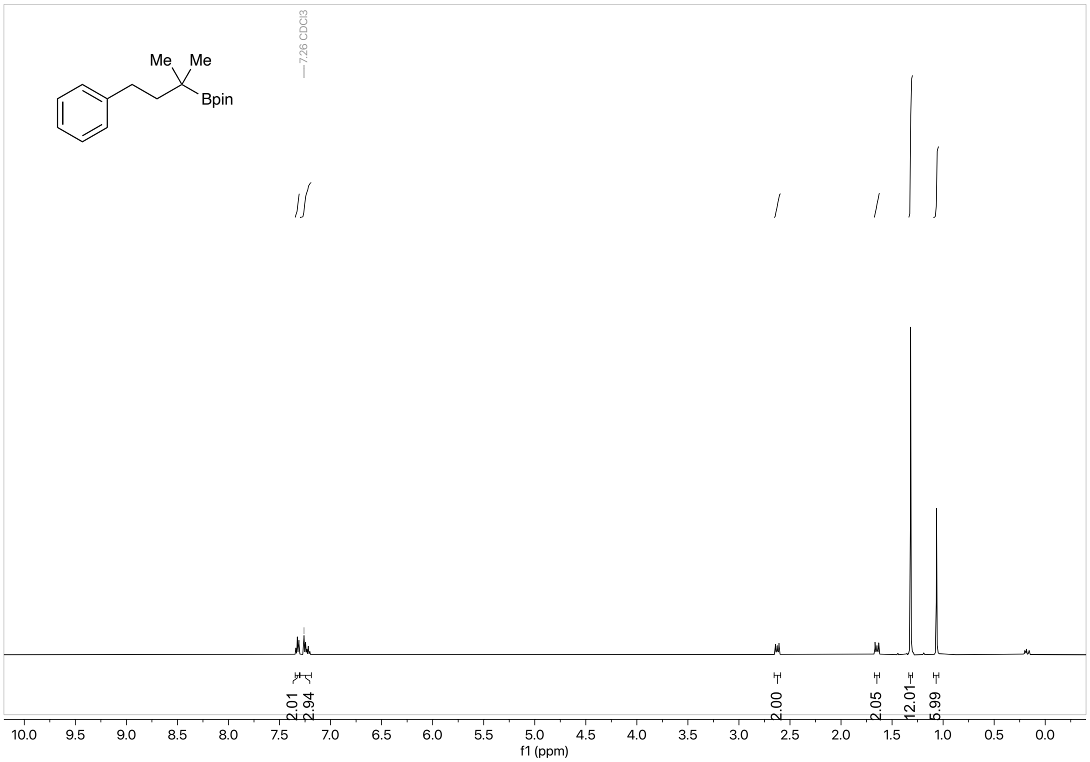

^{13}C NMR of 4,4,5,5-tetraethyl-2-(4-methyltetrahydro-2*H*-pyran-4-yl)-1,3,2-dioxaborolane (**3y**) (500 MHz, $CDCl_3$)

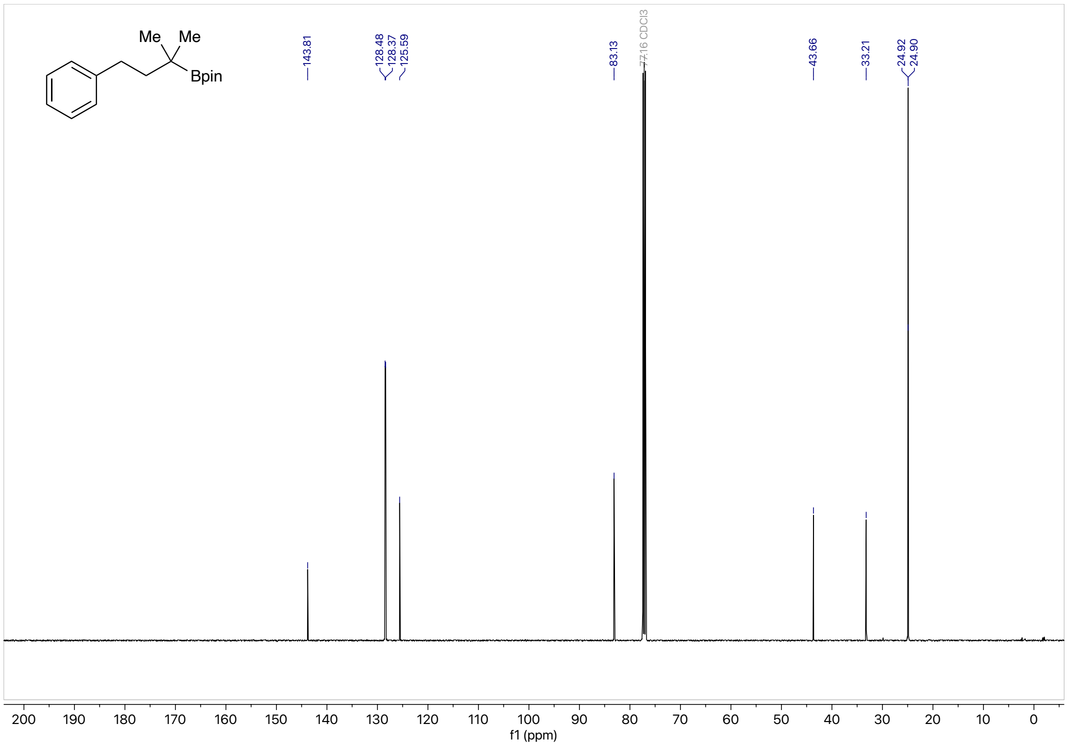

^{1}H NMR of 4-methyl-4-(4,4,5,5-tetraethyl-1,3,2-dioxaborolan-2-yl)pentyl 4-(trifluoromethyl)benzoate (**3z**) (500 MHz, $CDCl_3$)

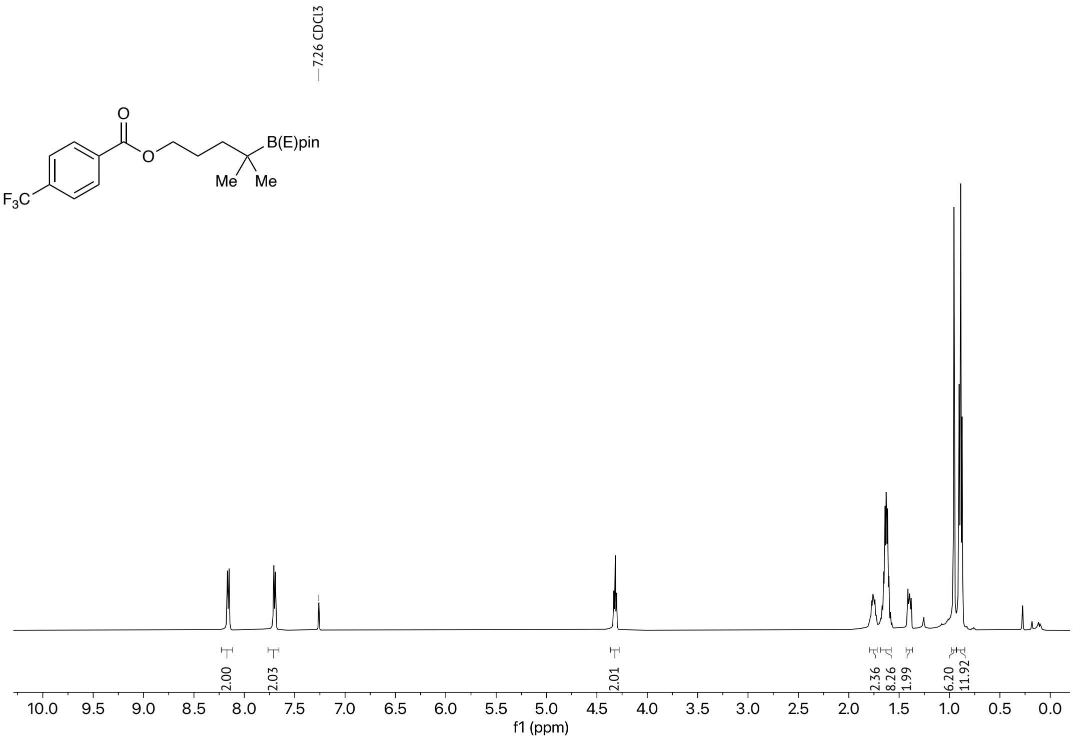

^{13}C NMR of (**3z**) (126 MHz, $CDCl_3$)

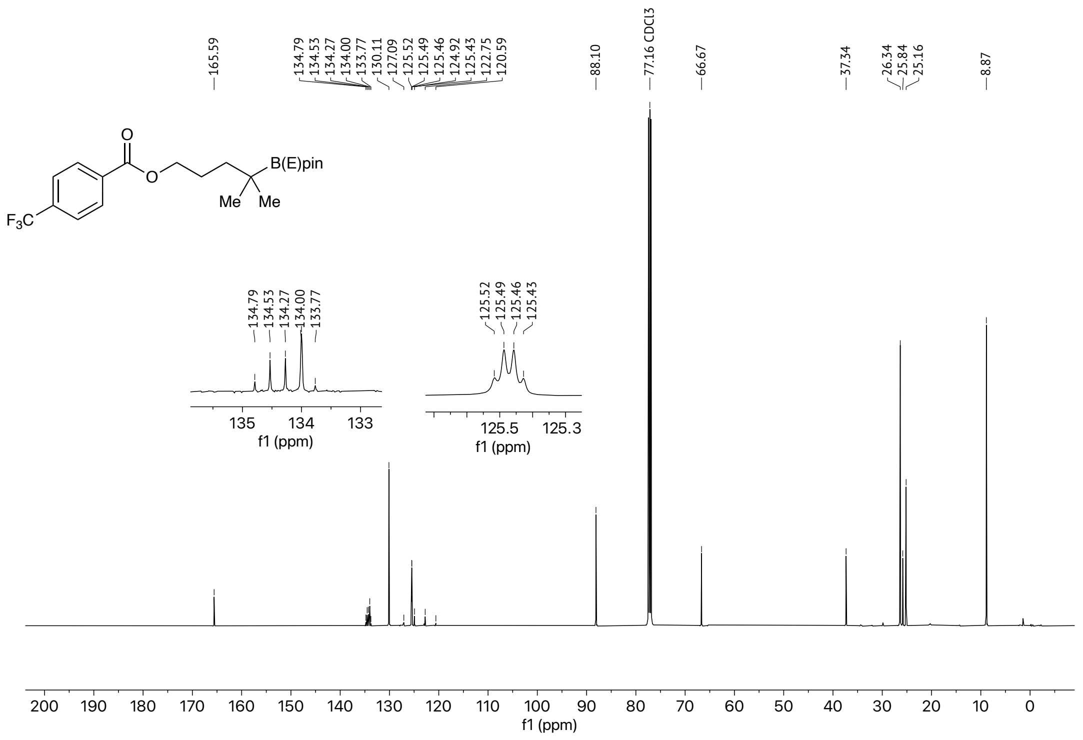

^{11}B NMR of (**3z**) (160 MHz, $CDCl_3$)

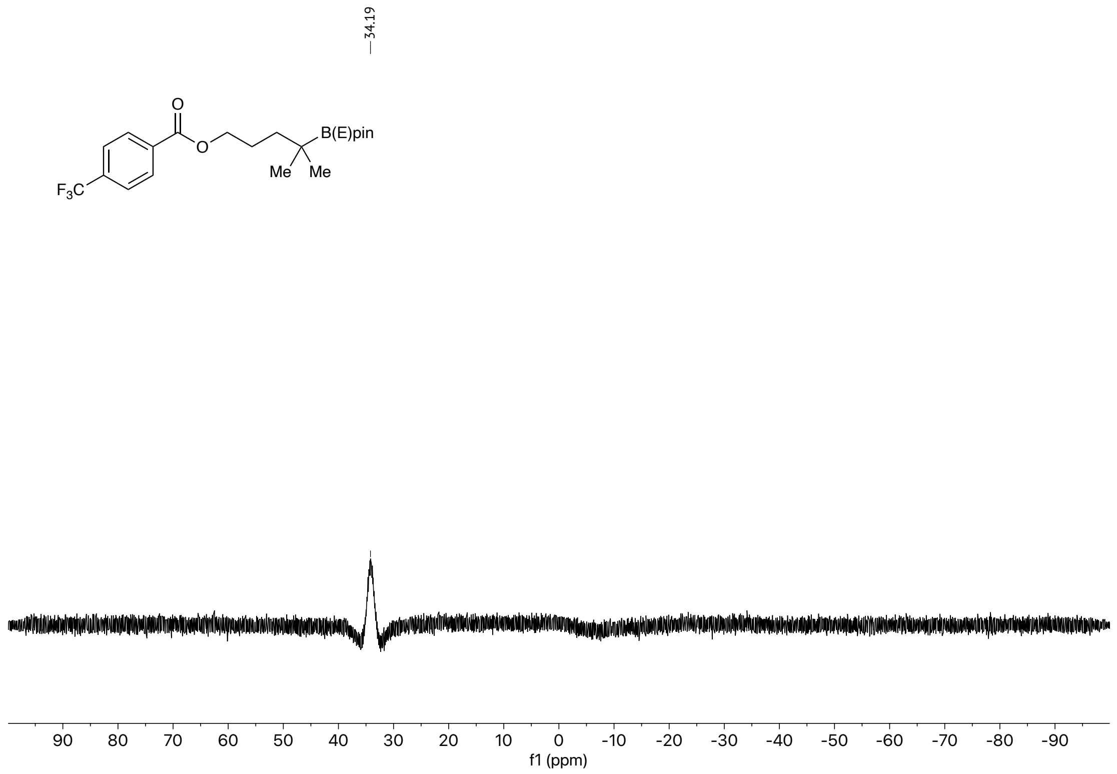

^{19}F NMR of **(3z)** (470 MHz, $CDCl_3$)

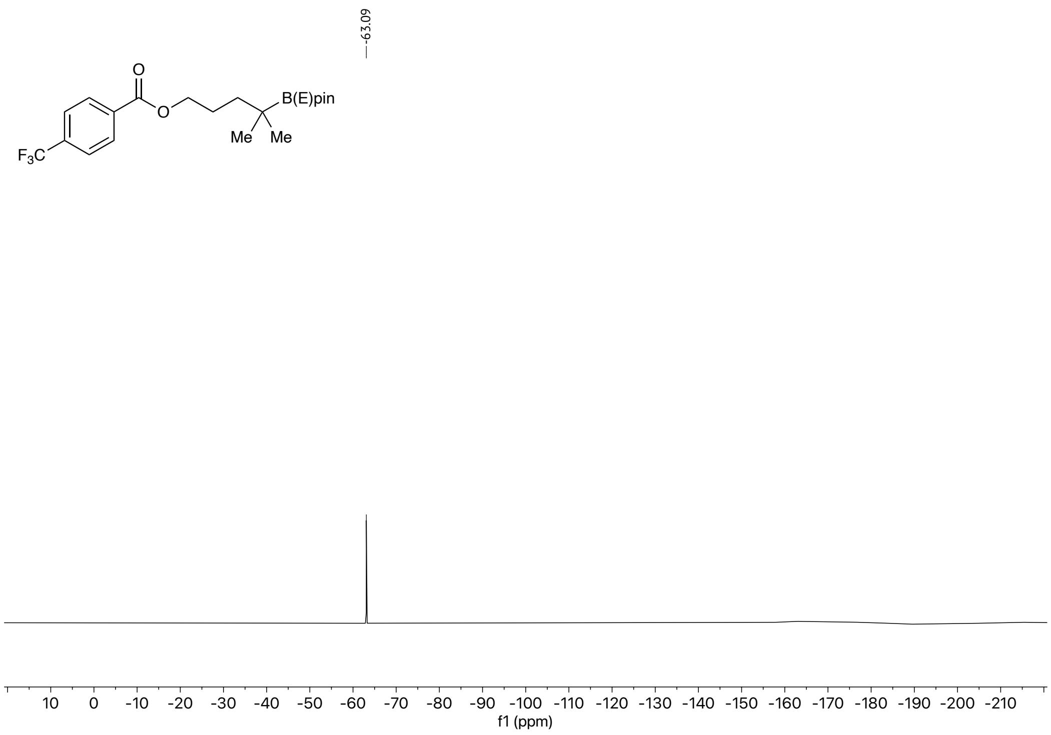

^1H NMR of 2-methoxy-4-(3-methyl-3-(4,4,5,5-tetraethyl-1,3,2-dioxaborolan-2-yl)butyl)phenol **(3aa)** (500 MHz, $CDCl_3$)

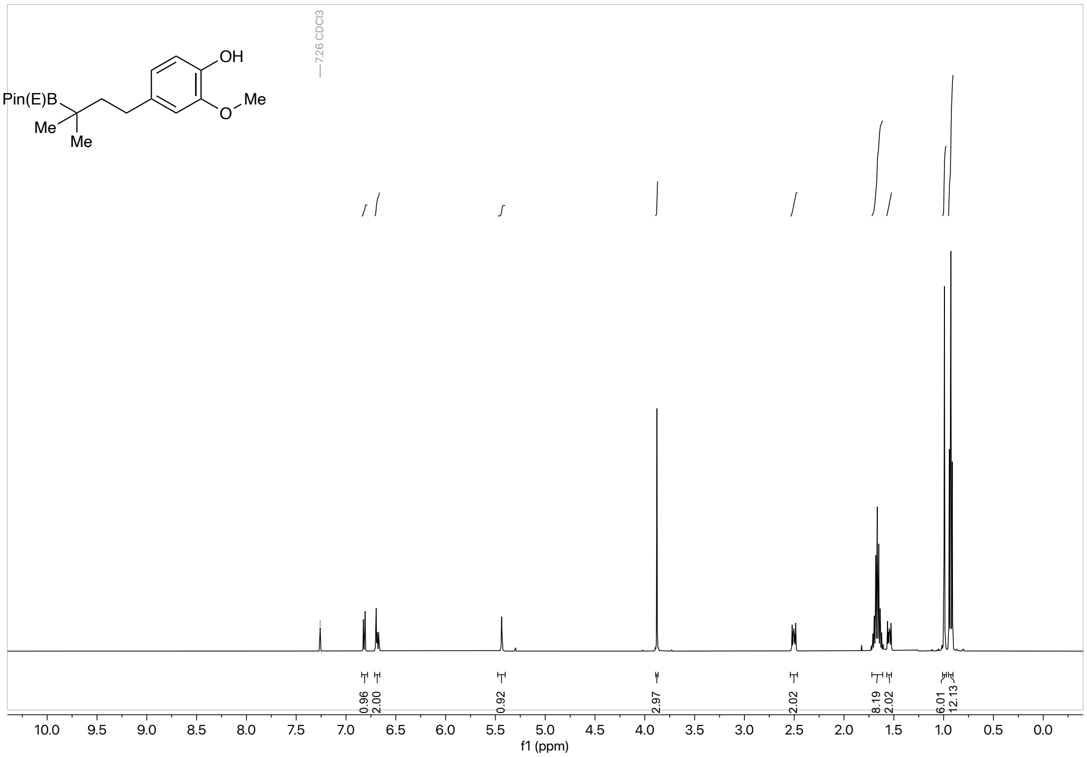

^{13}C NMR of **(3aa)** (126 MHz, $CDCl_3$)

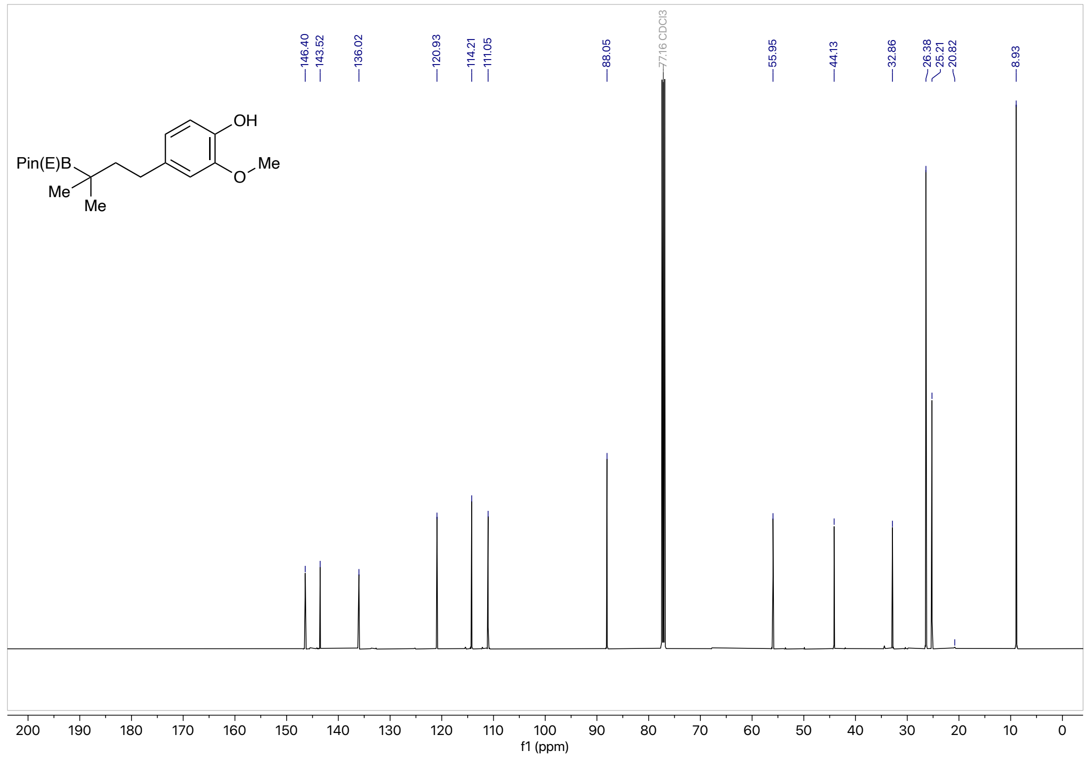

^{11}B NMR of **(3aa)** (160 MHz, $CDCl_3$)

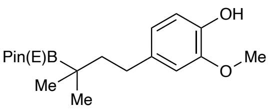

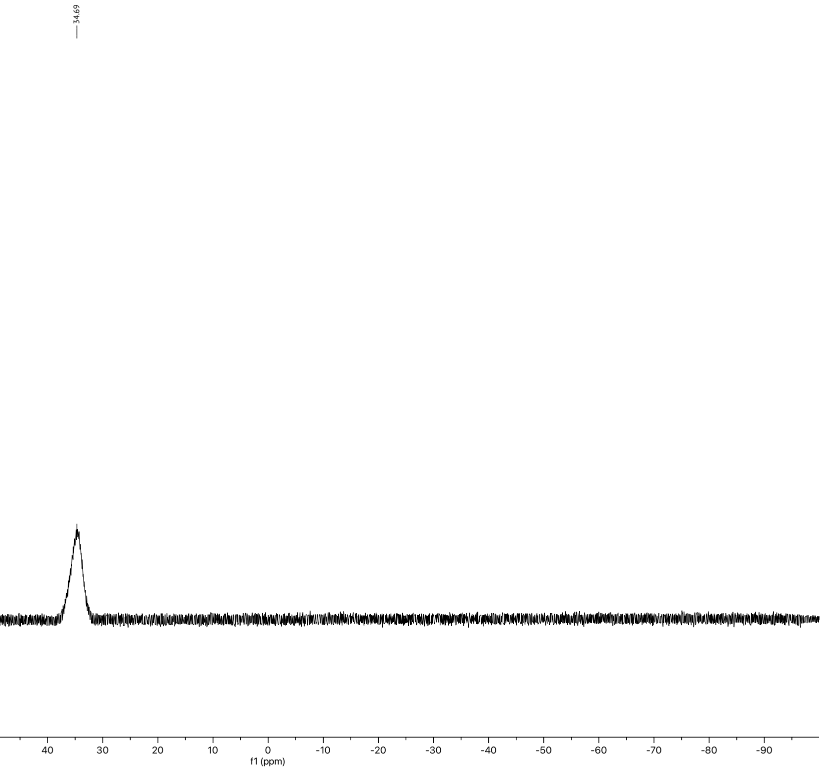

^{1}H NMR of 4-methyl-4-(4,4,5,5-tetraethyl-1,3,2-dioxaborolan-2-yl)pentyl (S)-2-(6-methoxynaphthalen-2-yl)propanoate **(3ab)** (500 MHz, $CDCl_3$)

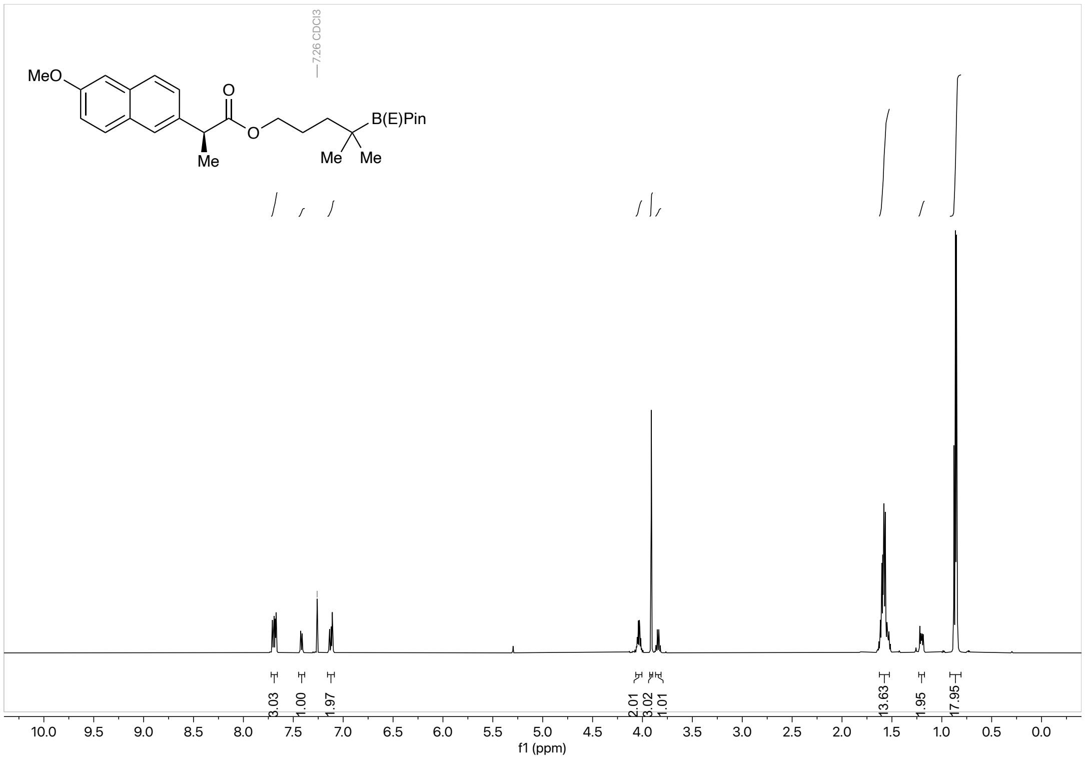

^{13}C NMR of **(3ab)** (126 MHz, $CDCl_3$)

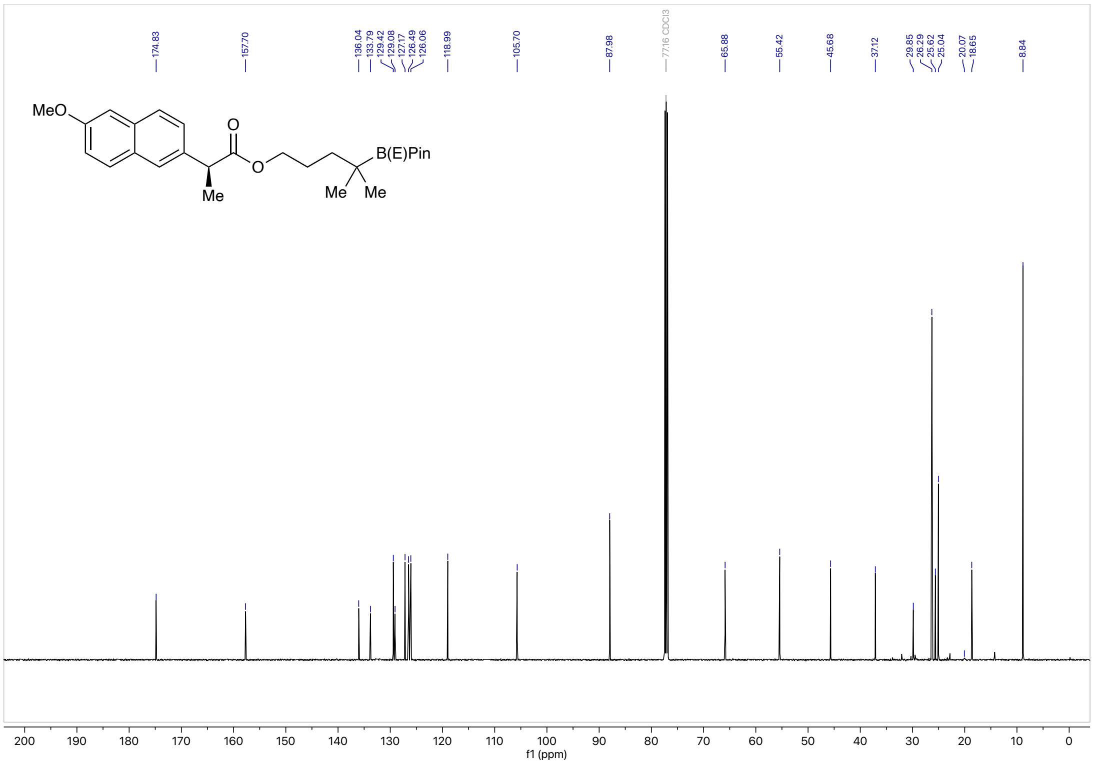

^{11}B NMR of **(3ab)** (160 MHz, $CDCl_3$)

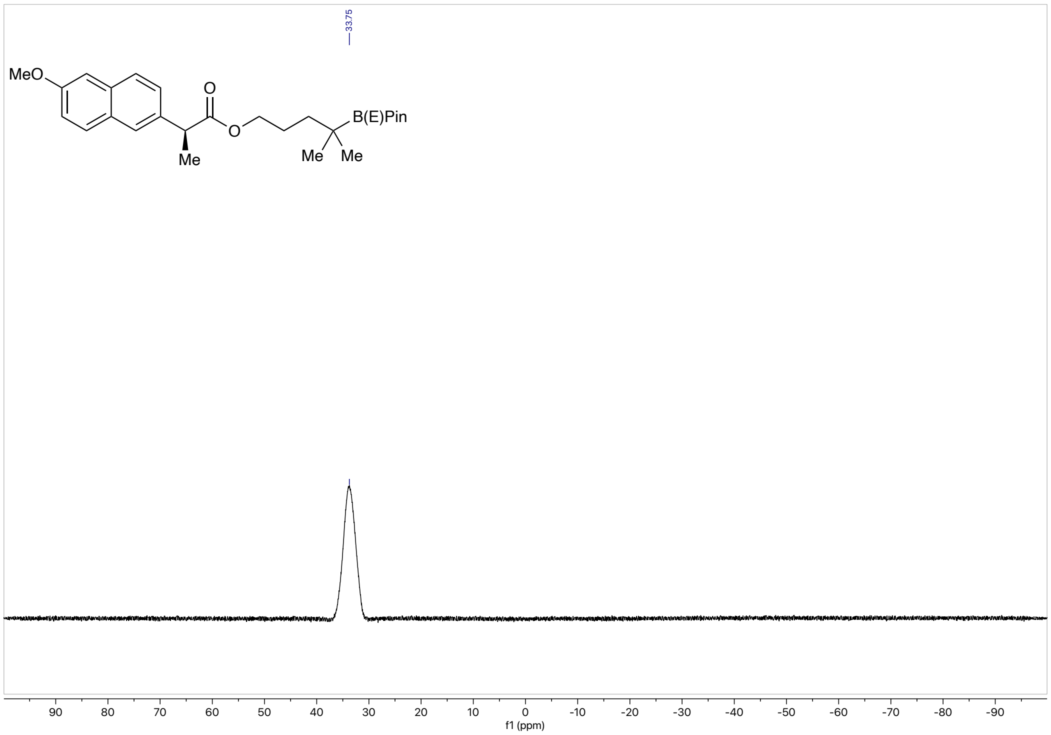

^{1}H NMR of methyl 2',4'-difluoro-4-(3-methyl-3-(4,4,5,5-tetraethyl-1,3,2-dioxaborolan-2-yl)butoxy)-[1,1'-biphenyl]-3-carboxylate **(3ac)** (500 MHz, $CDCl_3$)

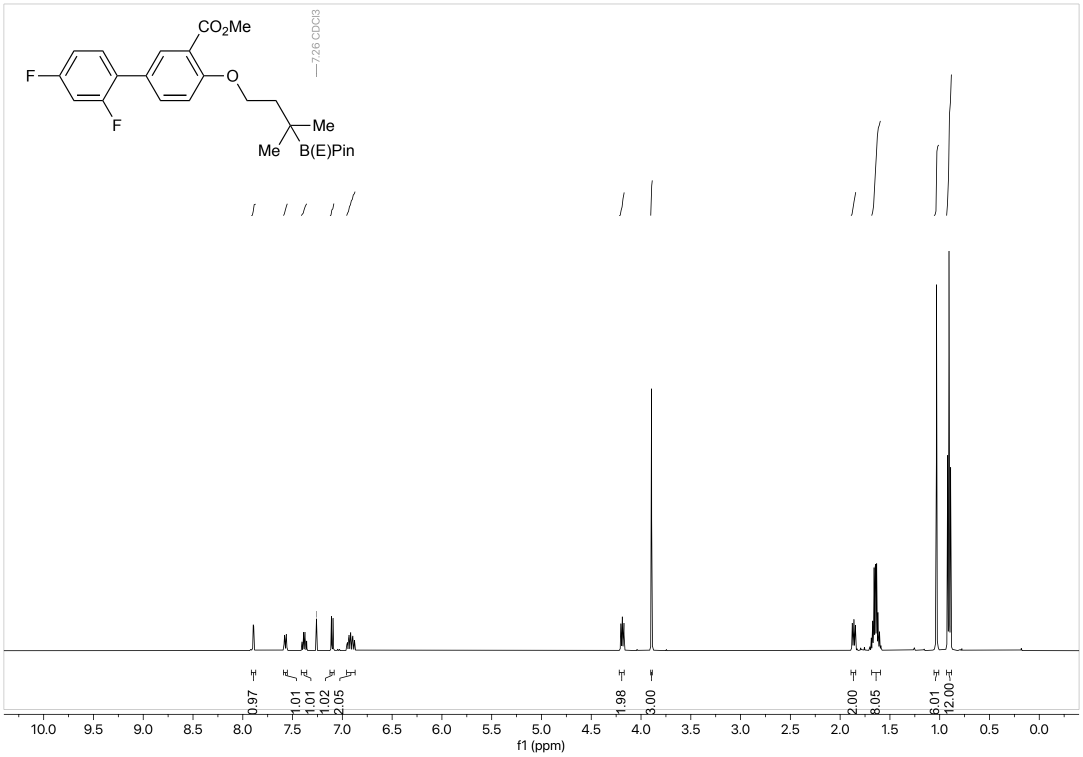

^{13}C NMR of **(3ac)** (126 MHz, $CDCl_3$)

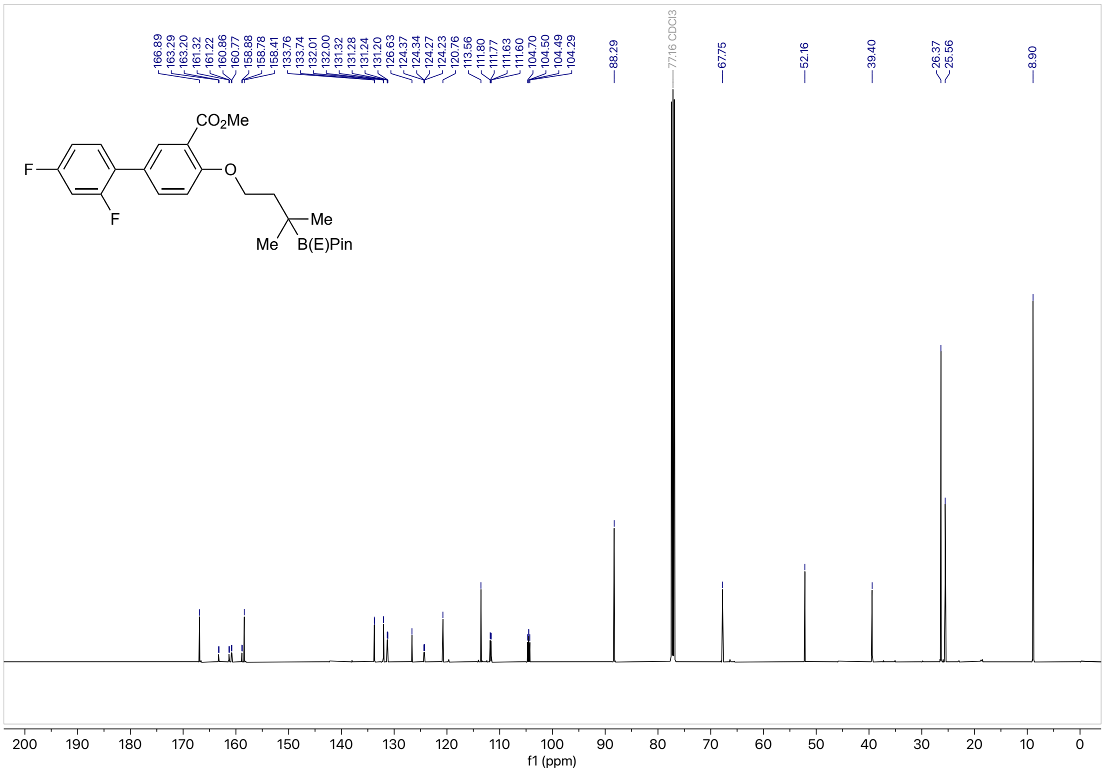

^{19}F NMR of **(3ac)** (470 MHz, $CDCl_3$)

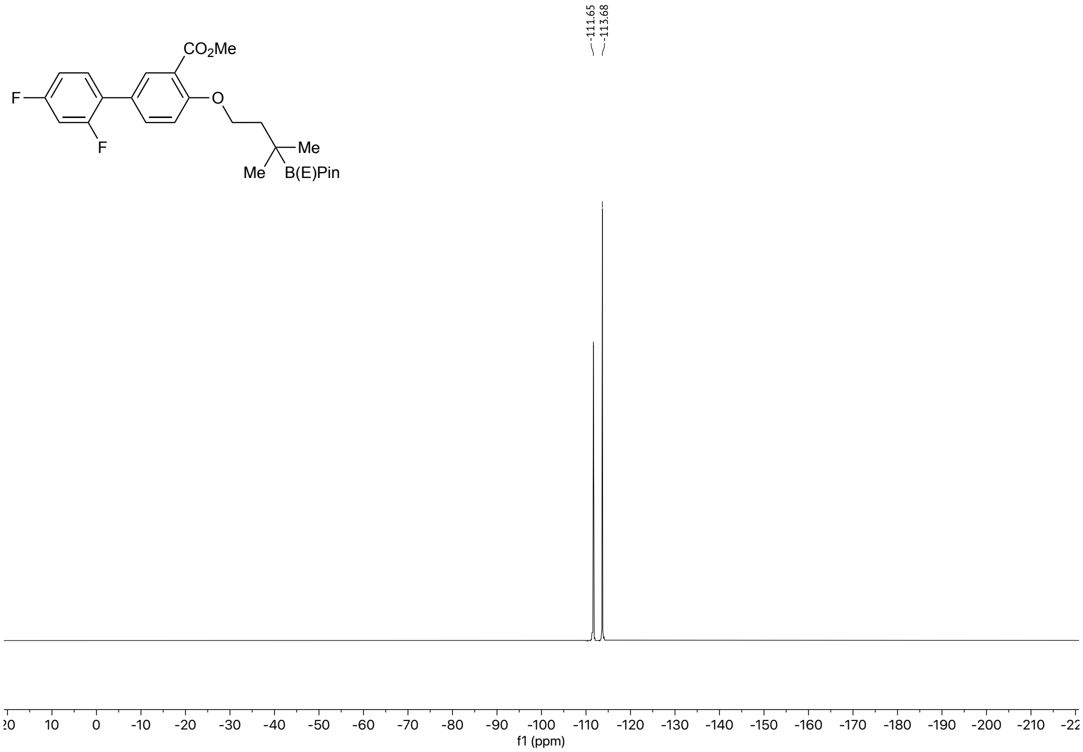

^{11}B NMR of **(3ac)** (160 MHz, $CDCl_3$)

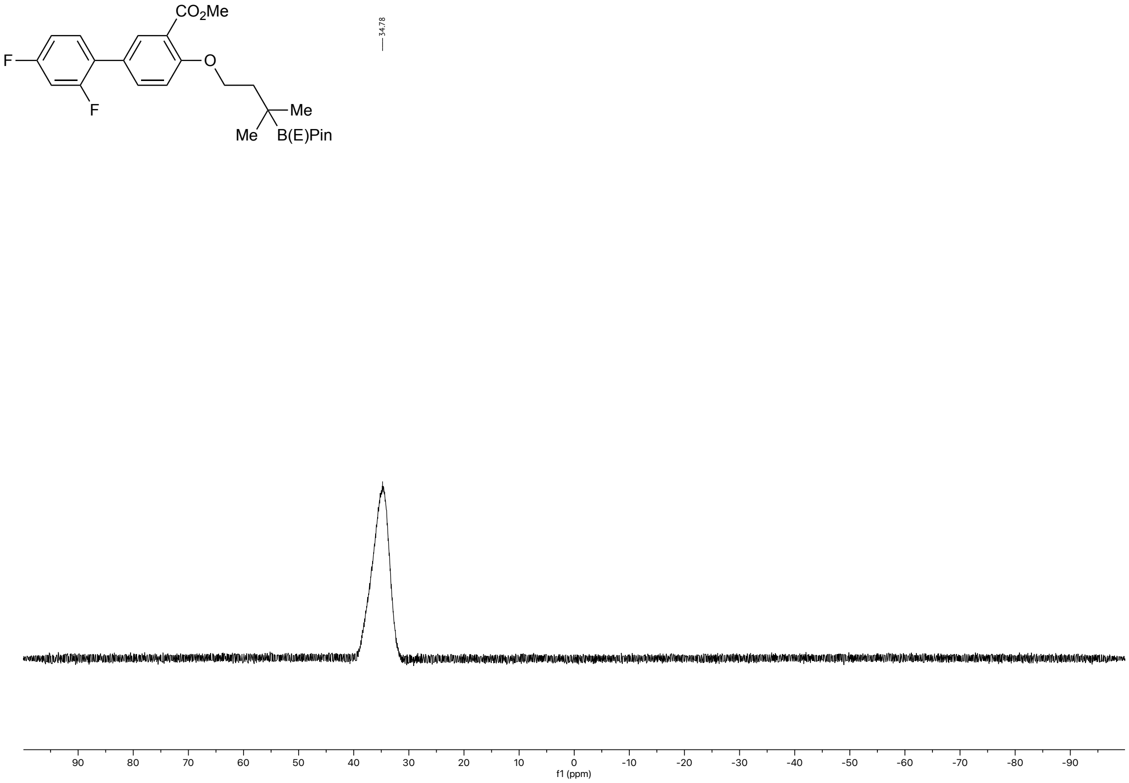

^{1}H NMR of azepan-4-amine 2,2,2-trifluoroacetate **(4)** (500 MHz, D_2O)

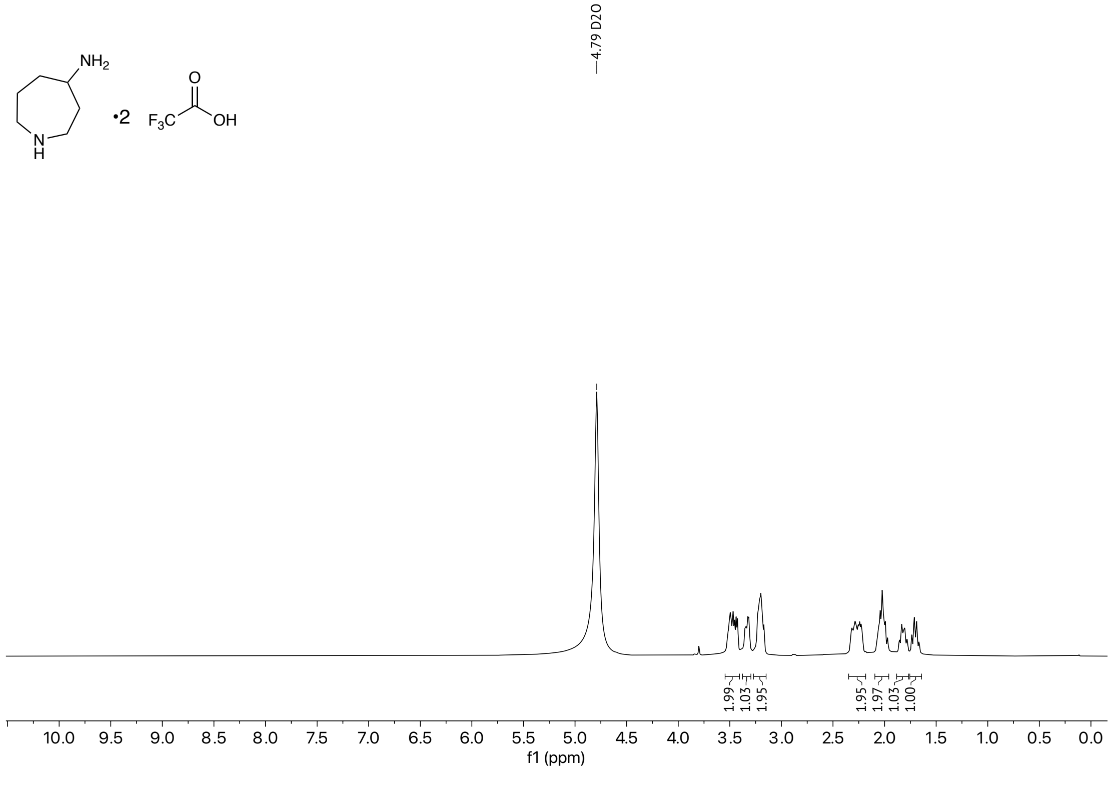

^{13}C NMR of **(4)** (126 MHz, D_2O)

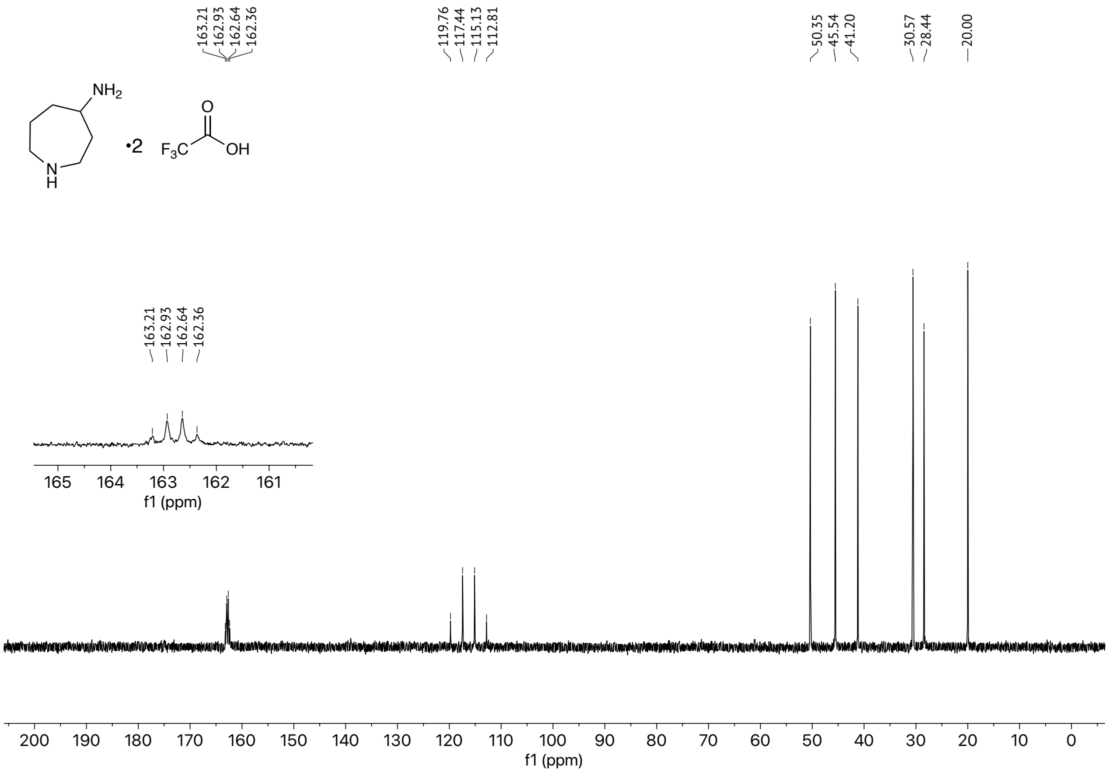

^{19}F NMR of **(4)** (470 MHz, D_2O)

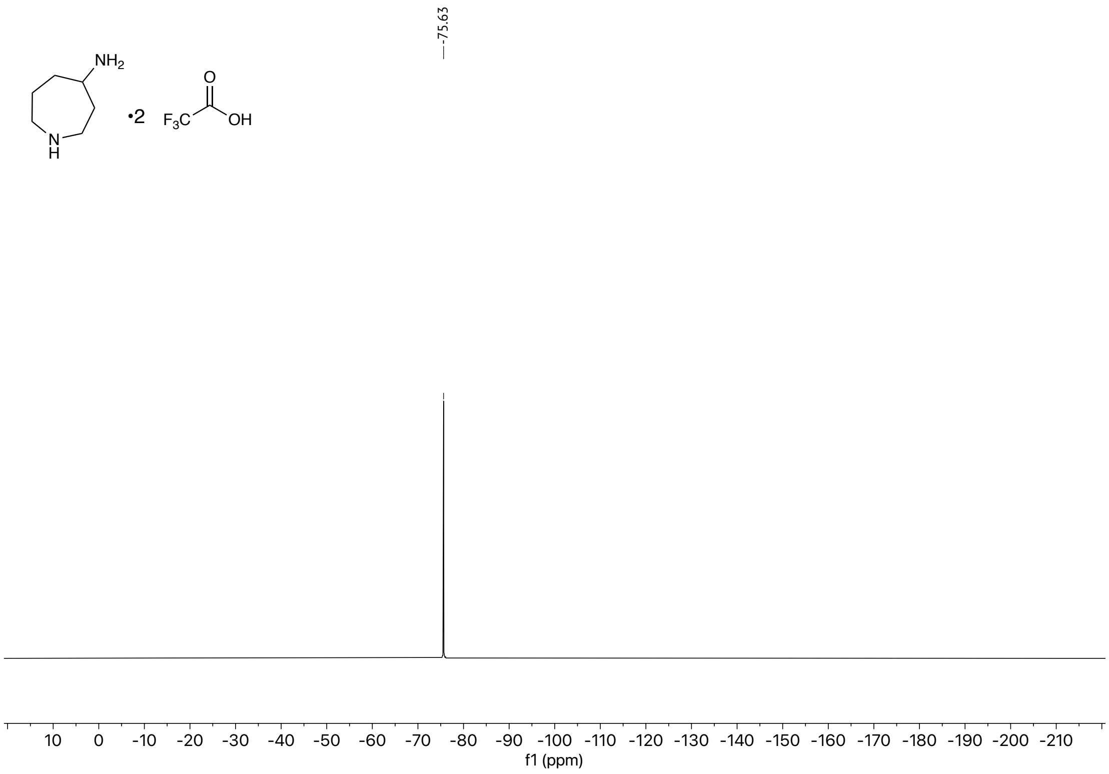

^{1}H NMR of *tert*-butyl 4-(hydroxymethyl)azepane-1-carboxylate **(5)** (500 MHz, $CDCl_3$)

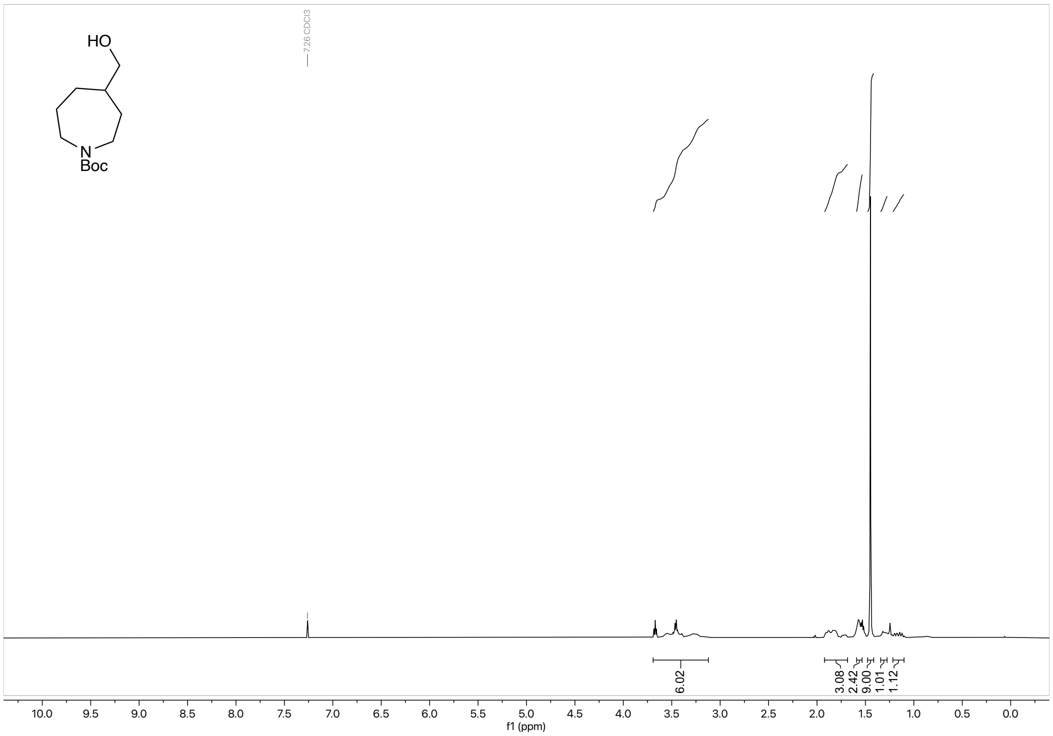

^{13}C NMR of *tert*-butyl 4-(hydroxymethyl)azepane-1-carboxylate **(5)** (126 MHz, $CDCl_3$)

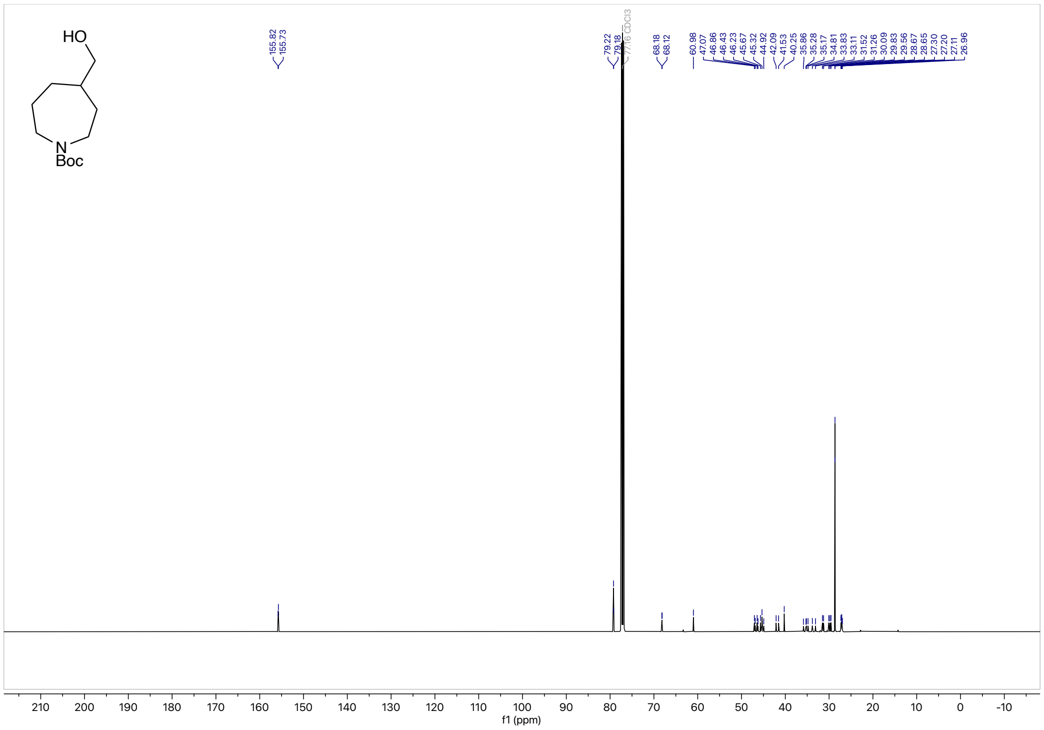

^{1}H NMR of *tert*-butyl 4-vinylazepane-1-carboxylate **(6)** (500 MHz, $CDCl_3$)

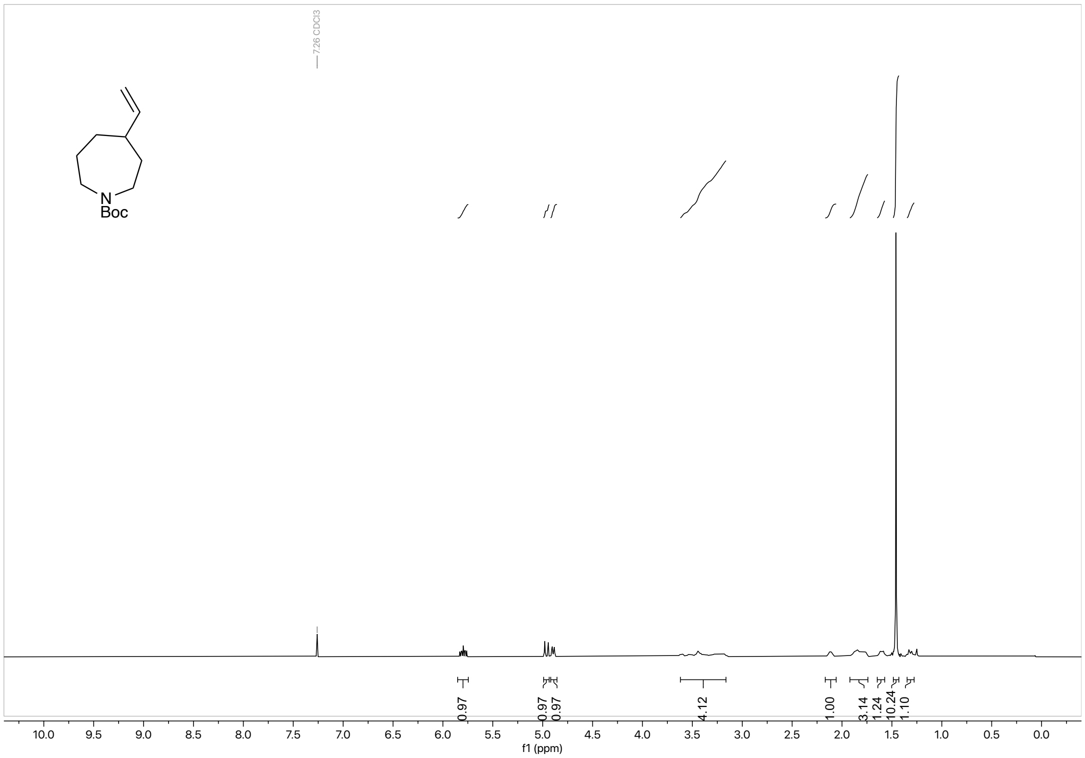

^{13}C NMR of *tert*-butyl 4-vinylazepane-1-carboxylate **(6)** (126 MHz, $CDCl_3$)

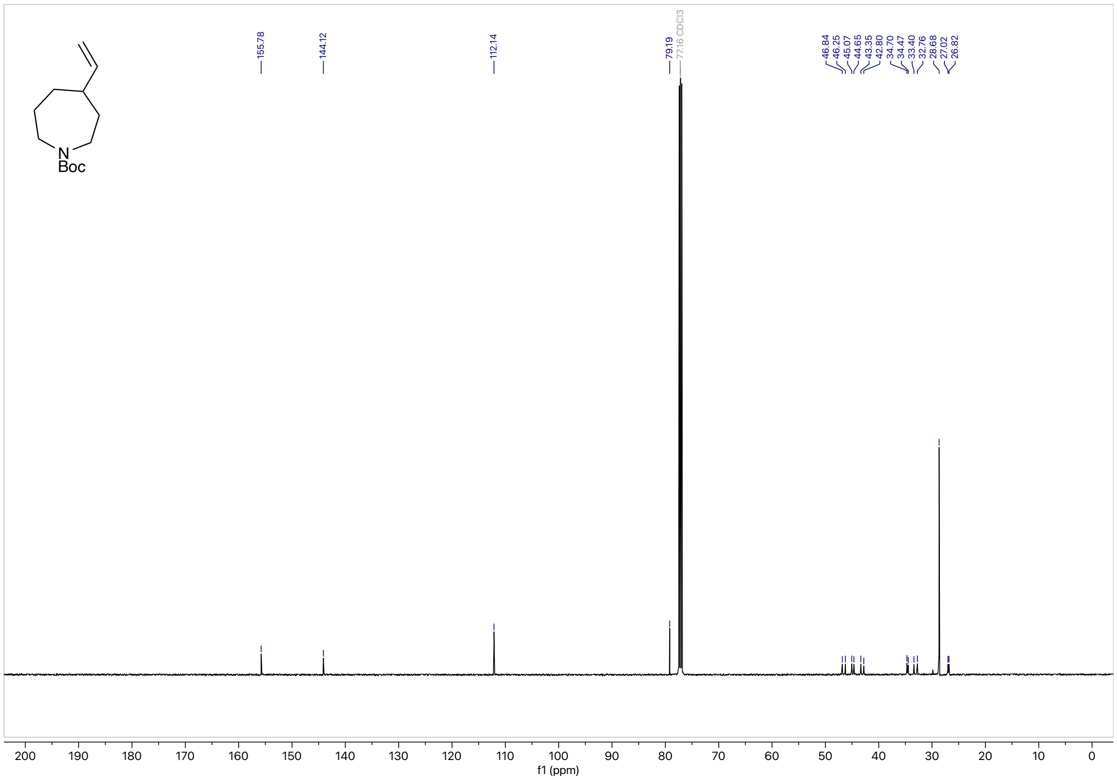

^{1}H NMR of 5-methyl-2-((1-tosylpyrrolidin-3-yl)methyl)-1,5,2-oxazaborepane-3,7-dione **(7)** (500 MHz, DMSO)

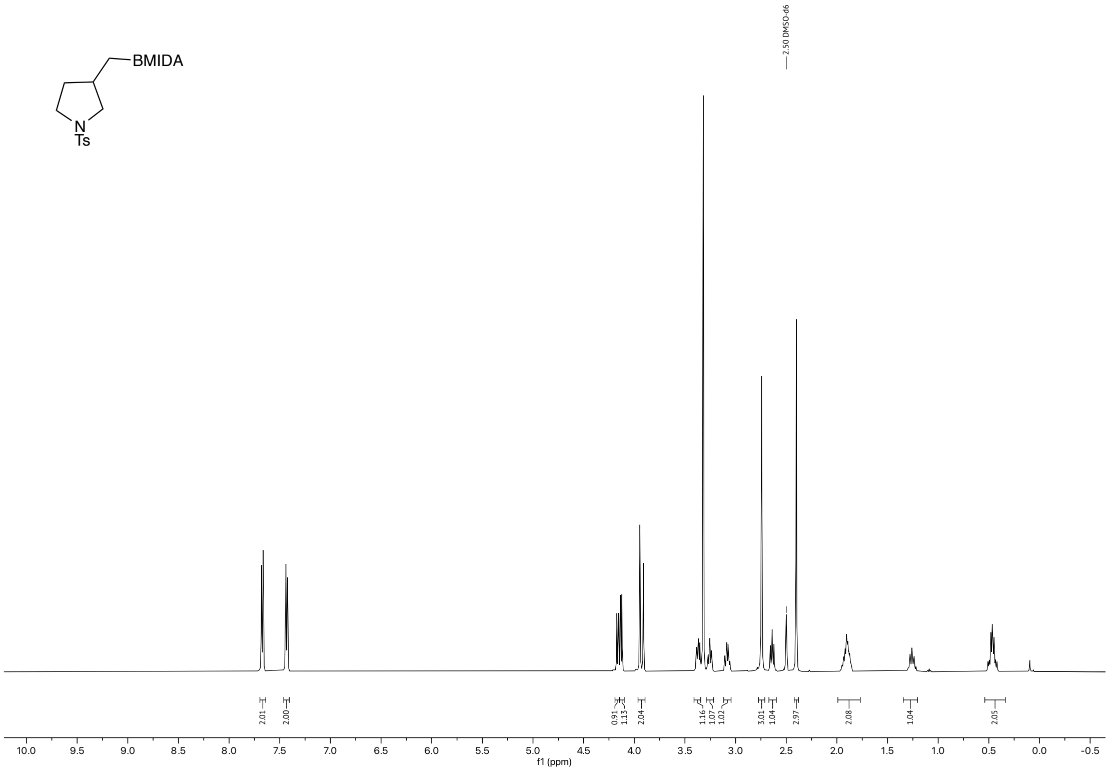

^{13}C NMR of **(7)** (126 MHz, DMSO)

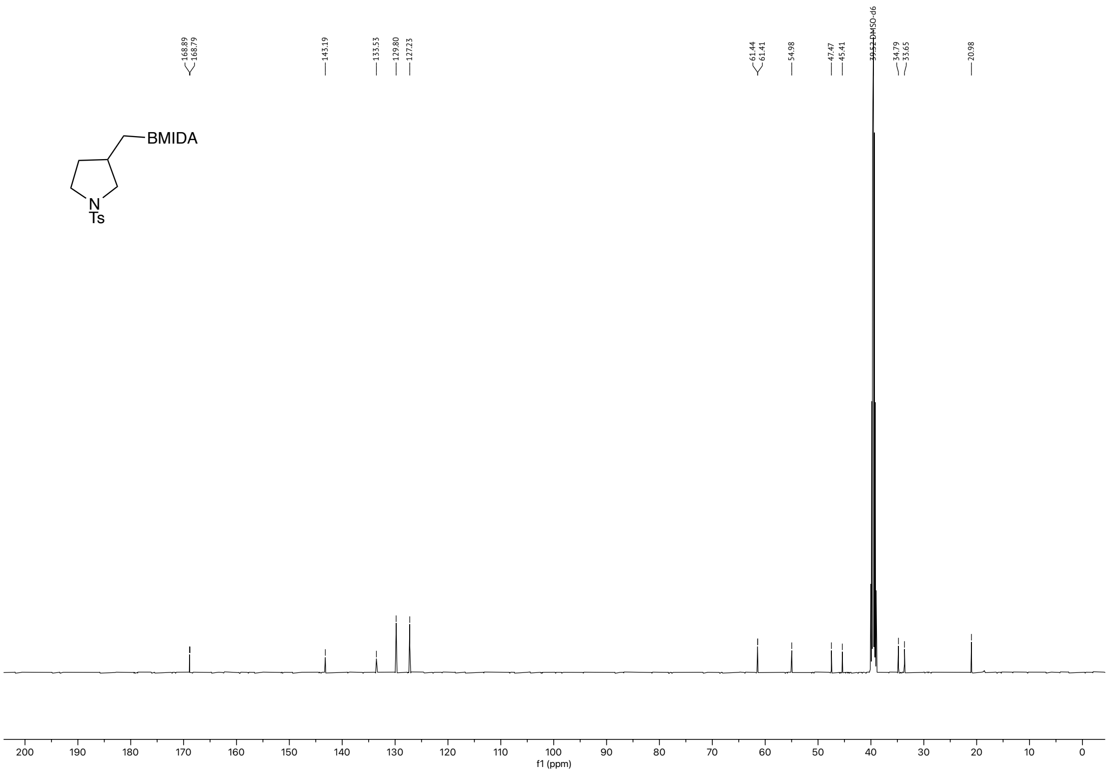

^{11}B NMR of **(7)** (160 MHz, DMSO)

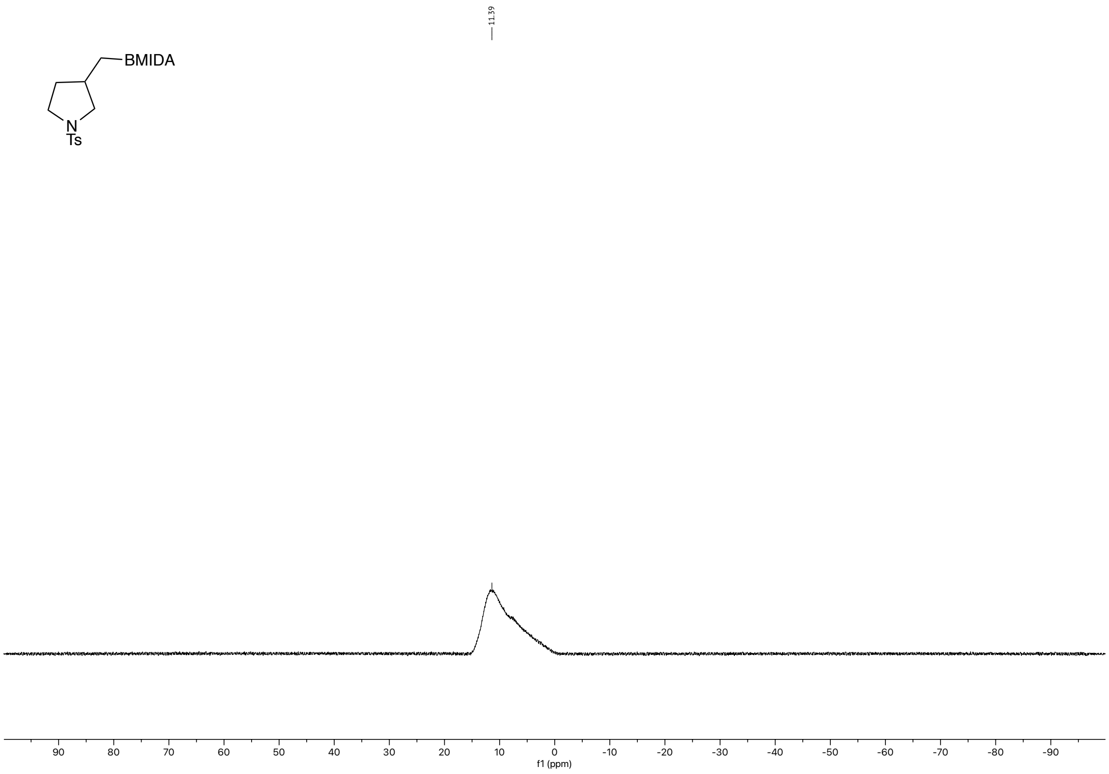

HSQC of **(7)** (500 MHz, DMSO)

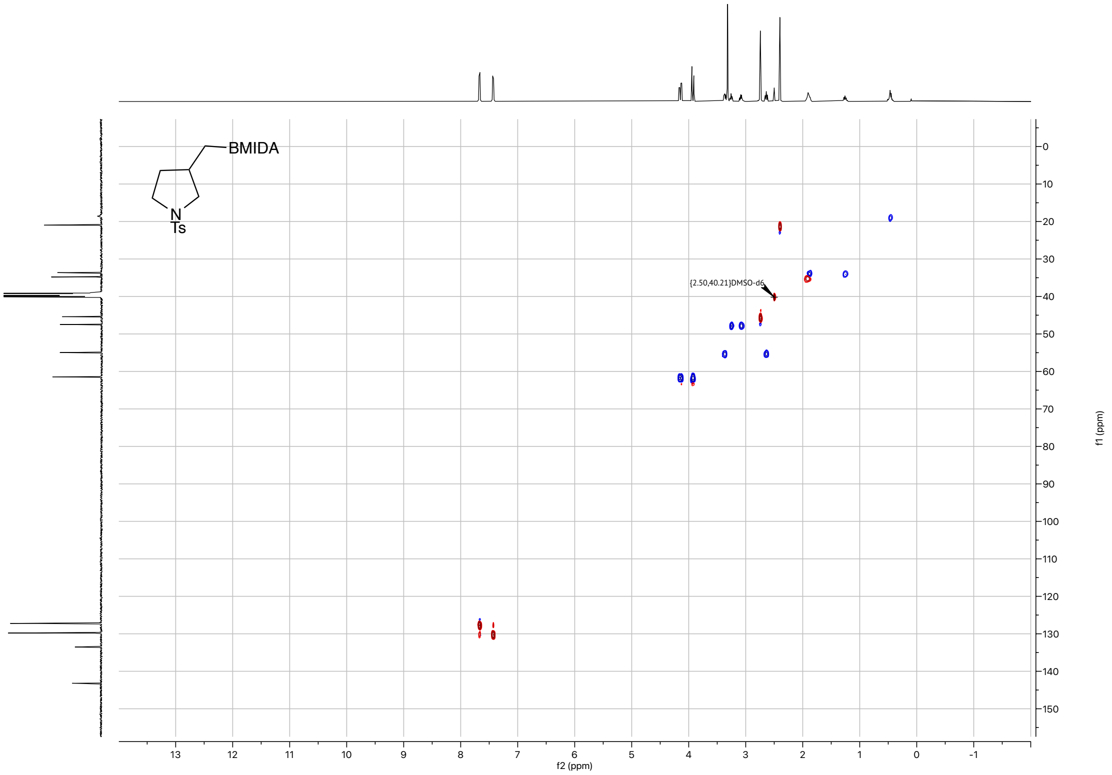

HMBC of **(7)** (500 MHz, DMSO)

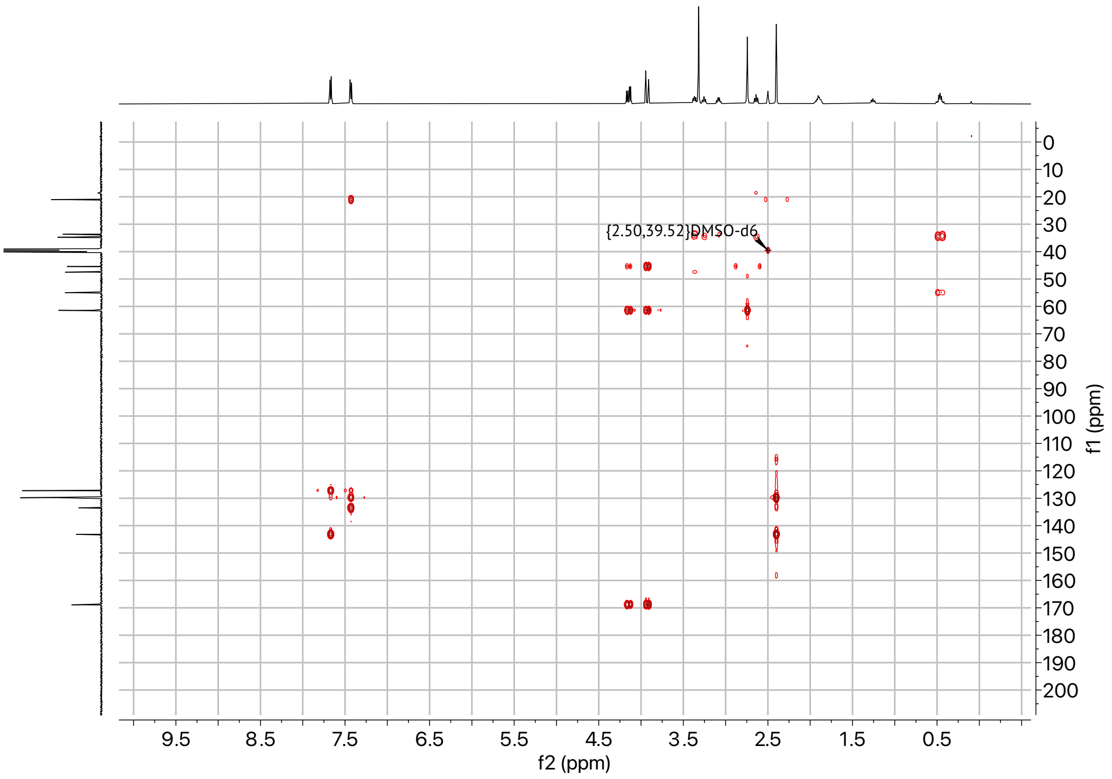

COSY of **(7)** (500 MHz, DMSO)

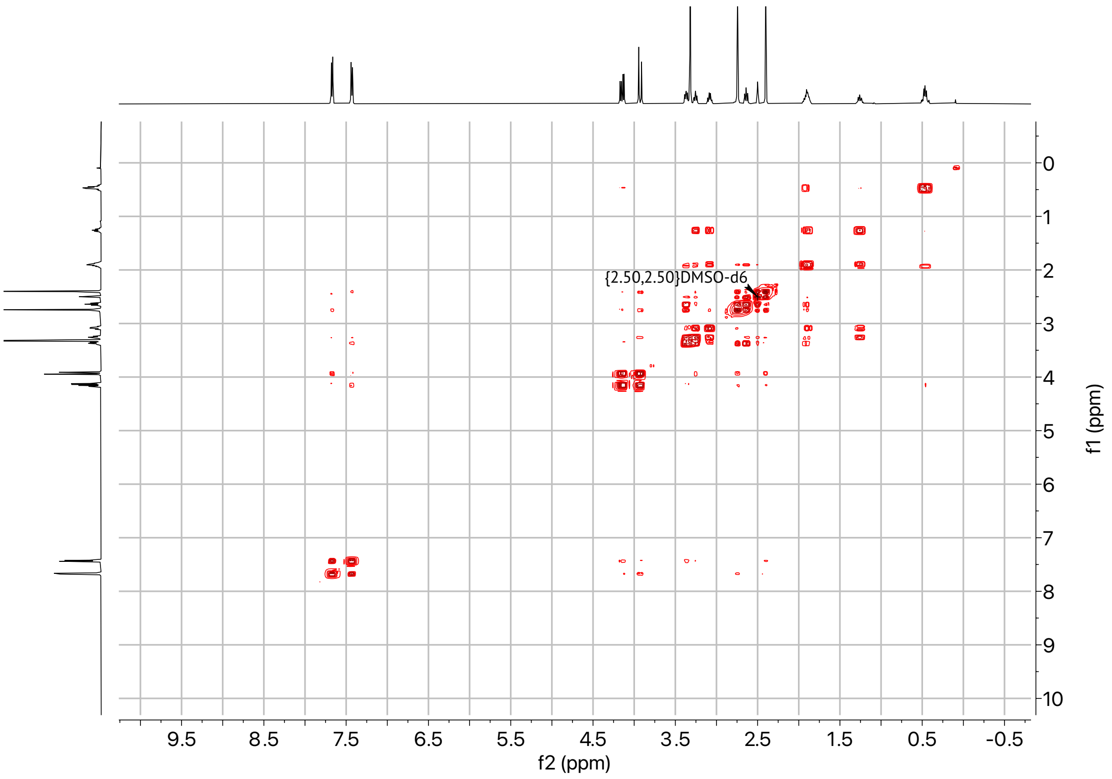

References

[1] H. Q. Cao, H. N. Liu, Z. Y. Liu, B. Qiao, F. G. Zhang, J. A. Ma, *Org. Lett.* **2020**, *22*, 6414-6419.

[2] S. R. Katamreddy, A. J. Carpenter, C. E. Ammala, E. E. Boros, R. L. Brashear, C. P. Briscoe, S. R. Bullard, R. D. Caldwell, C. R. Conlee, D. K. Croom, S. M. Hart, D. O. Heyer, P. R. Johnson, J. A. Kashatus, D. J. Minick, G. E. Peckham, S. A. Ross, S. G. Roller, V. A. Samano, H. R. Sauls, S. M. Tadepalli, J. B. Thompson, Y. Xu, J. M. Way, *J. Med. Chem.* **2012**, *55*, 10972-10994.

[3] T. B. Hamby, M. J. LaLama, C. S. Sevov, *Science* **2022**, *376*, 410-416.

[4] H. Zhao, V. D. Cuomo, J. A. Rossi-Ashton, D. J. Procter, *Chem* **2024**, *10*, 1240-1251.

[5] D. Sarkar, M. K. Ghosh, N. Rout, *Org. Biomol. Chem.* **2016**, *14*, 7883-7898.

[6] H. M. Huang, P. Bellotti, P. M. Pfluger, J. L. Schwarz, B. Heidrich, F. Glorius, *J. Am. Chem. Soc.* **2020**, *142*, 10173-10183.

[7] A. Elmabruk, B. Das, D. Yedlapudi, L. Xu, T. Antonio, M. E. A. Reith, A. K. Dutta, *ACS Chem. Neurosci.* **2019**, *10*, 396-411.

[8] W. R. Bowman, M. R. Elsegood, T. Stein, G. W. Weaver, *Org. Biomol. Chem.* **2007**, *5*, 103-113.

[9] R. Pilli, K. Selvam, B. S. S. Balamurugan, V. Jose, R. Rasappan, *Org. Lett.* **2024**, *26*, 2993-2998.

[10] Y. Liu, C. Zhou, M. Jiang, B. A. Arndtsen, *J. Am. Chem. Soc.* **2022**, *144*, 9413-9420.

[11] A. S. Dudnik, G. C. Fu, *J. Am. Chem. Soc.* **2012**, *134*, 10693-10697.

[12] H. Someya, H. Yorimitsu, K. Oshima, *Tetrahedron* **2010**, *66*, 5993-5999.

[13] C. Dai, J. M. Narayanam, C. R. Stephenson, *Nat. Chem.* **2011**, *3*, 140-145.

[14] C. A. Gould, A. L. Pace, D. W. C. MacMillan, *J. Am. Chem. Soc.* **2023**, *145*, 16330-16336.

[15] S. Nave, R. P. Sonawane, T. G. Elford, V. K. Aggarwal, *J. Am. Chem. Soc.* **2010**, *132*, 17096-17098.

[16] J. Wu, L. He, A. Noble, V. K. Aggarwal, *J. Am. Chem. Soc.* **2018**, *140*, 10700-10704.

[17] D. Mazzarella, G. Magagnano, B. Schweitzer-Chaput, P. Melchiorre, *ACS Catal.* **2019**, *9*, 5876-5880.

[18] E. K. Edelstein, A. C. Grote, M. D. Palkowitz, J. P. Morken, *Synlett* **2018**, *29*, 1749-1752.

[19] Y. Xi, J. F. Hartwig, *J. Am. Chem. Soc.* **2016**, *138*, 6703-6706.

[20] L. Caiger, C. Sinton, T. Constantin, J. J. Douglas, N. S. Sheikh, F. Julia, D. Leonori, *Chem. Sci.* **2021**, *12*, 10448-10454.

[21] R. J. Armstrong, W. Niwetmarin, V. K. Aggarwal, *Org. Lett.* **2017**, *19*, 2762-2765.

[22] B. Lansbergen, S. Tewari, I. Tomczyk, M. Seemann, H. L. Buchholz, M. Rippegarten, D. C. Cieminski, F. Julia, T. Ritter, *Angew. Chem., Int. Ed. Engl.* **2023**, *62*, e202313659.

[23] A. Fawcett, J. Pradeilles, Y. Wang, T. Mutsuga, E. L. Myers, V. K. Aggarwal, *Science* **2017**, *357*, 283-286.

[24] C. Lee, W. Yang, R. G. Parr, *Phys. Rev. B* **1988**, *37*, 785-789.

[25] A. D. Becke, *J. Chem. Phys.* **1993**, *98*, 5648-5652.

[26] a.) S. Grimme, *J. Comput. Chem.* **2004**, *25*, 1463-1473; b.) S. Grimme, J. Antony, S. Ehrlich, H. Krieg, *J. Chem. Phys.* **2010**, *132*, 154104; c.) S. Grimme, *WIREs Comput. Mol. Sci.* **2011**, *1*, 211-228; d.) S. Ehrlich, J. Moellmann, S. Grimme, *Acc. Chem. Res* **2012**, *46*, 916-926.

[27] F. Weigend, R. Ahlrichs, *Phys. Chem. Chem. Phys.* **2005**, *7*, 3297-3305.
[28] F. Weigend, *Phys. Chem. Chem. Phys.* **2006**, *8*, 1057-1065.
[29] a.) A. Klamt, G. Schüürmann, *J. Chem. Soc. Perkin Trans.* **1993**, *2*; b.) J. Tomasi, M. Persico, *Chem. Rev.* **1994**, *94*, 2027-2094; c.) J. Andzelm, C. Kölmel, A. Klamt, *J. Chem. Phys.* **1995**, *103*, 9312-9320; d.) V. Barone, M. Cossi, *J. Phys.Chem. A* **1998**, *102*, 1995-2001; e.) M. Cossi, N. Rega, G. Scalmani, V. Barone, *J. Comput. Chem.* **2003**, *24*, 669-681.
[38] Gaussian 16, revision C.01 (Gaussian Inc., 2019).
[39] C. Y. Legault, CYLview20 (Université de Sherbrooke, 2020); www.cylview.org
